# Supplementary material for: Pushing redox potentials to highly positive values using inert fluorobenzenes and weakly coordinating anions
Source: Nat Commun. 2024 Aug 7;15:6721. doi: 10.1038/s41467-024-50669-3 (PMC11306567; doi:10.1038/s41467-024-50669-3)
Supplement: Supplementary file 1 — Supplementary Information [file 41467_2024_50669_MOESM1_ESM.pdf]

Supplementary Information

to

"Pushing redox potentials to highly positive values using inert fluorobenzenes and weakly coordinating anions"

Christian Armbruster,[1#] Malte Sellin,[1#] Matthis Seiler,[1] Tanja Würz[1], Friederike Oesten,[1] Maximilian Schmucker,[1] Tabea Sterbak,[1] Julia Fischer,[1] Valentin Radtke,[1] Johannes Hunger,[2]* and Ingo Krossing[1*]

[1] Institut für Anorganische und Analytische Chemie and Freiburger Materialforschungszentrum (FMF), Albert-Ludwigs-Universität Freiburg, Albertstr. 21, 79104 Freiburg, Germany.

[2] Molecular Spectroscopy Department, Max-Planck-Institut for Polymer Research, Ackermannweg 10, 55128 Mainz, Germany.

* email: hunger@mpip-mainz.mpg.de; krossing@uni-freiburg.de.

[#] These authors contributed equally

Table of Contents

1 **Supplementary Note 1** **4**

2 **Supplementary Note 2** **7**

2.1 General Conditions 7

2.2 Methods 8

2.2.1 NMR Spectroscopy 8

2.2.2 Vibrational Spectroscopy 9

2.2.3 Computational details 9

2.2.4 Single Crystal X-Ray Diffraction 9

2.2.5 DSC measurement 10

2.2.6 Dielectric Relaxation Spectroscopy 10

3 **Supplementary Note 3** **11**

3.1 Procedure of Measurements 11

3.2 Remarks 12

3.2.1 The Use of $E_{1/2}$ and $E°´$ 12

3.2.2 Correction of the Fc compartment Reference 13

3.3 Electrochemical Windows 25

3.3.1 Determination of the Electrochemical Window 25

3.3.2 Electrochemical Windows of the Investigated Solvents 25

3.3.3 Illustrated ECWs of Fluorinated Arenes 27

3.3.4 Illustrated ECWs of Selected Solvents 30

3.4 Measurements of Redox Systems in Fluorinated Arenes Using the Fc-Compartment Reference 34

3.4.1 NO^+(S)/NO 34

3.4.2 ($[TBPA]^{\cdot+}$/TBPA) S 40

3.4.3 ($[anthracene^F]^+$/$anthracene^F$) 4FB: Perfluoroanthracene ($Anthracene^F$) 44

3.4.4 ($[phenanthrene^F]^+$/$phenanthene^F$) 4FB: Perfluorophenanthrene ($Phenanthrene^F$) 46

3.5 Measurements of Redox Systems in Fluorinated Arenes Using the Ag-compartment Reference 48

3.5.1 (Fc^+/Fc) S 48

3.5.2 NO^+(S)/NO 51

3.5.3 Triangular Born-Fajans-Haber-Cycles of the Redox Systems NO^+/NO – Ag^+/Ag – Fc^+/Fc 54

3.5.4 Comparison of the redox potentials of NO^+(S)/NO and Ag^+(S)/Ag versus ((Fc^+/Fc) S) in the fluorinated arenes 56

3.6 Measurements of Redox Systems in Selected Solvents Using the Fc-Compartment Reference 57

3.6.1 NO^+(S)/NO 57

3.6.2 ($[TBPA]^{\cdot+}$/TBPA) S 67

3.7 Measurements of Redox Systems in Selected Solvents Using the Ag-Compartment Reference 70

3.7.1 (Fc^+/Fc) S 70

3.7.2 NO^+(S)/NO 74

3.7.3 Triangular Born-Fajans-Haber-Cycles of the Redox Systems NO^+/NO – Ag^+/Ag – Fc^+/Fc 76

3.8 Reactions of Ag[pf] with $anthracene^F$- and $anthracene^{Hal}$-solution in 4FB 78

4 **Supplementary Note 4** **79**

4.1 General Remarks 79

4.1.1 General Remarks towards the used DFT functional 79

4.1.2 Comparison between the two possible solvation models CPCM and COSMO-RS 80

4.1.3 Input file for Ag related single point calculations 81

4.2 Summarization of all quantum chemical calculations results *82*
4.2.1 Calculation of the DLPNO-CCSD(T)/CBS values 87
4.2.2 Calculation of $H°_{(solv)}$ 90

4.3 Silver ion solvent S-complexation and [pf]⁻ ion-pairing enthalpies and Gibbs energies in the gas phase (g) and in S. *94*

4.4 NO^+ ion solvent S-complexation enthalpies and Gibbs energies in the gas phase (g) and in S. *96*

4.5 Comparison of the Half-Wave Potentials of the Innocent Deelectronators with their Ionization Energies *98*

5 Supplementary Note 5 **100**

5.1 Neat solvents at 298 K *103*

5.2 Temperature dependent relaxation of neat solvents *105*

5.3 Relaxation of 1:1 mixtures *106*

6 Supplementary Note 6 **111**

7 Supplementary Note 7 **112**

8 Supplementary Note 8 **118**

8.1 Original Measurement data *118*

8.2 DSC Diagram *121*

9 Supplementary Note 9 **122**

10 Supplementary Note 10 **124**

10.1 (RI-)BP86(D3BJ)/def2-TZVPP level of theory *124*
10.1.1 Solvent structures 124
10.1.2 $[NO(S)_1]^+$ structures 133
10.1.3 $[NO(S)_2]^+$ structures 142
10.1.4 Ag^+ related structures 156
10.1.5 Further structures 234

10.2 EDA-NOCV results *242*

11 Supplementary References **243**

1 Supplementary Note 1

Supplementary Table 1: Abbreviations and their respective meaning.

Abbreviation	Meaning
[*al-f-al*]$^-$	$[F\{Al(OC(CF_3)_3)_3\}_2]^-$
[*pf*]$^-$	$[Al\{OC(CF_3)_3\}_4]^-$
$\{[Cation[Anion]]\}_{ip.}$	Ion-paired Cation/Anion pair
°C	Degree Celsius
1FB	Fluorobenzene
2FB	1,2-difluorobenzene
3FB	1,2,3-trifluorobenzene
4FB	1,2,3,4-tetrafluorobenzene
5FB	Pentafluorobenzene
6FB	Hexafluorobenzene
Å	Ångstrøm (10^{-10} metres)
AN	Acetonitrile
ATR	Attenuated total reflection
br. s. (NMR)	Broad singulett
CBS	Complete basis set
Cc	Cobaltocene
Cc^+	Cobaltocenium
CE	Counter electrode
cm^{-1}	Reciprocal centimeter
COSMO-RS	Conductor-like screening model for real solvents
CPCM	Conductor-like polarizable continuum model
CV	Cyclic voltammogram
D	Diffusion constant
DCE	1,2-dichloroethane
DCM	Dichloromethane
DFT	Density functional theory
DMF	Dimethylformamide
DRS	Dielectric relaxation spectroscopy
DSC	Differential scanning calorimetry
$E°$	Standard potential
$E°'$	Formal potential
$E_{1/2} = E_{mid}$	Half-wave potential; mid peak potential
E_A	Activation energy
ECW	Electrochemical window
E_m	Measured potential
E_{pos}	Positive oxidation potential
E_{pa}	Anodic peak potential
E_{pc}	Cathodic peak potential
eq.	Equation
E_{neg}	Negative reduction potential
et al.	*et alia*
Et_2O	Diethyl ether
E_{WE}	Potential of the working electrode
F	Faraday constant
Fc	Ferrocene
Fc^+	Ferrocenium

Supplementary Table 1, continued.

Abbreviation	Meaning
FTIR (FT-IR)	Fourier-transform infrared spectroscopy
FT-Raman	Fourier-transform Raman spectroscopy
g	Gas phase
$G°$	Gibbs Energy
GHz	Gigahertz
$H°$	Standard enthalpy
Hz	Hertz
i.e.	*Id est*
I_p	Peak current
I_{pa}	Anodic peak current
I_{pc}	Cathodic peak current
IUPAC	International Union of Pure and Applied Chemistry
J (NMR)	Coupling constant
K	Kelvin
k_B	Boltzmann constant
kJ	Kilojoule
ln	Natural logarithm
m (NMR)	Multiplet
m (IR and Raman)	Medium
MHz	Megahertz
mL	Mililitre
mM	Millimolar
mm	Millimetre
mmol	Millimole
mV s^{-1}	Millivolt per second
mW	Milliwatt
NMR	Nuclear magnetic resonance
Pa	Pascal (Unit)
PC	Propylene carbonate
ppm	Parts per million
ps	Picosecond
PSGE(-NMR)	Pulsed gradient spin echo
PTFE	Polytetrafluoroethylene
R	Ideal gas constant
R^2	Coefficient of determination
R^FOH	$HOC(CF_3)_3$ with $R^F = C(CF_3)_3$
RE	Reference electrode
RT	Room temperature
s (IR and Raman)	Strong
S	Solvent
s, solv.	solvated
s (NMR)	Singulett
$S°$	Standard entropy
SCE	Saturated calomel electrode
SCF	Self-consistent field
scXRD	Single crystal X-ray diffraction
t (NMR)	Triplett
T	Temperature

Supplementary Table 1, continued.

Abbreviation	Meaning
TBPA	Tris(4-bromophenyl)amine
$[TBPA]^{+}$	Tris(4-bromophenyl)ammoniumyl
THF	Tetrahydrofurane
TMS	Tetramethylsilane
V	Volt
v	Scan Rate
$\tilde{\nu}$	Wavenumber
vs (IR and Raman)	Very strong
vw (IR and Raman)	Very weak
w (IR and Raman)	Weak
WE	Working electrode
a	Activity
ε	Permittivity
$\hat{\varepsilon}(\nu)$	Complex permittivity
$\varepsilon'(\nu)$	Real part of complex permittivity
$\varepsilon''(\nu)$	Imaginary part of complex permittivity
ε_s	Low frequency limit of $\hat{\varepsilon}(\nu)$
μ_{eff}	Effective dipole moment
µmol	Micromole
τ	Relaxation time

2 Supplementary Note 2

2.1 General Conditions

All reactions and manipulations, unless otherwise stated, were carried out under exclusion of moisture and air through usage of standard Schlenk techniques and a MBraun glovebox filled with either nitrogen or argon (O_2/H_2O < 1 ppm). All glassware used in reactions have been stored in a drying oven at 180 °C overnight and were additionally dried with a heat gun prior to usage. All solvents were distilled from standard drying agents, degassed prior to use, and stored over CaH_2, P_4O_{10} or 3 Å molecular sieves. The solvent 1,2,3,4-tetrafluorobenzene (4FB) was additionally treated with Ag^+[*pf*]$^-$ to remove traces of less fluorinated benzenes. Due to this procedure, 4FB gets contaminated by traces of R^FOH (< 1%), but we could not observe an influence on the CV measurements by this impurity.

Supplementary Table 2: Chemicals (if given, including purity) used in this work with distributor and purification method.

Chemical	Distributor	purification method
1,2,3,4-Tetrafluorobenzene (4FB)	Apollo Scientific	Dried over CaH_2, distilled onto Ag^+[*pf*]$^-$, condensed onto molecular sieves
1,2,3-Trifluorobenzene (3FB)	Apollo Scientific	Dried over CaH_2, distilled onto molecular sieves
1,2-Dichloroethane (DCE), ≥99 %	Riedel – de Häen	Dried over CaH_2, distilled onto molecular sieves
1,2-Difluorobenzene (2FB)	Fluorochem	Dried over CaH_2, distilled onto molecular sieves
9,10-Dichlorooctafluoro-anthracene, 97 %	Sigma/Aldrich	Used as received
Acetone (GPR RECATPUR®), ≥99.5 %	VWR	None purification required for $[NBu_4]^+$[*pf*]$^-$ synthesis
Acetonitrile (AN), 99.9 %	Acros Organics	Dried over CaH_2, distilled onto molecular sieves
AgF	Fluorochem	Used as received
Ag^+[*al-f-al*]$^-$	Synthetic protocol given in the methods section	-
Anthracene^F ($C_{14}F_{10}$)	Synthetic protocol given in the methods section	-
CaH_2, ≥97.0 %	Sigma/Aldrich	Used as received
CD_2Cl_2, 99.6 %	Deutero	Dried over CaH_2, distilled onto molecular sieves
CH_2Cl_2 (DCM), 99.8 %	Sigma/Aldrich	Dried over CaH_2, distilled onto molecular sieves
N,N-dimethylformamide (DMF), 99.9 %	Acros Organics	Distillation onto molecular sieves
Diethylether (Et_2O), ≥99.8 %	Honeywell / Riedel – de Häen	Dried through purification of $Li^+[AlH_4]^-$, distilled onto molecular sieves
$Fc^+[PF_6]^-$, 97 %	Sigma/Aldrich	Used as received

Ferrocene (Fc)	Used from laboratory stock	Sublimed at 100 °C and 10^{-3} mbar.
Fluorobenzene (1FB), 99 %	Sigma/Aldrich	Dried over CaH_2, distilled onto molecular sieves
Hexafluorobenzene (6FB), 99 %	P&M Invest	Dried over CaH_2, distilled onto molecular sieves
$Li^+[AlH_4]^-$ (solid gained from 2.3 m solution in THF)	Sigma/Aldrich	Solid freshly extracted with Et_2O prior to use
Li^+[*pf*]$^-$	Used from laboratory stock	Used as received
$[NBu_4]^+[PF_6]^-$, ≥99.0 %	Sigma/Aldrich	Used as received
n-Hexane	Absolute over SPS	Dried over CaH_2, distilled onto molecular sieves
Nitromethane, ≥98.5 %	Carl Roth	Available in our group
n-Pentane	VWR	Distilled onto molecular sieves
$HOC(CF_3)_3$ (R^FOH), 98 %	Fluorochem	Drying over P_4O_{10}, condensation
NO^+[*al-f-al*]$^-$	Synthetic protocol given in the methods section	-
$NO[PF_6]$	Fluorochem	Sublimation (10^{-3} mbar, 140 °C)
Pentafluorobenzene (5FB), 99 %	ABCR	Dried over CaH_2, distilled onto molecular sieves
Perfluoroperhydro-phenanthrene	Fischer Scientific	Used as received
Phenanthrene^F ($C_{14}F_{10}$)	Synthetic protocol given in the methods section	-
Phosphorus pentoxide, ≥98.5 %	Carl Roth	Used as received
Tetrahydrofuran (THF)	Absolute over SPS	Distilled onto molecular sieves
Tris(4-bromophenyl)amine (TBPA), 98 %	J&K Scientific	Used as received

2.2 Methods

2.2.1 NMR Spectroscopy

NMR spectra were recorded at RT either on a Bruker Avance DPX 200 MHz, Bruker Avance III HD 300 MHz or Bruker Avance II Widebore 400 MHz. The Bruker BioSpin *Topspin* software package (version 4.0.8) was used for measuring, processing, and creation of the graphical representations of the spectra. For samples which needed to be measured under exclusion of moisture and air an NMR tube with J. Young PTFE valve were used, otherwise an NMR tube with standard sealing cap. The field correction of hetero nuclei was adjusted with regarding to the ^1H NMR spectra of the sample, using the Ξ-tables from IUPAC.[1] ^1H, ^{13}C and ^{29}Si NMR spectra is referenced against TMS, ^7Li NMR spectra against $LiNO_3$ (9.7 mM) in D_2O, ^{11}B NMR spectra against $BF_3 \cdot OEt_2$, ^{14}N NMR spectra against CH_3NO_2, ^{19}F NMR spectra against $CFCl_3$, ^{27}Al NMR spectra against $Al(NO_3)_3$ (1.1 mM) in D_2O. It should be noted that typically small impurities of $HOC(CF_3)_3$ were detected either in ^1H NMR in the range of 3.70 or in ^{19}F NMR at –75.0 ppm.

2.2.2 Vibrational Spectroscopy

FTIR spectra were recorded inside a nitrogen or argon filled glovebox with a Bruker *ALPHA FT-IR* spectrometer equipped with QuickSnap Eco-ATR module and ZnSe crystal. The spectra were measured at RT in the range of 4000-550 cm^{-1} with 64 scans and a resolution of 2 cm^{-1}. All IR spectra were normalized to 1 and, if not stated otherwise, a baseline correction with three iterations was performed. FT-Raman spectra were recorded with a *VERTEX 70* with Bruker *RAM II* Modul (1064 nm exciting line of a ND-YAG laser) and liquid nitrogen cooled Ge detector. The samples were flame-sealed in soda-lime glass Pasteur pipettes and were measured at RT in the range of 4000-80 cm^{-1} with up to 1000 scans and a resolution of 4 cm^{-1}. All Raman spectra were normalized to 1 and, if not stated otherwise, a baseline correction with five iterations was performed. For both type of measurements and analyses, the Bruker *OPUS* 7.5.18 software package was used. The intensities of all IR and Raman spectra are reported as follows: ≥ 0.8 = very strong (vs), ≥ 0.6 = strong (s), ≥ 0.4 = medium (m), ≥ 0.2 = weak (w), < 0.2 = very weak (vw).

2.2.3 Computational details

Geometry optimizations were performed with the Turbomole[2,3] software (v7.2 or v7.5) using the DFT functionals BP86[4,5] or B3LYP[6–8] with the def2-TZVPP[9] basis set, the resolution-of-identity (RI) approximation[10–12], dispersion correction (D3BJ)[13,14], a fine integration grid, the default SCF convergence criteria and due to radical character of the neutral NO molecule and Ag atom, in single occupation condition, to be consistent through all calculations. All structures were checked for proper spin occupancies and imaginary frequencies with *EIGER* and *AOFORCE*[15] modules. Thermal and entropic contributions to the Gibbs energy were calculated either on (RI-)BP86 or (RI-)B3LYP(D3BJ)/def2-TZVPP level of theory without scaling factor at standard conditions (298.15 K, 0.1 MPa) with the *FREEH* module. The calculated structures at (RI-)BP86(D3BJ)/def2-TZVPP level of theory were then used for single point calculations performed with Orca software[16–18] (v5.0.0/v5.0.1) using DLPNO-CCSD(T)[19–21] with cc-pVDZ, cc-pvTZ and cc-pVQZ[22–24] basis set and the corresponding auxiliary basis cc-pVDZ/C, cc-pVTZ/C and cc-pVQZ/C[25] as well as the RIJCOSX[26,27] approximation. Both SCF and PNO settings were set to tight. For silver containing structures additionally, the cc-pwCVDZ-PP, cc-pwCVTZ-PP and cc-pwCVQZ-PP[28] basis set together and the auxiliary basis cc-pwCVQZ-PP/C[29] as well as the effective core potential SK-MCDHF-RSC[30] was used, together with the frozen core option. For NO^+ related single point calculations, UHF was turned on. For silver containing single point calculations an input file is exemplary shown in section 4.1.3. For B3LYP, only the sample reaction was calculated as a single point calculation, see section 4.1.1, using the different quadruple (Q)" basis set above. The Gibbs energies of solvation were calculated using the COSMO-RS[31–33] model at BP86(D3)/def2-TZVPD//BP86(D3)/def-TZVP level of theory[34] using the fine cavity construction algorithm ($cosmo_isorad) and the COSMOThermX software (v C30_1501).[35]

2.2.4 Single Crystal X-Ray Diffraction

The data were collected on a Bruker *D8 VENTURE* dual wavelength Mo/Cu three-circle diffractometer with a microfocus sealed X-ray tube using mirror optics as monochromator and a Bruker *PHOTON III* detector. Single crystals were selected at rt under perfluoropolyalkylether oil (AB128330, ABCR GmbH & Co. KG), mounted on CryoLoops with a diameter of 0.1 to 0.2 mm and shock-cooled using an Oxford Cryostream 800 low temperature device. The data were gathered at 100(2) K using Mo K_α radiation (λ = 0.71073 Å). All data were integrated with *SAINT* (version 8.38A) and a multi-scan absorption correction using *SADABS* or *TWINABS* was applied.[36–39] The structures were solved by direct methods using *SHELXT*[40] and refined by full-matrix least-

squares methods against F^2 by *SHELXL-2018/3*[41] using the GUI software ShelXle.[42] Disordered moieties were refined using bond lengths restraints and displacement parameter restraints and were modeled with the program DSR.[43,44] The gathered data were finalized with the tool FinalCif.[45] The graphical representations of the crystal structures were generated with Olex2 (version 1.2).[46] Crystallographic data for the structures reported in this paper have been deposited with the Cambridge Crystallographic Data Centre[47]. Copies of the data can be obtained free of charge from the Cambridge Crystallographic Data Centre via www.ccdc.cam.ac.uk/structures.

2.2.5 DSC measurement

The DSC measurement was performed by A. Warmbold with a Netzsch *DSC 204F1 Phoenix* in a temperature range from –30 °C up to 10 °C with a heating rate of 0.5 K min^{-1}. The graphical representations were created with OriginPro 2020. The original data is shown in section 8 below.

2.2.6 Dielectric Relaxation Spectroscopy

The dielectric properties of the neat solvents fluorobenzene (1FB), 1,2-difluorobenzene (2FB), 1,2,3-trifuorobenzene (3FB), 1,2,3,4-tetrafluorobenzene (4FB), pentafluorobenzene (5FB), and hexafluorobenzene (6FB) were investigated. Additionally, also the 1:1 mixtures of these fluorobenzenes. All solvents were dried like already described in section 2.1 . Binary mixtures were prepared by mixing equal volumes of two fluorobenzenes. The graphical representations were created with OriginPro 2020. Complex permittivity spectra were recorded using an Anritsu *MS4647A vector network analyser* at frequencies ranging from 1 GHz to 70 GHz together with an open-ended, 1.85 mm connector based, coaxial probe.[48,49] The coaxial probe was calibrated using air, conductive silver paint, and N,N-dimethylacetamide[50] as calibration standards. The probe was partly immersed into the samples and complex permittivity spectra were calculated from the complex scattering parameter.[51,52] The experimental uncertainty is determined by the accuracy of the impedance model for the probe and is estimated to 1% of the permittivity of the calibration standard *N*,*N*-dimethylacetamide: $\Delta\varepsilon', \Delta\varepsilon'' < 0.4$.[53,54] To maintain constant temperature, the samples were placed into a double-walled sample holder connected to a Julabo-*E12 thermostat*. The temperature stability in the center of the sample was estimated to be ±1 K. Spectra for the neat fluorobenzene were recorded at 278, 283, 291, 298, 306 and 313 K and for the mixtures at 283, 298, and 313 K. All spectra were recorded twice.

3 Supplementary Note 3

3.1 Procedure of Measurements

All cyclic voltammograms were recorded in an argon filled glovebox at room temperature. A three-electrode arrangement was used with a self-manufactured Pt disc (1 mm in diameter) electrode as working electrode and a Pt mesh or a Pt wire, respectively, as counter electrode. Before each measurement, the working electrode was polished with MetaDi® diamond polishing compounds from Buehler with the smallest grid size of 0.25 µm, rinsed thoroughly with isopropyl alcohol and with the solvent under investigation.

In case of the determination of the electrochemical window (ECW) of a solvent, the solvent contained $[NBu_4]^+[pf]^-$ (100 mM) as supporting electrolyte. The reference electrode was a Pt wire immersed in a solution of ferrocene (10 mM), $Fc^+[pf]^-$ (10 mM), and $[NBu_4]^+[pf]^-$ (100 mM) as supporting electrolyte in the solvent under investigation and separated from the test solution by a glas frit, we refer to them as Fc-compartment reference (Supplementary Figure 1c).

In case of the determination of $E°'$ (or $E_{1/2}$) of a redox system the test solution contained $[NBu_4]^+[pf]^-$ (100 mM) as supporting electrolyte in the solvent under investigation and the redox system under investigation, i.e. $NO^+[pf]^-$ (10 mM) or $N(4\text{-}BrC_6H_4)_3$ (TBPA) (10 mM), respectively. The reference electrode was the same as above (Supplementary Figure 1c).

In case of the determination of $E°'(Ag^+/Ag, S)$ the reference electrode was a silver wire immersed in a solution of $Ag^+[pf]^-$ (10 mM) and $[NBu_4]^+[pf]^-$ (100 mM) as supporting electrolyte in the solvent under investigation S and separated from the test solution by a glas frit, we refer to them as Ag-compartment reference. The test solution contained $Fc^+[pf]^-$ (10 mM) or $NO^+[pf]^-$ (10 mM), respectively, and $[NBu_4]^+[pf]^-$ (100 mM) as supporting electrolyte (Supplementary Figure 1d).

The potentiostat VMP3 (Bio-LogicScience Instruments) was used, controlled via PC using the software EC-Lab (V11.21). Before each set of measurements, the resistance between working and reference electrode was determined with the ZIR-technique (implemented in EC-Lab), 85% of which were compensated during the measurements by the potentiostat. The graphical representations were created with OriginPro 2020.

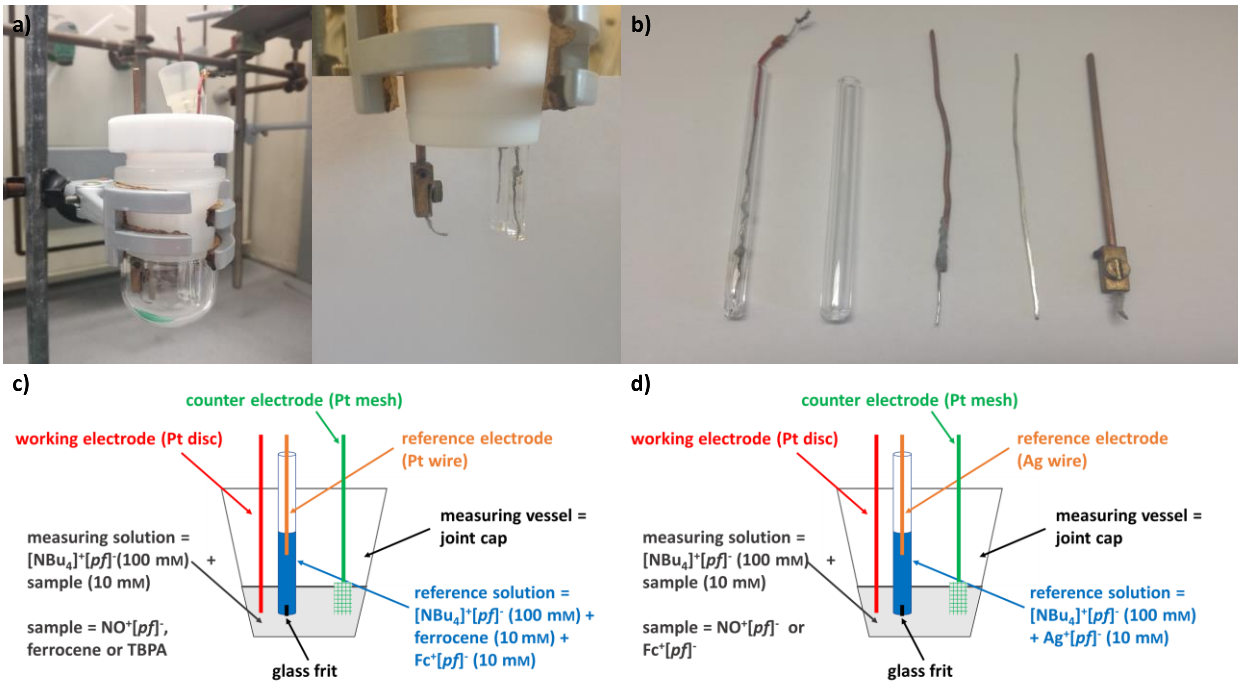

Supplementary Figure 1: a) General setup of the CV measurement; b) From left to right: Working electrode, glass compartment, Pt and Ag wire, counter electrode; c) Schematic setup measurements against Fc compartment reference and d) Schematic setup measurements against Ag compartment reference.

3.2 Remarks

3.2.1 The Use of $E_{1/2}$ and $E°´$

In this work we mainly report formal potentials, $E°´$, of redox systems. They can be obtained from the mid peak potential, E_{mid}, usually called half-wave potential, $E_{1/2}$, as $E_{1/2} = (E_{pc} + E_{pa})/2$, with E_{pc} and E_{pa} are the peak potentials of the cathodic and the anodic wave, respectively. $E°´ = E_{1/2}$ is valid with the condition that the diffusion coefficients D_{red} and D_{ox}, respectively, of the reduced and the oxidized form of the redox system are identical. Since the CVs shown in this work are quasi-reversible, it should be noted at this point that the rate constant of the heterogeneous charge transfer k^0 has almost no influence on this relation. For example, as evaluated with DigiElch by ElchSoft (v = 0.1 V s^{-1}, D_{ox} = 3·10^{-6} cm^2 s^{-1}, D_{red} = 10^{-5} cm^2 s^{-1}, α = 0.5), the peak separation with k^0 = 10 cm s^{-1} (reversibility) is 57 mV, and with k^0 = 10^{-5} (appr. border of quasi-reversibilty to irreversibility) 670 mV. The deviation of $E_{1/2}$ from $E°$ is 15 mV in the first case and 13 mV in the second. Under the additional condition that the activity coefficients of the redox species are identical, $a_{red} = a_{ox}$, the formal potential equals the standard potential, $E° = E°´$.[55] Besides that, formal potentials may include additional, known or unknown chemical equilibria affecting the activity of one or both species of the redox system.[56] In Section 3.6.1.3 we use measured data to illustrate how important a clear distinction between the specifications $E°$, $E°´$ and $E_{1/2}$ can be.

Exemplarily, we determined diffusion coefficients with PSGE-NMR technique as described elsewhere.[57] Since paramagnetic Fc^+ is invisible for NMR, we measured Cc^+ (= cobaltocinium = $[Co(C_5H_5)_2]^+$) with its almost identical size of Fc^+, and we measured diamagnetic Fc, since paramagnetic Cc is invisible in NMR. The ratio of the diffusion coefficients is inbetween 1.3 and 2.3 in all fluorinated arenes as well as in acetonitrile and propylene carbonate leading to a discrepancy between $E°´$ and $E_{1/2}$ of about 10 mV at most, thus we assign $E°´ = E_{1/2}$. Concerning large molecules and their ions, e.g. as of TBPA$^{0\text{ and }+}$, we assume the same characteristics as for the Fc^+/Fc redox pair, and therefore specify $E°´$. Concerning the small NO^+, we do not assume this

behavior, as the cation will have a rather different diffusion coefficient in the Wheland-complex compared to the neutral NO molecule, therefore we give $E_{1/2}$.

The potential value of the silver redox system is given as $E°´$ for two reasons. First, it is related to the $E°´((Fc^+/Fc)\ S)$ potential. Second, the activity coefficient of the Ag^+ ion in solutions of the investigated solvents containing 0.01 M is unknown. Since standard potential values $E°$ are given at the activity a = 1, we adjusted the measured values by the potential change which would occur from a change of the activity a = 0.01 to a = 1. Thus, the formal potential of the Ag^+/Ag redox system can be given with respect to the formal potential of the Fc^+/Fc redox system, $E°´(Ag^+(S)/Ag) = -(E_m + RT/F \cdot \ln c(Ag^+, S))$, with E_m is the measured potential $E_{1/2}((Fc^+/Fc)\ S)$ or $E_{1/2}(NO^+(S)/NO)$, respectively. Thus, the adjusting term amounts to 0.118 V.

3.2.2 Correction of the Fc compartment Reference

The potential value $E((Fc^+(10\ mM)/Fc(10\ mM))\ S)$ of each Fc-compartment reference was calibrated to $E_{1/2}((Fc^+/Fc)\ S)$ by CV measurements (Supplementary Figure 2–Supplementary Figure 8 in the fluorinated arenes, Supplementary Figure 9–Supplementary Figure 17 in the selected solvents). This was done due to unknown activity coefficients of the Fc^+ ion in the Fc-compartment reference on the one hand, and to uncertainties of laboratory work (weighing, etc.) on the other. The values given in Supplementary Table 3 (fluorinated arenes) and Supplementary Table 4 (other selected solvents) were used to correct values obtained with the Fc-compartment reference. All electrode potentials determined with the Fc-compartment reference were corrected with $E_{1/2}((Fc^+/Fc)\ S)$ values and are specified as referenced versus $E°´((Fc^+/Fc)\ S)$.

3.2.2.1 Correction Values for Fluorinated Arenes

Supplementary Table 3: Measured half-wave potentials of Ferrocene versus $E((Fc^{+}(10\ mM)/Fc(10\ mM))\ S)$ in selected fluorinated arenes.

Solvent S	v / mV s^{-1}	$E_{1/2}((Fc^{+}/Fc)\ S)$ vs. $E((Fc^{+}(10\ mM)/Fc(10\ mM))\ S)$ / V
1FB[a]	20	0.031
	50	0.034
	100	0.033
	200	0.035
1FB[b]	20	0.015
	50	0.020
	100	0.022
	200	0.029
2FB[a]	20	0.008
	50	0.011
	100	0.011
	200	0.011
2FB[b]	20	–0.047
	50	–0.045
	100	–0.044
	200	–0.044
3FB	20	–0.003
	50	–0.002
	100	–0.003
	200	–0.002
4FB	20	–0.001
	50	–0.001
	100	–0.001
	200	–0.001
5FB	20	0.052
	50	0.052
	100	0.044
	200	0.047

a) Values used for NO^{+} correction. b) Values used for $N(4\text{-}BrC_6H_4)_3$ correction.

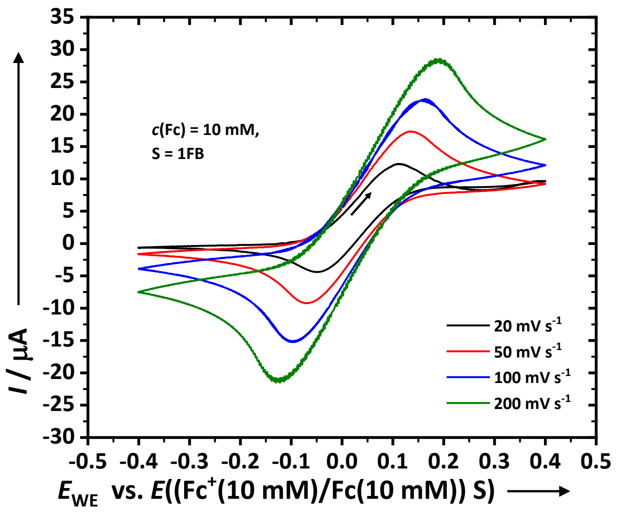

Supplementary Figure 2: 2nd cycles of ferrocene (Fc, 10 mM) in S = 1FB versus *E*((Fc^+(10 mM)/Fc(10 mM)) S) measured at four different scan rates (20, 50, 100 & 200 mV s^{-1}). These measurements were used for the correction of cyclic voltammograms of NO^+. The black arrow indicates for all scan rates the start and the direction of the 2nd cycle.

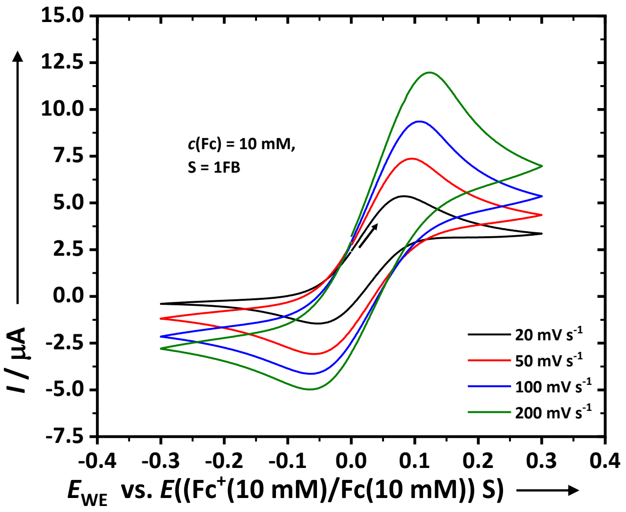

Supplementary Figure 3: 2nd cycles of ferrocene (Fc, 10 mM) in S = 1FB versus *E*((Fc^+(10 mM)/Fc(10 mM)) S) measured at four different scan rates (20, 50, 100 & 200 mV s^{-1}). These measurements were used for the correction of cyclic voltammograms of TBPA. The black arrow indicates for all scan rates the start and the direction of the 2nd cycle.

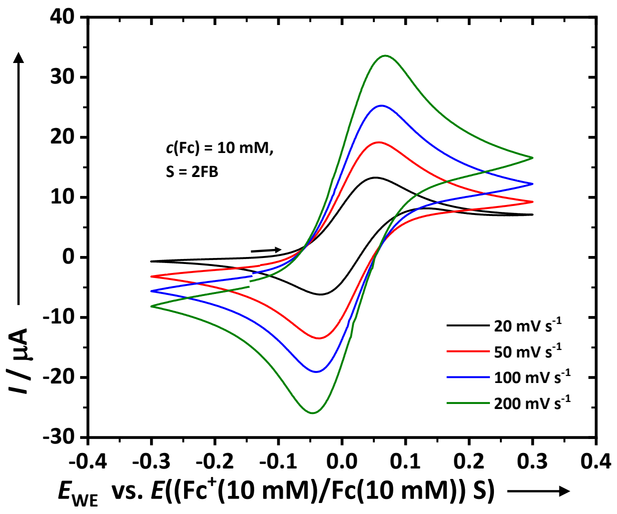

Supplementary Figure 4: 2nd cycles of ferrocene (Fc, 10 mM) in S = 2FB versus *E*((Fc$^+$(10 mM)/Fc(10 mM)) S) measured at four different scan rates (20, 50, 100 & 200 mV s^{-1}). These measurements were used for the correction of cyclic voltammograms of NO$^+$. The black arrow indicates for all scan rates the start and the direction of the 2nd cycle.

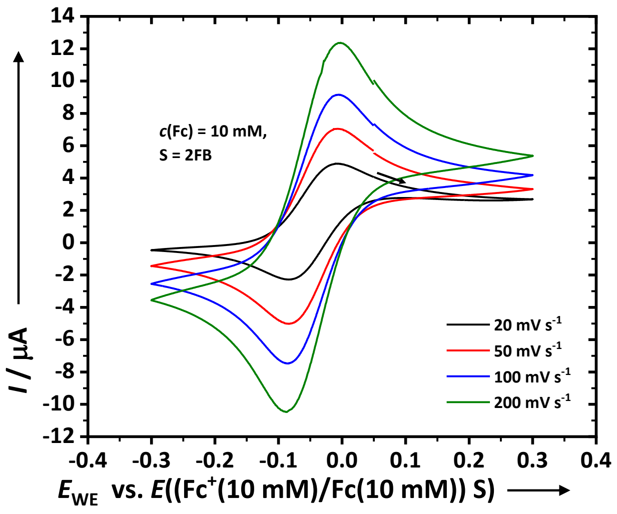

Supplementary Figure 5: 2nd cycles of ferrocene (Fc, 10 mM) in S = 2FB versus *E*((Fc$^+$(10 mM)/Fc(10 mM)) S) measured at four different scan rates (20, 50, 100 & 200 mV s^{-1}). These measurements were used for the correction of cyclic voltammograms of TBPA. The black arrow indicates for all scan rates the start and the direction of the 2nd cycle.

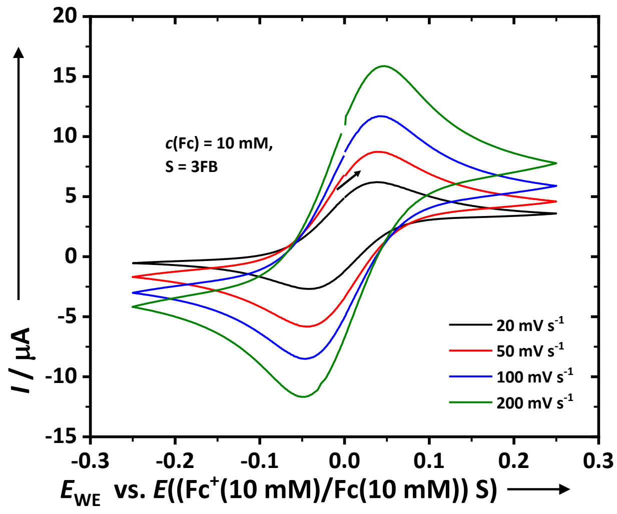

Supplementary Figure 6: 2nd cycles of ferrocene (Fc, 10 mM) in S = 3FB versus *E*((Fc^+(10 mM)/Fc(10 mM)) S) measured at four different scan rates (20, 50, 100 & 200 mV s^{-1}). The black arrow indicates for all scan rates the start and the direction of the 2nd cycle.

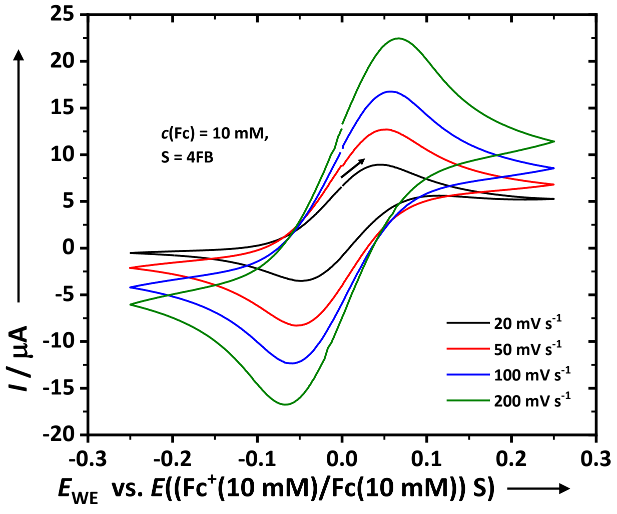

Supplementary Figure 7: 2nd cycles of ferrocene (Fc, 10 mM) in S = 4FB versus *E*((Fc^+(10 mM)/Fc(10 mM)) S) measured at four different scan rates (20, 50, 100 & 200 mV s^{-1}). The black arrow indicates for all scan rates the start and the direction of the 2nd cycle.

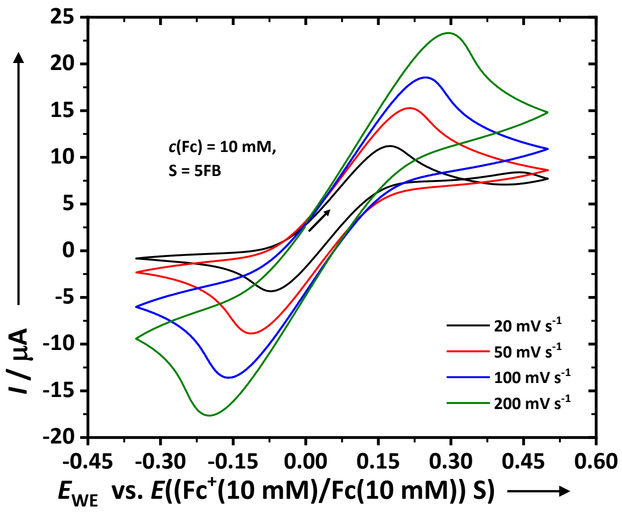

Supplementary Figure 8: 2nd cycles of ferrocene (Fc, 10 mM) in S = 5FB versus *E*((Fc^+(10 mM)/Fc(10 mM)) S) measured at four different scan rates (20, 50, 100 & 200 mV s^{-1}). The black arrow indicates for all scan rates the start and the direction of the 2nd cycle.

3.2.2.2 Correction Values for Selected Solvents

Supplementary Table 4: Measured half-wave potentials of Ferrocene versus $E((Fc^+(10\ mM)/Fc(10\ mM))\ S)$ in selected solvents at different scan rates (v).

Solvent	v / mV s^{-1}	$E_{1/2}((Fc^+/Fc)\ S)$ vs. $E((Fc^+(10\ mM)/Fc(10\ mM))\ S)$ / V
DCM[a,b]	20	0.019
	50	0.022
	100	0.021
	200	0.021
DCM[c]	100	0.014
DCM[d]	20	0.042
	50	0.054
	100	0.060
	200	0.069
DCE	20	0.014
	50	0.012
	100	0.010
	200	0.015
DMF	20	0.035
	50	0.033
	100	0.033
	200	0.034
Nitromethane	20	–0.006
	50	–0.005
	100	–0.005
	200	–0.005
PC	20	–0.019
	50	–0.017
	100	–0.018
	200	–0.020
AN	20	–0.001
	50	0.005
	100	0.005
	200	0.004
THF	20	0.017
	50	0.019
	100	0.018
	200	0.019

a) Values used for NO^+ correction. b) Values used for $N(4\text{-}BrC_6H_4)_3$ correction. c) Value used for NO^+ correction of the concentration measurements in dichloromethane. d) Value for $NO^+[PF_6]^-$ correction.

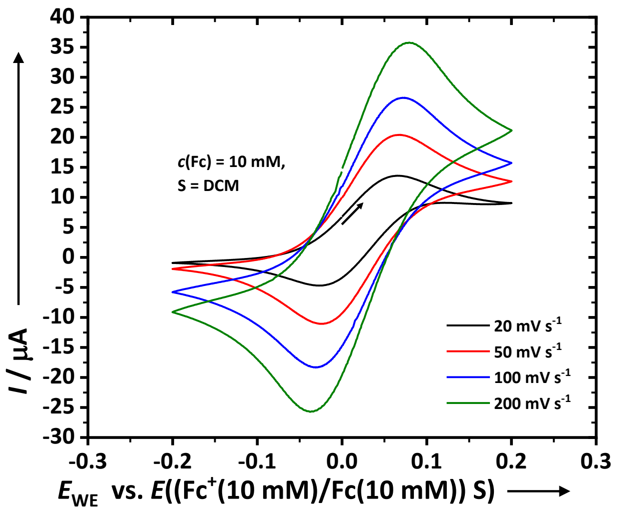

Supplementary Figure 9: 2nd cycles of ferrocene (Fc, 10 mM) in S = DCM versus *E*((Fc^+(10 mM)/Fc(10 mM)) S) measured at four different scan rates (20, 50, 100 & 200 mV s^{-1}). These measurements were used for the correction of cyclic voltammograms of $NO^+$and TBPA. The black arrow indicates for all scan rates the start and the direction of the 2nd cycle.

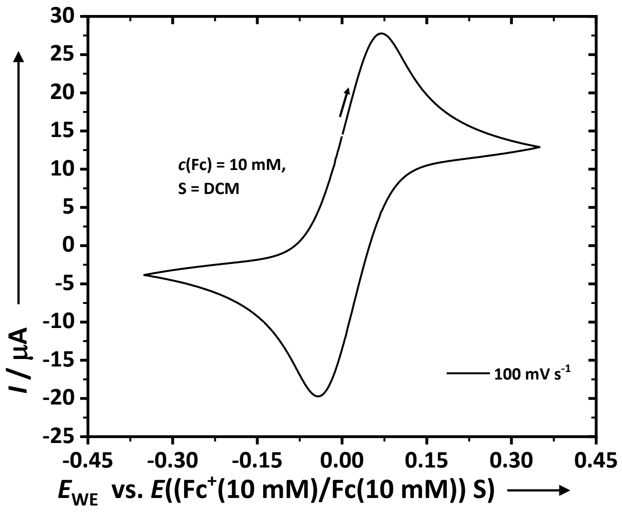

Supplementary Figure 10: 2nd cycles of ferrocene (Fc, 10 mM) in S = DCM versus *E*((Fc^+(10 mM)/Fc(10 mM)) S) measured at a scan rate of 100 mV s^{-1}. This measurement was used for the correction of cyclic voltammograms of different NO^+ concentrations. The black arrow indicates for all scan rates the start and the direction of the 2nd cycle.

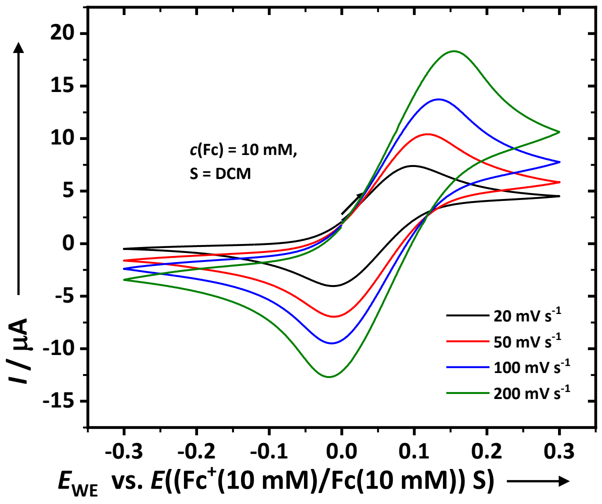

Supplementary Figure 11: 2nd cycles of ferrocene (Fc, 10 mM) in S = DCM versus *E*((Fc^+(10 mM)/Fc(10 mM)) S) measured at four different scan rates (20, 50, 100 & 200 mV s^{-1}). This measurement was used for the correction of cyclic voltammogram of $NO^+[PF_6]^-$ and the corresponding potential window of stability. The black arrow indicates the start and the direction of the 2nd cycle.

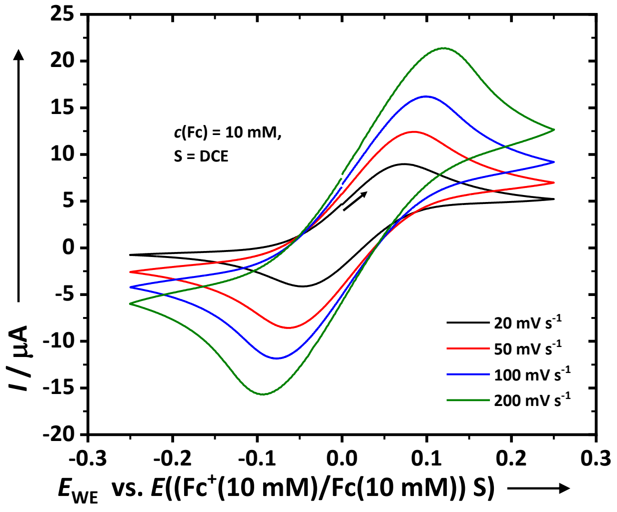

Supplementary Figure 12: 2nd cycles of ferrocene (Fc, 10 mM) in S = DCE versus *E*((Fc^+(10 mM)/Fc(10 mM)) S) measured at four different scan rates (20, 50, 100 & 200 mV s^{-1}). The black arrow indicates for all scan rates the start and the direction of the 2nd cycle.

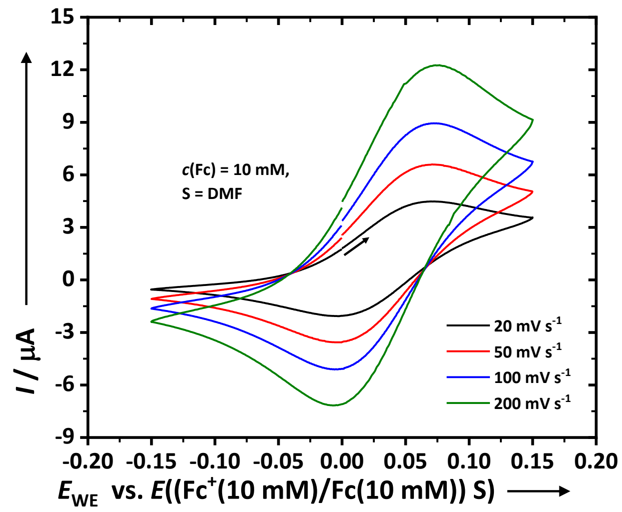

Supplementary Figure 13: 2nd cycles of ferrocene (Fc, 10 mM) in S = DMF versus $E((Fc^+(10\ mM)/Fc(10\ mM))\ S)$ measured at four different scan rates (20, 50, 100 & 200 mV s^{-1}). The black arrow indicates for all scan rates the start and the direction of the 2nd cycle.

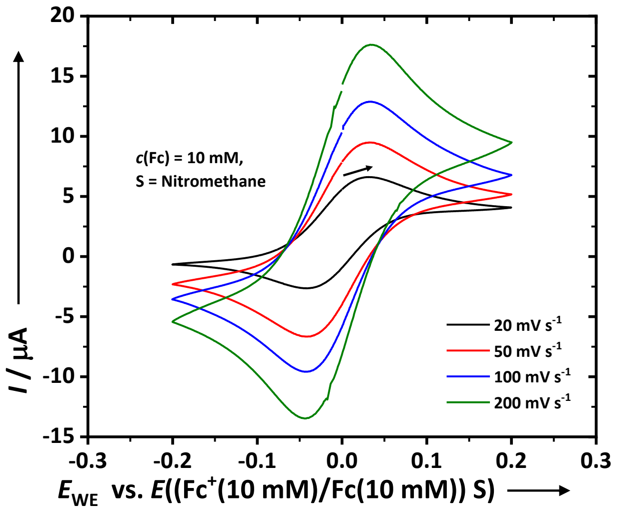

Supplementary Figure 14: 2nd cycles of ferrocene (Fc, 10 mM) in S = Nitromethane versus $E((Fc^+(10\ mM)/Fc(10\ mM))\ S)$ measured at four different scan rates (20, 50, 100 & 200 mV s^{-1}). The black arrow indicates for all scan rates the start and the direction of the 2nd cycle.

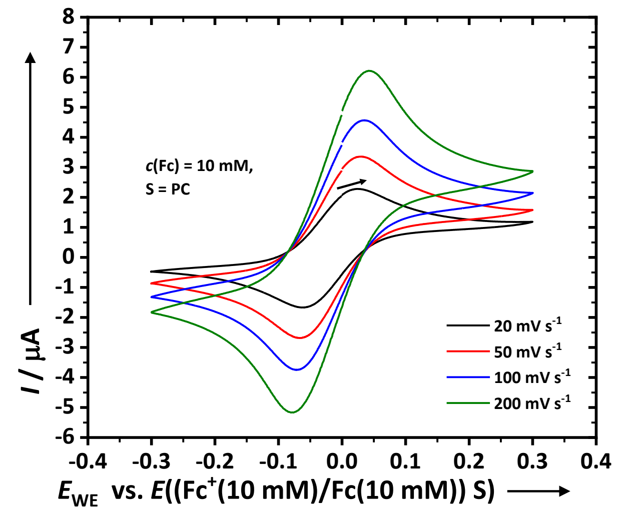

Supplementary Figure 15: 2nd cycles of ferrocene (Fc, 10 mM) in S = PC versus *E*((Fc^+(10 mM)/Fc(10 mM)) S) measured at four different scan rates (20, 50, 100 & 200 mV s^{-1}). The black arrow indicates for all scan rates the start and the direction of the 2nd cycle.

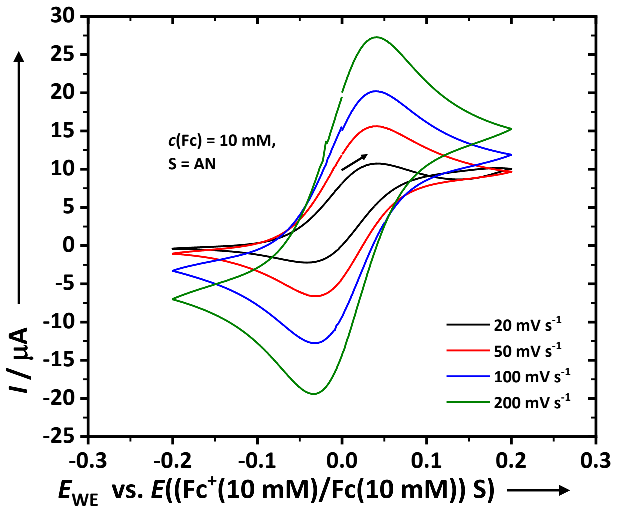

Supplementary Figure 16: 2nd cycles of ferrocene (Fc, 10 mM) in S = AN versus *E*((Fc^+(10 mM)/Fc(10 mM)) S) measured at four different scan rates (20, 50, 100 & 200 mV s^{-1}). The black arrow indicates for all scan rates the start and the direction of the 2nd cycle.

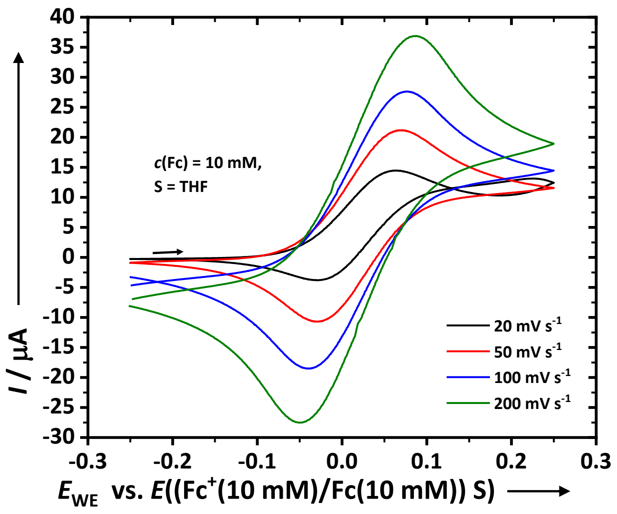

Supplementary Figure 17: 2nd cycles of ferrocene (Fc, 10 mM) in S = THF versus E((Fc$^+$(10 mM)/Fc(10 mM)) S) measured at four different scan rates (20, 50, 100 & 200 mV s^{-1}). The black arrow indicates for all scan rates the start and the direction of the 2nd cycle.

3.3 Electrochemical Windows

3.3.1 Determination of the Electrochemical Window

The values for the limits of the electrochemical window (ECW) were evaluated as follows:

1) The 2nd cycle with the scan rate of v = 100 mV s^{-1} was used.
2) Two linear regressions were made, for which the respective slope of the forward thrust of the solvent decomposition at positive and negative potential was used.
3) The intersection point of the respective linear regression with I = 0 µA was determined. These points represent the positive and negative limits.
4) Impurities within the visual boundaries were neglected, to avoid a significant underestimation of the width of the electrochemical window.

It should be mentioned that the ECW depends on the used electrode material and supporting electrolyte.[55]

3.3.2 Electrochemical Windows of the Investigated Solvents

Measurements were performed in 1FB up to 5FB and additionally for selected solvents. In further selected fluoroarenes, i.e. 6FB and 1,3-difluorobenzene, CV measurements were not feasible due to conductivity and/or solubility problems. The used reference electrode was the above described Fc-compartment reference, and all values reported here were corrected with the values given in section 3.2.2.

The graphic illustrations of the different potential windows of stability for all five fluorinated arenes and the selected solvents are given with the Supplementary Figure 18 up to Supplementary Figure 32, included is the determination of the limits E_{neg} and E_{pos} of the ECW for each solvent. The results are summarized in Supplementary Table 5.

Based on the – rather imprecise – ferrocene assumption, i.e. the assumption that the redox potential of the redox system Fc^+/Fc is independent of the solvent, all investigated fluorinated arenes except for 5FB show a similar reductive behavior. The stability towards oxidation increases – in accordance with the measured ionization energies (refer to Supplementary Table 15) – with the number of fluorine atoms. All investigated fluorinated arenes feature ECWs of 5 – 5.5 V. O´Toole *et al*. determined the ECW of 2FB to be in the range of 3.8 to 4.2 V.[58] However, they used $[NBu_4]^+[PF_6]^-$ as supporting electrolyte explaining the discrepancy to the values of Supplementary Table 5.[55] Since they used the SCE as reference electrode the limiting values cannot compared to the values given here. To our best knowledge no other ECW values of fluorinated arenes were published so far. ECW values of the selected solvents as reported by different work groups are summarized in Supplementary Table 6.

Supplementary Table 5: Summary of the relevant data for the different potential windows of stability for the fluoroarenes and selected solvents at v = 100 mV s^{-1}.

Solvent S	E_{neg} vs. $E°'$((Fc$^+$/Fc) S) / V	E_{pos} vs. $E°'$((Fc$^+$/Fc) S) / V	ECW / V
1FB	−3.18	+1.82	5.00
2FB	−3.09	+2.05	5.14
3FB	−3.02	+2.35	5.37
4FB	−3.08	+2.43	5.51
5FB	−2.37	+2.67	5.04
DCM	−2.91	+3.36	6.27
	−2.62§	+2.58§	5.20§
DCE	−2.28	+3.13	5.41
DMF	−3.60	+1.73	5.33
Nitromethane	−1.87	+3.96	5.83
PC	−3.50	+4.15	7.65
AN	−3.09	+4.59	7.68
THF	−3.60	+1.33	4.93

§= With $[NBu_4]^+[PF_6]^-$ as conducting salt.

Supplementary Table 6: Summarization of selected literature values mentioning the used conducting salt, working (WE) or reference electrode (RE) and the width of the ECW.

Solvent	Conducting salt	WE	RE	ECW / V	Literature
DCM	$[NBu_4]^+[ClO_4]^-$	Pt	SCE	3.5	[59,60]
	n/a	Pt	Fc$^+$/Fc	4.9	[61]
DCE	n/a	Pt	Fc$^+$/Fc	4.0	[61]
DMF	$[NEt_4]^+[ClO_4]^-$	Pt	SCE	4.7	[59,62]
	n/a	Pt	Fc$^+$/Fc	5.3	[61]
Nitromethane	$Li^+[ClO_4]^-$	Pt	SCE	5.4	[59,63]
	n/a	Pt	Fc$^+$/Fc	6.9	[61]
PC	$[NEt_4]^+[ClO_4]^-$	Pt	SCE	3.6	[59,64]
	n/a	Pt	Fc$^+$/Fc	6.8	[61]
AN	$[NBu_4]^+$ $[ClO_4]^-$	Pt	SCE	5.3	[59,65]
	n/a	Pt	Fc$^+$/Fc	6.5	[61]
THF	$Li^+[ClO_4]^-$	Pt	SCE	4.8	[59,62,66]
	n/a	Pt	Fc$^+$/Fc	5.4	[61]

The comparison of the literature data with our data shows a clear influence of the supporting electrolyte on the ECWs, i.e. the accessible potential range of the solvents. With the $[NBu_4]^+[pf]^-$ supporting electrolyte we used, this is considerably extended, with the exception of the solvent nitromethane.

Exemplarily, Supplementary Figure 26 shows the effect of replacing the supporting electrolyte anion from $[PF_6]^-$ to $[pf]^-$ within DCM. In this case, the solutions reduction stability is increased slightly, but the oxidation stability is enhanced considerably. In total, the ECW is widened by more than 1 V.

We therefore recommend the use of the $[pf]^-$ ion as anion of the supporting electrolyte but also as counter ion of cationic species of redox systems, particularly if oxidation processes are being investigated.

3.3.3 Illustrated ECWs of Fluorinated Arenes

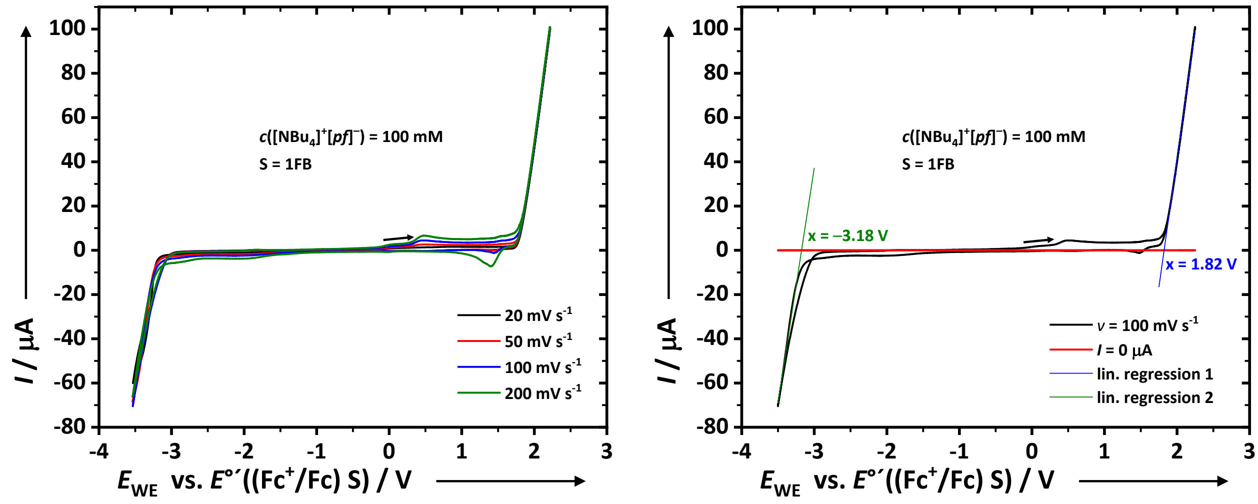

Supplementary Figure 18: Left: 2nd cycle of S = neat 1FB with dissolved $[NBu_4]^+[pf]^-$ (100 mM) versus $E°'$((Fc+/Fc) S) measured at four different scan rates (20, 50, 100 & 200 mV s⁻¹). Right: Determination of the limits from the ECW for 1FB from the CV measurement at v = 100 mV s^{-1}. The black arrows indicate, in both illustrations, the start and the direction of each cycle shown.

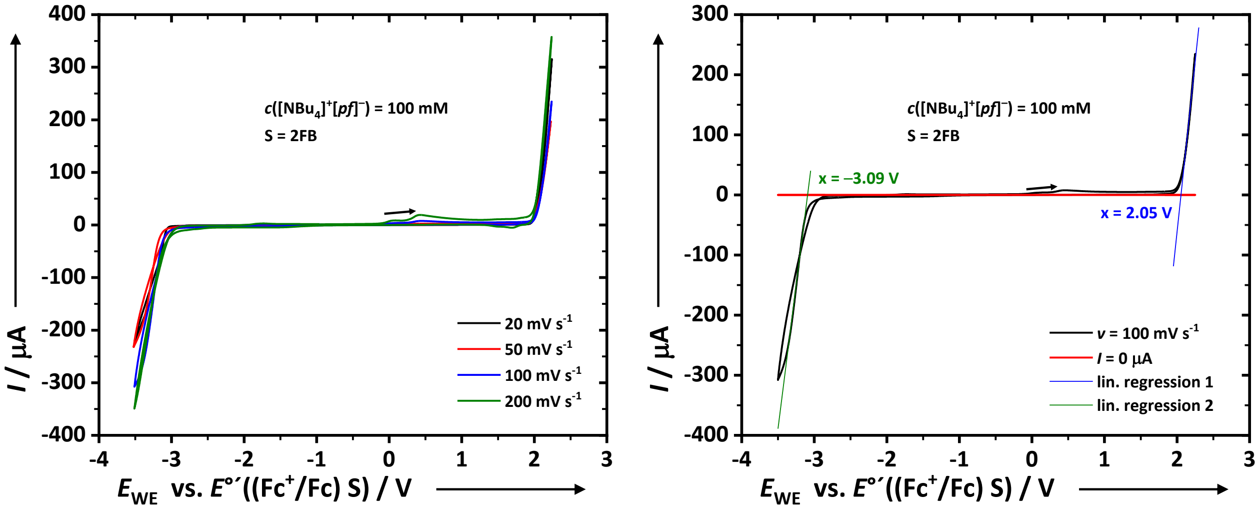

Supplementary Figure 19: Left: 2nd cycle of S = neat 2FB with dissolved $[NBu_4]^+[pf]^-$ (100 mM) versus $E°'$((Fc+/Fc) S) measured at four different scan rates (20, 50, 100 & 200 mV s^{-1}). Right: Determination of the limits from the ECW for 2FB from the CV measurement at v = 100 mV s^{-1}. The black arrows indicate, in both illustrations, the start and the direction of each cycle shown.

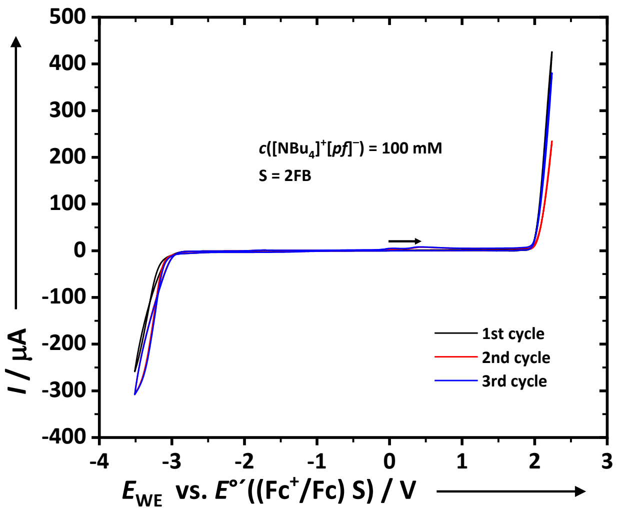

Supplementary Figure 20: All cycles of the CV measurement of S = neat 2FB with dissolved $[NBu_4]^+[pf]^-$ (100 mM) versus $E°´$((Fc$^+$/Fc) S) measured at v = 100 mV s^{-1}, as an example for the complex nature of solvent decomposition and the resulting differences through the different cycles. The black arrow indicates the direction of each cycle shown.

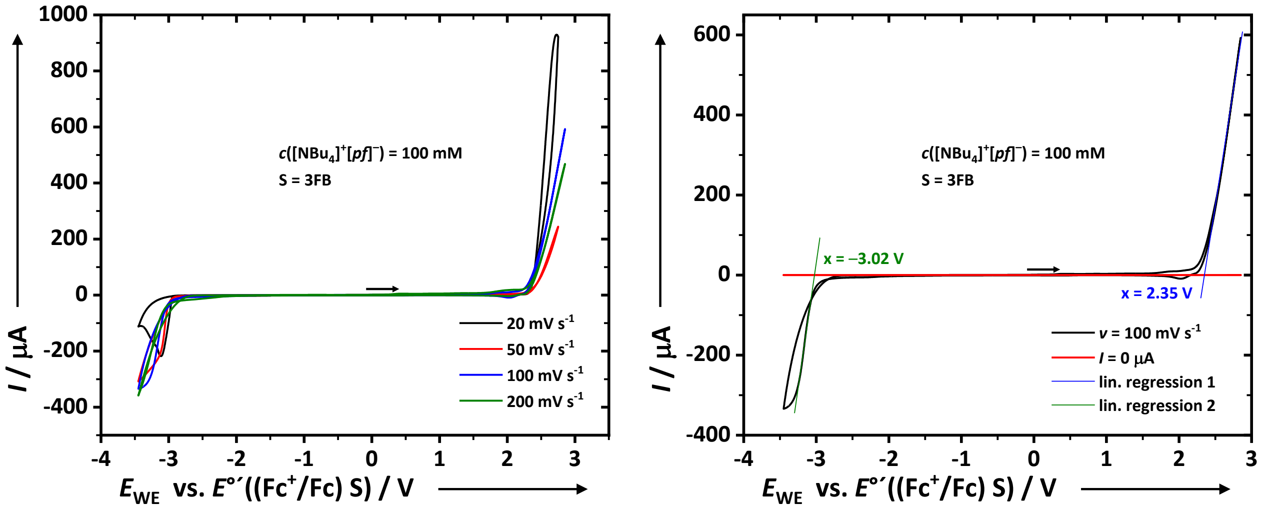

Supplementary Figure 21: Left: 2nd cycle of S = neat 3FB with dissolved $[NBu_4]^+[pf]^-$ (100 mM) versus $E°´$((Fc$^+$/Fc) S) measured at four different scan rates (20, 50, 100 & 200 mV s^{-1}). Right: Determination of the limits from the ECW for 3FB from the CV measurement at v = 100 mV s^{-1}. The black arrows indicate, in both illustrations, the start and the direction of each cycle shown.

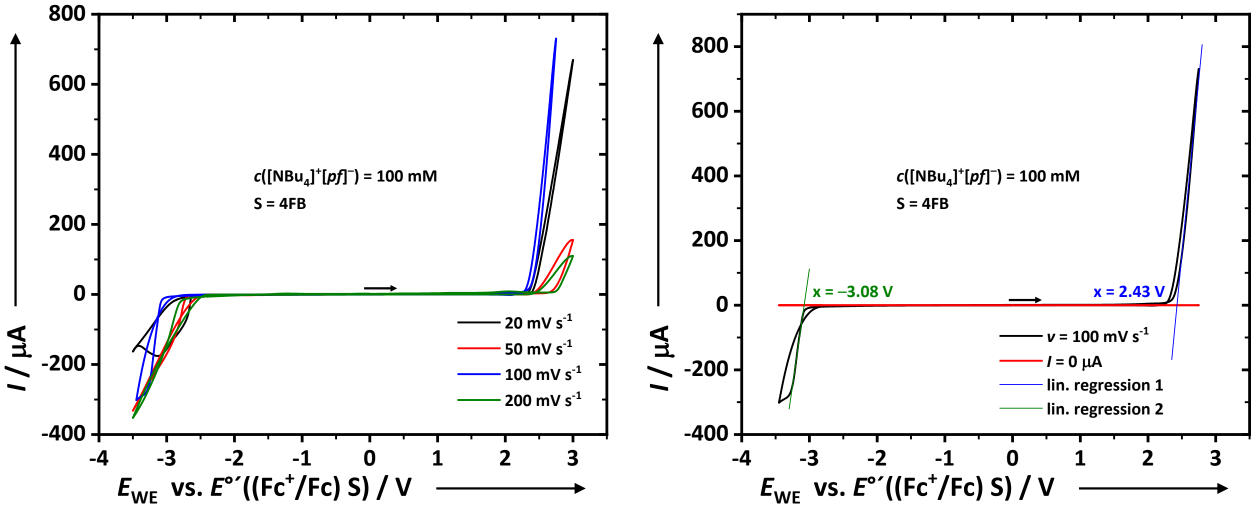

Supplementary Figure 22: Left: 2nd cycle of S = neat 4FB with dissolved $[NBu_4]^+[pf]^-$ (100 mM) versus $E°´$((Fc+/Fc) S) measured at four different scan rates (20, 50, 100 & 200 mV s^{-1}). Right: Determination of the limits from the ECW for 4FB from the CV measurement at *v* = 100 mV s^{-1}. The black arrows indicate, in both illustrations, the start and the direction of each cycle shown.

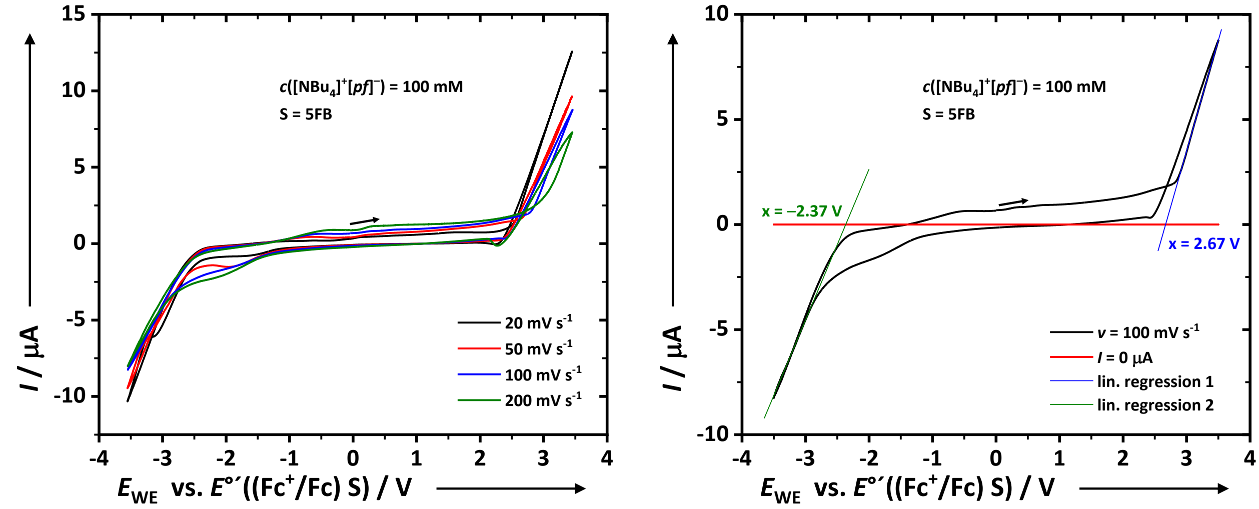

Supplementary Figure 23: Left: 2nd cycle of S = neat 5FB with dissolved $[NBu_4]^+[pf]^-$ (100 mM) versus $E°´$((Fc+/Fc) S) measured at four different scan rates (20, 50, 100 & 200 mV s^{-1}). Right: Determination of the limits from the ECW for 5FB from the CV measurement at *v* = 100 mV s^{-1}. The black arrows indicate, in both illustrations, the start and the direction of each cycle shown.

3.3.4 Illustrated ECWs of Selected Solvents

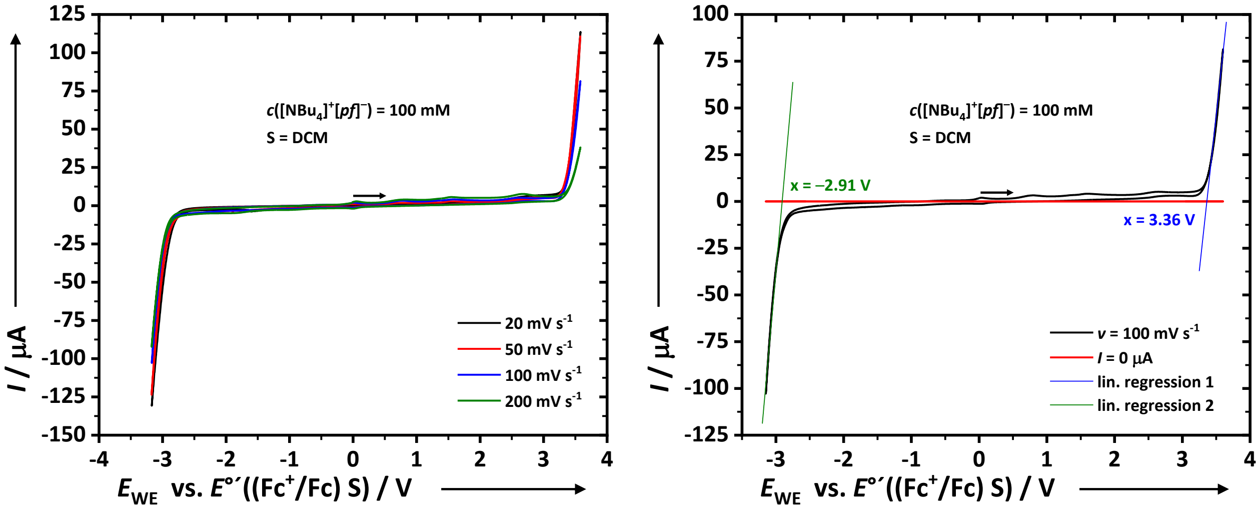

Supplementary Figure 24: Left: 2nd cycle of S = neat DCM with dissolved $[NBu_4]^+[pf]^-$ (100 mM) versus $E^{\circ\prime}$((Fc$^+$/Fc) S) measured at four different scan rates (20, 50, 100 & 200 mV s^{-1}). Right: Determination of the limits from the ECW for DCM from the CV measurement at v = 100 mV s^{-1}. The black arrows indicate, in both illustrations, the start and the direction of each cycle shown.

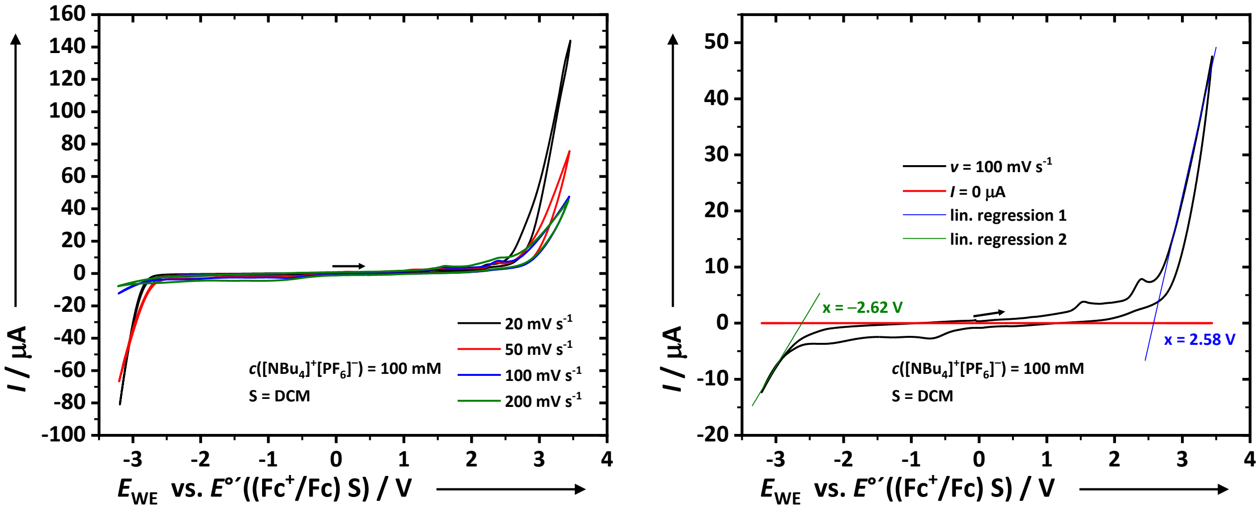

Supplementary Figure 25: Left: 2nd cycle of S = neat DCM with dissolved $[NBu_4]^+[PF_6]^-$ (100 mM) versus $E^{\circ\prime}$((Fc$^+$/Fc) S) measured at four different scan rates (20, 50, 100 & 200 mV s^{-1}). Right: Determination of the limits from the ECW for DCM from the CV measurement at v = 100 mV s^{-1}. The black arrows indicate, in both illustrations, the start and the direction of each cycle shown.

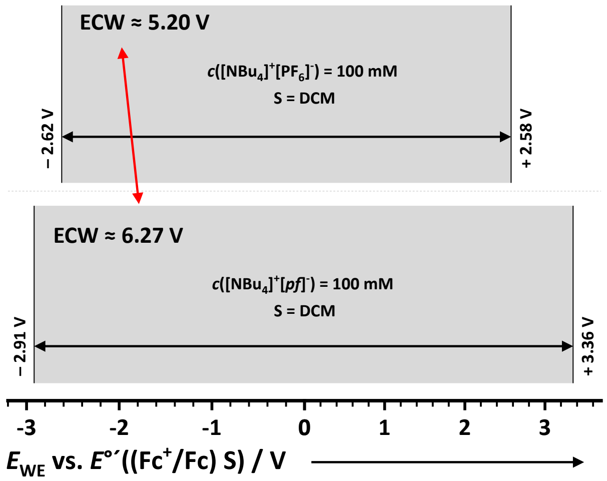

Supplementary Figure 26: Comparison of the width of the ECWs with $[NBu_4]^+[pf]^-$, respectively $[NBu_4]^+[PF_6]^-$ as conducting salt in S = neat DCM.

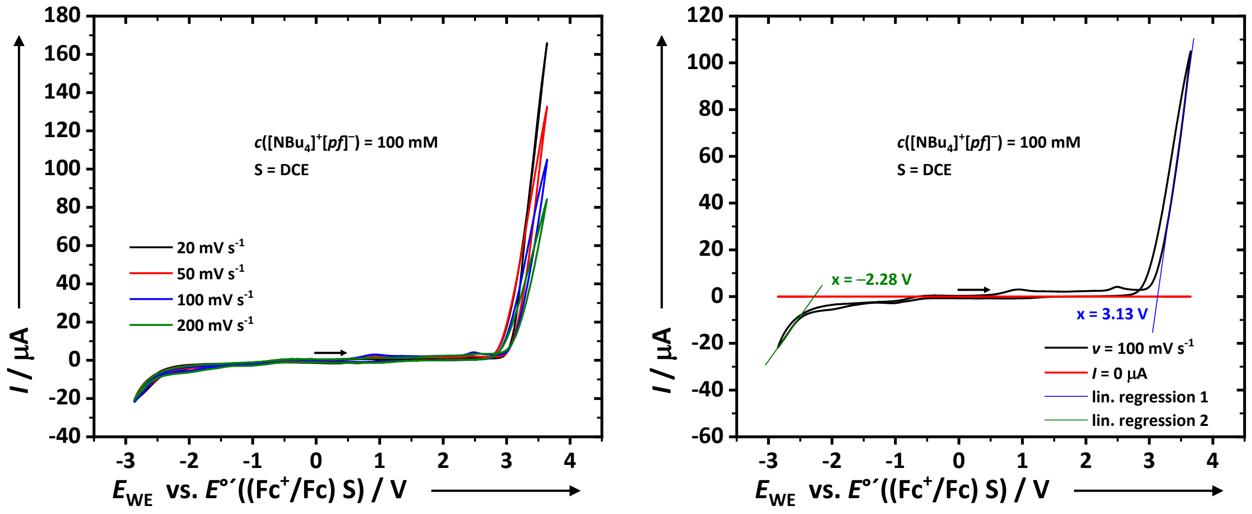

Supplementary Figure 27: Left: 2nd cycle of S = neat DCE with dissolved $[NBu_4]^+[pf]^-$ (100 mM) versus $E^{\circ\prime}$((Fc⁺/Fc) S) measured at four different scan rates (20, 50, 100 & 200 mV s^{-1}). Right: Determination of the limits from the ECW for DCE from the CV measurement at v = 100 mV s^{-1}. The black arrows indicate, in both illustrations, the start and the direction of each cycle shown.

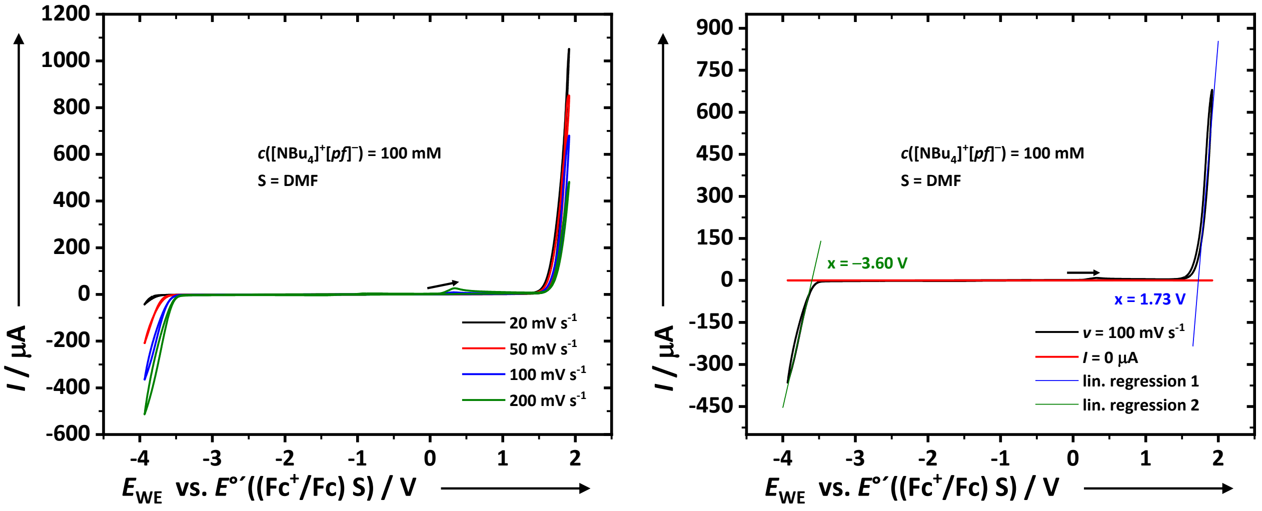

Supplementary Figure 28: Left: 2nd cycle of S = neat DMF with dissolved $[NBu_4]^+[pf]^-$ (100 mM) versus $E°'((Fc^+/Fc)\ S)$ measured at four different scan rates (20, 50, 100 & 200 mV s^{-1}). Right: Determination of the limits from the ECW for DMF from the CV measurement at v = 100 mV s^{-1}. The black arrows indicate, in both illustrations, the start and the direction of each cycle shown.

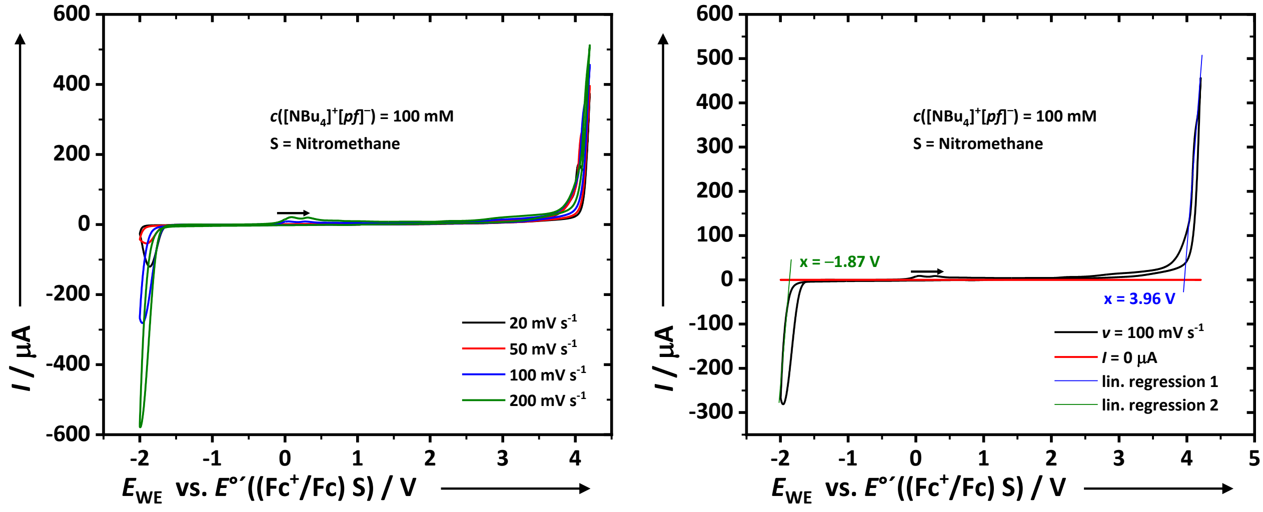

Supplementary Figure 29: Left: 2nd cycle of S = neat nitromethane with dissolved $[NBu_4]^+[pf]^-$ (100 mM) versus $E°'((Fc^+/Fc)\ S)$ measured at four different scan rates (20, 50, 100 & 200 mV s^{-1}). Right: Determination of the limits from the ECW for nitromethane from the CV measurement at v = 100 mV s^{-1}. The black arrows indicate, in both illustrations, the start and the direction of each cycle shown.

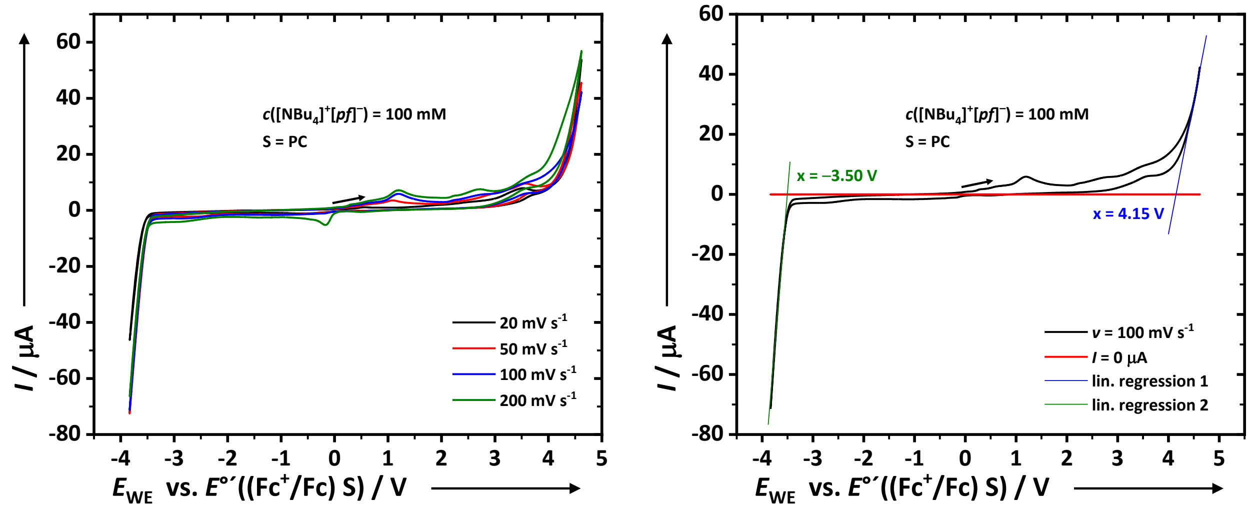

Supplementary Figure 30: Left: 2nd cycle of S = neat PC with dissolved $[NBu_4]^+[pf]^-$ (100 mM) versus $E°´$((Fc+/Fc) S) measured at four different scan rates (20, 50, 100 & 200 mV s^{-1}). Right: Determination of the limits from the ECW for PC from the CV measurement at v = 100 mV s^{-1}. The black arrows indicate, in both illustrations, the start and the direction of each cycle shown.

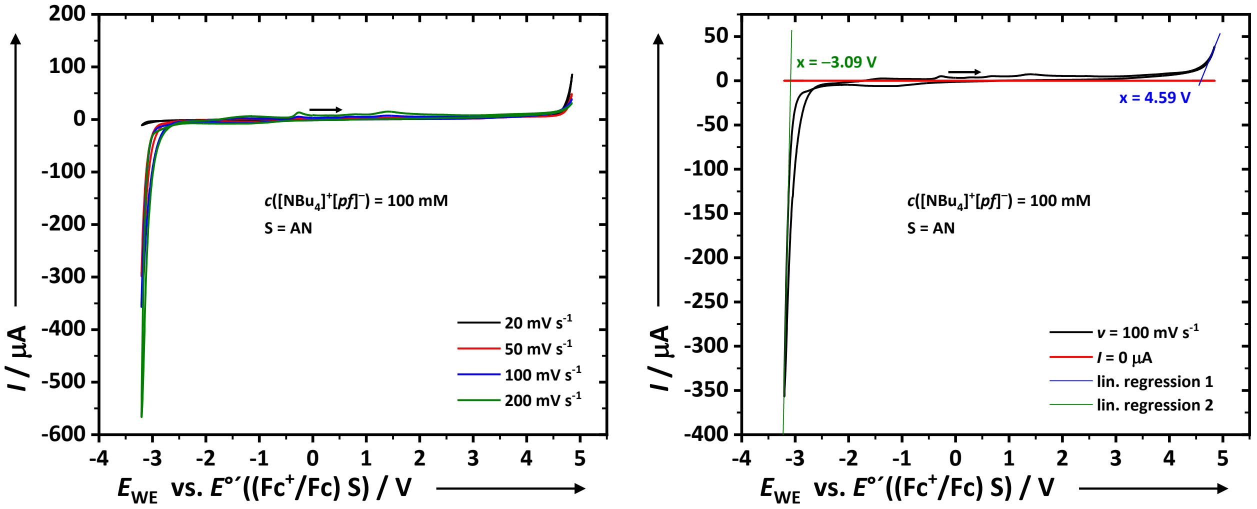

Supplementary Figure 31: Left: 2nd cycle of S = neat AN with dissolved $[NBu_4]^+[pf]^-$ (100 mM) versus $E°´$((Fc+/Fc) S) measured at four different scan rates (20, 50, 100 & 200 mV s^{-1}). Right: Determination of the limits from the ECW for AN from the CV measurement at v = 100 mV s^{-1}. The black arrows indicate, in both illustrations, the start and the direction of each cycle shown.

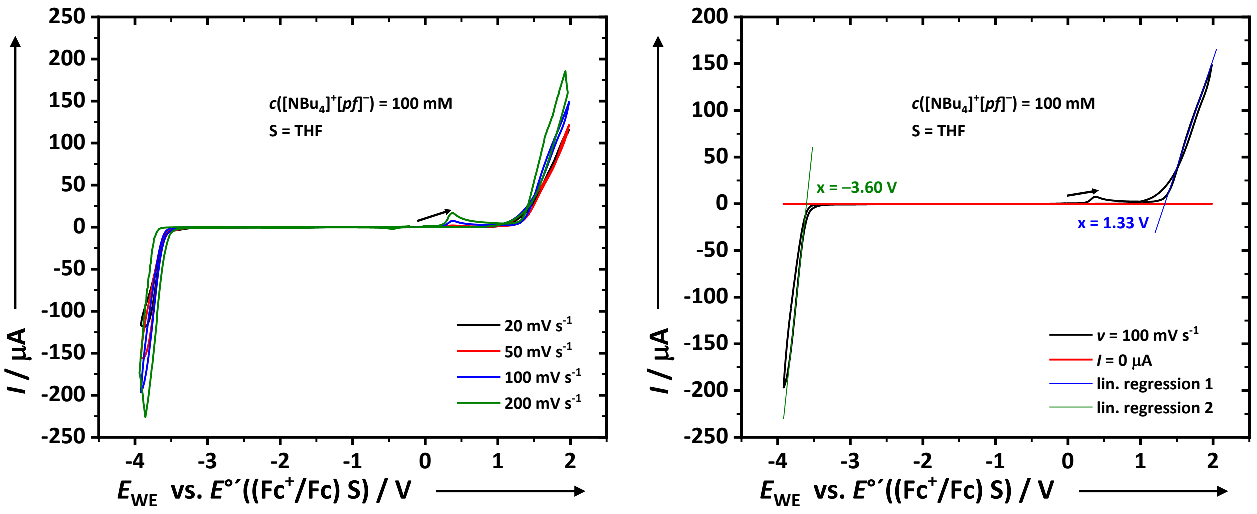

Supplementary Figure 32: Left: 2nd cycle of S = neat THF with dissolved $[NBu_4]^+[pf]^-$ (100 mM) versus $E°'$((Fc⁺/Fc) S) measured at four different scan rates (20, 50, 100 & 200 mV s^{-1}). Right: Determination of the limits from the ECW for THF from the CV measurement at v = 100 mV s^{-1}. The black arrows indicate, in both illustrations, the start and the direction of each cycle shown.

3.4 Measurements of Redox Systems in Fluorinated Arenes Using the Fc-Compartment Reference

CV measurements were performed in 1FB up to 5FB, in further selected fluoroarenes, 6FB and 1,3-difluorobenzene, CV measurements were not feasible due to conductivity and/or solubility problems. The used solvents were selected due to the usage within our group.

3.4.1 NO^+(S)/NO

This section contains measurements of the redox systems NO^+(S)/NO, ($[TBPA]^{\cdot+}$/TBPA) S) Perfluoroanthracene (Anthracene^F) in 4FB, and Perfluorophenanthrene (Phenanthrene^F) in 4FB. The used reference electrode was the Fc-Compartement Reference as described in section 3.1. All values reported here are corrected with the values given in Section 3.2.2.

Supplementary Table 7: Measured half-wave potentials of NO^+ versus $E°´((Fc^+/Fc)\ S)$ in selected fluorinated arenes S at different scan rates (*v*).

Solvent S	*v* / mV s⁻¹	$E_{1/2}(NO^+(S)/NO)$ vs. $E°´((Fc^+/Fc)\ S)$ / V
1FB	20	1.11
	50	1.11
	100	1.11
	200	1.11
2FB	20	1.24
	50	1.23
	100	1.23
	200	1.23
3FB	20	1.42
	50	1.42
	100	1.42
	200	1.42
4FB	20	1.52
	50	1.52
	100	1.52
	200	1.52
5FB	20	1.47
	50	1.47
	100	1.47
	200	1.48

The conclusion from Supplementary Table 7 is that the half-wave potential from NO^+ in these solvents are almost independent from the scan rate *v*. These differences can be explained due to rounding to two decimal spaces.

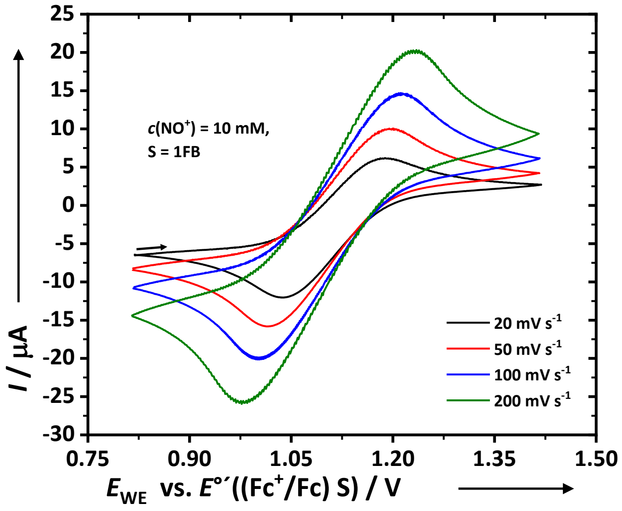

Supplementary Figure 33: 2nd cycles of NO^+ (10 mM) in S = 1FB versus $E°´((Fc^+/Fc)\ S)$ measured at four different scan rates (20, 50, 100 & 200 mV s^{-1}). The black arrow indicates for all scan rates the start and the direction of the 2nd cycle.

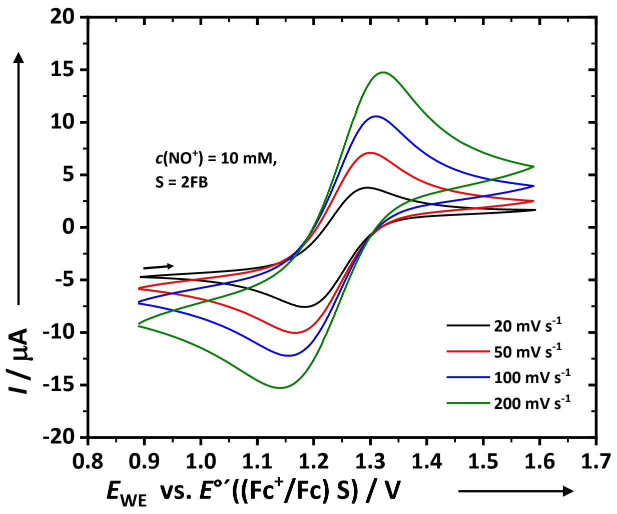

Supplementary Figure 34: 2nd cycles of NO^+ (10 mM) in S = 2FB versus *E*°´((Fc⁺/Fc) S) measured at four different scan rates (20, 50, 100 & 200 mV s^{-1}). The black arrow indicates for all scan rates the start and the direction of the 2nd cycle.

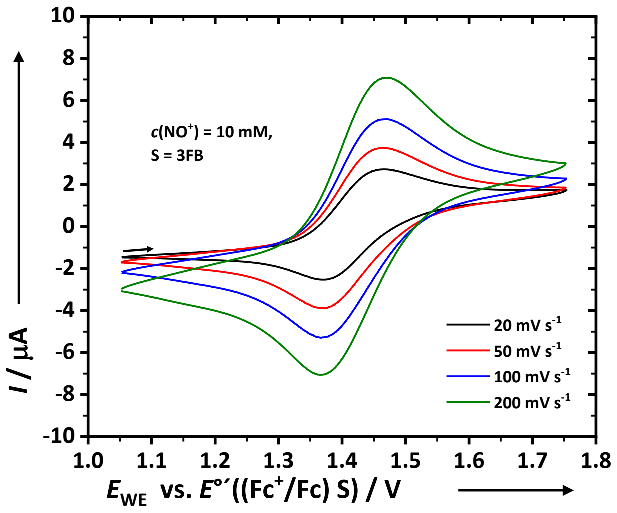

Supplementary Figure 35: 2nd cycles of NO^+ (10 mM) in S = 3FB versus *E*°´((Fc⁺/Fc) S) measured at four different scan rates (20, 50, 100 & 200 mV s^{-1}). The black arrow indicates for all scan rates the start and the direction of the 2nd cycle.

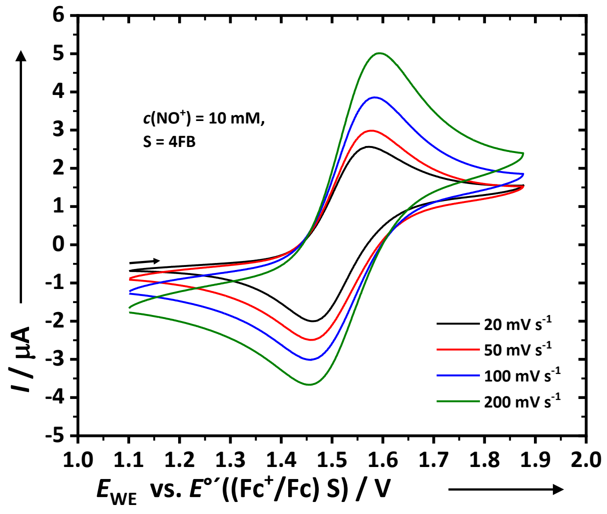

Supplementary Figure 36: 2nd cycles of NO^+ (10 mM) in S = 4FB versus $E°'$((Fc^+/Fc) S) measured at four different scan rates (20, 50, 100 & 200 mV s^{-1}). The black arrow indicates for all scan rates the start and the direction of the 2nd cycle.

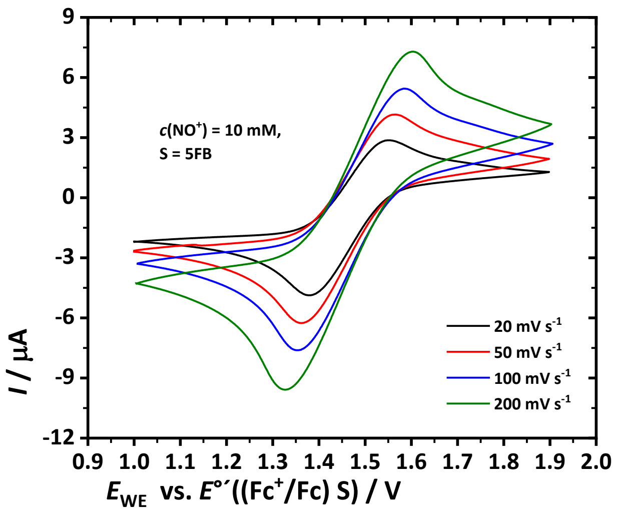

Supplementary Figure 37: 2nd cycles of NO^+ (10 mM) in S = 5FB versus $E°'$((Fc^+/Fc) S) measured at four different scan rates (20, 50, 100 & 200 mV s^{-1}). The black arrow indicates for all scan rates the start and the direction of the 2nd cycle.

3.4.1.1 $I_p \propto v^{1/2}$ Plot

Supplementary Table 8: Anodic peak current obtained by cyclic voltammetry of NO^+ in in selected fluorinated arenes and other solvents at different scan rates *v* given together with $v^{1/2}$ and R^2 from the linear regression.

Solvent	$v^{1/2}$ / (mV $s^{-1})^{1/2}$	I_{pa} / µA	R^2
1FB	4.47	6.20	0.99887
	7.07	10.05	
	10.00	14.70	
	14.14	20.32	
2FB	4.47	3.78	0.99686
	7.07	7.09	
	10.00	10.57	
	14.14	14.75	
3FB	4.47	2.72	0.99860
	7.07	3.75	
	10.00	5.12	
	14.14	7.08	
4FB	4.47	2.56	0.98979
	7.07	2.99	
	10.00	3.86	
	14.14	5.01	
5FB	4.47	2.87	0.99942
	7.07	4.15	
	10.00	5.45	
	14.14	7.29	

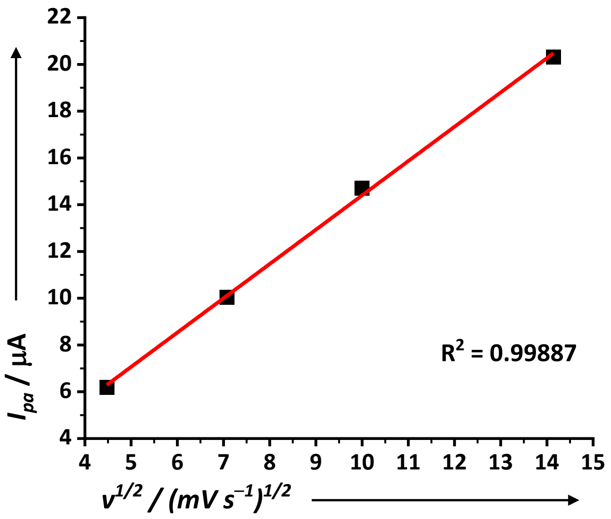

Supplementary Figure 38: Plot of the anodic peak current I_{pa} versus the sqaure root of the scan rate $v^{1/2}$ from the cyclic voltammograms of NO^+ vs. $E°'((Fc^+/Fc)\ S)$ in S = 1FB, together with the linear regression and corresponding R^2 value for the linear regression.

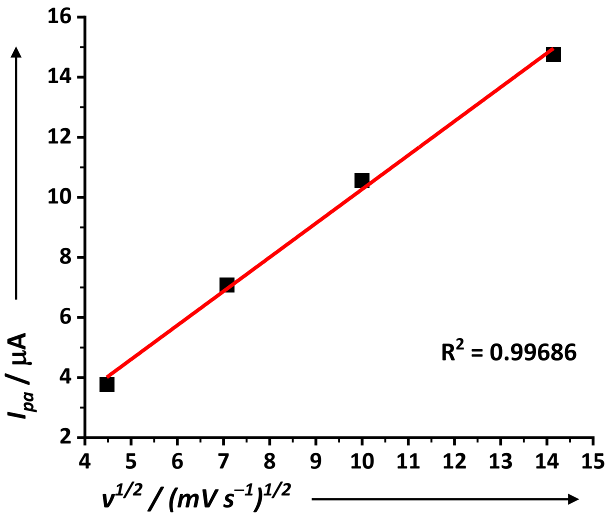

Supplementary Figure 39: Plot of the anodic peak current I_{pa} versus the square root of the scan rate $v^{1/2}$ from the cyclic voltammograms of NO^+ vs. $E°'$((Fc$^+$/Fc) S) in S = 2FB, together with the linear regression and corresponding R^2 value for the linear regression.

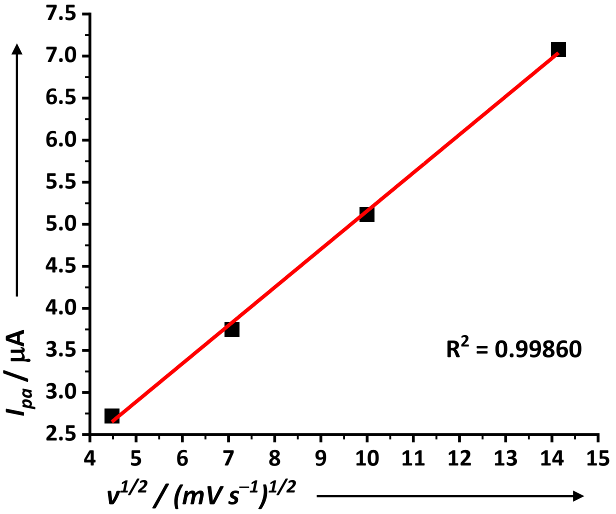

Supplementary Figure 40: Plot of the anodic peak current I_{pa} versus the square root of the scan rate $v^{1/2}$ from the cyclic voltammograms of NO^+ vs. $E°'$((Fc$^+$/Fc) S) in S = 3FB, together with the linear regression and corresponding R^2 value for the linear regression.

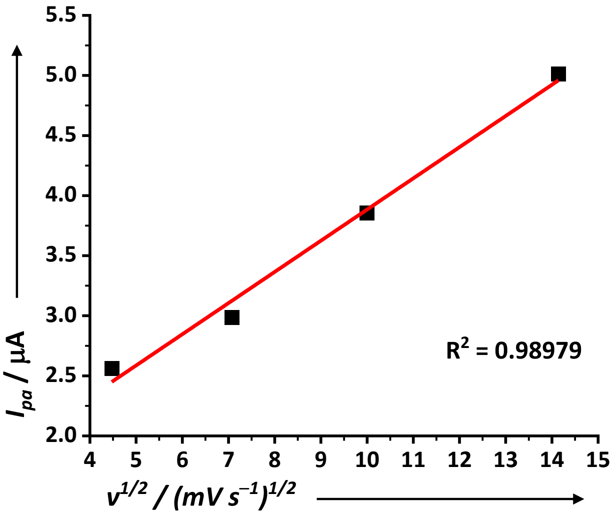

Supplementary Figure 41: Plot of the anodic peak current I_{pa} versus the square root of the scan rate $v^{1/2}$ from the cyclic voltammograms of NO$^+$ vs. $E^{\circ\prime}$((Fc$^+$/Fc) S) in S = 4FB, together with the linear regression and corresponding R^2 value for the linear regression.

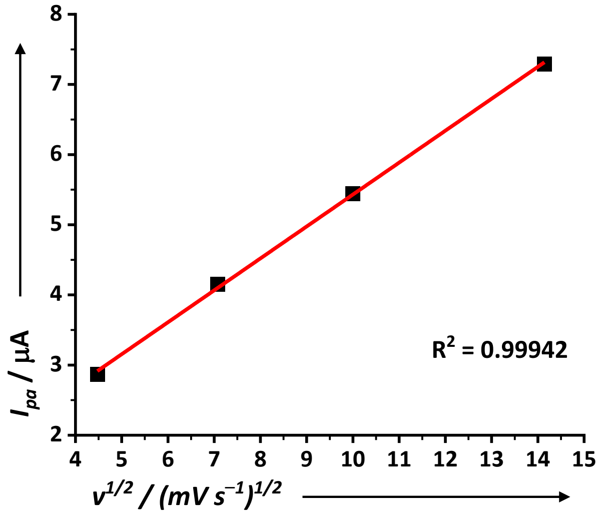

Supplementary Figure 42: Plot of the anodic peak current I_{pa} versus the square root of the scan rate $v^{1/2}$ from the cyclic voltammograms of NO$^+$ vs. $E^{\circ\prime}$((Fc$^+$/Fc) S) in S = 5FB, together with the linear regression and corresponding R^2 value for the linear regression.

3.4.2 ([TBPA]$^{\cdot+}$/TBPA) S

The Tris(4-bromophenyl)amine (TBPA) represents the neutral form of the cation from the well-known compound TBPA[$SbCl_6$], colloquially called Magic Blue, because of its intense dark blue colour. Magic blue serves as example for a huge group of one-electron deelectronators, with large applications all over the organic chemistry.[67] The TBPA-values measured in this work agree with the literature obtained by measurements with the [TBPA]$^{\cdot+}$/TBPA for DCM and AN. This indicates the expected small solvent dependence of the potential of the TBPA cation.

$E^{\circ\prime}$(([TBPA]$^{\cdot+}$/TBPA) S) in the used fluorinated arenes S are summarized in Supplementary Table 9 and visualised in Supplementary Figure 43–Supplementary Figure 47. As shown in Supplementary Table 9, the potentials are rarely influenced by the scan rate. Small differences can probably be attributed to

measurement inaccuracies and rounding errors.

Supplementary Table 9: Measured $E°'$(([TBPA]$^{\cdot+}$/TBPA) S) versus $E°'$((Fc^+/Fc) S) in selected fluorinated arenes S and other solvents at different scan rates (v).

Solvent S	v / mV s^{-1}	$E°'$(([TBPA]$^{\cdot+}$/TBPA) S) vs. $E°'$((Fc^+/Fc) S) / V
1FB	20	0.70
	50	0.70
	100	0.69
	200	0.68
2FB	20	0.70
	50	0.70
	100	0.69
	200	0.70
3FB	20	0.69
	50	0.69
	100	0.69
	200	0.69
4FB	20	0.69
	50	0.69
	100	0.69
	200	0.70
5FB	20	0.68
	50	0.68
	100	0.69
	200	0.69

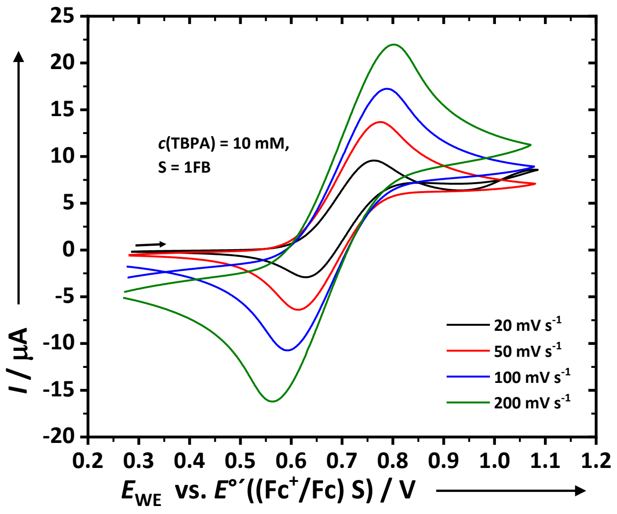

Supplementary Figure 43: 2nd cycles of TBPA (10 mM) in S = 1FB versus $E°'$((Fc^+/Fc) S) measured at four different scan rates (20, 50, 100 & 200 mV s^{-1}). The black arrow indicates for all scan rates the start and the direction of the 2nd cycle.

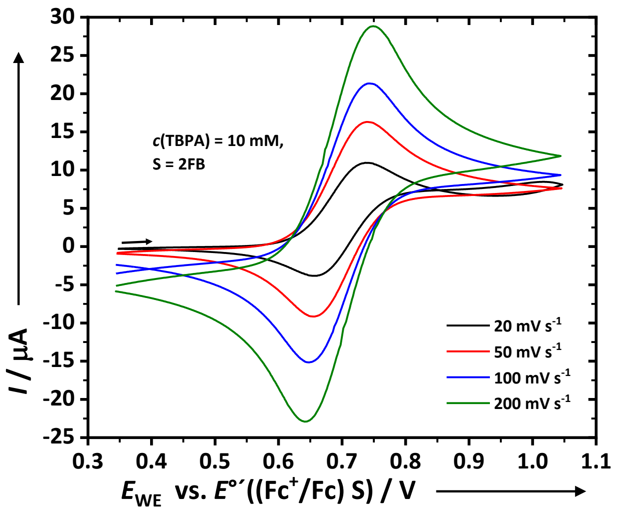

Supplementary Figure 44: 2nd cycles of TBPA (10 mM) in S = 2FB versus $E°´$((Fc$^+$/Fc) S) measured at four different scan rates (20, 50, 100 & 200 mV s^{-1}). The black arrow indicates for all scan rates the start and the direction of the 2nd cycle.

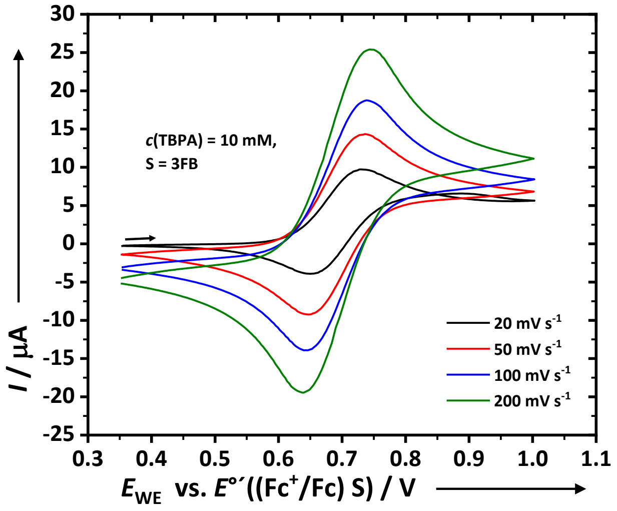

Supplementary Figure 45: 2nd cycles of TBPA (10 mM) in S = 3FB versus $E°´$((Fc$^+$/Fc) S) measured at four different scan rates (20, 50, 100 & 200 mV s^{-1}). The black arrow indicates for all scan rates the start and the direction of the 2nd cycle.

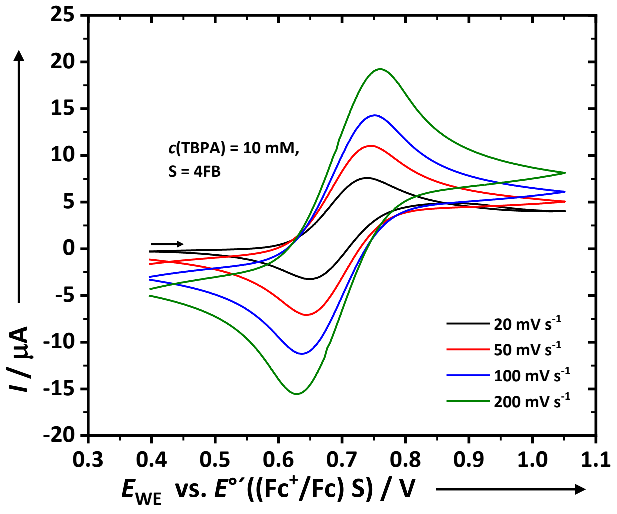

Supplementary Figure 46: 2nd cycles of TBPA (10 mM) in S = 4FB versus $E^{\circ\prime}$((Fc$^+$/Fc) S) measured at four different scan rates (20, 50, 100 & 200 mV s^{-1}). The black arrow indicates for all scan rates the start and the direction of the 2nd cycle.

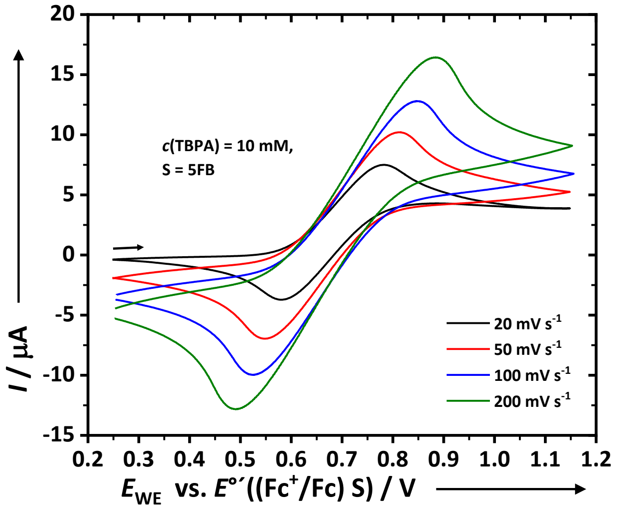

Supplementary Figure 47: 2nd cycles of TBPA (10 mM) in S = 5FB versus $E^{\circ\prime}$((Fc$^+$/Fc) S) measured at four different scan rates (20, 50, 100 & 200 mV s^{-1}). The black arrow indicates for all scan rates the start and the direction of the 2nd cycle.

3.4.3 ([anthracene^F]$^+$/anthracene^F) 4FB: Perfluoroanthracene (Anthracene^F)

The redox system [anthracene^F]$^+$/anthracene^F was investigated in 4FB only. The data are summarized with Supplementary Table 10, and Supplementary Figure 48–Supplementary Figure 50.

Supplementary Table 10: Measured $E°´$(([anthracene^F]$^{·+}$/anthracene^F) S) versus $E°´$((Fc$^+$/Fc) S) in 4FB at different scan rates (v) using [NBu$_4$]$^+$[Al(OR^F)$_4$]$^-$ (100 mM) as supporting electrolyte.

v / mV s^{-1}	$E°´$ vs. $E°´$((Fc$^+$/Fc) 4FB) / V
20	1.471
50	1.474
100	1.469
200	1.469
500	1.474
1000	1.474

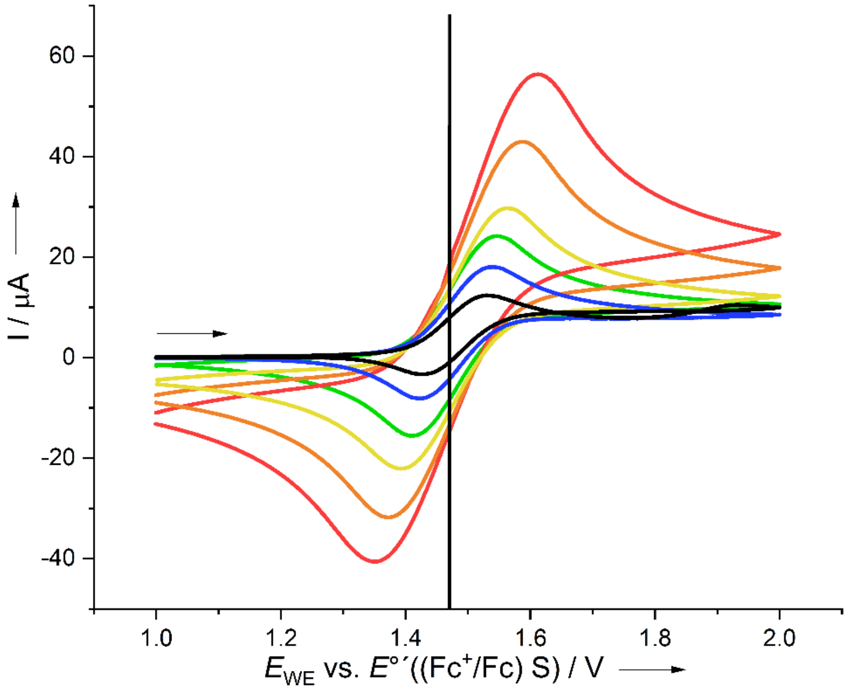

Supplementary Figure 48: Cyclic voltammograms (2nd cycle) at different scan rates (black (20 mV s^{-1}) → green (100 mV s^{-1}) → red (1000 mV s^{-1})) of anthracene^F (10 mM) in 1,2,3,4-tetrafluorobenzene using [NBu$_4$]$^+$[Al(OR^F)$_4$]$^-$ (100 mM) supporting electrolyte.

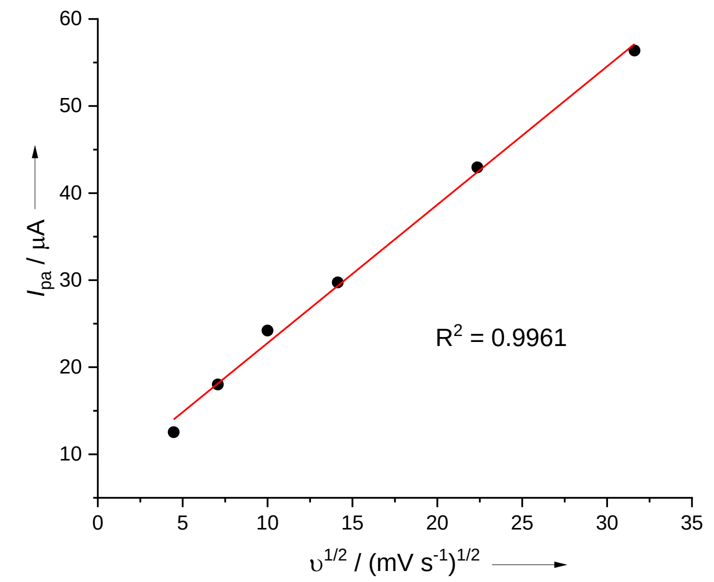

Supplementary Figure 49: Linear fit of the anodic peak current I_{pa} against the square root of the scan rate $v^{1/2}$ of anthracene^F.

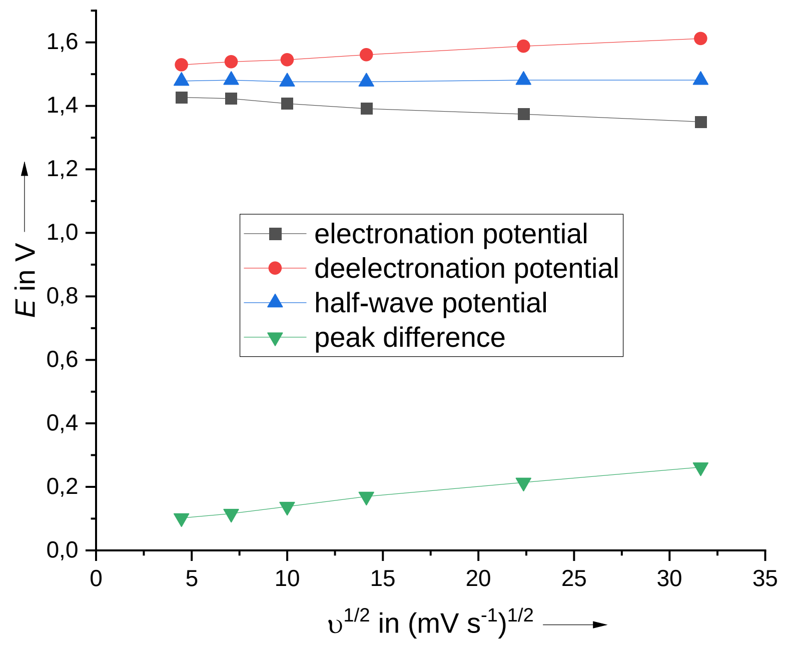

Supplementary Figure 50: Electronation-, deelectronation- and half-wave potential & peak difference against the square root of the scan rate $v^{1/2}$ of anthracene^F

3.4.4 ([phenanthrene^F]$^+$/phenanthene^F) 4FB: Perfluorophenanthrene (Phenanthrene^F)

The redox system [phenanthrene^F]$^+$/phenanthene^F was investigated in 4FB only. The data are summarized with Supplementary Table 11, and Supplementary Figure 51 up to Supplementary Figure 53.

Supplementary Table 11: Measured $E°'$(([phenanthrene^F]$^{\cdot+}$/phenanthrene^F) S) versus $E°'$((Fc^+/Fc) S) in 4FB at different scan rates (v) using $[NBu_4]^+[Al(OR^F)_4]^-$ (100 mM) as supporting electrolyte.

v / mV s^{-1}	$E°'$ vs. $E°'$((Fc^+/Fc) 4FB) / V
20	1.896
50	1.894
100	1.894
200	1.898
500	1.904
1000	1.911

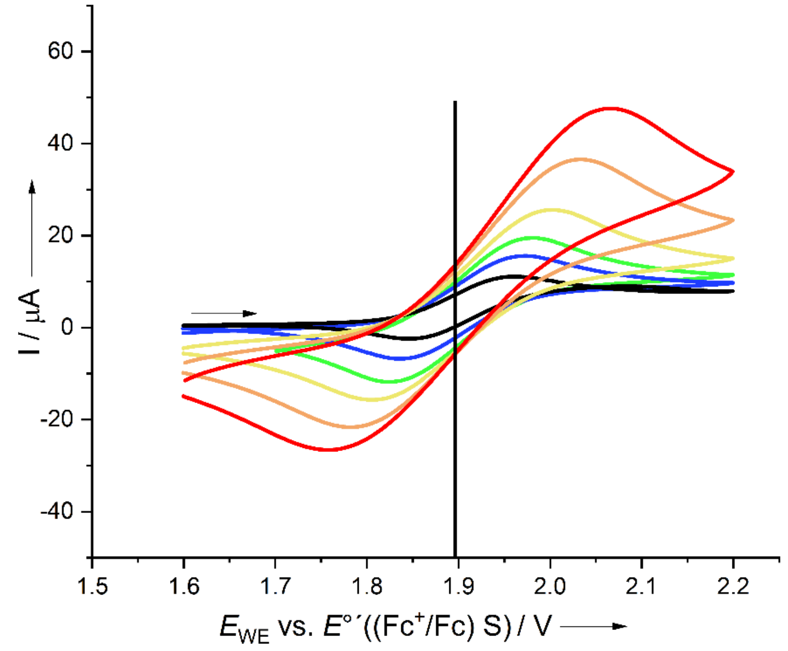

Supplementary Figure 51: Cyclic voltammograms (2nd cycle) at different scan rates (black (20 mV s^{-1}), blue (50 mV s^{-1}), green (100 mV s^{-1}), yellow (200 mV s^{-1}), orange (500 mV s^{-1}), red (1000 mV s^{-1})) of phenanthrene^F (10 mM) in 1,2,3,4-tetrafluorobenzene using $[NBu_4]^+[Al(OR^F)_4]^-$ (100 mM) as conducting salt.

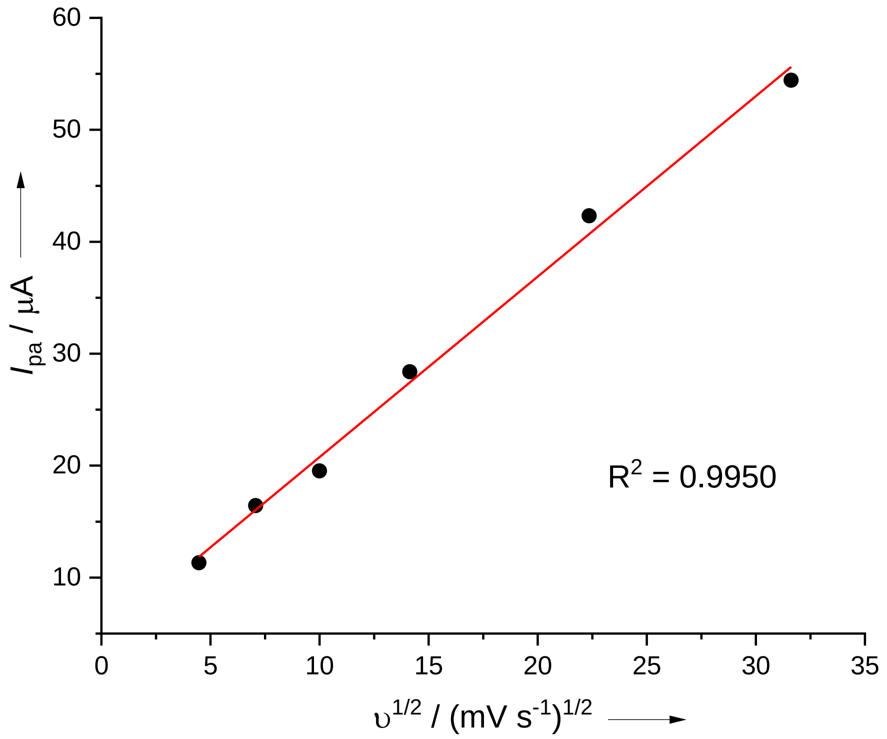

Supplementary Figure 52: Linear fit of the anodic peak current I_{pa} against the square root of the scan rate $v^{1/2}$ of phenanthrene[F].

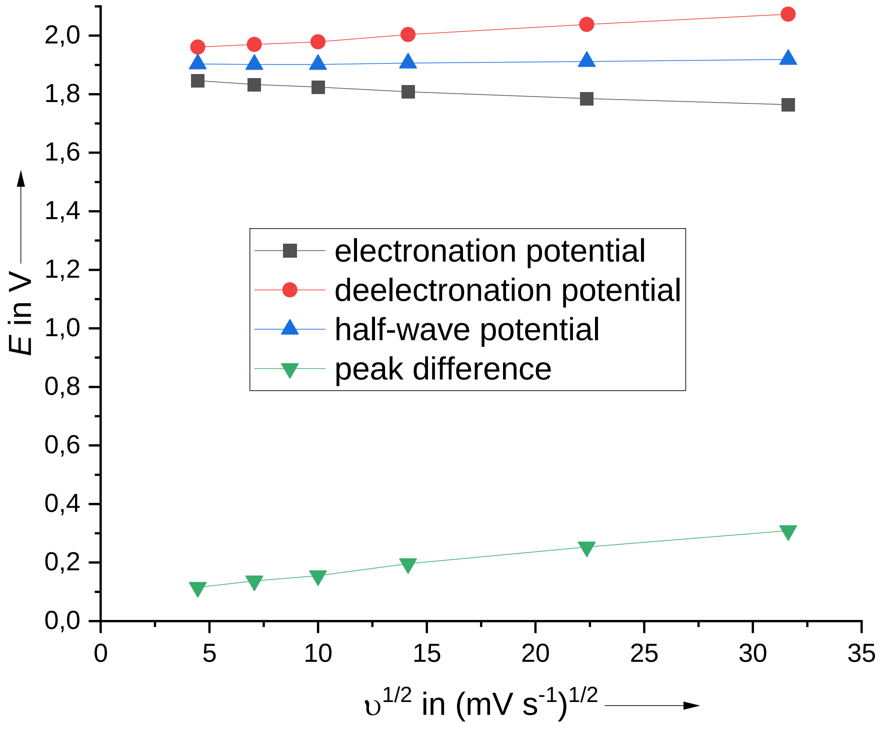

Supplementary Figure 53: Electronation-, deelectronation- and half-wave potential & peak difference against the square root of the scan rate $v^{1/2}$ of phenanthrene[F].

3.5 Measurements of Redox Systems in Fluorinated Arenes Using the Ag-compartment Reference

This section contains measurements of the redox systems (Fc^+/Fc) S and NO^+(S)/NO. The used reference electrode was the Ag-compartment reference as described in Section 3.1. Thus, the formal potential of the Ag^+/Ag redox system can be given with respect to the formal potential of the Fc^+/Fc redox system, $E°'(Ag^+(S)/Ag) = -(E_m + RT/F \cdot \ln c(Ag^+, S)) = -(E_m - 0{,}118\ V)$, with E_m is the measured potential $E_{1/2}((Fc^+/Fc)\ S)$ vs. $E(Ag^+(10mM, S)/Ag)$ or $E_{1/2}(NO^+(S)/NO)$ vs. $E(Ag^+(10mM, S)/Ag)$.

3.5.1 (Fc^+/Fc) S

The experimental values of $E_{1/2}((Fc^+/Fc)\ S)$ and the corresponding calculated value $E°'(Ag^+(S)/Ag)$ are summarized in Supplementary Table 12, reported together with solvent (S) and scan rate *v*. Additionally, the experimental results are illustrated in Supplementary Figure 54-Supplementary Figure 58 for each solvent.

Supplementary Table 12: Measured half-wave potentials $E_{1/2}(Fc^+/Fc, S)$ versus $E(Ag^+(10\ mM)/Ag, S)$ and the calculated potentials $E°'(Ag^+/Ag, S)$ vs. $E°'(Fc^+/Fc, S)$ in selected fluorinated arenes and other solvents at different scan rates (*v*).

Solvent	v / mV s^{-1}	$E_{1/2}((Fc^+/Fc)\ S)$ vs. $E(Ag^+(10\ mM, S)/Ag)$ / V	$E°'(Ag^+(S)/Ag)$ vs. $E°'((Fc^+/Fc)\ S)$ / V
1FB	20	−0.63	0.74
	50	−0.63	0.74
	100	−0.63	0.74
	200	−0.62	0.74
2FB	20	−0.87	0.99
	50	−0.87	0.99
	100	−0.87	0.99
	200	−0.87	0.99
3FB	20	−1.14	1.26
	50	−1.15	1.26
	100	−1.14	1.26
	200	−1.14	1.26
4FB	20	−1.35	1.47
	50	−1.35	1.47
	100	−1.35	1.47
	200	−1.35	1.47
5FB	20	−1.38	1.50
	50	−1.38	1.50
	100	−1.38	1.50
	200	−1.39	1.50

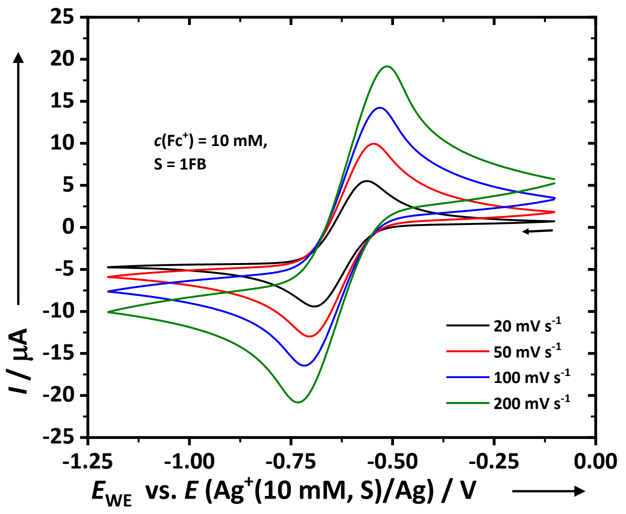

Supplementary Figure 54: 2nd cycles of Fc^+ (10 mM) in S = 1FB versus *E*(Ag^+(10 mM, S)/Ag) measured at four different scan rates (20, 50, 100 & 200 mV s^{-1}). The black arrow indicates for all scan rates the start and the direction of the 2nd cycle.

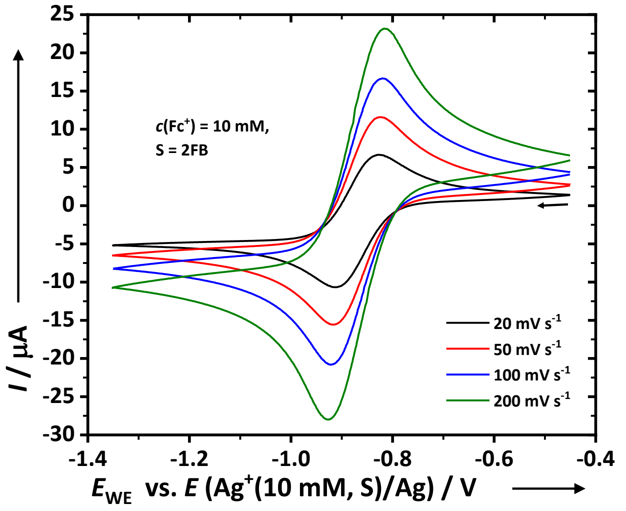

Supplementary Figure 55: 2nd cycles of Fc^+ (10 mM) in S = 2FB versus *E*(Ag^+(10 mM, S)/Ag) measured at four different scan rates (20, 50, 100 & 200 mV s^{-1}). The black arrow indicates for all scan rates the start and the direction of the 2nd cycle.

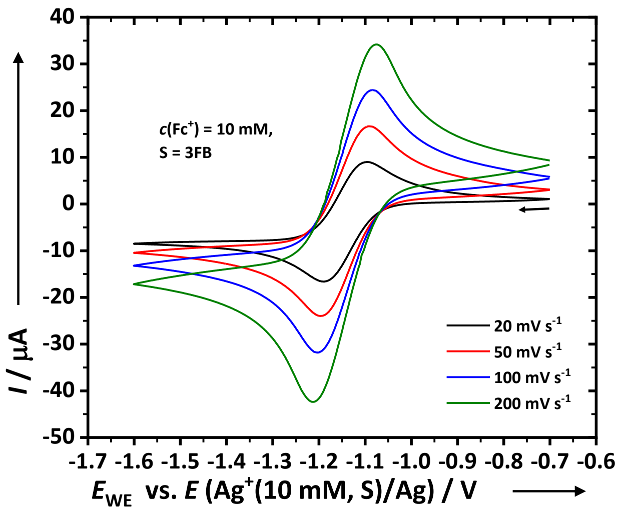

Supplementary Figure 56: 2nd cycles of Fc^+ (10 mM) in S = 3FB versus *E*(Ag^+(10 mM, S)/Ag) measured at four different scan rates (20, 50, 100 & 200 mV s^{-1}). The black arrow indicates for all scan rates the start and the direction of the 2nd cycle.

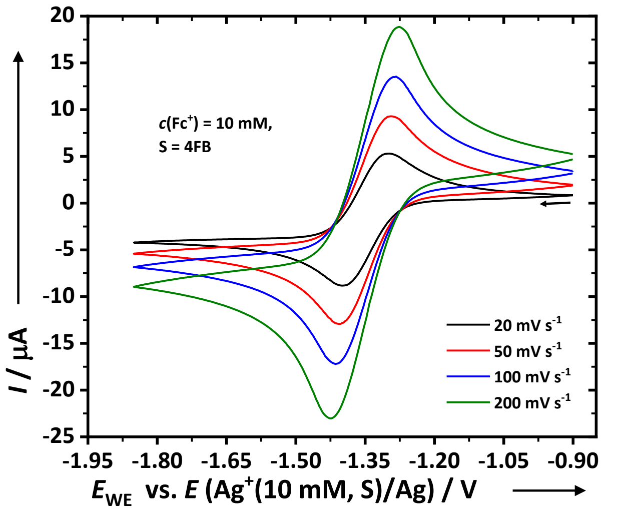

Supplementary Figure 57: 2nd cycles of Fc^+ (10 mM) in S = 4FB versus *E*(Ag^+(10 mM, S)/Ag) measured at four different scan rates (20, 50, 100 & 200 mV s^{-1}). The black arrow indicates for all scan rates the start and the direction of the 2nd cycle.

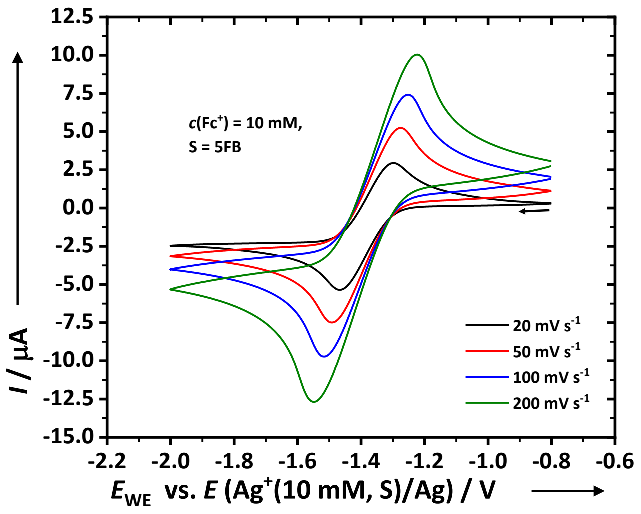

Supplementary Figure 58: 2nd cycles of Fc^+ (10 mM) in S = 5FB versus $E(Ag^+$(10 mM, S)/Ag) measured at four different scan rates (20, 50, 100 & 200 mV s^{-1}). The black arrow indicates for all scan rates the start and the direction of the 2nd cycle.

3.5.2 NO^+(S)/NO

Additionally, $E_{1/2}(NO^+$(S)/NO) was measured with the Ag-compartment reference and recalculated to $E°´(Ag^+$(S)/Ag) as above. The results of these experiments are summarized in Supplementary Table 13 and visualised in Supplementary Figure 59–-Supplementary Figure 63.

Supplementary Table 13: Measured half-wave potentials of NO^+ versus $E(Ag^+$(10 mM, S)/Ag) and calculated values for $E_{1/2}(NO^+$(S)/NO) versus $E°´(Ag^+$(S)/Ag) / V in selected fluorinated arenes at different scan rates (v).

Solvent S	v / mV s^{-1}	$E_{1/2}(NO^+$(S)/NO) vs. $E(Ag^+$(10 mM, S)/Ag) / V	$E_{1/2}(NO^+$(S)/NO) vs. $E°´(Ag^+$(S)/Ag) / V
1FB	20	0.51	0.39
	50	0.51	0.39
	100	0.51	0.39
	200	0.51	0.39
2FB	20	0.37	0.25
	50	0.37	0.25
	100	0.37	0.25
	200	0.37	0.25
3FB	20	0.23	0.12
	50	0.23	0.11
	100	0.23	0.11
	200	0.23	0.11
4FB	20	0.13	0.01
	50	0.13	0.01
	100	0.13	0.01
	200	0.13	0.01
5FB	20	0.12	0.00
	50	0.12	0.00
	100	0.11	–0.01
	200	0.11	–0.01

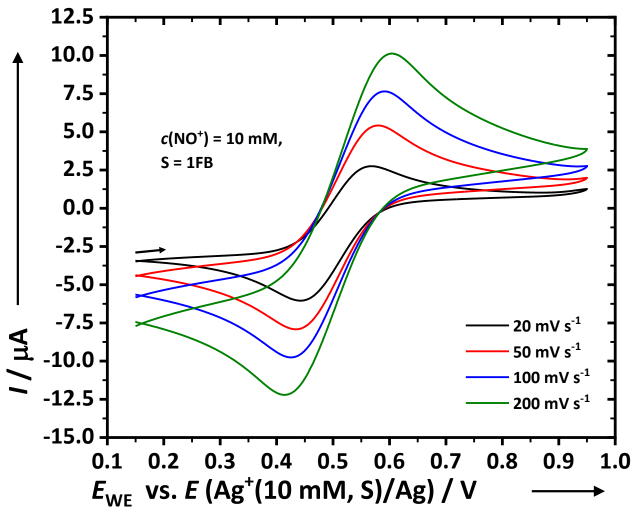

Supplementary Figure 59: 2nd cycles of NO^+ (10 mM) in S = 1FB versus *E*(Ag^+(10 mM, S)/Ag) measured at four different scan rates (20, 50, 100 & 200 mV s^{-1}). The black arrow indicates for all scan rates the start and the direction of the 2nd cycle.

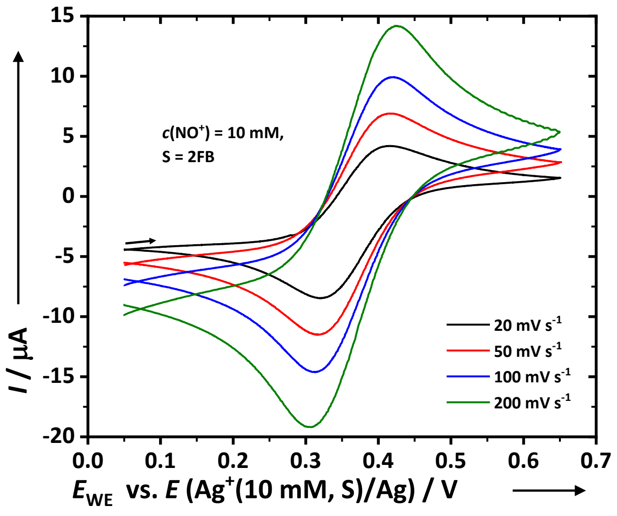

Supplementary Figure 60: 2nd cycles of NO^+ (10 mM) in S = 2FB versus *E*(Ag^+(10 mM, S)/Ag) measured at four different scan rates (20, 50, 100 & 200 mV s^{-1}). The black arrow indicates for all scan rates the start and the direction of the 2nd cycle.

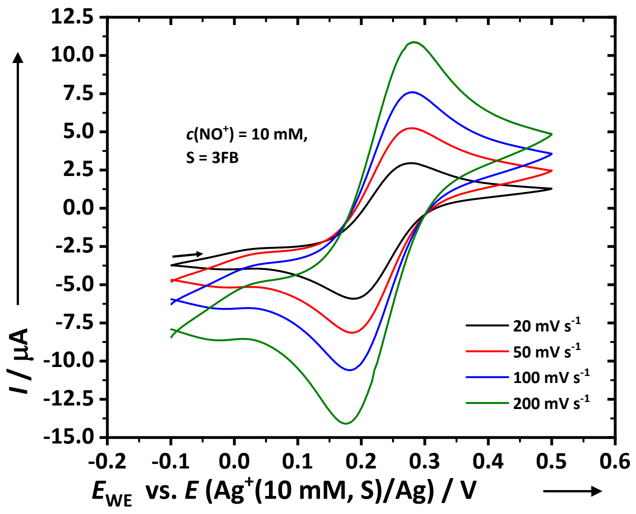

Supplementary Figure 61: 2nd cycles of NO^+ (10 mM) in S = 3FB versus *E*(Ag^+(10 mM, S)/Ag) measured at four different scan rates (20, 50, 100 & 200 mV s^{-1}). The black arrow indicates for all scan rates the start and the direction of the 2nd cycle.

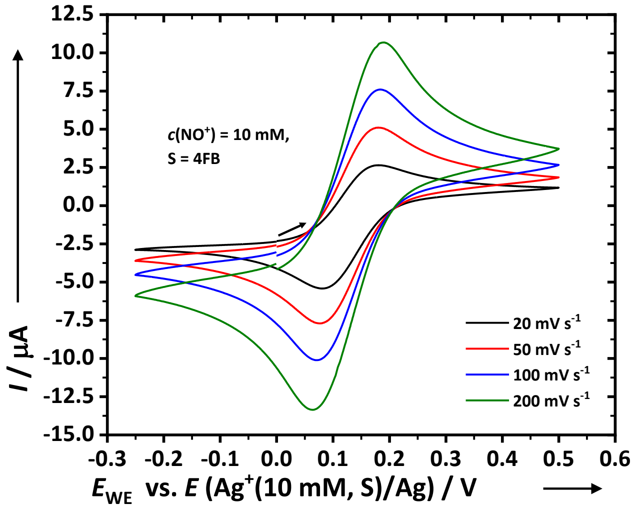

Supplementary Figure 62: 2nd cycles of NO^+ (10 mM) in S = 4FB versus *E*(Ag^+(10 mM, S)/Ag) measured at four different scan rates (20, 50, 100 & 200 mV s^{-1}). The black arrow indicates for all scan rates the start and the direction of the 2nd cycle.

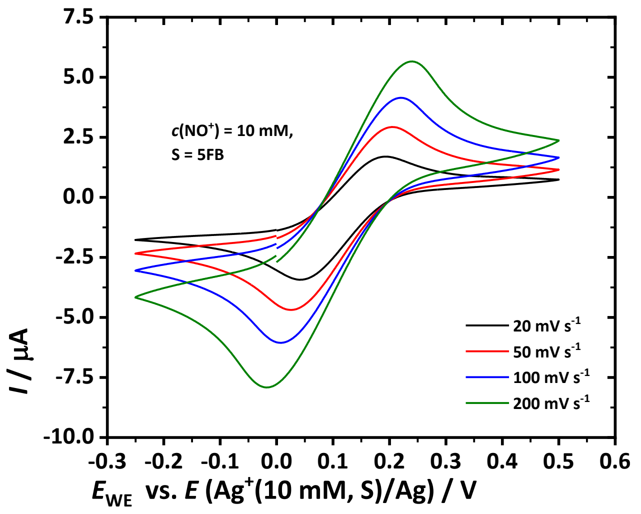

Supplementary Figure 63: 2nd cycles of NO^+ (10 mM) in S = 5FB versus $E(Ag^+(10\text{ mM}, S)/Ag)$ measured at four different scan rates (20, 50, 100 & 200 mV s^{-1}). The black arrow indicates for all scan rates the start and the direction of the 2nd cycle.

3.5.3 Triangular Born-Fajans-Haber-Cycles of the Redox Systems NO^+/NO – Ag^+/Ag – Fc^+/Fc

The results presented in Sections 3.4.1 (i.e. $E_{1/2}(NO^+(S)/NO)$ vs. $E°´((Fc^+/Fc)\ S)$, 3.5.1 (i.e. $E_{1/2}((Fc^+/Fc)\ S)$ vs. $E°´(Ag^+(S)/Ag)$, and 3.5.2 (i.e. $E_{1/2}(NO^+(S)/NO)$ vs. $E(Ag^+(S)/Ag)$ allow the presentation of a triangular Born-Fajans-Haber-Cycles to assess the consistency of our measurements. Perfect consistent data must show, for example, $\Delta E \coloneqq 0 = E°´(Ag^+(S)/Ag)$ vs. $E°´((Fc^+/Fc)\ S) + E_{1/2}(NO^+(S)/NO)$ vs. $E°´(Ag^+(S)/Ag) - E_{1/2}(NO^+(S)/NO)$ vs. $E°´((Fc^+/Fc)\ S)$, and the more the value deviates from zero the less consistent the data are.

Supplementary Table 14 and Supplementary Figure 64 show the results of this analysis. The consistency is fairly well depending on the solvent.

Supplementary Table 14: Measured $E_{1/2}(NO^+(S)/NO)$ recalculated value of $E°´(Ag^+(S)/Ag)$ versus $E°´((Fc^+/Fc)\ S)$ and the recalculated value of $E_{1/2}(NO^+(S)/NO)$ versus $E°´(Ag^+(S)/Ag)$ in selected fluorinated arenes at a scan rate of 100 mV s^{-1}, together with the value of the calculated potential difference (ΔE) and the value of the corresponding Gibbs Energy difference (ΔG). S represents the used solvent.

Solvent S	$E_{1/2}(NO^+(S)/NO)$ vs. $E°´((Fc^+/Fc)\ S)$ / V	$E°´(Ag^+(S)/Ag)$ vs. $E°´((Fc^+/Fc)\ S)$ / V	$E_{1/2}(NO^+(S)/NO)$ vs. $E°´(Ag^+(S)/Ag)$ / V	$\lvert\Delta E\rvert$ / V	$\lvert\Delta G\rvert^a$ / kJ mol^{-1}
1FB	1.11	0.74	0.39	0.03	2.51
2FB	1.23	0.99	0.25	0.00	0.34
3FB	1.42	1.26	0.11	0.04	3.72
4FB	1.52	1.47	0.01	0.05	4.68
5FB	1.47	1.50	–0.01	0.02	1.70

a) Values were calculated by $\Delta G = -nF\Delta E$, with n = number of electrons, ΔE = potential difference, F = Faraday constant = 96485 C mol^{-1} [68].

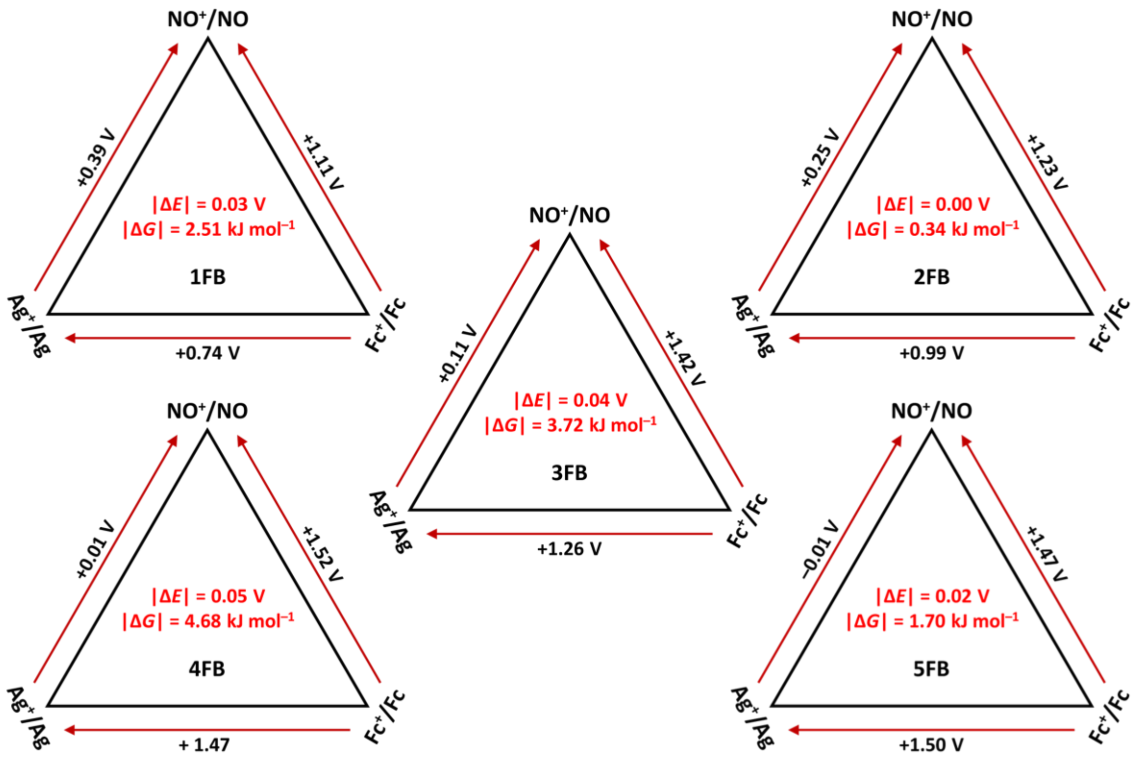

Supplementary Figure 64: Triangle diagrams of the three redox couples NO^+/NO, Ag^+/Ag und Fc^+/Fc in 1FB – 5FB. The mean errors of the calculated potential difference (ΔE) / the corresponding Gibbs Energy difference (ΔG) are given in red in the center of the triangle.

As shown in Supplementary Table 14, our measurements demonstrate overall only small deviations through the solvents, with no more than 4.35 kJ mol^{-1} towards the absolute value for the Gibbs energy error. In the light of these results, it was shown that our measurements for these solvents shows a great consistency.

3.5.4 Comparison of the redox potentials of NO^+(S)/NO and Ag^+(S)/Ag versus ((Fc^+/Fc) S) in the fluorinated arenes

Supplementary Table 15: Ionization energy, measured half-wave potentials of NO^+(S)/NO versus $E°´$((Fc^+/Fc) S) and calculated formal potential of Ag^+(S)/Ag in selected fluorinated arenes S at a scan rate of 100 mV s^{-1}.

Solvent S	IE / eV	$E_{1/2}$(NO^+(S)/NO) vs. $E°´$((Fc^+/Fc) S) / V	$E°´$(Ag^+(S)/Ag) vs. $E°´$((Fc^+/Fc) S) / V
1FB	9.20[68]	1.11	0.74
2FB	9.29[68]	1.23	0.99
3FB	9.40[69]	1.42	1.26
4FB	9.53[68]	1.52	1.47
5FB	9.63[68]	1.47	1.50

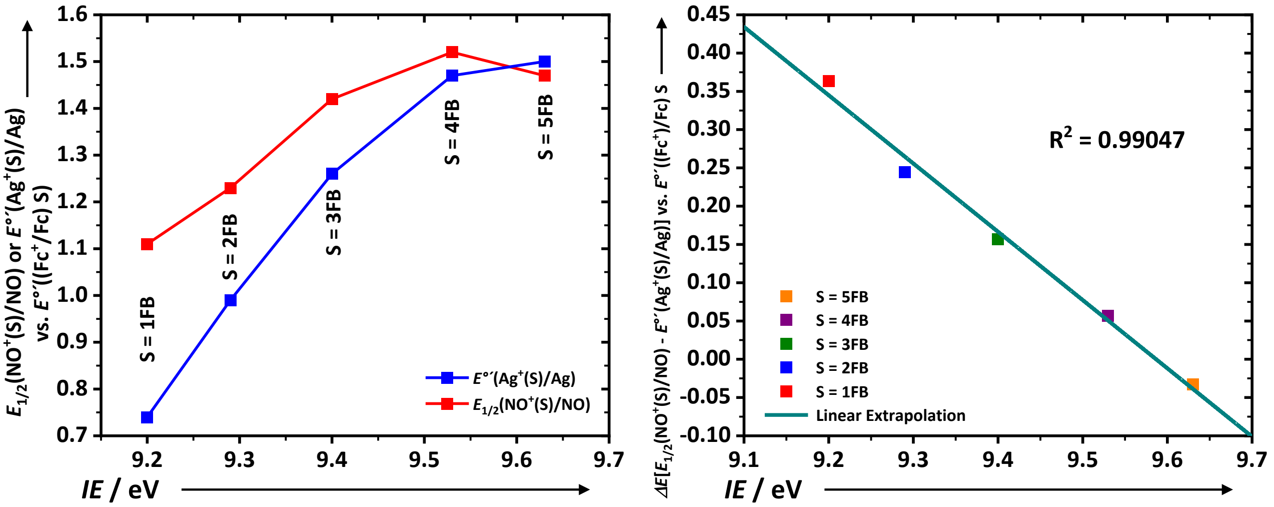

Supplementary Figure 65: Left: Comparison of $E_{1/2}$(NO^+(S)/NO) and $E°´$(Ag^+(S)/Ag) versus $E°´$((Fc^+/Fc) S) / V against the ionization energy of the used fluorinated arenes. Right: Differences between $E_{1/2}$(NO^+(S)/NO) and $E°´$(Ag^+(S)/Ag) versus $E°´$((Fc^+/Fc) S) / V against the ionization energy of the used fluorinated arenes. Linear regression of these values provides R^2 = 0.99047.

3.6 Measurements of Redox Systems in Selected Solvents Using the Fc-Compartment Reference

This section contains measurements of the redox systems $NO^+(S)/NO$ and $([N(4\text{-}BrC_6H_4)_3]^+/N(4\text{-}BrC_6H_4)_3)$ S. The selected solvents DCM, DCE, DMF, nitromethane, PC, AN and THF were chosen due to their relevance in a highly cited review article by Connelly and Geiger[67] and due to the use of these solvents in our group. The used reference electrode was the Fc-Compartement reference as described in section 3.1. All values reported here are corrected with the values given in Section 3.2.2.

3.6.1 $NO^+(S)/NO$

This section contains the summarized and illustrated results for the different CV measurements of the redox system NO^+/NO in the selected solvents. Supplementary Table 16 summarizes the results, Supplementary Figure 66 up to Supplementary Figure 71 show the corresponding CVs. The $E_{1/2}$ value of the redox system in dichloromethane differs significantly from the value reported by Lee *et al.*[70] Thus, this redox system was examined more closely in DCM. The concentration dependence of NO^+ is discussed in Section 3.6.1.2 and a comparison with data by Lee *et al.* is given in Section 3.6.1.3.

Supplementary Table 16: Measured half-wave potentials of $NO^+(S)/NO$ versus $E^{\circ\prime}((Fc^+/Fc)\ S)$ in selected solvents at different scan rates (v).

Solvent S	v / mV s^{-1}	$E_{1/2}(NO^+(S)/NO)$ vs. $E^{\circ\prime}((Fc^+/Fc)\ S)$ / V
DCM	20	1.41
	50	1.40
	100	1.40
	200	1.40
DCE	20	1.24
	50	1.23
	100	1.23
	200	1.23
DMF	20	0.54
	50	0.54
	100	0.54
	200	0.54
Nitromethane	20	1.00
	50	0.99
	100	0.99
	200	0.99
PC	20	0.82
	50	0.81
	100	0.81
	200	0.82
AN	20	0.88
	50	0.87
	100	0.87
	200	0.87

The conclusion from Supplementary Table 16 is that the half-wave potential of NO^+/NO in these solvents, as already found for the fluorinated arenes, are almost independent from the scan rate v. The differences, which can be found in the table above, are based on rounding errors due to the rounding to two decimal spaces.

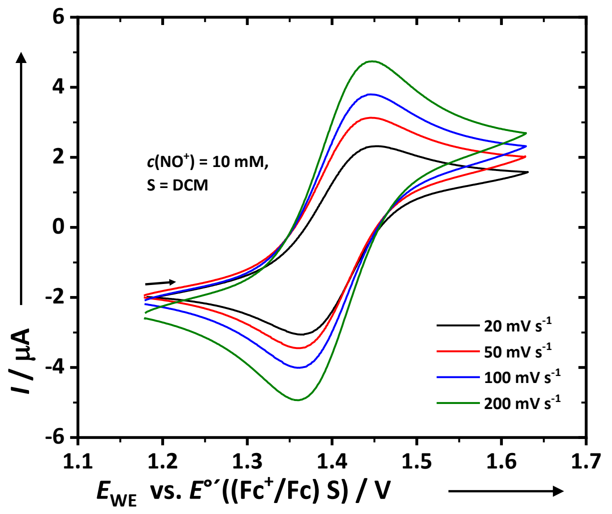

Supplementary Figure 66: 2nd cycles of NO^+ (10 mM) in S = DCM versus $E°´$((Fc+/Fc) S) measured at four different scan rates (20, 50, 100 & 200 mV s^{-1}). The black arrow indicates for all scan rates the start and the direction of the 2nd cycle.

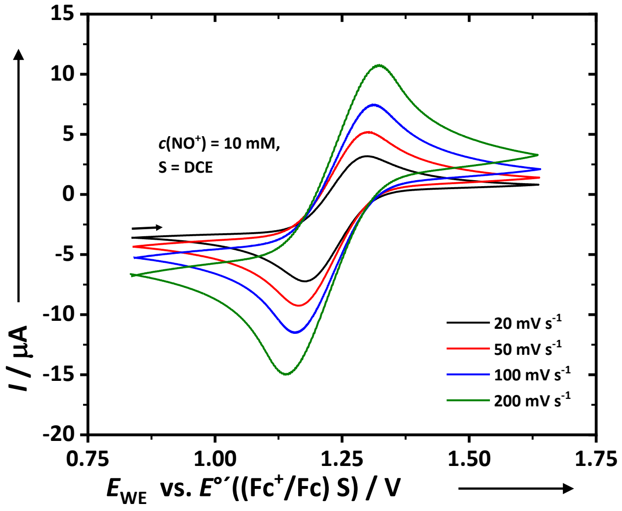

Supplementary Figure 67: 2nd cycles of NO^+ (10 mM) in S = DCE versus $E°´$((Fc+/Fc) S) measured at four different scan rates (20, 50, 100 & 200 mV s^{-1}). The black arrow indicates for all scan rates the start and the direction of the 2nd cycle.

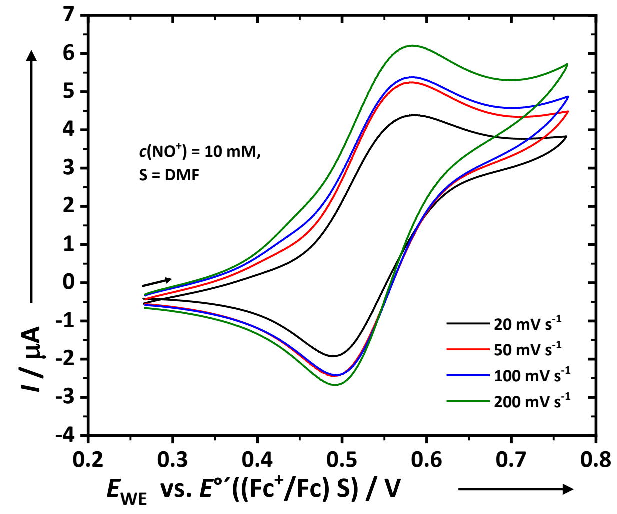

Supplementary Figure 68: 2nd cycles of NO^+ (10 mM) in S = DMF versus E°´((Fc^+/Fc) S) measured at four different scan rates (20, 50, 100 & 200 mV s^{-1}). The black arrow indicates for all scan rates the start and the direction of the 2nd cycle.

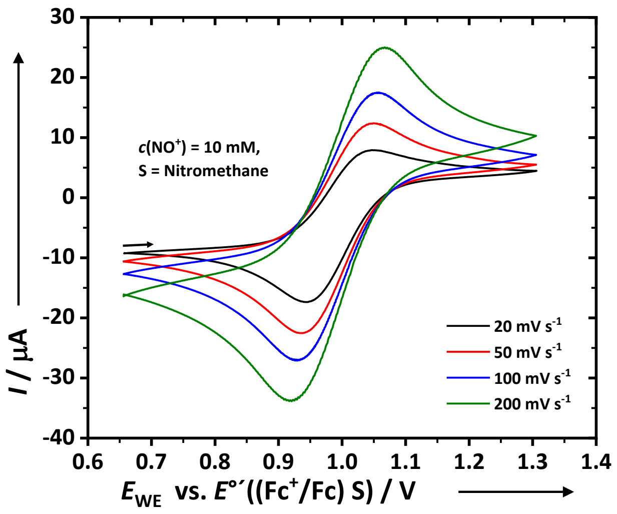

Supplementary Figure 69: 2nd cycles of NO^+ (10 mM) in S = Nitromethane versus E°´((Fc^+/Fc) S) measured at four different scan rates (20, 50, 100 & 200 mV s^{-1}). The black arrow indicates for all scan rates the start and the direction of the 2nd cycle.

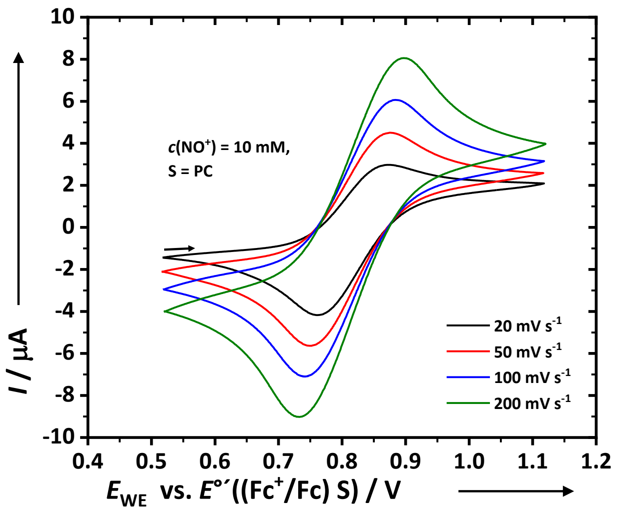

Supplementary Figure 70: 2nd cycles of NO^+ (10 mM) in S = PC versus $E°´$((Fc$^+$/Fc) S) measured at four different scan rates (20, 50, 100 & 200 mV s^{-1}). The black arrow indicates for all scan rates the start and the direction of the 2nd cycle.

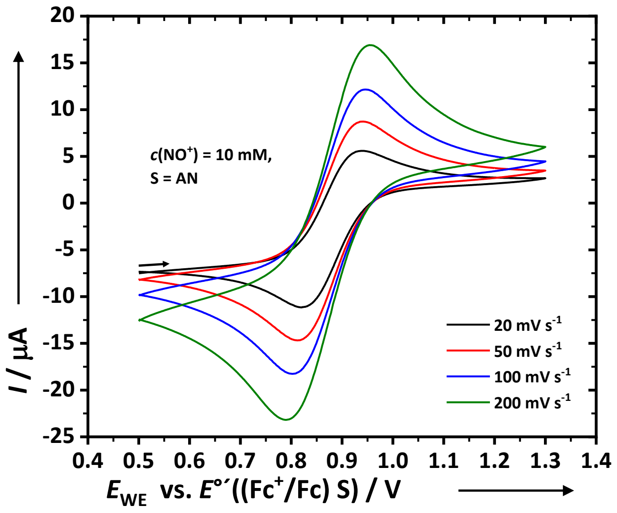

Supplementary Figure 71: 2nd cycles of NO^+ (10 mM) in S = AN versus $E°´$((Fc$^+$/Fc) S) measured at four different scan rates (20, 50, 100 & 200 mV s^{-1}). The black arrow indicates for all scan rates the start and the direction of the 2nd cycle.

3.6.1.1 $I_p \propto v^{1/2}$ Plot

Supplementary Table 17: Anodic peak current obtained by cyclic voltammetry of NO^+ in selected solvents at different scan rates *v* given together with $v^{1/2}$ and R^2 from the linear regression.

Solvent	$v^{1/2}$ / (mV s^{-1})$^{1/2}$	I_{pa} / µA	R^2
DCM	4.47	2.32	0.99444
	7.07	3.13	
	10.00	3.80	
	14.14	4.74	
DCE	4.47	3.18	0.99997
	7.07	5.21	
	10.00	7.49	
	14.14	10.78	
DMF	4.47	4.39	(0.94055)
	(7.07)	(5.24)	0.99920$
	10.00	5.39	
	14.14	6.21	
Nitromethane	4.47	7.91	0.99985
	7.07	12.40	
	10.00	17.50	
	14.14	25.01	
PC	4.47	2.98	0.99790
	7.07	4.50	
	10.00	6.07	
	14.14	8.06	
AN	4.47	5.59	0.99988
	7.07	8.72	
	10.00	12.15	
	14.14	16.90	

$ = Value without from $v^{1/2}$ = 7.07 (mV s^{-1})$^{1/2}$.

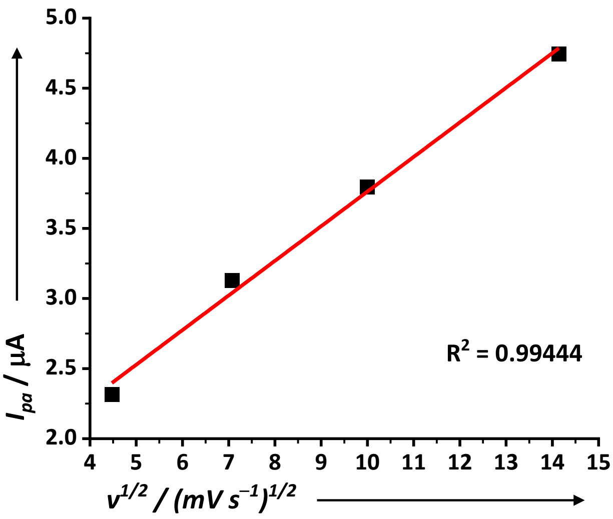

Supplementary Figure 72: Plot of the anodic peak current I_{pa} versus the sqaure root of the scan rate $v^{1/2}$ from the cyclic voltammograms of NO^+ vs. $E°'$((Fc$^+$/Fc) S) in S = DCM, together with the linear regression and corresponding R^2 value for the linear regression.

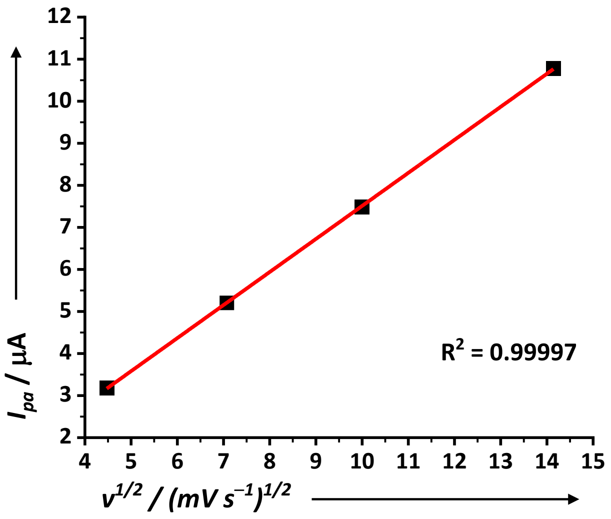

Supplementary Figure 73: Plot of the anodic peak current I_{pa} versus the square root of the scan rate $v^{1/2}$ from the cyclic voltammograms of NO^+ vs. $E°´$((Fc$^+$/Fc) S) in S = DCE together with the linear regression and corresponding R^2 value for the linear regression.

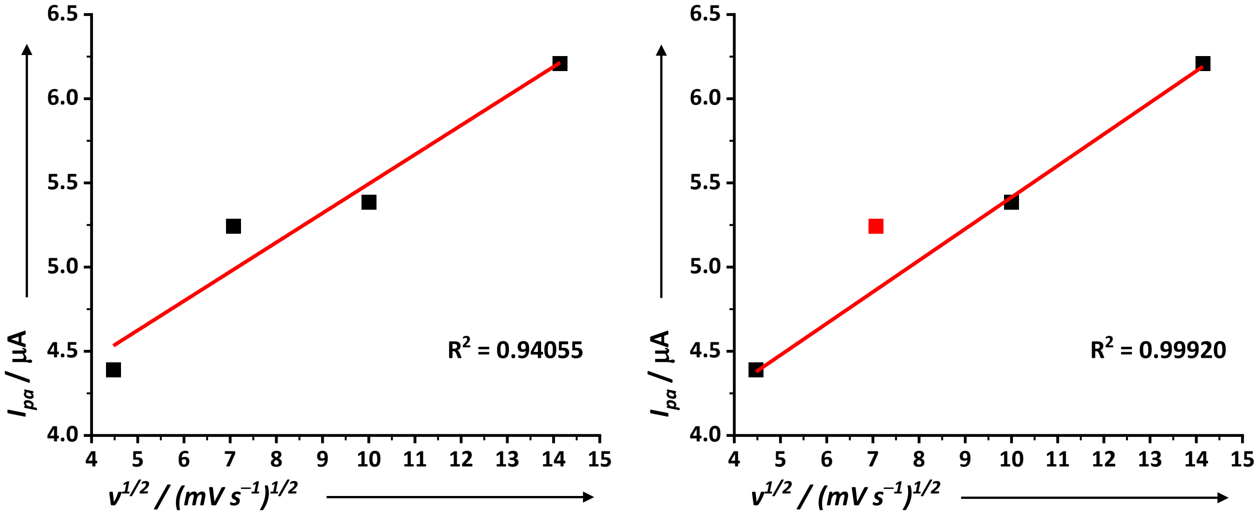

Supplementary Figure 74: Plots of the anodic peak current I_{pa} versus the square root of the scan rate $v^{1/2}$ from the cyclic voltammograms of NO^+ vs. $E°´$((Fc$^+$/Fc) S) in S = DMF, together with the linear regression and corresponding R^2 value for the linear regression. On the left side the linear regression was done with all four scan rates, the right sight was performed without the value for 50 mV s^{-1}.

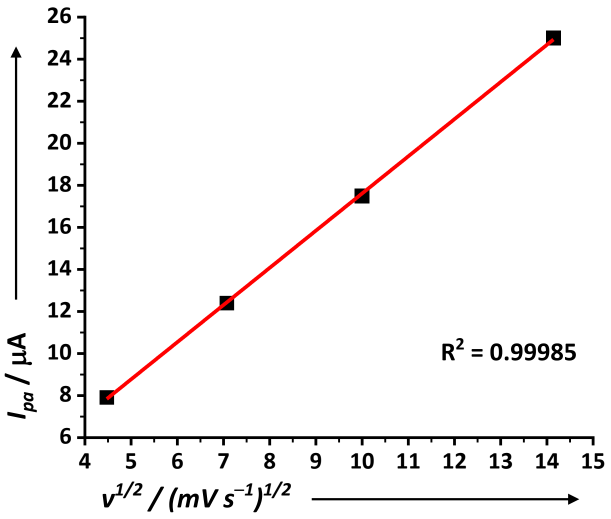

Supplementary Figure 75: Plot of the anodic peak current I_{pa} versus the square root of the scan rate $v^{1/2}$ from the cyclic voltammograms of NO^+ vs. $E°'((Fc^+/Fc)\ S)$ in S = Nitromethane, together with the linear regression and corresponding R^2 value for the linear regression.

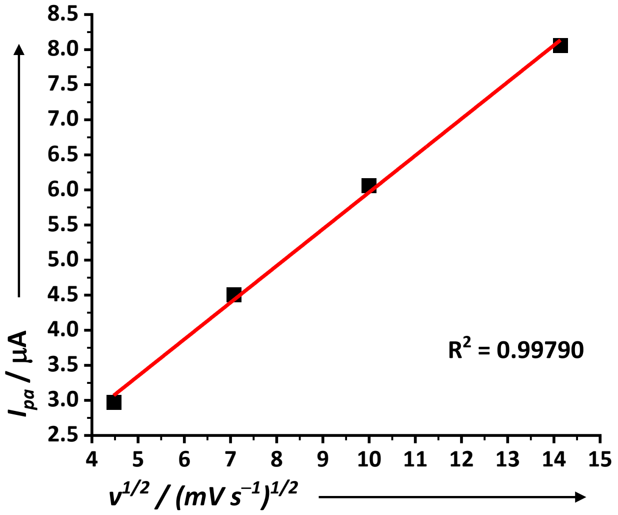

Supplementary Figure 76: Plot of the anodic peak current I_{pa} versus the square root of the scan rate $v^{1/2}$ from the cyclic voltammograms of NO^+ vs. $E°'((Fc^+/Fc)\ S)$ in S = PC, together with the linear regression and corresponding R^2 value for the linear regression.

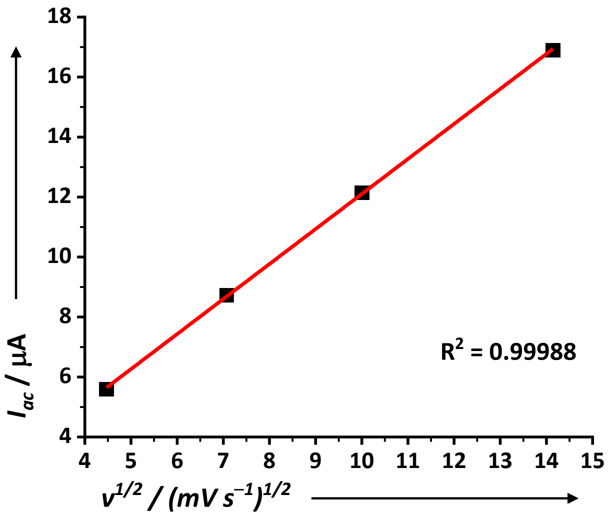

Supplementary Figure 77: Plot of the anodic peak current I_{pa} versus the square root of the scan rate $v^{1/2}$ from the cyclic voltammograms of NO^+ vs. $E°'((Fc^+/Fc)\ S)$ in S = AN, together with the linear regression and corresponding R^2 value for the linear regression.

3.6.1.2 Concentration Dependence of NO^+(DCM)/NO

The influence of the NO^+ concentration on the half-wave potential $E_{1/2}$(NO^+(DCM)/NO) was evaluated. CV measurements of NO^+[*pf*]$^-$ using four different concentrations (5, 10, 20 and 40 mM) were performed. The results are summarized in Supplementary Table 18 and illustrated in Supplementary Figure 78.

Supplementary Table 18: Measured $E_{1/2}$(NO^+(DCM)/NO) versus $E°'$((Fc^+/Fc) DCM) for four different NO^+ concentrations given at a scan rate of 100 mV s^{-1}.

c(NO^+[*pf*]$^-$) / mM	$E_{1/2}$(NO^+(DCM)/NO) vs. $E°'$((Fc^+/Fc) DCM) / V
40	1.39
20	1.40
10	1.38
5	1.41

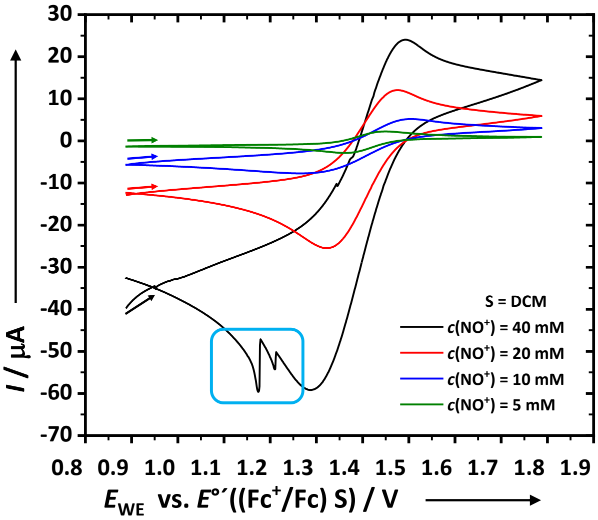

Supplementary Figure 78: 2nd cycles of the different concentrations (5, 10, 20 & 40 mM) of NO^+ in DCM versus $E°'$((Fc^+/Fc) DCM at a scan rate of 100 mV s^{-1}. The light blue box highlighted artifacts from evolving nitrogen monoxide, as the product of the reduction of NO^+. The different coloured arrows indicate the start and the direction of the same-coloured cycle.

As shown in Supplementary Figure 78 the concentration of NO^+ has no major impact on the half-wave potential and therefore on the activity *a* of NO^+ in the ensemble with the solvent DCM and the counterion [*pf*]$^-$. The small differences of $E_{1/2}$ can probably be attributed to measurement inaccuracies and rounding errors. It remains to mention that only at the highest concentration of 40 mM, artefacts from gaseous NO can be found during the CV measurement.

3.6.1.3 Comparison with Literature Data of NO^+(DCM)/NO

Lee *et al.* report the value $E°$(NO^+(DCM/NO) = 1.00 V vs. $E_{1/2}$((Fc^+/Fc) DCM) where they equate $E° = E_{1/2}$ due to the constancy of the values of $(E_{pc} + E_{pa})/2$ at the various CV scan rates used (cf. Section 3.2.1).[70] Thus, their $E°$ value has to be compared to our $E_{1/2}$ value, which is 1.4 V vs. $E°'$((Fc^+/Fc) DCM). For their measurements, Lee *et al.* intended to use $NO^+[BF_4]^-$ but they used NO gas for the insolubility of $NO^+[BF_4]^-$ in DCM; the supporting electrolyte they used was 0.1 M $[NBu_4]^+[PF_6]^-$. We could reproduce the value of Lee *et al.* within 0.05 V using 10 mM $NO^+[PF_6]^-$ and 0.1 M $[NBu_4]^+[PF_6]^-$ as supporting electrolyte, see Supplementary Table 19 and Supplementary Figure 79. Most probably, the difference can be explained with the use of different reference electrodes. Lee *et al.* used a saturated calomel electrode (SCE) and corrected for junction and reference potentials, which is still no straightforward operation even today.

In terms of the activity $a(NO^+, DCM)$ the difference $\Delta E_{pf,PF6} = E_{1/2}(NO[pf], DCM) – E_{1/2}(NO[PF_6], DCM) = 0.45$ V amounts to about 10^7, i.e. the NO^+ ion activity is 7 orders of magnitude lower in presence of the $[PF_6]^-$ ion. Therefore, it can be assumed that the $[PF_6]^-$ anion supplies a strongly bound contact-ion pair with NO^+ in solution. If the NO^+ ion is considered as naked in the presence of the $[pf]^-$ ion, i.e. it interacts only with solvent molecules, the ion pair formation constant K_f of the hypothetic reaction NO^+(solv, DCM) + $[PF_6]^-$(solv, DCM) ⟶ $NO^+[PF_6]^-$(solv, DCM) can be estimated to be $K_f > 10^7$, given that this ion-pair forms (and no other ion-tuple)

This comparison shows impressively to what extent measured potentials can depend on the counterion of the oxidized (or reduced or both) form(s) of the redox system (and hence of the supporting electrolyte). This effect is particularly important when using solvents with low permittivity and/or low coordination properties. Besides that, it emphazises the importance to distinguish $E°$ and $E°'$ (or $E_{1/2}$ if the diffusion coefficients are unknown) and to indicate the measurement conditions. Certainly, the published value by Lee et al. is not a standard potential value. In addition, since we cannot estimate how much the diffusion coefficient of the NO^+ ion differs from that of the neutral NO molecule (due to the high charge density of the NO^+ ion, it is likely to be much stronger solvated, resulting in a significantly different diffusion coefficient), we specify $E_{1/2}$ instead of $E°'$. If, for example, the diffusion coefficients differ by a factor of 10, $E_{1/2}$ appears shifted by 28mV relative to $E°'$.

Supplementary Table 19: Measured half-wave potential for NO^+(DCM)/NO from $NO^+[PF_6]^-$ versus $E°'((Fc^+/Fc)$ DCM) at a scan rate of 100 mV s^{-1}.[a]

$E_{1/2}(NO^+(DCM)/NO)$ vs. $E°'((Fc^+/Fc)$ DCM) / V
0.95

a) Due to solubility problems of $[NO]^+[PF_6]^-$ in dichloromethane, it was only possible to measure it at one scan rate.

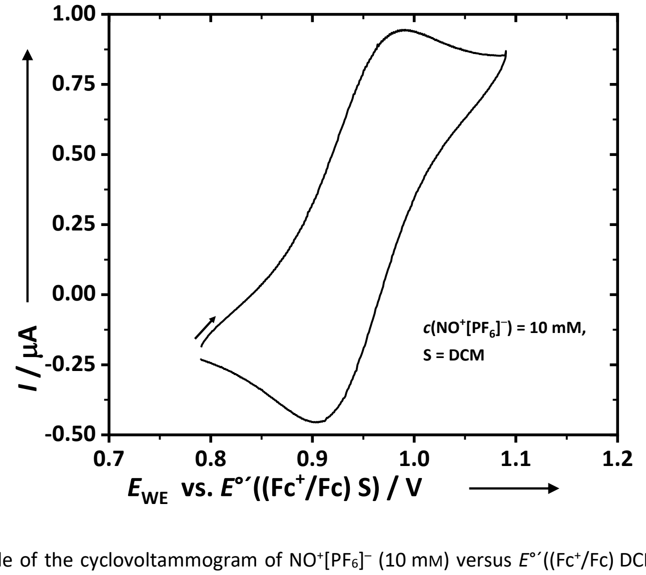

Supplementary Figure 79: 2nd cycle of the cyclovoltammogram of $NO^+[PF_6]^-$ (10 mM) versus $E°'((Fc^+/Fc)$ DCM) at a scan rate of 100 mV s^{-1} as an approach to reproduce the literature value from Lee *et al.*.[70] The black arrow indicates the start and the direction of the 2nd cycle.

3.6.2 ([TBPA]$^{\cdot+}$/TBPA) S

Analogous to the CV measurements of TBPA in the different fluorinated arenes, described in section 3.4.2, the corresponding CV measurements were performed for the selected solvents, except DCM and AN. For both solvents, Conelly and Geiger already provides a value for TBPA$^{\cdot+}$/TBPA (refer to section 3.4.2).[67]

The $E°'$(([TBPA]$^{\cdot+}$/TBPA) S) in the used solvents at four different scan rates are summarized in Supplementary Table 20 and visualised in Supplementary Figure 80-Supplementary Figure 84. As shown in Supplementary Table 20, the potentials are rarely influenced by the scan rate. Small differences can probably be attributed to measurement inaccuracies and rounding errors.

Supplementary Table 20: Measured $E°'$(([TBPA]$^{+}$/TBPA) S) vs. $E°'$((Fc^+/Fc) S) / V in selected solvents at different scan rates (*v*).

Solvent S	*v* / mV s^{-1}	$E°'$(([TBPA]$^{\cdot+}$/TBPA) S) vs. $E°'$((Fc^+/Fc) S) / V
DCE	20	0.68
	50	0.68
	100	0.69
	200	0.68
DMF	20	0.70
	50	0.70
	100	0.70
	200	0.70
Nitromethane	20	0.74
	50	0.75
	100	0.75
	200	0.75
PC	20	0.72
	50	0.71
	100	0.71
	200	0.72
THF	20	0.69
	50	0.69
	100	0.69
	200	0.68

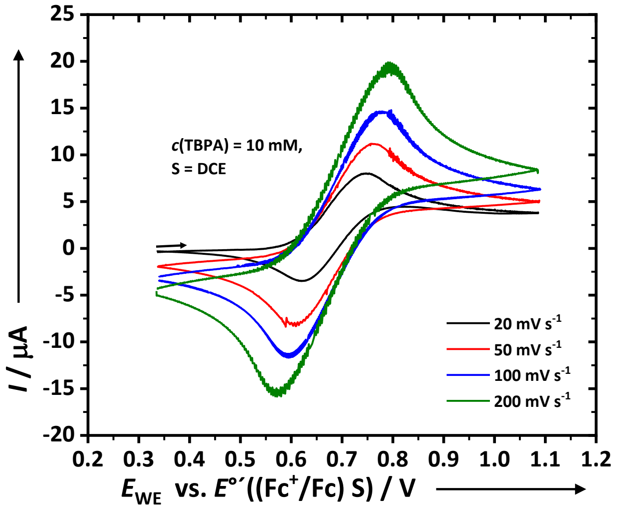

Supplementary Figure 80: 2nd cycles of TBPA (10 mM) in S = DCE versus $E°'$((Fc^+/Fc) S) measured at four different scan rates (20, 50, 100 & 200 mV s^{-}

¹). The black arrow indicates for all scan rates the start and the direction of the 2nd cycle.

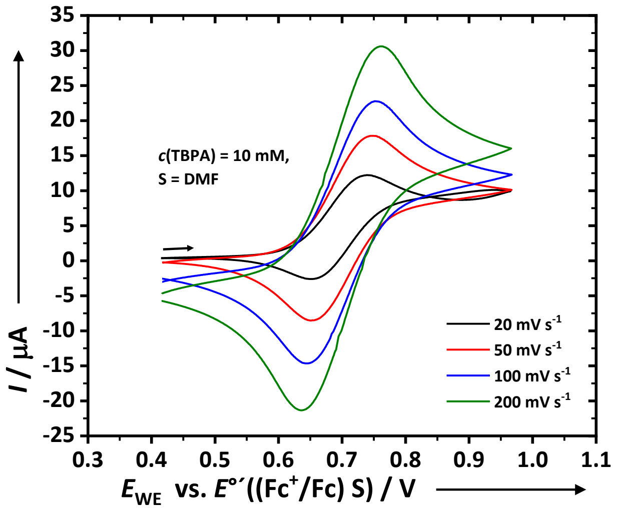

Supplementary Figure 81: 2nd cycles of TBPA (10 mм) in S = DMF versus $E°´$((Fc⁺/Fc) S) measured at four different scan rates (20, 50, 100 & 200 mV s^{-1}). The black arrow indicates for all scan rates the start and the direction of the 2nd cycle.

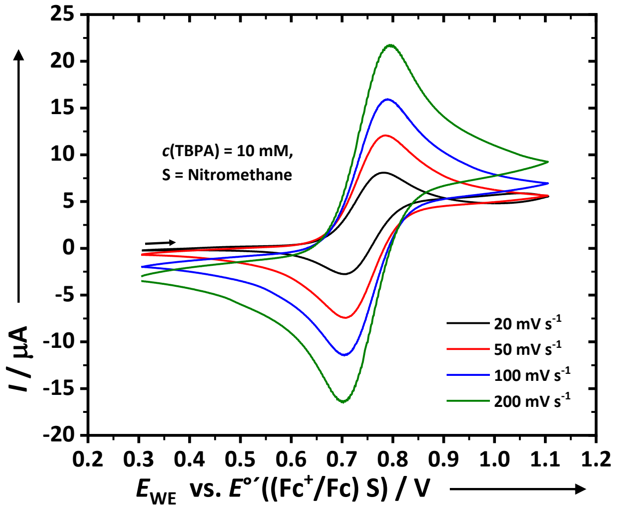

Supplementary Figure 82: 2nd cycles of TBPA (10 mм) in S = Nitromethane versus $E°´$((Fc⁺/Fc) S) measured at four different scan rates (20, 50, 100 & 200 mV s^{-1}). The black arrow indicates for all scan rates the start and the direction of the 2nd cycle.

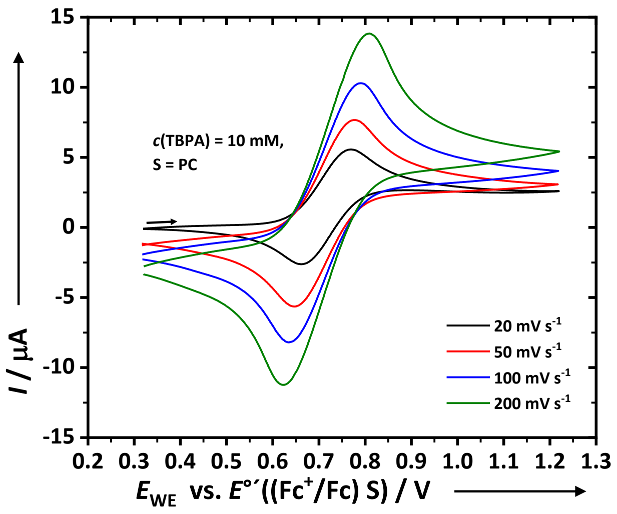

Supplementary Figure 83: 2nd cycles of TBPA (10 mM) in S = PC versus $E°´$((Fc+/Fc) S) measured at four different scan rates (20, 50, 100 & 200 mV s^{-1}). The black arrow indicates for all scan rates the start and the direction of the 2nd cycle.

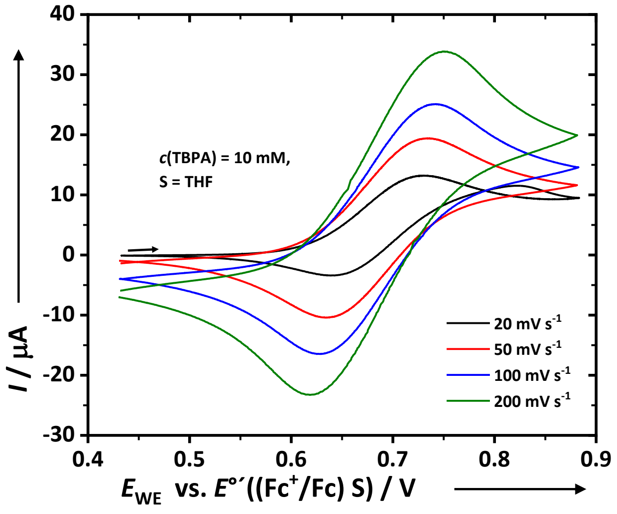

Supplementary Figure 84: 2nd cycles of TBPA (10 mM) in S = THF versus $E°´$((Fc+/Fc) S) measured at four different scan rates (20, 50, 100 & 200 mV s^{-1}). The black arrow indicates for all scan rates the start and the direction of the 2nd cycle.

3.7 Measurements of Redox Systems in Selected Solvents Using the Ag-Compartment Reference

This section contains measurements of the redox systems (Fc^+/Fc) S and NO^+(S)/NO. The used reference electrode was the Ag-compartment reference as described in Section 3.1. Thus, the formal potential of the Ag^+/Ag redox system can be given with respect to the formal potential of the Fc^+/Fc redox system, $E°´(Ag^+(S)/Ag) = -(E_m + RT/F \cdot \ln c(Ag^+, S)) = -(E_m - 0{,}118\ V)$, with E_m is the measured potential $E_{1/2}((Fc^+/Fc)\ S)$ vs. $E(Ag^+(S)/Ag)$.

3.7.1 (Fc^+/Fc) S

The experimental values of $E_{1/2}((Fc^+/Fc)\ S)$ and corresponding calculated value $E°´(Ag^+(S)/Ag)$ are summarized inSupplementary Table 21, reported together with solvent (S) and scan rate v. Additionally, the experimental results are illustrated in Supplementary Figure 85-Supplementary Figure 89 for each solvent.

Supplementary Table 21: Measured half-wave potentials $E_{1/2}(Fc^+/Fc, S)$ versus $E(Ag^+(10\ \text{mM})/Ag, S)$ and the calculated potentials $E°´(Ag^+/Ag, S)$ vs. $E°´(Fc^+/Fc, S)$ in selected solvents at different scan rates (v).

Solvent S	v / mV s^{-1}	$E_{1/2}((Fc^+/Fc)\ S)$ vs. $E(Ag^+(10\ \text{mM}, S)/Ag)$ / V	$E°´(Ag^+(S)/Ag)$ vs. $E°´((Fc^+/Fc)\ S)$ / V
DCM	20	–0.77	0.88
	50	–0.76	0.88
	100	–0.76	0.88
	200	–0.76	0.88
DCE	20	–0.54	0.66
	50	–0.54	0.66
	100	–0.55	0.66
	200	–0.55	0.66
DMF	20	0.02	0.10
	50	0.01	0.11
	100	0.01	0.11
	200	0.01	0.11
THF	20	–0.18	0.29
	50	–0.18	0.30
	100	–0.18	0.29
	200	–0.18	0.30
AN	20	0.09	0.02
	50	0.09	0.03
	100	0.09	0.02
	200	0.09	0.03

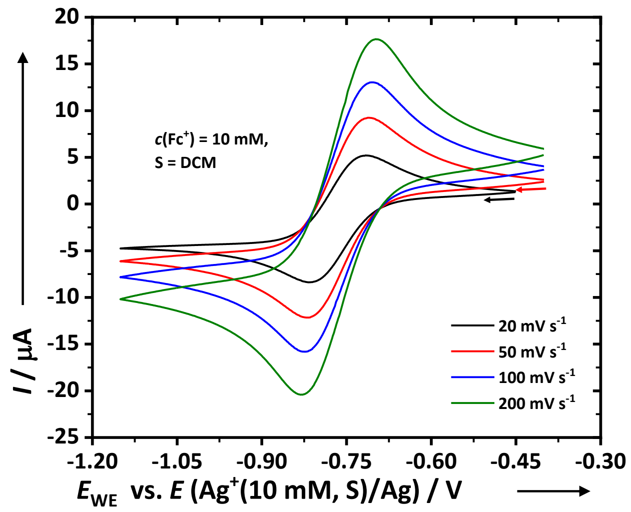

Supplementary Figure 85: 2nd cycles of Fc^+ (10 mM) in S = DCM versus *E*(Ag^+(10 mM, S)/Ag) measured at four different scan rates (20, 50, 100 & 200 mV s^{-1}). The black arrow indicates for the scan rate 20 mV s^{-1} the start and the direction of the cycle. The red arrow indicates the same for the red, blue and green cycle.

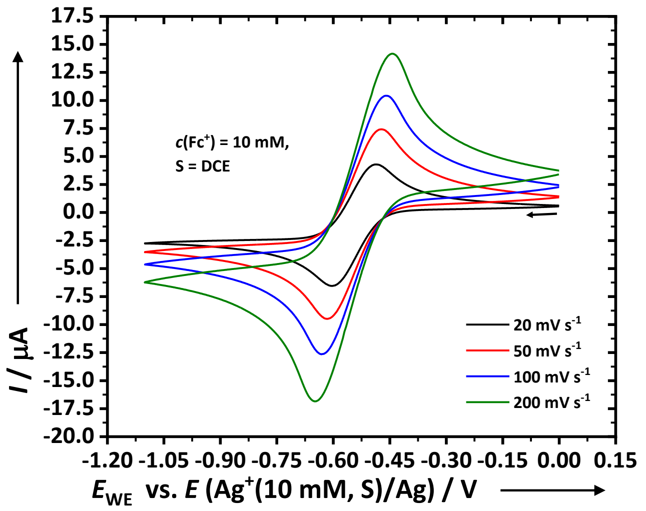

Supplementary Figure 86: 2nd cycles of Fc^+ (10 mM) in S = DCE versus *E*(Ag^+(10 mM, S)/Ag) measured at four different scan rates (20, 50, 100 & 200 mV s^{-1}). The black arrow indicates for all scan rates the start and the direction of the 2nd cycle.

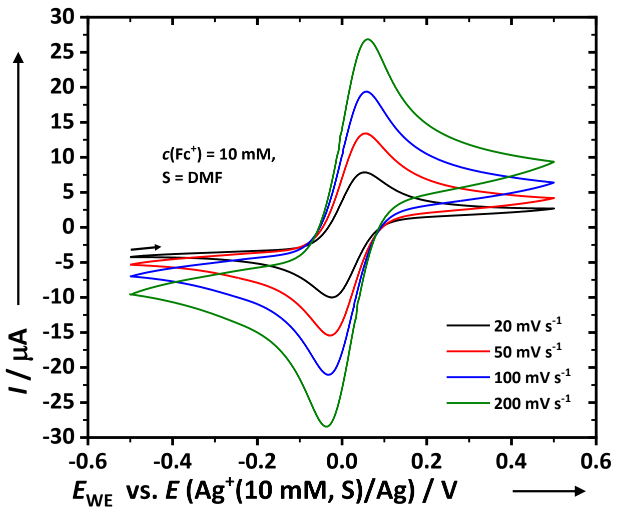

Supplementary Figure 87: 2nd cycles of Fc^+ (10 mM) in S = DMF versus $E(Ag^+(10\ mM,\ S)/Ag)$ measured at four different scan rates (20, 50, 100 & 200 mV s^{-1}). The black arrow indicates for all scan rates the start and the direction of the 2nd cycle.

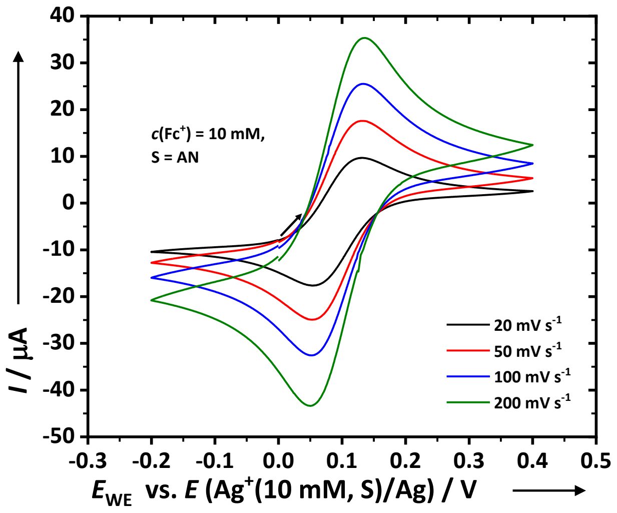

Supplementary Figure 88: 2nd cycles of Fc^+ (10 mM) in S = AN versus $E(Ag^+(10\ mM,\ S)/Ag)$ measured at four different scan rates (20, 50, 100 & 200 mV s^{-1}). The black arrow indicates for all scan rates the start and the direction of the 2nd cycle.

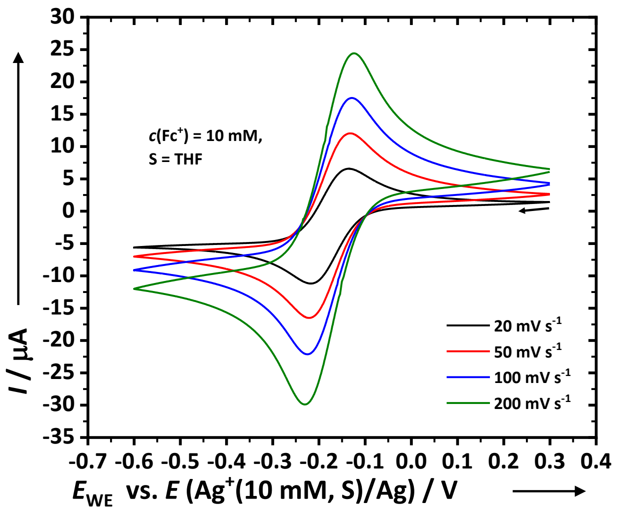

Supplementary Figure 89: 2nd cycles of Fc^+ (10 mM) in S = THF versus *E*(Ag^+(10 mM, S)/Ag) measured at four different scan rates (20, 50, 100 & 200 mV s^{-1}). The black arrow indicates for all scan rates the start and the direction of the 2nd cycle.

3.7.2 NO^+(S)/NO

The results for the corresponding CV measurement of NO^+(S)/NO with the Ag-compartment reference (and recalculated to $E°´(Ag^+(S)/Ag)$ as above) can be found in Supplementary Table 22, Supplementary Figure 90–Supplementary Figure 93 show the CVs.

Supplementary Table 22: Measured half-wave potentials of NO^+ versus $E(Ag^+(10\ \text{mM}, S)/Ag)$ and calculated values for $E_{1/2}(NO^+(S)/NO)$ versus $E°´(Ag^+(S)/Ag)$ / V in selected solvents at different scan rates (v).

Solvent S	v / mV s^{-1}	$E_{1/2}(NO^+(S)/NO)$ vs. $E(Ag^+(10\ \text{mM}, S)/Ag)$ / V	$E_{1/2}(NO^+(S)/NO)$ vs. $E°´(Ag^+(S)/Ag)$ / V
DCM	20	0.65	0.53
	50	0.64	0.53
	100	0.64	0.52
	200	0.64	0.52
DCE	20	0.71	0.59
	50	0.71	0.59
	100	0.71	0.59
	200	0.71	0.59
DMF	20	0.57	0.45
	50	0.57	0.45
	100	0.56	0.44
	200	0.56	0.44
AN	20	0.97	0.85
	50	0.97	0.85
	100	0.97	0.85
	200	0.97	0.85

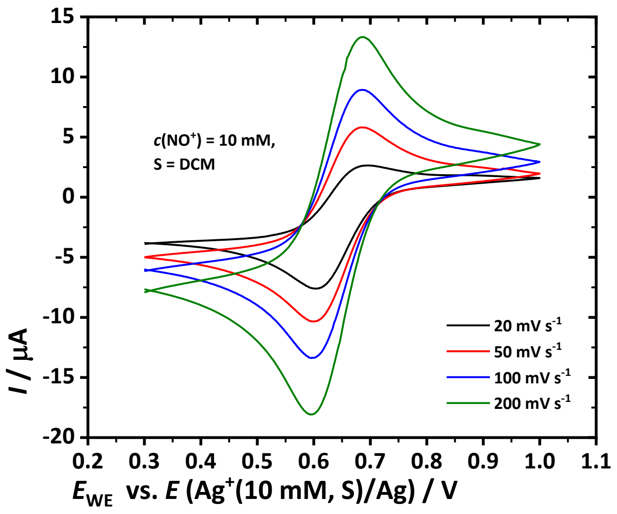

Supplementary Figure 90: 2nd cycles of NO^+ (10 mM) in S = DCM versus $E(Ag^+(10\ \text{mM}, S)/Ag)$ measured at four different scan rates (20, 50, 100 & 200 mV s^{-1}). The black arrow indicates for all scan rates the start and the direction of the 2nd cycle.

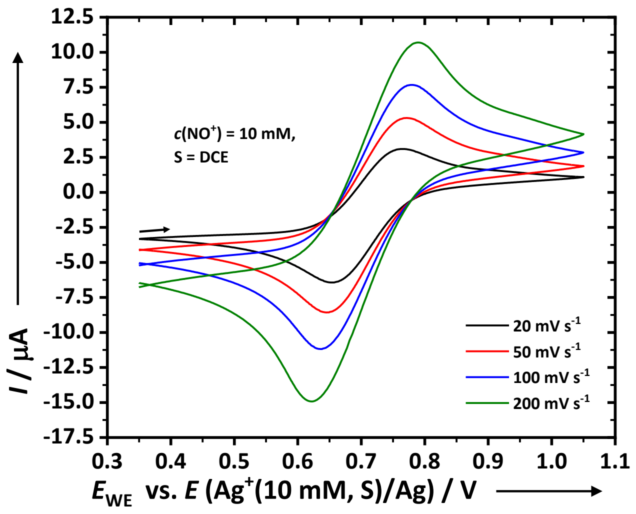

Supplementary Figure 91: 2nd cycles of NO^+ (10 mM) in S = DCE versus *E*(Ag^+(10 mM, S)/Ag) measured at four different scan rates (20, 50, 100 & 200 mV s^{-1}). The black arrow indicates for all scan rates the start and the direction of the 2nd cycle.

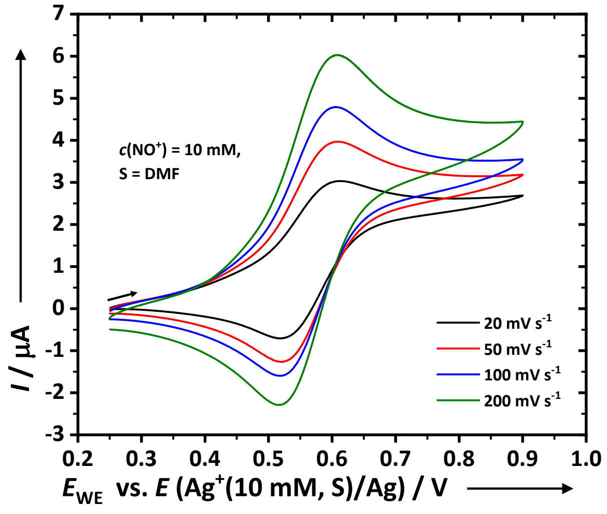

Supplementary Figure 92: 2nd cycles of NO^+ (10 mM) in S = DMF versus *E*(Ag^+(10 mM, S)/Ag) measured at four different scan rates (20, 50, 100 & 200 mV s^{-1}). The black arrow indicates for all scan rates the start and the direction of the 2nd cycle.

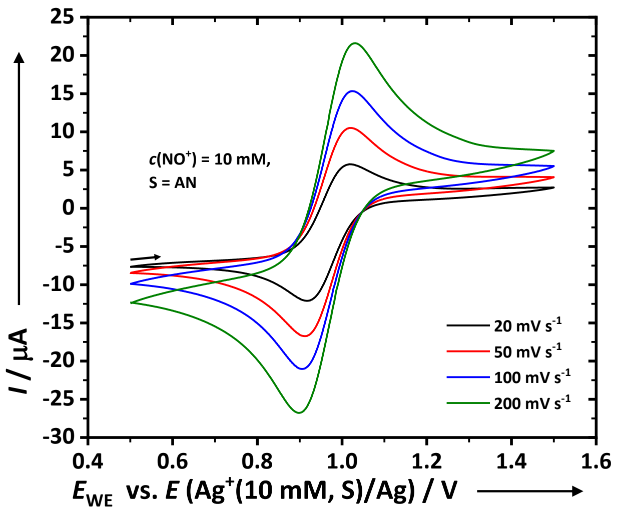

Supplementary Figure 93: 2nd cycles of NO^+ (10 mM) in S = AN versus E(Ag^+(10 mM, S)/Ag) measured at four different scan rates (20, 50, 100 & 200 mV s^{-1}). The black arrow indicates for all scan rates the start and the direction of the 2nd cycle.

3.7.3 Triangular Born-Fajans-Haber-Cycles of the Redox Systems NO^+/NO – Ag^+/Ag – Fc^+/Fc

The results presented in Sections 3.6.1 (i.e. $E_{1/2}$(NO^+(S)/NO) vs. $E°´$((Fc^+/Fc) S), 3.7.1 (i.e. $E_{1/2}$((Fc^+/Fc) S) vs. $E°´$(Ag^+(S)/Ag), and 3.7.2 (i.e. $E_{1/2}$(NO^+(S)/NO) vs. E(Ag^+(S)/Ag) allow the presentation of a triangular Born-Fajans-Haber-Cycles to assess the consistency of our measurements. Perfect consistent data must show, for example, $\Delta E \coloneqq 0 = E°´$($Ag^+$(S)/Ag) vs. $E°´$((Fc^+/Fc) S) + $E_{1/2}$(NO^+(S)/NO) vs. $E°´$(Ag^+(S)/Ag) − $E_{1/2}$(NO^+(S)/NO) vs. $E°´$((Fc^+/Fc) S), and the more the value deviates from zero the less consistent the data are.

Supplementary Table 23: Measured $E_{1/2}$(NO^+(S)/NO) recalculated value of $E°´$(Ag^+(S)/Ag) versus $E°´$((Fc^+/Fc) S) and the recalculated value of $E_{1/2}$(NO^+(S)/NO) versus $E°´$(Ag^+(S)/Ag) in DCM, DCE, DMF and AN at a scan rate of 100 mV s^{-1}, together with the value of the calculated potential difference (ΔE) and the value of the corresponding Gibbs Energy difference (ΔG). S represents the used solvent.

Solvent S	$E_{1/2}$(NO^+(S)/NO) vs. $E°´$((Fc^+/Fc) S) / V	$E°´$(Ag^+(S)/Ag) vs. $E°´$((Fc^+/Fc) S) / V	$E_{1/2}$(NO^+(S)/NO) vs. $E°´$(Ag^+(S)/Ag) / V	$\lvert\Delta E\rvert$ / V	$\lvert\Delta G\rvert^a$ / kJ mol^{-1}
DCM	1.40	0.88	0.52	0.00	0.00
DCE	1.23	0.66	0.59	0.02	2.07
DMF	0.54	0.11	0.44	0.01	0.87
AN	0.87	0.02	0.85	0.00	0.15

a) Values were calculated by $\Delta G = -nF\Delta E$, with n = number of electrons, ΔE = potential difference, F = Faraday constant = 96485 C mol^{-1} [68].

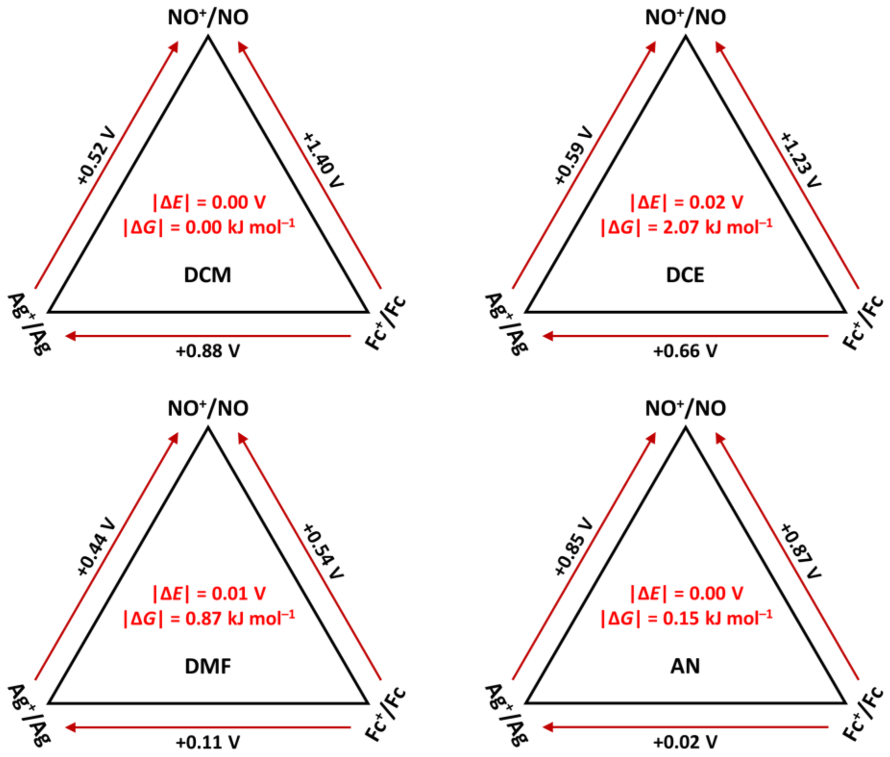

Supplementary Figure 94: Triangle diagrams of the three redox couples NO^+/NO, Ag^+/Ag und Fc^+/Fc in DCM, DCE, DMF and AN. The mean errors of the calculated potential difference (Δ*E*) / the corresponding Gibbs Energy difference (Δ*G*) are given in red in the center of the triangle.

3.8 Reactions of Ag[*pf*] with anthracene^{F}- and anthracene^{Hal}-solution in 4FB

Ag[*pf*] (70 mg, 65 µmol, 1.0 eq.) and anthracene^{F} (23 mg, 0.065 µmol, 1.0 eq.) were separately dissolved in 4FB (ca. 1.5 mL). The solution containing Ag[*pf*] was added to the anthracene^{F}-solution within 30 seconds. A colour change from yellow to dark green-blue was observed (Supplementary Figure 95).

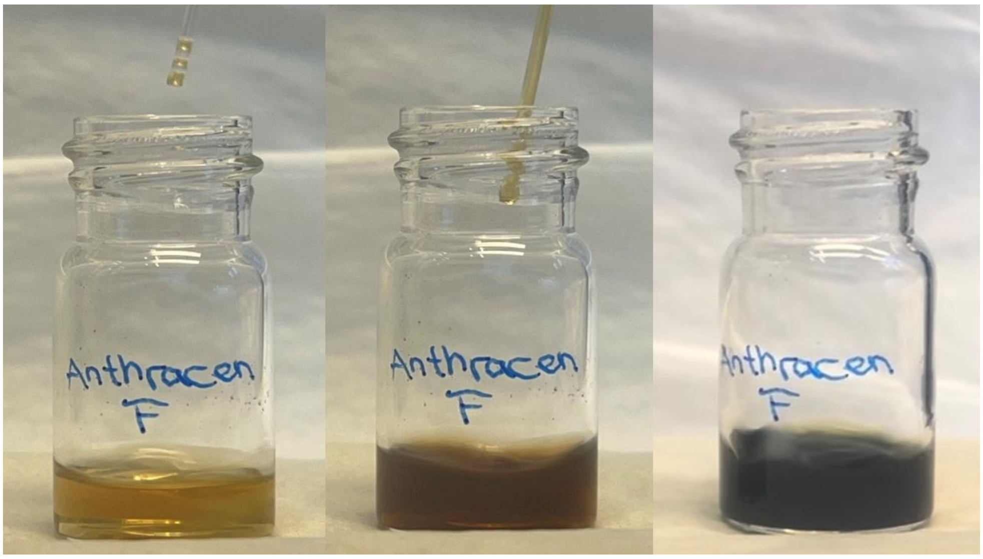

Supplementary Figure 95: Dropwise addition of an Ag[*pf*] solution in 4FB to anthracene^{F} dissolved in 4FB.

Ag[*pf*] (71 mg, 66 µmol, 1.0 eq.) and anthracene^{Hal} (26 mg, 0.066 µmol, 1.0 eq.) were separately dissolved in 4FB (ca. 1.5 mL). The solution containing Ag[*pf*] was added to the anthracene^{Hal}-solution within 30 seconds. A colour change from yellow to dark green-blue was observed (Supplementary Figure 96).

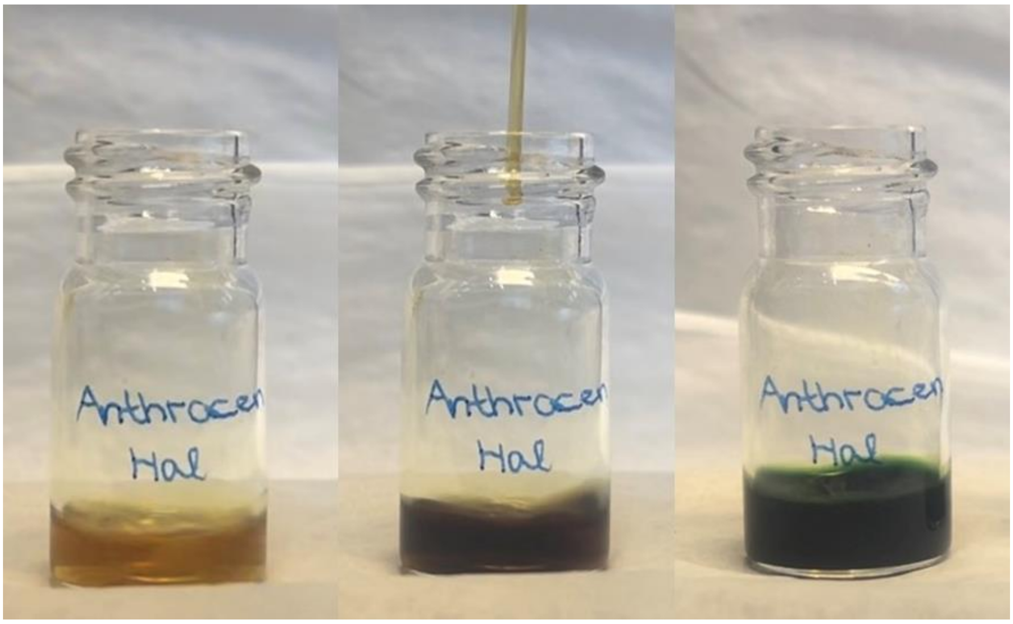

Supplementary Figure 96: Dropwise addition of an Ag[*pf*] solution in 4FB to anthracene^{Hal} dissolved in 4FB.

4 Supplementary Note 4

4.1 General Remarks

Standard enthalpy $H°$ and Gibbs energy $G°$ were calculated according to eq. (S1) and (S2) at 298.15 K and 0.1 MPa.

$$H° = E_{SCF} + E_{vrt} + R \cdot T \tag{S1}$$

$$G° = H° - T \cdot S° \tag{S2}$$

E_{SCF} represent the electronic energy, E_{vrt} is the sum of translation, rotational, and vibrational energy incl. zero-point energy, R is the ideal gas constant, T is the temperature given in Kelvin and $S°$ is the standard entropy.

Note, for a monoatomic structure, E_{vrt} is equal to $\frac{3}{2}RT$ and the standard entropy has to be calculated with the Sackur-Tetrode equation.[71]

The Gibbs energies of solvation $\Delta G_{(\mathrm{solv})}$ were calculated by using COSMO-RS[31–33] model using COSMOThermX software[35].

Therefore $G°_{(solv)}$ was calculated according to eq. (S3).

$$G°_{(solv)} = G° + \Delta G_{solv} \tag{S3}$$

Additionally, $H°_{(solv)}$ was determined using COSMOThermX software[35]. The determination of $H°_{(solv)}$ will be explained in the corresponding section.

4.1.1 General Remarks towards the used DFT functional

Due to the high number of structures to be calculated, the settings of the calculations were determined using a sample calculation from the Ag^+ part. As sample calculation, the dissociation of {Ag[*pf*]}$_{ip.}$ to the ions Ag^+ and [*pf*]$^-$ in the gas phase was used for both DFT functionals BP86[4,5] and B3LYP[6–8], which used the same basis set def2-TZVPP[9]. Additionally, single point calculations using DLPNO-CCSD(T)[19–21] with cc-pVQZ[22–24] and cc-pwCVQZ-PP[28], together with their corresponding auxiliary basis sets, were performed based on the BP86 or B3LYP structures, respectively. The result of the comparison is summarized in the table below.

Supplementary Table 24: Summary of the comparison between the DFT functionals BP86 and B3LYP for the dissociation of {Ag[*pf*]}$_{ip.}$ into the ions Ag^+ and [*pf*]$^-$.

Calculated thermodynamics[a)] → ↓ Structure obtained by DFT functional:	$\Delta G_r°_{(g)}$ / kJ mol^{-1}	$\Delta H_r°_{(g)}$ / kJ mol^{-1}
BP86	413.60	452.39
B3LYP	412.45	452.29

a) Calculated using either a BP86/def2-TZVPP or a B3LYP/def2-TZVPP structure as the basis for a series of single point calculation at the DLPNO-CCSD(T)/cc-pVQZ for the gas phase energies at 0 K in vaccum and enthalpic / entropic contributions at the respective DFT level to correct to standard conditions.

The comparison shows almost similar values for $\Delta G_r°_{(g)}$ and $\Delta H_r°_{(g)}$, for the used sample calculation. Therefore, for further calculations only the DFT functional BP86 based structures were used through this work. The usage of BP86 was adopted for the NO^+ related calculations without further review.

4.1.2 Comparison between the two possible solvation models CPCM and COSMO-RS

The conductor-like polarizable continuum model, short CPCM, places the dissolved substance in a roughly molecular shaped cavity. The cavity itself is located in the bulk solvent, which is treated as a conductor-like polarizable continuum. Therefore, the interaction from the solvent, which has to be at least described by the permittivity, with the dissolved substance, can be described as the interaction of polarization charges on the cavity surface from the substance. These charges can be treated as point charges or spherical Gaussians.[72] In our case, these charges were treated as punctual. It is worth to mention, that this differentiation is important for the construction of the cavity performed ORCA.[16–18] For further details, the original literature from Cossi *et al.*[73] and the ORCA manual can be recommended.

The conductor-like screening model for real solvents, short COSMO-RS,[32] is an extension of the COSMO (conductor-like screening model) solvation model, which was first published by Schüürmann *et al.*.[74] For a better understanding of the COSMO-RS solvation model, previously a short explanation about the COSMO model is required. The COSMO model sets the permittivity to infinite, instead of a finite value for each solvent. Therefore, a scaling function of ε is required, to take the different permittivities into account. The determination of the scaling function was performed by an empirical comparison between unscaled (COSMO) and correct electrostatic solute-solvent energies, which results in the following formula:

$$f(\varepsilon) = \frac{\varepsilon - 1}{\varepsilon + k}, with\ k\ small. \qquad \text{(S4)}$$

At this point, it should be mentioned, that Cossi *et al.* set for the CPCM solvation model, the value of k equal to zero, based on an analogy with the Gauss law[73,75] and Klamt *et al.* in the original publication for COSMO from 1993, *k* was set to 0.5, with the remark that *k* may be influenced by the dissolved substance and the resulting cavity shape and distribution of charges.[74]

The value of parameter *k* was manifold discussed by different authors through the literature, which can comprehended in the review article of Cammi *et al.* from 2005.[75] To sum up, the influence of *k* is decreased with an increased permittivity ε and increased with a decreased permittivity, respectively.

Based on the COSMO solvation model, Klamt *et al.*[32] introduced the COSMO-RS model, where the interaction in a non-linear behaved fluid is described as a local contact interaction of molecular surfaces and all interactions are reduced to local interactions between two surface segments. Through the usage of such pairwise surface interactions and experimental data, the solvation model COSMO-RS can calculate standard thermodynamic data of fluids, like vapor pressures, with corresponding computational software COSMOthermX.[35,75]

In this article, and after validation (see section 4.5) with the ionization energies and deelectronation potentials obtained accurately with the COSMO-RS model, exclusively the COSMO-RS model was used to calculate solvation energies.

4.1.3 Input file for Ag related single point calculations

Below is a general input file for single point calculations using Orca software[16–18] for the cc-pV**X**Z (**X**= D, T, Q) basis sets used for silver containing structures.

```
!dlpno-ccsd(t) cc-pVXZ cc-pVXZ/C printbasis tightscf tightpno UNO UCO RIJCOSX
%maxcore memory in MB
%pal
nprocs number of cors end

%basis
  NewGTO Ag  "cc-pwCVXZ-PP" end
  NewECP AG  "SK-MCDHF-RSC" end
  NewAuxCGTO Ag "cc-pwCVXZ-PP/C" end
end

%method
   frozencore FC_ELECTRONS
end

%mdci
   UseFullLmp2Guess false
end

*xyzfile Charge Multiplicity coord.xyz
```

4.2 Summarization of all quantum chemical calculations results

Supplementary Table 25: Summary of thermodynamic data calculated at the DLPNO-CCSD(T)/CBS and BP86(D3BJ)/def2-TZVPP levels of theory.

Structure (Symmetry)	E_{SCF} (DLPNO-CCSD(T)/CBS))[$] / H	E_{vrt} (BP86) / kJ mol^{-1}	S^0(BP86) / kJ mol^{-1}	$G^0_{(g)}$ / kJ mol^{-1}	$H^0_{(g)}$ / kJ mol^{-1}
**NO^+(C_{6v})**	–129.43392	20.31	0.19845	–339865.07	–339805.90
**Ag^+(O_h)**	–146.63292	3.72[§]	0.16723[§]	–385028.32	–384978.46
**[*pf*]$^-$ (C_1)**	–4744.18760	704.28	1.36743	–12455563.12	–12455155.42
**{Ag[*pf*]}$_{ip.}$ (C_1)**	–4890.98990	713.16	1.40457	–12840994.68	–12840575.91
**5FB (C_{2v})**	–727.76100	175.10	0.38152	–1910672.32	–1910558.57
**$[NO(5FB)_1]^+$ (C_1)**	–857.21587	201.22	0.46015	–2250553.33	–2250416.13
**$[NO(5FB)_2]^+$ (C_1)**	–1584.99015	384.59	0.68030	–4161206.61	–4161003.78
**$[Ag(5FB)_1]^+$ (C_1)**	–874.42459	182.97	0.44883	–2295749.70	–2295615.88
**$[Ag(5FB)_2]^+$ (C_1)**	–1602.21829	365.94	0.70313	–4206464.55	–4206254.91
**$[Ag(5FB)_3]^+$ (C_1)**	–2329.99812	548.58	0.92036	–6117132.25	–6116857.84
**{(5FB)Ag[*pf*]}$_{ip.}$ (C_1)**	–5618.76946	896.07	1.59862	–14751654.49	–14751177.86
**4FB (C_{2v})**	–628.59354	193.55	0.36206	–1650283.95	–1650176.01
**$[NO(4FB)_1]^+$ (C_1)**	–758.05405	219.88	0.43586	–1990178.13	–1990048.18
**$[NO(4FB)_2]^+$ (C_1)**	–1386.66296	421.56	0.65082	–3640452.91	–3640258.87
**$[Ag(4FB)_1]^+$ (C_1)**	–775.26351	201.38	0.43538	–2035379.91	–2035250.10
**$[Ag(4FB)_2]^+$ (C_2)**	–1403.89476	403.09	0.66128	–3685716.57	–3685519.41
**$[Ag(4FB)_3]^+$ (C_1)**	–2032.50878	604.37	0.87696	–5336005.41	–5335743.95
**{(4FB)Ag[*pf*]}$_{ip.}$ (C_1)**	–5519.60677	914.68	1.58014	–14491278.79	–14490807.67
**3FB (C_{2v})**	–529.42496	212.07	0.34270	–1389892.60	–1389790.43
**$[NO(3FB)_1]^+$ (C_s)**	–658.89000	238.36	0.42284	–1729800.60	–1729674.53
**$[NO(3FB)_2]^+$ (C_1)**	–1188.33410	458.97	0.61159	–3119691.47	–3119509.13
**$[Ag(3FB)_1]^+$ (C_1)**	–676.10174	220.06	0.42035	–1775007.56	–1774882.23
**$[Ag(3FB)_2]^+$ (C_1)**	–1205.57018	440.55	0.63424	–3164969.97	–3164780.87
**$[Ag(3FB)_3]^+$ (C_1)**	–1735.01563	660.71	0.82978	–4554866.88	–4554619.48
**{(3FB)Ag[*pf*]}$_{ip.}$(C_1)**	–5420.43975	933.31	1.57732	–14230896.35	–14230426.07
**2FB (C_{2v})**	–430.25471	230.60	0.32344	–1129496.89	–1129400.45
**$[NO(2FB)_1]^+$ (C_s)**	–559.72586	257.04	0.40190	–1469420.27	–1469300.45
**$[NO(2FB)_2]^+$ (C_1)**	–990.00325	496.48	0.57085	–2598924.28	–2598754.08
**$[Ag(2FB)_1]^+$ (C_s)**	–576.93780	238.62	0.39524	–1514626.66	–1514508.82
**$[Ag(2FB)_2]^+$ (C_2)**	–1007.23920	477.71	0.59418	–2644202.99	–2644025.84
**$[Ag(2FB)_3]^+$ (C_1)**	–1437.51536	716.64	0.76772	–3773705.63	–3773476.73
**{(2FB)Ag[*pf*]}$_{ip.}$(C_1)**	–5321.27182	951.91	1.55581	–13970505.99	–13970042.12
**1FB (C_{2v})**	–331.08349	249.34	0.30411	–869098.39	–869007.72
**$[NO(1FB)_1]^+$ (C_s)**	–460.56172	275.99	0.37699	–1209038.49	–1208926.09
**$[NO(1FB)_2]^+$ (C_1)**	–791.66770	533.84	0.53387	–2078146.01	–2077986.84

Supplementary Table 25, continued.

Structure (Symmetry)	E_{SCF} (DLPNO-CCSD(T)/CBS))$ / H	E_{vrt} (BP86) / kJ mol^{-1}	S^0(BP86) / kJ mol^{-1}	$G^0_{(g)}$ / kJ mol^{-1}	$H^0_{(g)}$ / kJ mol^{-1}
$[Ag(1FB)_1]^+$ (C_1) (Isomer 1.2)	−477.77063	257.35	0.37622	−1254238.90	−1254126.73
$[Ag(1FB)_1]^+$ (C_s) (Isomer 1.1)	−477.77203	257.52	0.37495	−1254242.02	−1254130.23
$[Ag(1FB)_2]^+$ (C_1) (Isomer 2.2)	−808.90408	515.14	0.57022	−2123429.65	−2123259.64
$[Ag(1FB)_2]^+$ (C_1) (Isomer 2.1)	−808.90637	515.24	0.55316	−2123430.46	−2123265.54
$[Ag(1FB)_3]^+$ (C_1)	−1140.01265	772.40	0.74174	−2992548.91	−2992327.76
{(1FB)Ag[*pf*]}$_{ip.}$(C_1)	−5222.10293	970.77	1.5303	−13710111.64	−13709655.38
DCM (C_{2v})	−958.78631	84.05	0.27153	−2517287.41	−2517206.46
$[NO(DCM)_1]^+$ (C_s)	−1088.24386	109.80	0.34587	−2857174.55	−2857071.43
$[NO(DCM)_2]^+$ (C_1)	−2047.04890	201.11	0.49395	−5374469.55	−5374322.28
$[Ag(DCM)_1]^+$ (C_{2v})	−1105.46782	91.60	0.34501	−2902413.99	−2902311.12
$[Ag(DCM)_2]^+$ (C_1)	−2064.29349	183.29	0.53252	−5419774.52	−5419615.75
$[Ag(DCM)_3]^+$ (C_3)	−3023.10120	274.87	0.67358	−7937074.16	−7936873.33
$[Ag(DCM)_4]^+$ (C_1)	−3981.90499	366.60	0.76518	−10454348.63	−10454120.49
DCE (C_2)	−998.04978	160.40	0.30160	−2620306.24	−2620216.32
$[NO(DCE)_1]^+$ (C_1)	−1127.51629	185.68	0.37696	−2960217.70	−2960105.31
$[NO(DCE)_2]^+$ (C_1)	−2125.58500	353.72	0.53076	−5580524.41	−5580366.16
$[Ag(DCE)_1]^+$ (C_2)	−1144.74632	168.16	0.35765	−3005466.89	−3005360.26
$[Ag(DCE)_2]^+$ (C_2)	−2142.84334	336.64	0.53326	−5625854.00	−5625695.01
$[Ag(DCE)_3]^+$ (C_1)	−3140.91560	504.69	0.74677	−8246187.82	−8245965.17
AN (C_{3v})	−132.58538	124.92	0.24343	−348048.03	−347975.45
$[NO(AN)_1]^+$ (C_1)	−262.05347	150.31	0.36485	−687977.24	−687868.46
$[NO(AN)_2]^+$ (C_1)	−394.66980	283.31	0.51565	−1036073.31	−1035919.57
$[Ag(AN)_1]^+$ (C_{3v})	−279.28951	132.87	0.31091	−733231.83	−733139.13
$[Ag(AN)_2]^+$ (D_{3d})	−411.94438	266.56	0.42475	−1081417.36	−1081290.72
$[Ag(AN)_3]^+$ (C_1)	−544.55416	398.63	0.66544	−1429523.98	−1429325.58
$[Ag(AN)_4]^+$ (C_1)	−677.15893	531.03	0.79621	−1777584.31	−1777346.92
DMF (C_s)	−248.22240	276.40	0.31903	−651524.03	−651428.91
$[NO(DMF)_1]^+$ (C_1)	−377.72156	304.76	0.38900	−991516.51	−991400.53
$[NO(DMF)_2]^+$ (C_1)	−625.95820	588.45	0.56036	−1643029.08	−1642862.01
$[Ag(DMF)_1]^+$ (C_s)	−394.93262	287.01	0.38761	−1036721.46	−1036605.89
$[Ag(DMF)_2]^+$ (C_2)	−643.22217	574.15	0.56134	−1688370.23	−1688202.87
$[Ag(DMF)_3]^+$ (C_1)	−891.47012	858.86	0.79267	−2339929.36	−2339693.02
$[Ag(DMF)_4]^+$ (C_1)	−1139.71387	1143.06	0.98485	−2991466.30	−2991172.67
THF (C_2)	−232.16691	310.19	0.29962	−609330.76	−609241.43

Supplementary Table 25, continued.

Structure (Symmetry)	E_{SCF} (DLPNO-CCSD(T)/CBS))$ / H	E_{vrt} (BP86) / kJ mol^{-1}	S^0(BP86) / kJ mol^{-1}	$G^0_{(g)}$ / kJ mol^{-1}	$H^0_{(g)}$ / kJ mol^{-1}
**$[Ag(THF)_1]^+$ (C_1)**	−378.86280	319.65	0.36833	−994491.79	−994381.97
**$[Ag(THF)_2]^+$ (C_2)**	−611.08910	640.29	0.54045	−1603932.50	−1603771.36
**$[Ag(THF)_3]^+$ (C_1)**	−843.27936	958.50	0.73132	−2213286.60	−2213068.56
**$[Ag(THF)_4]^+$ (C_1)**	−1075.46785	1276.92	0.89394	−2822627.44	−2822360.91

$: The calculation of E_{SCF} (DLPNO-CCSD(T)/CBS) will be explained in the section 4.2.1 below;§: Note, for Ag^+, E_{vrt} is equal to $\frac{3}{2}RT$ and the standard entropy has to be calculated with the Sackur-Tetrode equation.[71]

Supplementary Table 26: Summary of $\Delta G_{(solv)}$, $G°_{(solv)}$ and $H°_{(solv)}$ for structures used for the determination of the energetically lowest state of Ag^+ in the corresponding solvent.

Solvent	Structure	$\Delta G_{(solv)}$ / kJ mol^{-1}§	$G°_{(solv)}$ / kJ mol^{-1}	$H°_{(solv)}$ / kJ mol^{-1}$
**5FB**	Ag^+	−192.02	−385220.35	−385188.16
	$[Ag(5FB)_1]^+$	−170.32	−2295920.02	−2295808.76
	$[Ag(5FB)_2]^+$	−164.80	−4206629.34	−4206447.49
	$[Ag(5FB)_3]^+$	−169.65	−6117301.90	−6117061.87
	$\{Ag[pf]\}_{ip.}$	−49.50	−12841044.19	−12840659.92
	$\{(5FB)Ag[pf]\}_{ip.}$	−59.78	−14751714.27	−14751277.92
	$[pf]^-$	−143.97	−12455707.09	−12455331.40
	5FB	−18.05	−1910690.37	−1910589.08
**4FB**	Ag^+	−207.65	−385235.98	−385204.64
	$[Ag(4FB)_1]^+$	−186.48	−2035566.40	−2035460.05
	$[Ag(4FB)_2]^+$	−178.60	−3685895.17	−3685726.83
	$[Ag(4FB)_3]^+$	−186.55	−5336191.96	−5335965.94
	$\{Ag[pf]\}_{ip.}$	−48.57	−12841043.25	−12840658.23
	$\{(4FB)Ag[pf]\}_{ip.}$	−60.11	−14491338.90	−14490908.00
	$[pf]^-$	−145.91	−12455709.03	−12455332.00
	4FB	−18.75	−1650302.70	−1650207.32
**3FB**	Ag^+	−216.52	−385244.84	−385214.24
	$[Ag(3FB)_1]^+$	−191.64	−1775199.20	−1775097.77
	$[Ag(3FB)_2]^+$	−183.64	−3165153.61	−3164993.42
	$[Ag(3FB)_3]^+$	−187.36	−4555054.24	−4554842.10
	$\{Ag[pf]\}_{ip.}$	−47.32	−12841042.00	−12840656.05
	$\{(3FB)Ag[pf]\}_{ip.}$	−58.79	−14230955.14	−14230524.71
	$[pf]^-$	−144.53	−12455707.65	−12455329.24
	3FB	−19.07	−1389911.68	−1389822.10
**2FB**	Ag^+	−221.83	−385250.15	−385220.67
	$[Ag(2FB)_1]^+$	−193.85	−1514820.50	−1514727.34
	$[Ag(2FB)_2]^+$	−189.64	−2644392.63	−2644247.05
	$[Ag(2FB)_3]^+$	−187.72	−3773893.35	−3773700.61
	$\{Ag[pf]\}_{ip.}$	−45.68	−12841040.37	−12840653.19
	$\{(2FB)Ag[pf]\}_{ip.}$	−58.65	−13970564.64	−13970139.73
	$[pf]^-$	−141.48	−12455704.60	−12455324.54
	2FB	−19.58	−1129516.46	−1129432.91
**1FB**	Ag^+	−222.37	−385250.69	−385222.22

Supplementary Table 26, continued.

Solvent	Structure	$\Delta G_{(solv)}$ / kJ mol$^{-1§}$	$G°_{(solv)}$ / kJ mol^{-1}	$H°_{(solv)}$ / kJ mol$^{-1\$}$
**1FB**	Ag^+	–222.37	–385250.69	–385222.22
	$[Ag(1FB)_2]^+$ (Isomer 2.2)	–187.90	–2123617.55	–2123476.92
	$[Ag(1FB)_3]^+$	–183.78	–2992732.70	–2992548.13
	{Ag[*pf*]}*ip.*	–43.99	–12841038.67	–12840649.99
	{(1FB)Ag[*pf*]}*ip.*	–58.13	–13710169.77	–13709751.39
	[*pf*]⁻	–137.90	–12455701.02	–12455319.39
	1FB	–19.57	–869117.96	–869040.35
**DCM**	Ag^+	–229.99	–385258.31	–385229.99
	$[Ag(DCM)_1]^+$	–205.19	–2902619.17	–2902542.24
	$[Ag(DCM)_2]^+$	–194.98	–5419969.49	–5419843.06
	$[Ag(DCM)_3]^+$	–185.19	–7937259.36	–7937093.76
	$[Ag(DCM)_4]^+$	–190.37	–10454539.00	–10454352.90
	DCM	–15.33	–2517302.74	–2517233.32
**DCE**	Ag^+	–250.40	–385278.72	–385229.99
	$[Ag(DCE)_1]^+$	–211.77	–3005678.66	–3005600.64
	$[Ag(DCE)_2]^+$	–196.82	–5626050.82	–5625928.24
	$[Ag(DCE)_3]^+$	–193.14	–8246380.95	–8246201.77
	DCE	–22.09	–2620328.33	–2620253.66
**AN**	Ag^+	–294.16	–385322.48	–385301.99
	$[Ag(AN)_1]^+$	–222.08	–733453.90	–733384.18
	$[Ag(AN)_2]^+$	–183.09	–1081600.45	–1081494.69
	$[Ag(AN)_3]^+$	–169.71	–1429693.69	–1429522.25
	$[Ag(AN)_4]^+$	–166.58	–1777750.88	–1777547.73
	AN	–21.97	–348070.00	–348008.39
**DMF**	Ag^+	–300.45	–385328.77	–385316.55
	$[Ag(DMF)_1]^+$	–214.03	–1036935.49	–1036847.76
	$[Ag(DMF)_2]^+$	–178.90	–1688549.13	–1688412.13
	$[Ag(DMF)_3]^+$	–172.95	–2340102.31	–2339905.46
	$[Ag(DMF)_4]^+$	–177.01	–2991643.31	–2991398.68
	DMF	–27.08	–651551.10	–651470.99
**THF**	Ag^+	–300.04	–385328.36	–385322.34
	$[Ag(THF)_1]^+$	–229.88	–994721.67	–994641.76
	$[Ag(THF)_2]^+$	–183.93	–1604116.43	–1603981.52
	$[Ag(THF)_3]^+$	–177.10	–2213463.70	–2213275.94
	$[Ag(THF)_4]^+$	–183.63	–2822811.07	–2822581.90
	THF	–17.11	–609347.87	–609270.36

§: Values given at 298.15 K; $: The calculation of $H°_{(solv)}$ will be explained in the section 4.2.2 below.

Supplementary Table 27: Summary of $\Delta G_{(solv)}$ and $G°_{(solv)}$ for structures used for the determination of the energetically lowest state of NO^+ in the corresponding solvent. The solvent values, as already shown in Supplementary Table 26, have not been repeated for clarity

Solvent	Structure	$\Delta G_{(solv)}$ / kJ mol^{-1}§	$G°_{(solv)}$ / kJ mol^{-1}
	NO^+	–205.85	–340070.92
**5FB**	$[NO(5FB)_1]^+$	–165.82	–2250719.15
	$[NO(5FB)_2]^+$	–161.86	–4161368.47
	NO^+	–221.97	–340087.04
**4FB**	$[NO(4FB)_1]^+$	–179.49	–1990357.62
	$[NO(4FB)_2]^+$	–174.61	–3640627.52
	NO^+	–230.98	–340096.05
**3FB**	$[NO(3FB)_1]^+$	–184.15	–1729984.74
	$[NO(3FB)_2]^+$	–178.86	–3119870.33
	NO^+	–235.98	–340101.05
**2FB**	$[NO(2FB)_1]^+$	–188.30	–1469608.58
	$[NO(2FB)_2]^+$	–179.53	–2599103.80
	NO^+	–235.53	–340100.59
**1FB**	$[NO(1FB)_1]^+$	–186.84	–1209225.33
	$[NO(1FB)_2]^+$	–179.03	–2078325.04
	NO^+	–244.07	–340109.14
**DCM**	$[NO(DCM)_1]^+$	–204.90	–2857379.45
	$[NO(DCM)_2]^+$	–194.48	–5374664.03
	NO^+	–267.55	–340132.62
**DCE**	$[NO(DCE)_1]^+$	–214.27	–2960431.96
	$[NO(DCE)_2]^+$	–205.66	–5580730.07
	NO^+	–315.58	–340180.64
**AN**	$[NO(AN)_1]^+$	–234.30	–688211.55
	$[NO(AN)_2]^+$	–196.07	–1036269.38
	NO^+	–332.49	–340197.56
**DMF**	$[NO(DMF)_1]^+$	–217.89	–991734.40
	$[NO(DMF)_2]^+$	–209.88	–1643238.95

§: Values given at 298.15 K

4.2.1 Calculation of the DLPNO-CCSD(T)/CBS values

The complete basis set (CBS) values were determined based on the method developed by Helgaker *et al.*.[76] The procedure will be described below and is exemplary illustrated for one molecule.

For the determination of the CBS value for each structure, the respective energies of the DFT structure were refined in a series of DLPNO-CCSD(T) single point calculations[19–21] with Dunning's[22–25,28,30] basis sets cc-pVDZ, cc-pVTZ, cc-pVQZ. These values were used for a graphical plot (Supplementary Figure 97a), with the individual energies of the single point calculations (ordinate) are plotted against X^{-3} (abscissa), with X = **2** for cc-pV**D**Z, X = **3** for cc-pV**T**Z and X = **4** for cc-pV**Q**Z. The graphical plot was then used for a linear regression. The resulting value for the ordinate intercept is equal to the CBS value for the corresponding structure (Supplementary Figure 97b).

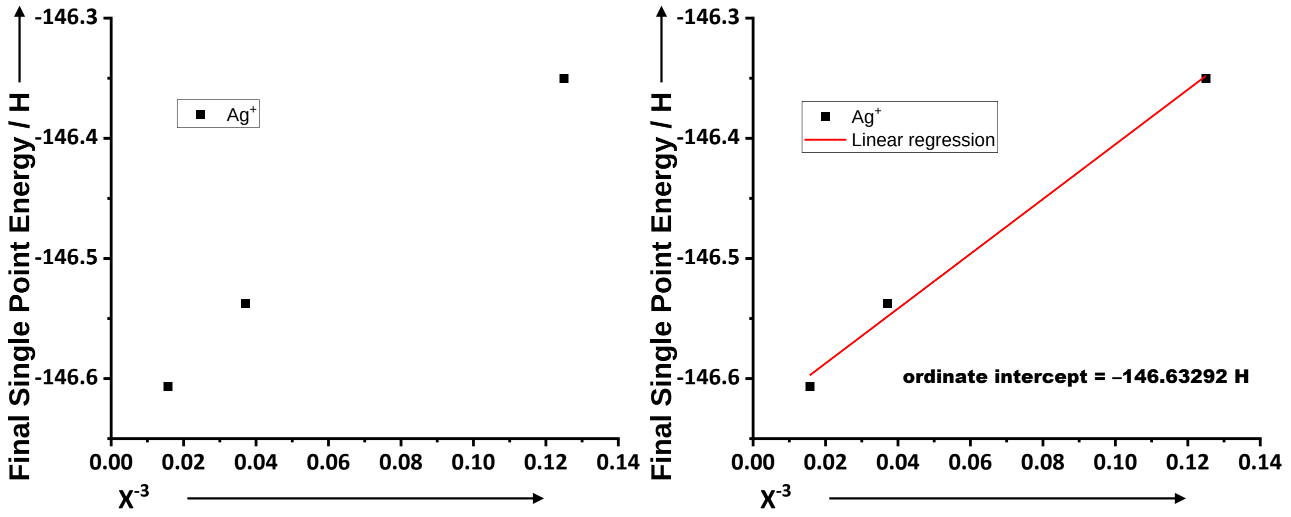

Supplementary Figure 97: Left.) Graphical plot of single point energies for Ag^+, from calculations using Dunning´s basis sets cc-pVDZ, cc-pVTZ, cc-pVQZ. **Right.)** Graphical plot of the left plot), given with a regression line and the resulting DLPNO-CCSD(T)/CBS value for Ag^+ from the ordinate intercept.

The DLPNO-CCSD(T)/CBS values, for each structure determined as described above, are summarized in section 4.2 (Supplementary Table 25) above. The energies used for the determination of the DLPNO-CCSD(T)/CBS values, which were received from the series of DLPNO-CCSD(T) single point calculations are summarized Supplementary Table 28 below.

Supplementary Table 28: Summary of the energies, which were received from the series of DLPNO ccsd(t) single point calculations using different basis sets. All values are given in Hartree H.

Structure	Final Single Point Energy (cc-pVDZ)/ H	Final Single Point Energy (cc-pVTZ)/ H	Final Single Point Energy (cc-pVQZ)/ H
NO^+	–129.27446	–129.38271	–129.41691
Ag^+	–146.35030	–146.53726	–146.60638
[*pf*]⁻	–4737.53831	–4742.05896	–4743.47325
{Ag[*pf*]}*ip.*	–4884.08838	–4888.77441	–4890.25295
5FB	–726.71394	–727.42606	–727.64832
$[NO(5FB)_1]^+$	–856.00740	–856.82963	–857.08557
$[NO(5FB)_2]^+$	–1582.73430	–1584.26939	–1584.74676
$[Ag(5FB)_1]^+$	–873.09363	–873.99424	–874.28475
$[Ag(5FB)_2]^+$	–1599.84026	–1601.45361	–1601.96532
$[Ag(5FB)_3]^+$	–2326.57904	–2328.90144	–2329.63236
{(5FB)Ag[*pf*]}*ip.*	–5610.82696	–5616.22171	–5617.91993
4FB	–627.69053	–628.30506	–628.49609
$[NO(4FB)_1]^+$	–756.98945	–757.71405	–757.93908
$[NO(4FB)_2]^+$	–1384.69521	–1386.03485	–1386.45021
$[Ag(4FB)_1]^+$	–774.07568	–774.87915	–775.13892
$[Ag(4FB)_2]^+$	–1401.80361	–1403.22278	–1403.67197
$[Ag(4FB)_3]^+$	–2029.51890	–2031.55020	–2032.18862
{(4FB)Ag[*pf*]}*ip.*	–5511.80591	–5517.10471	–5518.77221
3FB	–528.66618	–529.18299	–529.34275
$[NO(3FB)_1]^+$	–657.96928	–658.59636	–658.79026
$[NO(3FB)_2]^+$	–1186.65474	–1187.79877	–1188.15199
$[Ag(3FB)_1]^+$	–675.05765	–675.76361	–675.99242
$[Ag(3FB)_2]^+$	–1203.76569	–1204.98994	–1205.37821
$[Ag(3FB)_3]^+$	–1732.45634	–1734.19524	–1734.74148
{(3FB)Ag[*pf*]}*ip.*	–5412.78272	–5417.98374	–5419.62064
2FB	–429.64010	–430.05922	–430.18775
$[NO(2FB)_1]^+$	–558.95021	–559.47886	–559.64156
$[NO(2FB)_2]^+$	–988.61203	–989.56059	–989.85178
$[Ag(2FB)_1]^+$	–576.03767	–576.64596	–576.84382
$[Ag(2FB)_2]^+$	–1005.72313	–1006.75159	–1007.07800
$[Ag(2FB)_3]^+$	–1435.38877	–1436.83400	–1437.28731
{(2FB)Ag[*pf*]}*ip.*	–5313.75855	–5318.86214	–5320.46793
1FB	–330.61324	–330.93460	–331.03175
$[NO(1FB)_1]^+$	–459.92981	–460.36101	–460.49266
$[NO(1FB)_2]^+$	–790.56516	–791.31801	–791.54684
$[Ag(1FB)_1]^+$ (Isomer 1.2)	–477.01500	–477.52524	–477.69203
$[Ag(1FB)_1]^+$ (Isomer 1.1)	–477.01632	–477.52668	–477.69337
$[Ag(1FB)_2]^+$ (Isomer 2.2)	–807.67686	–808.50945	–808.77354
$[Ag(1FB)_2]^+$ (Isomer 2.1)	–807.67907	–808.51166	–808.77585
$[Ag(1FB)_3]^+$	–1138.31820	–1139.47016	–1139.83064
{(1FB)Ag[*pf*]}*ip.*	–5214.73328	–5219.73934	–5221.31437

Supplementary Table 28, continued.

Structure	Final Single Point Energy (cc-pVDZ)/ H	Final Single Point Energy (cc-pVTZ)/ H	Final Single Point Energy (cc-pVQZ)/ H
DCM	–958.49328	–958.69337	–958.75419
$[NO(DCM)_1]^+$	–1087.78905	–1088.09859	–1088.19475
$[NO(DCM)_2]^+$	–2046.29963	–2046.80990	–2046.96777
$[Ag(DCM)_1]^+$	–1104.89024	–1105.27770	–1105.40961
$[Ag(DCM)_2]^+$	–2063.42372	–2064.00978	–2064.20393
$[Ag(DCM)_3]^+$	–3021.94282	–3022.72498	–3022.98072
$[Ag(DCM)_4]^+$	–3980.45279	–3981.43567	–3981.75224
DCE	–997.69711	–997.93834	–998.01081
$[NO(DCE)_1]^+$	–1127.00175	–1127.35241	–1127.46040
$[NO(DCE)_2]^+$	–2124.71650	–2125.30884	–2125.49031
$[Ag(DCE)_1]^+$	–1144.10794	–1144.53737	–1144.68112
$[Ag(DCE)_2]^+$	–2141.85363	–2142.52223	–2142.74017
$[Ag(DCE)_3]^+$	–3139.57778	–3140.48359	–3140.77462
AN	–132.39875	–132.52674	–132.56451
$[NO(AN)_1]^+$	–261.70750	–261.94348	–262.01574
$[NO(AN)_2]^+$	–394.13654	–394.50105	–394.61106
$[Ag(AN)_1]^+$	–278.81769	–279.13425	–279.24193
$[Ag(AN)_2]^+$	–411.28280	–411.72957	–411.87553
$[Ag(AN)_3]^+$	–543.70952	–544.28167	–544.46497
$[Ag(AN)_4]^+$	–676.13125	–676.82877	–677.04938
DMF	–247.84883	–248.10480	–248.18080
$[NO(DMF)_1]^+$	–377.18788	–377.55257	–377.66286
$[NO(DMF)_2]^+$	–625.05562	–625.67290	–625.85855
$[Ag(DMF)_1]^+$	–394.27805	–394.71923	–394.86512
$[Ag(DMF)_2]^+$	–642.19622	–642.89136	–643.11370
$[Ag(DMF)_3]^+$	–890.07531	–891.02300	–891.32072
$[Ag(DMF)_4]^+$	–1137.95277	–1139.15131	–1139.52377
THF	–231.81332	–232.05649	–232.12688
$[Ag(THF)_1]^+$	–378.22952	–378.65681	–378.79717
$[Ag(THF)_2]^+$	–610.10523	–610.77322	–610.98408
$[Ag(THF)_3]^+$	–841.94858	–842.85474	–843.13536
$[Ag(THF)_4]^+$	–1073.79142	–1074.93519	–1075.28479

4.2.2 Calculation of $H°_{(solv)}$

For the calculation of $H°_{(solv)}$, which was only performed for Ag^+ related structures and the corresponding solvent molecules, the following equation was used.

$$H°_{(solv)} = H° + \Delta H_{(solv)} \quad \text{(S5)}$$

Therefore, the corresponding value for $\Delta H_{(solv)}$ has to be determined for each molecule for the calculation of $H°_{(solv)}$. For the determination of $\Delta H_{(solv)}$, the generally valid equation (S2) was used for the solvation reaction, resulting in the equation (S6). Compared to a general linear equation, $\Delta G_{(solv)}$ can be identified as y (ordinate), $\Delta H_{(solv)}$ as the ordinate intercept t, $\Delta S_{(solv)}$ as the slope m and the temperature T as value for the abscissa.

$$\underbrace{\Delta G_{(solv)}}_{y} = \underbrace{\Delta H_{(solv)}}_{t} - \underbrace{T}_{x} \cdot \underbrace{\Delta S_{(solv)}}_{m} \quad \text{(S6)}$$

From the equation (S6) only $\Delta G_{(solv)}$ can be directly calculated, using the calculated structures at (RI-)BP86(D3BJ)/def2-TZVPP level of theory for the COSMO-RS[31–33] model at BP86(D3)/def2-TZVPD//BP86(D3)/def-TZVP level of theory[34]. By plotting $\Delta G_{(solv)}$ against T and using linear regression, the ordinate intercept, which is equal to $\Delta H_{(solv)}$ can be determined and therefore $H°_{(solv)}$ can be calculated, as well. Therefore, for each silver related structure in each used solvent, the value $\Delta G_{(solv)}$ was calculated with COSMO-RS[31–33] at five different temperatures (294.15 K, 296.15 K, 298.15 K, 300.15 K and 302.15 K) and plotted against the temperature T. As described above, the plot was then be used for a linear regression for each structure, to yield the wanted values $\Delta H_{(solv)}$ as the ordinate intercepts of the corresponding linear regression as illustrated in Supplementary Figure 98 for 1FB as solvent.

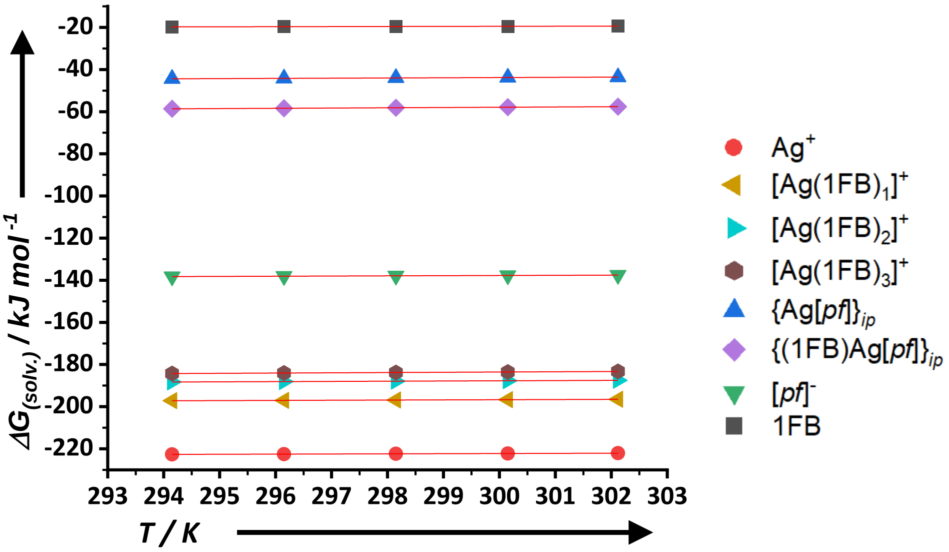

Supplementary Figure 98: Graphical plot of $\Delta G_{(solv)}$, given in kJ mol^{-1}, against the temperature T, given in Kelvin, for five different temperatures (294.15 K, 296.15 K, 298.15 K, 300.15 K and 302.15 K) for Ag^+, $[Ag(1FB)_1]^+$,$[Ag(1FB)_2]^+$, $[Ag(1FB)_3]^+$, $\{Ag[pf]\}_{ip.}$, $\{(1FB)Ag[pf]\}_{ip.}$, $[pf]^-$ and 1FB in 1FB, given with the corresponding regression line for each structure.

The results for $\Delta H_{(solv)}$ from the structures shown in Supplementary Figure 98 for 1FB as solvent are summarised together with the values for corresponding structures in the used solvents determined as described above, are given in Supplementary Table 29 together with the $\Delta G_{(solv)}$ energies used from the series of COSMO-RS[31–33] calculation. The calculated values for the standard enthalpy $H°_{(solv)}$ are given in the section 4.2 (Supplementary Table 26) above.

Supplementary Table 29: Summary of the $\underline{\Delta H_{(solv)}}$ values for each structures used in solvent, together with the corresponding $\Delta G_{(solv)}$ energies from the series of COSMO-RS[31–33] calculations at five different temperatures (294.15 K, 296.15 K, 298.15 K, 300.15 K and 302.15 K). All energies are given in kJ mol^{-1}.

Solvent	Structure	$\Delta G_{(solv)}$ / kJ mol^{-1} at 294.15 K	$\Delta G_{(solv)}$ / kJ mol^{-1} at 296.15 K	$\Delta G_{(solv)}$ / kJ mol^{-1} at 298.15 K	$\Delta G_{(solv)}$ / kJ mol^{-1} at 300.15 K	$\Delta G_{(solv)}$ / kJ mol^{-1} at 302.15 K	$\Delta H_{(solv)}$ / kJ mol^{-1}
**5FB**	Ag^+	−192.26	−192.14	−192.02	−191.91	−191.79	−209.70
	$[Ag(5FB)_1]^+$	−170.63	−170.47	−170.32	−170.17	−170.02	−192.88
	$[Ag(5FB)_2]^+$	−165.17	−164.98	−164.80	−164.61	−164.43	−192.58
	$[Ag(5FB)_3]^+$	−170.11	−169.88	−169.65	−169.42	−169.20	−204.02
	{Ag[*pf*]}*ip.*	−49.97	−49.74	−49.50	−49.27	−49.05	−84.01
	{(5FB)Ag[*pf*]}*ip.*	−60.33	−60.05	−59.78	−59.51	−59.25	−100.06
	[*pf*]$^-$	−144.40	−144.19	−143.97	−143.76	−143.55	−175.98
	5FB	−18.22	−18.13	−18.05	−17.96	−17.88	−30.51
**4FB**	Ag^+	−207.90	−207.78	−207.65	−207.53	−207.41	−226.18
	$[Ag(4FB)_1]^+$	−186.80	−186.64	−186.48	−186.33	−186.17	−209.95
	$[Ag(4FB)_2]^+$	−178.99	−178.79	−178.60	−178.41	−178.22	−207.42
	$[Ag(4FB)_3]^+$	−187.03	−186.79	−186.55	−186.32	−186.08	−222.00
	{Ag[*pf*]}*ip.*	−49.02	−48.79	−48.57	−48.34	−48.12	−82.32
	{(4FB)Ag[*pf*]}*ip.*	−60.65	−60.38	−60.11	−59.84	−59.58	−100.33
	[*pf*]$^-$	−146.32	−146.11	−145.91	−145.70	−145.50	−176.58
	4FB	−18.91	−18.83	−18.75	−18.66	−18.58	−31.31
**3FB**	Ag^+	−216.78	−216.65	−216.52	−216.39	−216.26	−235.78
	$[Ag(3FB)_1]^+$	−191.96	−191.80	−191.64	−191.48	−191.32	−215.54
	$[Ag(3FB)_2]^+$	−183.45	−183.25	−183.64	−182.86	−182.66	−212.54
	$[Ag(3FB)_3]^+$	−187.84	−187.60	−187.36	−187.13	−186.90	−222.62
	{Ag[*pf*]}*ip.*	−47.76	−47.54	−47.32	−47.10	−46.88	−80.14
	{(3FB)Ag[*pf*]}*ip.*	−59.33	−59.06	−58.79	−58.53	−58.26	−98.64
	[*pf*]$^-$	−144.92	−144.72	−144.53	−144.33	−144.14	−173.82
	3FB	−19.24	−19.16	−19.07	−18.99	−18.90	−31.68
**2FB**	Ag^+	−222.11	−221.97	−221.83	−221.70	−221.56	−242.21
	$[Ag(2FB)_1]^+$	−194.18	−194.01	−193.85	−193.68	−193.52	−218.52
	$[Ag(2FB)_2]^+$	−190.06	−189.85	−189.64	−189.43	−189.22	−221.21
	$[Ag(2FB)_3]^+$	−188.21	−187.96	−187.72	−187.48	−187.24	−223.87
	{Ag[*pf*]}*ip.*	−46.11	−45.90	−45.68	−45.47	−45.26	−77.28

Supplementary Table 29, continued.

Solvent	Structure	$\Delta G°_{(solv)}$ / kJ mol^{-1} at 294.15 K	$\Delta G°_{(solv)}$ / kJ mol^{-1} at 296.15 K	$\Delta G°_{(solv)}$ / kJ mol^{-1} at 298.15 K	$\Delta G°_{(solv)}$ / kJ mol^{-1} at 300.15 K	$\Delta G°_{(solv)}$ / kJ mol^{-1} at 302.15 K	$\Delta H°_{(solv)}$ / kJ mol^{-1}
**2FB**	{(2FB)Ag[*pf*]}$_{ip.}$	−59.18	−58.91	−58.65	−58.39	−58.14	−97.61
	[*pf*]$^{-}$	−141.85	−141.67	−141.48	−141.30	−141.11	−169.12
	2FB	−19.75	−19.67	−19.58	−19.49	−19.41	−32.46
**1FB**	Ag^{+}	−222.66	−222.51	−222.37	−222.23	−222.08	−243.76
	[Ag(1FB)$_1$]$^+$	−197.18	−197.01	−196.85	−196.69	−196.52	−221.42
	(Isomer 1.2)) [Ag(1FB)$_2$]$^+$	−188.29	−188.10	−187.90	−187.70	−187.51	−217.28
	(Isomer 2.2) [Ag(1FB)$_3$]$^+$	−184.28	−184.03	−183.78	−183.54	−183.30	−220.36
	{Ag[*pf*]}$_{ip.}$	−44.39	−44.19	−43.99	−43.79	−43.59	−74.08
	{(1FB)Ag[*pf*]}$_{ip.}$	−58.64	−58.38	−58.13	−57.87	−57.62	−96.01
	[*pf*]$^{-}$	−138.25	−138.08	−137.90	−137.73	−137.56	−163.97
	1FB	−19.75	−19.66	−19.57	−19.48	−19.40	−32.63
**DCM**	Ag^{+}	−230.28	−230.13	−229.99	−229.85	−229.70	−251.53
	[Ag(DCM)$_1$]$^+$	−205.53	−205.36	−205.19	−205.01	−204.84	−231.11
	[Ag(DCM)$_2$]$^+$	−195.41	−195.20	−194.98	−194.76	−194.55	−227.31
	[Ag(DCM)$_3$]$^+$	−185.67	−185.43	−185.19	−184.96	−184.73	−220.42
	[Ag(DCM)$_4$]$^+$	−190.94	−190.66	−190.37	−190.09	−189.81	−232.41
	DCM	−15.48	−15.41	−15.33	−15.25	−15.18	−26.86
**DCE**	Ag^{+}	−250.73	−250.56	−250.40	−250.24	−250.07	−274.78
	[Ag(DCE)$_1$]$^+$	−212.15	−211.96	−211.77	−211.58	−211.39	−240.38
	[Ag(DCE)$_2$]$^+$	−197.31	−197.06	−196.82	−196.58	−196.33	−233.23
	[Ag(DCE)$_3$]$^+$	−193.72	−193.43	−193.14	−192.85	−192.56	−236.61
	DCE	−22.30	−22.19	−22.09	−21.99	−21.89	−37.34
**AN**	Ag^{+}	−294.55	−294.36	−294.16	−293.96	−293.77	−323.53
	[Ag(AN)$_1$]$^+$	−222.39	−222.23	−222.08	−221.92	−221.77	−245.05
	[Ag(AN)$_2$]$^+$	−183.37	−183.23	−183.09	−182.95	−182.81	−203.97
	[Ag(AN)$_3$]$^+$	−170.07	−169.89	−169.71	−169.53	−169.35	−196.68
	[Ag(AN)$_4$]$^+$	−167.04	−166.81	−166.58	−166.35	−166.12	−200.82
	AN	−22.12	−22.04	−21.97	−21.90	−21.82	−32.94

Supplementary Table 29, continued.

Solvent	Structure	Δ$G°_{(solv)}$ / kJ mol^{-1} at 294.15 K	Δ$G°_{(solv)}$ / kJ mol^{-1} at 296.15 K	Δ$G°_{(solv)}$ / kJ mol^{-1} at 298.15 K	Δ$G°_{(solv)}$ / kJ mol^{-1} at 300.15 K	Δ$G°_{(solv)}$ / kJ mol^{-1} at 302.15 K	Δ$H°_{(solv)}$ / kJ mol^{-1}
**DMF**	Ag^+	−300.96	−300.71	−300.45	−300.20	−299.95	<u>−338.09</u>
	$[Ag(DMF)_1]^+$	−214.41	−214.22	−214.03	−213.85	−213.66	<u>−241.86</u>
	$[Ag(DMF)_2]^+$	−179.31	−179.10	−178.90	−178.70	−178.50	<u>−209.27</u>
	$[Ag(DMF)_3]^+$	−173.48	−173.22	−172.95	−172.69	−172.43	<u>−212.43</u>
	$[Ag(DMF)_4]^+$	−177.67	−177.34	−177.01	−176.69	−176.36	<u>−226.01</u>
	DMF	−27.28	−27.18	−27.08	−26.98	−26.88	<u>−42.09</u>
**THF**	Ag^+	−300.63	−300.34	−300.04	−299.75	−299.46	<u>−343.88</u>
	$[Ag(THF)_1]^+$	−230.28	−230.08	−229.88	−229.68	−229.48	<u>−259.78</u>
	$[Ag(THF)_2]^+$	−184.28	−184.10	−183.93	−183.75	−183.58	<u>−210.16</u>
	$[Ag(THF)_3]^+$	−177.51	−177.30	−177.10	−176.90	−176.70	<u>−207.38</u>
	$[Ag(THF)_4]^+$	−184.13	−183.88	−183.63	−183.38	−183.13	<u>−220.99</u>
	THF	−17.27	−17.19	−17.11	−17.04	−16.96	<u>−28.94</u>

4.3 Silver ion solvent S-complexation and [*pf*]$^-$ ion-pairing enthalpies and Gibbs energies in the gas phase (g) and in S.

Calculated gaseous quantities $\Delta_r H°$(g) / $\Delta_r G°$(g) as well as $\Delta_r H°$(solv) / $\Delta_r G°$(solv) in solution in S at standard conditions are collected in Supplementary Table 30. The solvent complexation energies of Ag^+, collected left of the grey Ag^+ column in Supplementary Table 30, greatly decrease with increasing fluorination of xFB, both in S as well as in the gas phase. Typically two or three xFB molecules may be taken up in the solvates $[Ag(S)_n]^+$. Yet, especially for the highly fluorinated xFB molecules with x = 4, 5, the desolvation Gibbs energy $\Delta_r G°$(xFB) of 9 to 28 kJ mol^{-1} is very low and, hence, the solvated Ag^+ ion is very reactive in these solvents. This agrees with the very high $E_{1/2}(Ag^+, S)$-values of 1.35 and 1.38 V vs. Fc^+/Fc measured in 4FB and 5FB.

Note: In order to use the most energetically favourable structure in the gas phase and in the solvated phase, two isomers were used for each of the following two structures: $[Ag(1FB)_1]^+$ and $[Ag(1FB)_2]^+$. Isomers 1.1 ($[Ag(1FB)_1]^+$) and 2.1 ($[Ag(1FB)_2]^+$) for the gas phase and isomers 1.2 ($[Ag(1FB)_1]^+$) and 2.2 ($[Ag(1FB)_2]^+$) for the solvated phase.

Supplementary Table 30: Successive silver ion solvent S-complexation and $[pf]^-$ ion-pairing enthalpies and Gibbs energies in the gas phase (g) and in the indicated solvents S. The gas phase values are accurate ccsd(t)/CBS values derived from single point calculations on (RI-)BP86(D3BJ)/def2-TZVPP DFT structures, contributions of solvation enthalpies and free energies in S were calculated with the COSMO-RS model at the BP86(D3)/def2-TZVPD//BP86(D3)/def-TZVP level of theory. All values are given in kJ mol^{-1}. The most stable solvates and ion-pairs (*ip*) expected to be present in solution in S are marked in **bold**.

Reaction → ↓ Magnitude (g, S)	$\{(S)Ag[pf]\}_{ip.} \xleftarrow{+S}$	$\{Ag[pf]\}_{ip.} \xleftarrow{+[pf]^-}$	**Ag^+**	$\xrightarrow{+S} [Ag(S)_1]^+$	$\xrightarrow{+S} [Ag(S)_2]^+$	$\xrightarrow{+S} [Ag(S)_3]^+$	$\xrightarrow{+S} [Ag(S)_4]^+$
$\Delta_r H°(g)/\Delta_r G°(g)$: 5FB	–43.4 / +12.5	–442.0 / –403.2		–78.8 / –49.1	–80.5 / –42.5	–44.4 / +4.6	-
$\Delta_r H°(5FB)/\Delta_r G°(5FB)$	–28.9 / +20.3	–140.4 / **–116.7**		–31.5 / –9.3	–49.6 / **–18.9**	–25.3 / +17.8	-
$\Delta_r H°(g)/\Delta_r G°(g)$: 4FB	–55.8 / –0.2	–442.0 / –403.2		–95.6 / –67.6	–93.3 / –52.7	–48.5 / –4.9	-
$\Delta_r H°(4FB)/\Delta_r G°(4FB)$	–42.5 / +7.1	–121.6 / **–98.2**		–48.1 / –27.7	–59.5 / **–26.1**	–31.8 / +5.9	-
$\Delta_r H°(g)/\Delta_r G°(g)$: 3FB	–59.7 / –9.1	–442.0 / –403.2		–113.3 / –86.6	–108.2 / –69.8	–48.2 / –4.3	-
$\Delta_r H°(3FB)/\Delta_r G°(3FB)$	–46.6 / **–1.5**	–112.6 / –89.5		–61.4/ –42.7	–73.5 / **–42.7**	–26.6/ +11.1	-
$\Delta_r H°(g)/\Delta_r G°(g)$: 2FB	–65.8/ –14.4	–442.0 / –403.2		–129.9 / –101.5	–116.6 / –79.4	–50.4 / –5.8	-
$\Delta_r H°(2FB)/\Delta_r G°(2FB)$	–53.6 / **–7.8**	–108.0 / –85.6		–73.8 / –53.9	–86.8 / **–55.7**	–20.6 / +15.7	-
$\Delta_r H°(g)/\Delta_r G°(g)$: 1FB§	–71.8 / –18.6	–442.0 / –403.2		–144.0 / –115.3	–127.6 / –90.1	–54.5 / –20.1	-
$\Delta_r H°(1FB)/\Delta_r G°(1FB)$$	–61.1 / **–13.1**	–108.4 / –87.0		–85.6 / –67.1	–88.4 / **–63.8**	–30.9/ +2.8	-
$\Delta_r H°(g)/\Delta_r G°(g)$: DCM	-	-		–126.2 / –98.3	–98.2 / –73.1	–51.1 / –12.2	–40.7 / +12.9
$\Delta_r H°(DCM)/\Delta_r G°(DCM)$	-	-		–78.9 / –58.1	–67.5 / **–47.6**	–17.4 / +12.9	–25.8 / +23.1
$\Delta_r H°(g)/\Delta_r G°(g)$: DCE	-	-		–165.5 / –132.3	–118.4 / –80.9	–53.8 / –27.6	-
$\Delta_r H°(DCE)/\Delta_r G°(DCE)$	-	-		–93.7 / –71.6	–73.9 / –43.8	–19.9 / **–1.8**	-
$\Delta_r H°(g)/\Delta_r G°(g)$: AN	-	-		–185.2 / –155.5	–176.1 / –137.5	–59.4 / –58.6	–45.9 / –12.3
$\Delta_r H°(AN)/\Delta_r G°(AN)$	-	-		–73.8 / –61.4	–102.1/ –76.5	–19.2 / **–23.2**	–17.1 / +12.8
$\Delta_r H°(g)/\Delta_r G°(g)$: DMF	-	-		–198.5 / –169.1	–168.1 / –124.7	–61.3 / –35.1	–50.7 / –12.9
$\Delta_r H°(DMF)/\Delta_r G°(DMF)$	-	-		–60.2 / –55.6	–93.4 / –62.5	–22.3 / **–2.1**	–22.2 / +10.1
$\Delta_r H°(g)/\Delta_r G°(g)$: THF	-	-		–162.1 / –132.7	–148.0 / –110.0	–55.8 / –23.3	–50.9 / –10.1
$\Delta_r H°(S)/\Delta_r G°(S)$ (THF)	-	-		–49.1 / –45.4	–69.4 / **–46.9**	–24.1/ +0.6	–35.6 / +0.5

§: For $[Ag(1FB)_1]^+$ Isomer 1.1 and for $[Ag(1FB)_2]^+$ Isomer 2.1 were used in the gas phase. $: For $[Ag(1FB)_1]^+$ Isomer 1.2 and for $[Ag(1FB)_2]^+$ Isomer 2.2 were used in the solvated phase.

In addition, for the three most fluorinated xFB solvents, the calculations predict that ion-pairs are more favorable than dissociated ions (entries left to the grey Ag^+ column in Supplementary Table 30). Pleasingly, the complexes or ion-pairs, calculated to be most favorable in solution, comply well with those found with scXRD analyses (Figure 3, main article). In addition, the complexation energies of Ag^+ with 2FB and CH_2Cl_2 are comparable as are their $E_{1/2}$-values of 0.99 and 0.88 V vs. Fc^+/Fc. Since the interaction energies calculated for 1FB are more favorable than for 2FB and CH_2Cl_2, $E_{1/2}(Ag^+, 1FB)$ is reduced to 0.74 V. The difference between the $E_{1/2}(Ag^+, S)$-values in 2FB and 1FB (0.99–0.74 = 0.25 V), the only apparently fully dissociated xFB solvents, also agrees with the difference of the sum of their 1st and 2nd complexation Gibbs energies in solution of 21.3 kJ mol^{-1} [= (–53.9 + –55.7)–(–67.5 + –63.4) kJ mol^{-1}]; this corresponds to 0.22 V. For x > 2, the values are influenced by ion-pairing and cannot be used for such quantitative evaluations. By contrast, the complexation energies of AN and DMF, which induce the lowest Ag^+ potentials around 0 V vs. Fc^+/Fc, are also by far the most favorable of all the nine solvents assessed within Supplementary Table 30. This also complies with experiment. Hence, we do suggest that the experimental $E_{1/2}(Ag^+, S)$-values collected in Table 2 (main article) reflect the true reactivity of the Ag^+ deelectronator ions in S.

4.4 NO^+ ion solvent S-complexation enthalpies and Gibbs energies in the gas phase (g) and in S.

All calculated NO^+ interaction energies are collected in Supplementary Table 31. Interestingly, while the formation of Wheland-complexes is favored for all the aromatic xFB molecules in the gas phase and in part even the uptake of a second xFB molecules is viable (upper line of each entry for xFB), the situation is very different in xFB-solution: Apparently only 1FB – with the lowest IE of all xFB molecules (main article, Table 2) – is electron-rich enough to slightly favor the complex $[NO(1FB)]^+$ also in 1FB-solution. By contrast, in all other cases, the calculations suggest the presence of uncomplexed NO^+ in xFB (x = 2-5) solution, although this is on the edge for 2FB which complies with the scXRD structure [NO(2FB)][*pf*] (Figure3A). This strive towards the formation of free NO^+ results from the rather high (calculated) solvation energies for the isolated NO^+ ion vs. the complexed $[NO(xFB)]^+$ systems. Overall, from 1FB to 5FB the interaction energies gradually get weaker, concomitant to the increase of the solvent *IE*s. This aligns with the potentials $E_{1/2}$ collected in Table 2 that spread in xFB solution over 1.52 (4FB) – 1.11 (1FB) = 0.41 V. Also the difference of $\Delta_r G°$(xFB) for the first arene complexation between 4FB and 1FB amounts to 32.1–(–6.8) = 38.9 kJ mol^{-1} or 0.40 V. Hence, the drastic increase of the NO^+ potentials results from the inferior interaction of the cation with the higher fluorinated xFB solvents. In addition, this weak tendency to form Wheland-complexes $[NO(xFB)]^+$ aligns with the *IE*s of the xFB solvents of 9.20-9.63 eV in Table 2 (main article): They are close to (1FB) or even higher (xFB, x = 2-5) than that of NO of 9.26 eV.

Supplementary Table 31: NO^+ ion solvent S-complexation enthalpies and Gibbs energies in the gas phase (g) and in the indicated solvents S. The gas phase values are accurate ccsd(t)/CBS values derived from single point calculations on (RI-)BP86(D3BJ)/def2-TZVPP DFT structures, contributions of solvation enthalpies and free energies in S were calculated with the COSMO-RS model at the (RI-)BP86(D3BJ)/def2-TZVPP level. All values are given in kJ mol^{-1}. The most stable solvates are marked in **bold**.

Reaction → ↓ Magnitude (g, S)	$NO^+ + S \rightarrow [NO(S)_1]^+$	$+S \rightarrow [NO(S)_2]^+$
$\Delta_r H°(g)/\Delta_r G°(g)$: 5FB	−51.7 / −15.9	−29.1/ +19.0
$\Delta_r G°$(5FB)	+36.9	+25.1
$\Delta_r H°(g)/\Delta_r G°(g)$: 4FB	−66.3 / −29.1	−34.7 / +9.2
$\Delta_r G°$(4FB)	+32.1	+32.8
$\Delta_r H°(g)/\Delta_r G°(g)$: 3FB	−78.2 / −42.9	−44.2 / +1.7
$\Delta_r G°$(3FB)	+23.0	+26.1
$\Delta_r H°(g)/\Delta_r G°(g)$: 2FB	−94.1 / −58.3	−53.2 / −7.1
$\Delta_r G°$(2FB)	+8.9	+21.2
$\Delta_r H°(g)/\Delta_r G°(g)$: 1FB	−112.5 / −75.0	−53.0 / −9.1
$\Delta_r G°$(1FB)	**−6.8**	+18.3
$\Delta_r H°(g)/\Delta_r G°(g)$: DCM	−59.1 / −22.1	−44.4 / −7.6
$\Delta_r G°$(DCM)	+32.4	+18.2
$\Delta_r H°(g)/\Delta_r G°(g)$: DCE	−83.1 / −46.4	−44.5 / −0.5
$\Delta_r G°$(DCE)	+29.0	+30.2
$\Delta_r H°(g)/\Delta_r G°(g)$: AN	−87.1 / −64.1	−75.7 / −48.0
$\Delta_r G°$(AN)	+39.1	+12.2
$\Delta_r H°(g)/\Delta_r G°(g)$: DMF	−165.7 / −127.4	−32.6 / +11.5
$\Delta_r G°$(DMF)	+14.3	+46.5

4.5 Comparison of the Half-Wave Potentials of the Innocent Deelectronators with their Ionization Energies

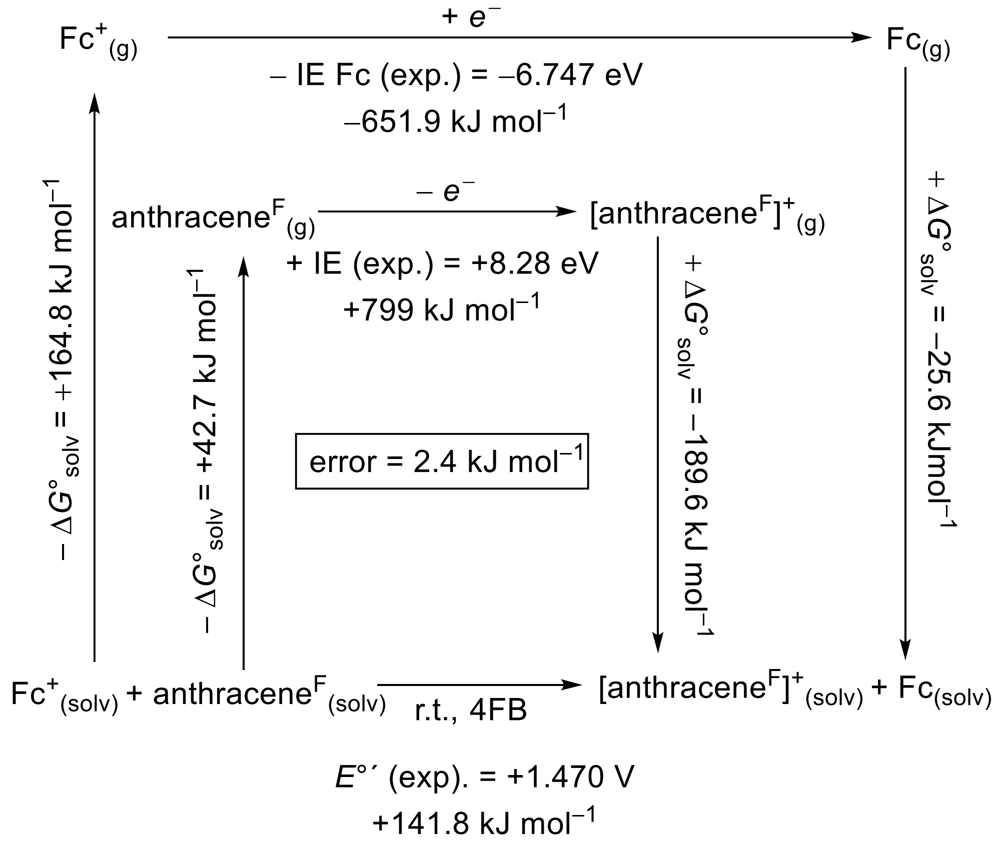

Supplementary Figure 99: Born-Haber-Fajans cycle of the reaction of anthracene^F with Fc^+ in 4FB using the experimental ionization energies of ferrocene[77] and anthracene^F, cyclovoltammetric half-wave potential in 4FB and calculated solvation enthalpies from COSMO-RS.

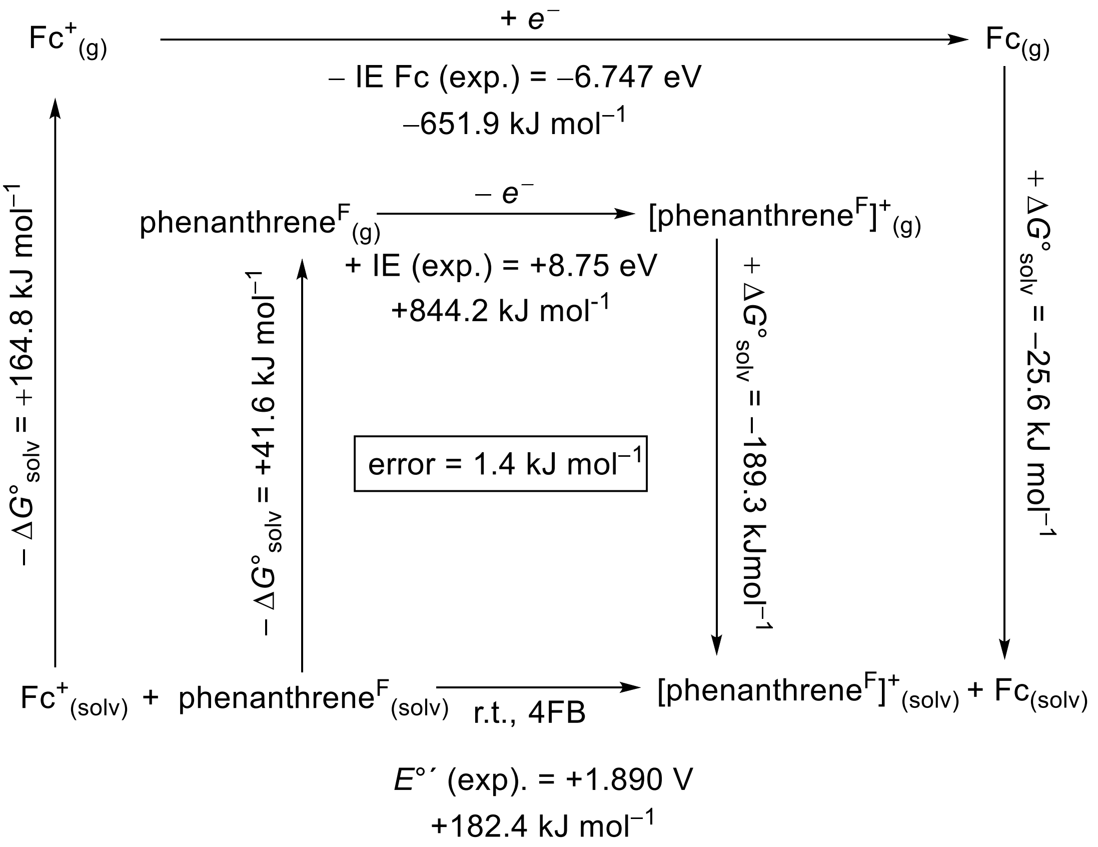

Supplementary Figure 100: Born-Haber-Fajans cycle of the reaction of phenanthrene^F with Fc^+ in 4FB using the experimental ionization energies of ferrocene[77] and phenanthrene^F,[78] cyclovoltammetric half-wave potential in 4FB and calculated solvation enthalpies from COSMO-RS.

5 Supplementary Note 5

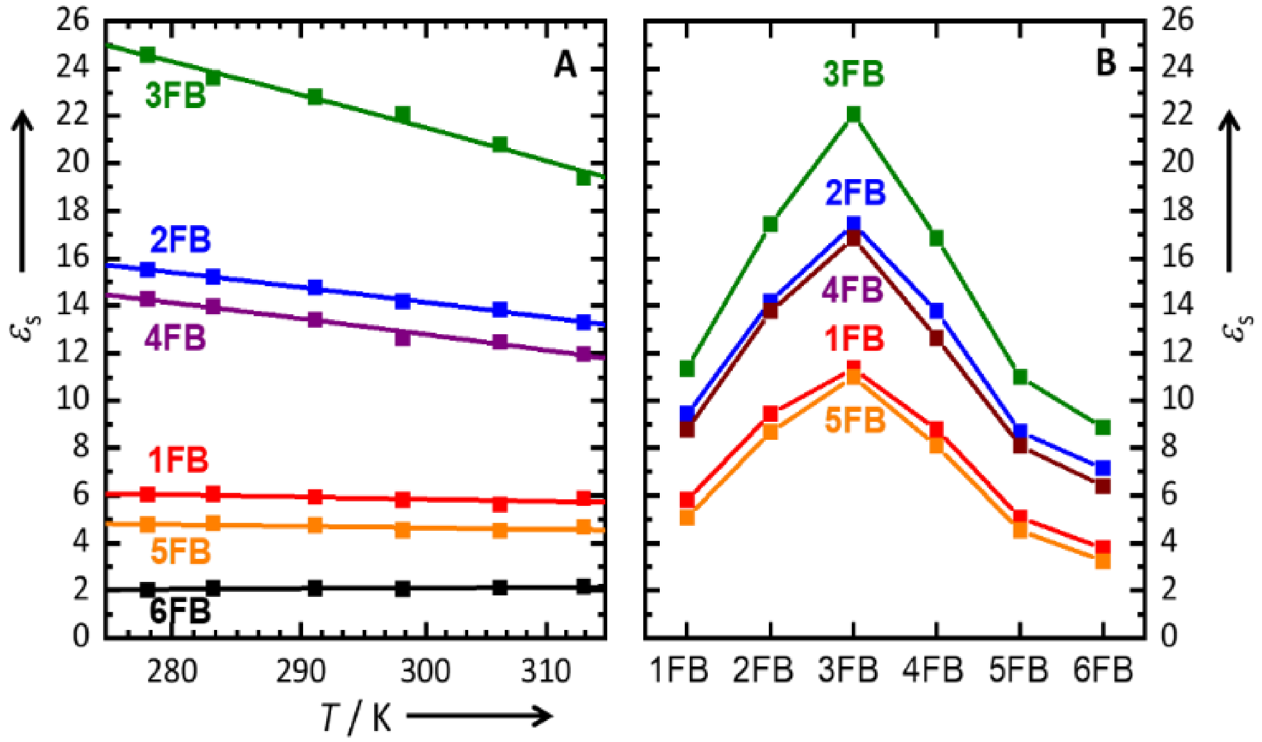

Supplementary Figure 101: Static dielectric constants of pure xFB solvents and mixtures **A** Static dielectric constants ε_S of the neat solvents as a function of the temperature *T*. Symbols correspond to the parameters obtained from fitting Eq. (S8), Supplementary Information, to the experimental spectra. Solid lines are linear fits. **B** Static dielectric constants ε_S for binary 1:1 xFB mixtures. Full data and analyses are deposited in the Supplementary Information section 5.

To study the dielectric response of all samples we use dielectric relaxation spectroscopy (DRS),[79,80] which measures the polarization response of the samples in an external oscillating electric field. The polarization dynamics are determined by recording the complex permittivity, $\hat{\varepsilon}(\nu)$, with $\varepsilon'(\nu)$ the real part and $\varepsilon''(\nu)$ the imaginary part of the complex permittivity as a function of the field frequency (ν) (see eq. (S7)):

$$\hat{\varepsilon}(\nu) = \varepsilon'(\nu) - i\varepsilon''(\nu) \tag{S7}$$

All spectra (except for 6FB, for which no significant frequency dependence of the dielectric function could be detected) were modelled using a single Debye relaxation:[81]

$$\hat{\varepsilon}(\nu) = \frac{\varepsilon_S - \varepsilon_\infty}{1 - 2\pi\nu\tau} + \varepsilon_\infty \tag{S8}$$

with ε_S the low frequency limit of $\varepsilon'(\nu)$ (i.e. the static dielectric constant), τ the dielectric relaxation time, and ε_∞ the limiting value of $\varepsilon'(\nu)$ at high frequencies, which subsumes all polarization dynamics that contribute at frequencies, higher than those covered experimentally in the present study.

For 6FB we modelled the spectra by a constant value $\hat{\varepsilon}(\nu) = \varepsilon_S$. The average values of these parameters from the three experiments are listed in Supplementary Table 32-Supplementary Table 35.

Supplementary Table 32: Relaxation parameters obtained by fitting eq. (**S8**) to the dielectric spectra of the neat solvents at six different temperatures: Static dielectric constant $\boldsymbol{\varepsilon_s}$, infinite frequency permittivity $\boldsymbol{\varepsilon_\infty}$, and relaxation time $\boldsymbol{\tau}$.

	ε_S	ε_∞	τ / ps	ε_S	ε_∞	τ / ps	ε_S	ε_∞	τ / ps
		**278 K**			**283 K**			**291 K**	
1FB	6.1	2.6	9.9	6.1	2.5	7.7	5.9	2.5	7.5
2FB	15.5	2.8	14.6	15.2	2.7	13.5	14.8	2.7	11.7
3FB	24.6	2.7	22.8	23.6	2.7	20.3	22.8	2.7	18.2
4FB	14.3	2.4	21.2	14.0	2.4	19.1	13.4	2.4	16.7
5FB	4.8	2.1	16.5	4.8	2.1	15.5	4.7	2.1	13.5
6FB	2.0	-	-	2.1	-	-	2.1	-	-
	ε_S	ε_∞	τ / ps	ε_S	ε_∞	τ / ps	ε_S	ε_∞	τ / ps
		**298 K**			**306 K**			**313 K**	
1FB	5.8	2.4	6.4	5.6	2.3	5.8	5.9	2.4	5.4
2FB	14.2	2.5	10.2	13.8	2.5	9.8	13.3	2.4	8.8
3FB	22.1	2.6	16.9	20.8	2.6	15.1	19.4	2.5	13.1
4FB	12.6	2.3	14.7	12.5	2.4	14.0	12.0	2.3	12.3
5FB	4.5	2.1	11.2	4.5	2.1	11.2	4.7	2.1	9.8
6FB	2.1	-	-	2.1	-	-	2.2	-	-

Supplementary Table 33: Parameters obtained by fitting eq. (S8) to the dielectric spectra of the 1:1 mixtures at 283 K: Static dielectric constant $\boldsymbol{\varepsilon_s}$, infinite frequency permittivity $\boldsymbol{\varepsilon_\infty}$, and relaxation time $\boldsymbol{\tau}$.

	ε_S	ε_∞	τ / ps	ε_S	ε_∞	τ / ps	ε_S	ε_∞	τ / ps
	**1FB:xFB mixtures**			**2FB:xFB mixtures**			**3FB:xFB mixtures**		
1FB	6.1	2.5	7.7	10.0	2.6	10.5	12.4	2.7	13.3
2FB	10.0	2.6	10.5	15.2	2.7	13.5	19.0	2.7	16.5
3FB	12.4	2.7	13.3	19.0	2.7	16.5	23.6	2.7	20.3
4FB	9.6	2.5	13.8	14.8	2.6	16.5	18.6	2.6	20.0
5FB	5.3	2.2	11.9	9.4	2.4	14.2	11.8	2.5	16.6
6FB	3.9	2.2	8.9	7.6	2.4	12.5	9.5	2.4	15.3
	ε_S	ε_∞	τ / ps	ε_S	ε_∞	τ / ps	ε_S	ε_∞	τ / ps
	**4FB:xFB mixtures**			**5FB:xFB mixtures**			**6FB:xFB mixtures**		
1FB	9.6	2.5	13.8	5.3	2.2	11.9	3.9	2.2	8.9
2FB	14.8	2.6	16.5	9.4	2.4	14.2	7.6	2.4	12.5
3FB	18.6	2.6	20.0	11.8	2.5	16.6	9.5	2.4	15.3
4FB	14.0	2.4	19.1	8.8	2.3	17.0	6.8	2.2	15.9
5FB	8.8	2.3	17.0	4.8	2.1	15.5	3.4	2.1	13.9
6FB	6.8	2.2	15.9	3.4	2.1	13.9	2.1	-	-

Supplementary Table 34: Parameters obtained by fitting eq. (S8) to the dielectric spectra of the 1:1 mixtures at 298 K: Static dielectric constant $\boldsymbol{\varepsilon_s}$, infinite frequency permittivity $\boldsymbol{\varepsilon_\infty}$, and relaxation time $\boldsymbol{\tau}$.

	ε_S	ε_∞	τ / ps	ε_S	ε_∞	τ / ps	ε_S	ε_∞	τ / ps
	**1FB:xFB mixtures**			**2FB:xFB mixtures**			**3FB:xFB mixtures**		
1FB	5.8	2.4	6.4	9.4	2.4	8.2	11.4	2.4	10.2
2FB	9.4	2.4	8.2	14.2	2.5	10.2	17.4	2.5	13.0
3FB	11.4	2.4	10.2	17.4	2.5	13.0	22.1	2.6	16.9
4FB	8.8	2.4	10.5	13.8	2.5	13.1	16.8	2.5	15.6
5FB	5.1	2.2	8.7	8.7	2.3	10.8	11.0	2.4	13.3
6FB	3.8	2.1	6.3	7.2	2.3	9.6	8.9	2.3	12.1
	ε_S	ε_∞	τ / ps	ε_S	ε_∞	τ / ps	ε_S	ε_∞	τ / ps
	**4FB:xFB mixtures**			**5FB:xFB mixtures**			**6FB:xFB mixtures**		
1FB	8.8	2.4	10.5	5.1	2.2	8.7	3.8	2.1	6.3
2FB	13.8	2.5	13.1	8.7	2.3	10.8	7.2	2.3	9.6
3FB	16.8	2.5	15.6	11.0	2.4	13.3	8.9	2.3	12.1
4FB	12.6	2.3	14.7	8.1	2.3	13.0	6.4	2.2	12.2
5FB	8.1	2.3	13.0	4.5	2.1	11.2	3.2	2.0	9.9
6FB	6.4	2.2	12.2	3.2	2.0	9.9	2.1	-	-

Supplementary Table 35: Parameters obtained by fitting eq. **(S8)** to the dielectric spectra of the 1:1 mixtures at 313 K: Static dielectric constant $\boldsymbol{\varepsilon_s}$, infinite frequency permittivity $\boldsymbol{\varepsilon_\infty}$, and relaxation time $\boldsymbol{\tau}$.

	ε_s	ε_∞	τ / ps	ε_s	ε_∞	τ / ps	ε_s	ε_∞	τ / ps
	**1FB:xFB mixtures**			**2FB:xFB mixtures**			**3FB:xFB mixtures**		
1FB	5.9	2.4	5.4	8.9	2.4	7.0	10.8	2.4	8.8
2FB	8.9	2.4	7.0	13.3	2.4	8.8	16.0	2.5	11.0
3FB	10.8	2.4	8.8	16.0	2.5	11.0	19.4	2.5	13.1
4FB	8.4	2.4	9.2	12.7	2.4	11.0	15.7	2.5	13.2
5FB	5.0	2.3	7.9	8.3	2.2	9.1	10.5	2.4	11.5
6FB	3.7	2.2	5.6	7.0	2.4	8.5	8.6	2.4	10.5
	ε_s	ε_∞	τ / ps	ε_s	ε_∞	τ / ps	ε_s	ε_∞	τ / ps
	**4FB:xFB mixtures**			**5FB:xFB mixtures**			**6FB:xFB mixtures**		
1FB	8.4	2.4	9.2	5.0	2.3	7.9	3.7	2.2	5.6
2FB	12.7	2.4	11.0	8.3	2.2	9.1	7.0	2.4	8.5
3FB	15.7	2.5	13.2	10.5	2.4	11.5	8.6	2.4	10.5
4FB	12.0	2.3	12.3	7.8	2.2	11.3	6.2	2.2	10.6
5FB	7.8	2.2	11.3	4.7	2.1	9.8	3.3	2.1	9.2
6FB	6.2	2.2	10.6	3.3	2.1	9.2	2.2	-	-

5.1 Neat solvents at 298 K

We show the dielectric spectra for the neat fluorobenzenes at 298K together with the static dielectric permittivities and the corresponding relaxations times at 298 K in Supplementary Figure 102.

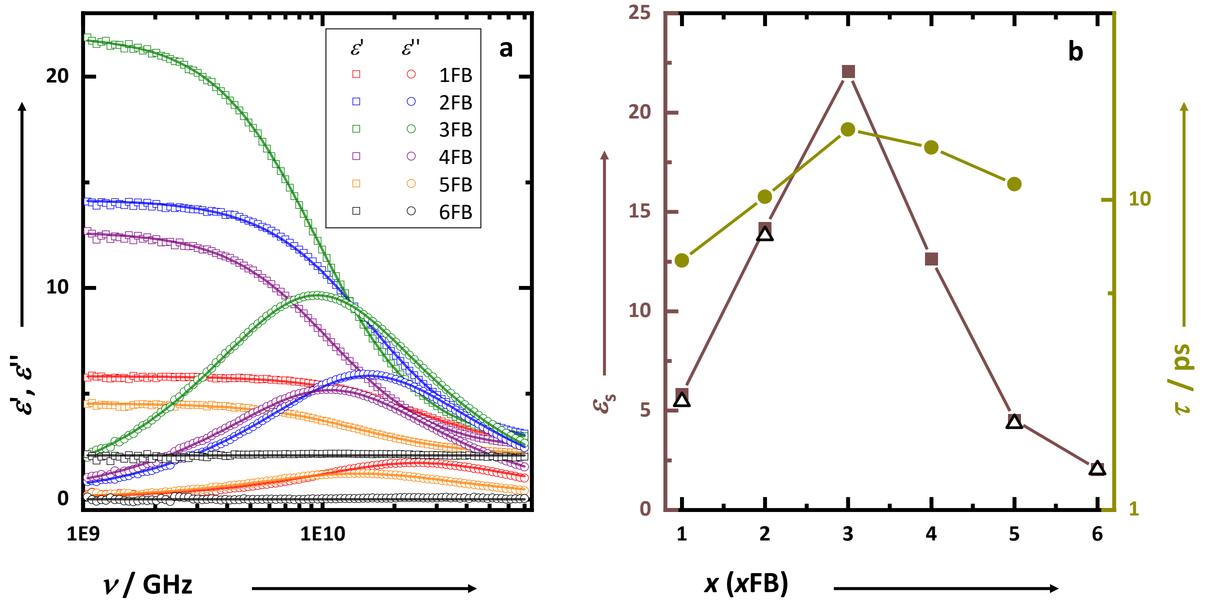

Supplementary Figure 102: (a) Complex permittivity spectra of the neat solvents 1FB, 2FB, 3FB, 4FB, 5FB and 6FB at 298 K. Symbols show experimental data and solid lines show the fits of eq. **(S8)** to the data. (b) Static dielectric constant (solid squares, left-hand axis) and relaxations time (solid circles, right-hand axis) as a function of the number of fluorine atoms, as obtained from fitting eq. **(S8)** to the experimental spectra. Data in (b) show the average of the parameters obtained from fitting the spectra of three independent experiments. Open triangles indicate literature values for $\boldsymbol{\varepsilon_s}$: 1FB at 293 K[68], 2FB at 293 K[68], 5FB at 298 K[82], and 6FB at 298 K[68].

As can be seen in Supplementary Figure 102a, a Debye relaxation (eq. (S8)) models the experimental data very well. The obtained dielectric constants at ambient temperature agree well with literature values for 1FB, 2FB, 5FB, and 6FB (Supplementary Figure 102b).[68,82] For the neat solvents we find the static dielectric

constant to increase from 1FB to 3FB with increasing fluorine substitution. Further fluorination results in a decrease of ε_s (Supplementary Figure 102b). As the static dielectric constant is dominated by alignment of molecular dipoles according to an external electric field (orientational polarization), the static dielectric constants reflect the trend in the molecular dipole moments of the fluorobenzenes: From the dielectric relaxation strengths ($\varepsilon_s - \varepsilon_\infty$), the effective dipole moments, μ_{eff}, in the liquid phase (including induced dipole moments in the condensed phase and also dipole-dipole correlations) of the fluorobenzenes can be determined according to:[81]

$$\mu_{eff} = \frac{2\varepsilon_s + 1}{\varepsilon_s}(\varepsilon_s - \varepsilon_\infty)\frac{k_B T \varepsilon_0}{N_A c} \tag{S9}$$

where k_B is the Boltzmann constant, T the thermodynamic temperature, ε_0 the permittivity of free space, and N_A Avogadro's constant. To calculate the molar concentrations, c, of the neat fluorobenzenes, we use the densities reported at ~296K in ref [83]. The thus determined effective dipole moments ($\mu_{eff}(1FB) = 1.9D$, $\mu_{eff}(2FB) = 3.6D$, $\mu_{eff}(3FB) = 4.7D$, $\mu_{eff}(4FB) = 3.5D$, $\mu_{eff}(5FB) = 1.8D$)), show the same trends as the variation of ε_s with fluorine substitution and are nearly symmetric: $\mu_{eff}(1FB) \approx \mu_{eff}(5FB)$ and $\mu_{eff}(2FB) \approx \mu_{eff}(4FB)$. The dipole moment increases with increasing fluorine substitution: $\mu_{eff}(1FB) < \mu_{eff}(2FB) < \mu_{eff}(3FB)$. Further fluorine substitution results in a reduction of the dipole moment, in line with the partial dipole moments of two fluorine substituents in para position cancelling: $\mu_{eff}(3FB) > \mu_{eff}(4FB) > \mu_{eff}(5FB)$.

These experimental effective dipole moments are higher than the dipole moments calculated using density functional theory using the ORCA[16,31] program package (M06[84] functional with def2-TZVPP basis set[9]; the solvent is approximated using the CPCM solvation model[73] and the dielectric constants listed in Supplementary Table 32): $\mu_{DFT}(1FB) = 1.84D$, $\mu_{DFT}(2FB) = 3.17D$, $\mu_{DFT}(3FB) = 3.64D$, $\mu_{DFT}(4FB) = 3.07D$, $\mu_{DFT}(5FB) = 1.64D$. Given that the solvation model accounts for induced dipole moments,[85] the difference between μ_{DFT} and μ_{eff} can be ascribed to dipolar correlations $\mu_{eff} = \sqrt{g}\mu_{DFT}$, where g is the Kirkwood correlation factor.[81] Based on the experimental and DFT values, our results suggest a slight tendency for positive (parallel) dipolar correlations with values of g somewhat higher than 1 (1.05-1.14).

Also, the dielectric relaxation time, τ, initially increases with increasing fluorine substitution and reaches a maximum for 3FB. Further fluorination results in a decrease in τ (Supplementary Figure 102b). To obtain the dipolar rotational correlation time, the dielectric relaxation time has to be corrected for the different local field in the liquids with different dielectric constants.[81] Yet, such correction does not affect the trend of the rotation time with increasing fluorine substitution and 3FB exhibits the longest rotational correlation time of the studied neat solvents at 298 K. For uncorrelated diffusive rotation of the dipolar fluorobenzenes, the Stokes-Einstein-Debye relation[81] predicts the relaxation time to scale with the dynamic viscosity of the solvents and the molecular volume of the rotating dipoles. The viscosity of the fluorobenzenes increase continuously from 1FB to 6FB, see Darges *et al.*[86] and section 9. Also, the molecular volumes obviously increase with increasing F substitution. As both, molecular volume and viscosity increase with increasing fluorination, the observed maximum of τ for 3FB cannot solely be explained by rotational diffusion. Rather, the observed maximum of τ for 3FB (Supplementary Figure 102b) correlates with the dipole moment of the fluorobenzenes. As such, our data suggest that dipole-dipole interactions, which scale with the dipoles magnitude, critically affect the dielectric relaxation time – in line with the parallel dipolar correlations observed for the relaxation strengths above.[87]

5.2 Temperature dependent relaxation of neat solvents

In Supplementary Figure 103 we show the temperature dependent spectra for the neat solvents, together with the fits of eq. (S8) to the data.

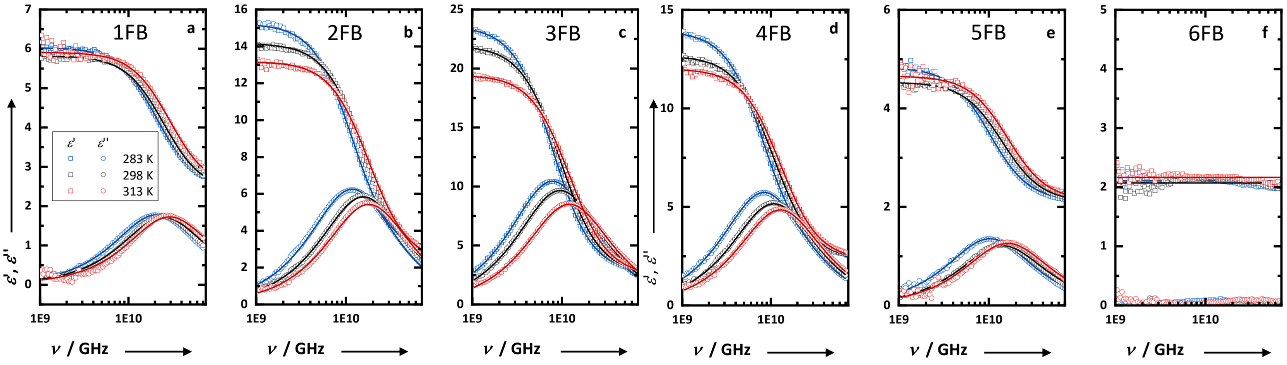

Supplementary Figure 103: (a) Complex permittivity spectra at 283, 298, and 313 K for (a) 1FB, (b) 2FB, (c) 3FB, (d) 4FB, (e) 5FB and (f) 6FB (other temperatures omitted for visual clarity). Symbols show experimental data and solid lines show the fits of eq. (**S8**) to the data.

Upon increasing temperature, we find the dispersion in the permittivity and the peak in the dielectric loss to gradually shift to higher frequencies. This shift of the Debye relaxation is indicative of faster relaxation dynamics with increasing temperature, consistent with a thermally activated relaxation process. To quantify the thermal activation, we determine the Arrhenius activation energy according to:

$$\tau(T) = \tau_0 e^{\frac{E_A}{RT}} \tag{S10}$$

where E_A is the activation energy, R the gas constant, and τ_0 the exponential pre-factor.

Such Arrhenius plots (Supplementary Figure 104b) demonstrate that the relaxation times markedly depend on fluorination, as already concluded from Supplementary Figure 102b.

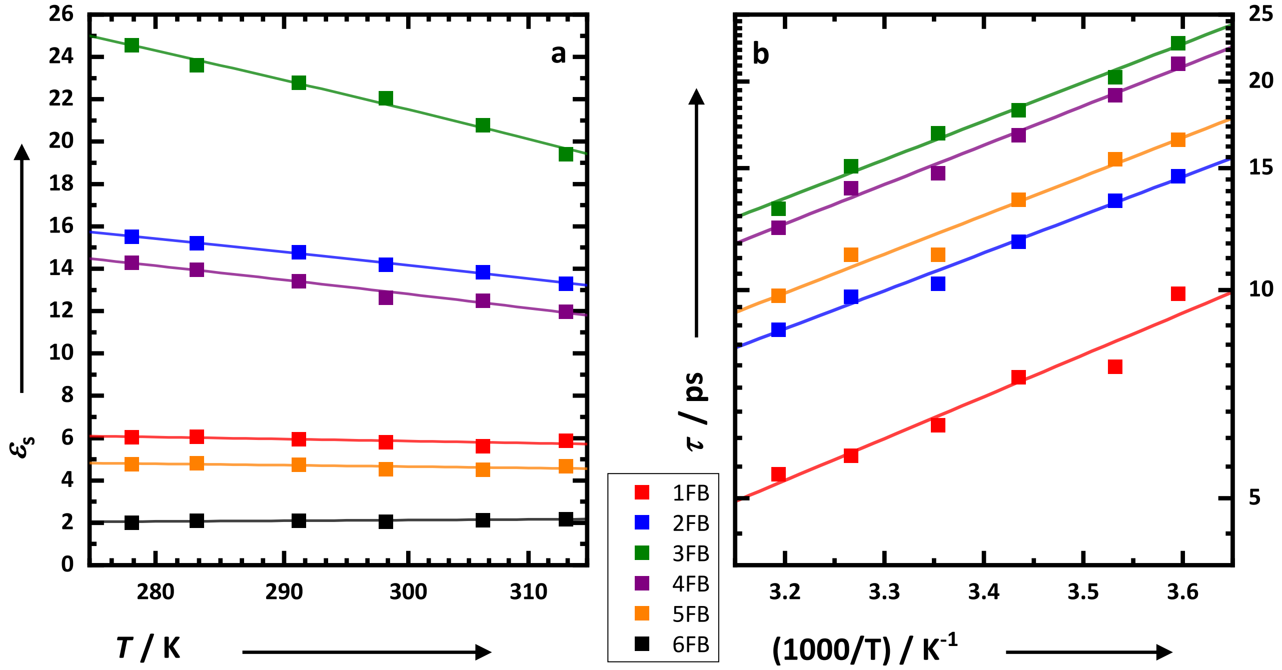

Supplementary Figure 104: (a) Static permittivity, ε_s, of the neat solvents as a function of temperature. Symbols correspond to the parameter obtained from fitting eq. (S8) to the experimental spectra. Solid lines are linear fits. (b) Relaxation time, τ, as a function of inverse temperature, $1/T$. Symbols correspond to the parameter obtained from fitting eq. (S8) to the experimental spectra. Solid lines are Arrhenius fits (eq. (S10)). All data show the average obtained from fitting the spectra of three independent experiments.

Yet, the slopes of such plots are rather insensitive to fluorination. From these slopes we obtain Arrhenius activation energies, E_A, ranging from 10.5 kJ mol^{-1} (2FB) to 11.6 kJ mol^{-1} (1FB), with no clear trend on the number of fluorine substituents. As such thermal activation of dipolar relaxation is rather insensitive to the degree of fluorination.

The relaxation amplitudes and the dielectric constants decrease with increasing temperature for all fluorobenzenes. This trend is consistent with thermal motion countering the alignment of the molecular-level dipoles according to the external field.

5.3 Relaxation of 1:1 mixtures

To further investigate dipolar correlations in fluorobenzenes we study a series of binary fluorobenzene mixtures. Supplementary Figure 105- Supplementary Figure 109 demonstrate that eq. (S8) provides an excellent description also for the binary (1:1) mixtures. This suggests that – despite one might expect for the binary mixtures either two relaxations of the two molecular species or a slightly broadened relaxation due to the different molecular volumes of both mixture components[81] – the dielectric response, can be well described assuming a single orientational relaxation process with a single characteristic relaxation time. Accordingly, the reorientation times of both mixture components are too similar to spectrally distinguish their individual contributions.

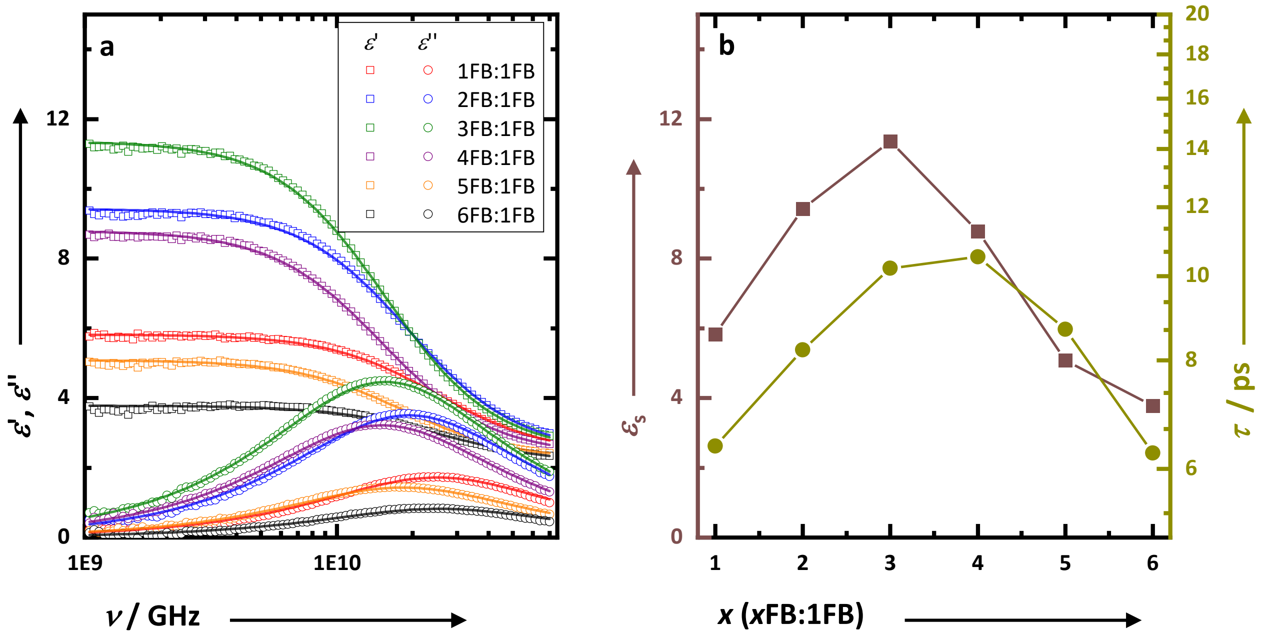

Supplementary Figure 105: (a) Complex permittivity spectra of 1:1 mixtures of 1FB, 2FB, 3FB, 4FB, 5FB, and 6FB with 1FB at 298 K. Symbols show experimental data and solid lines show the fits of eq. (S8) to the data. (b) Static dielectric constant (solid squares, left-hand axis) and relaxations time (solid circles, right-hand axis) as a function of the number of fluorine atoms of the co-solvent, as obtained from fitting eq. (S8) to the experimental spectra. Data in (b) show the average of the parameters obtained from fitting the spectra of three independent experiments.

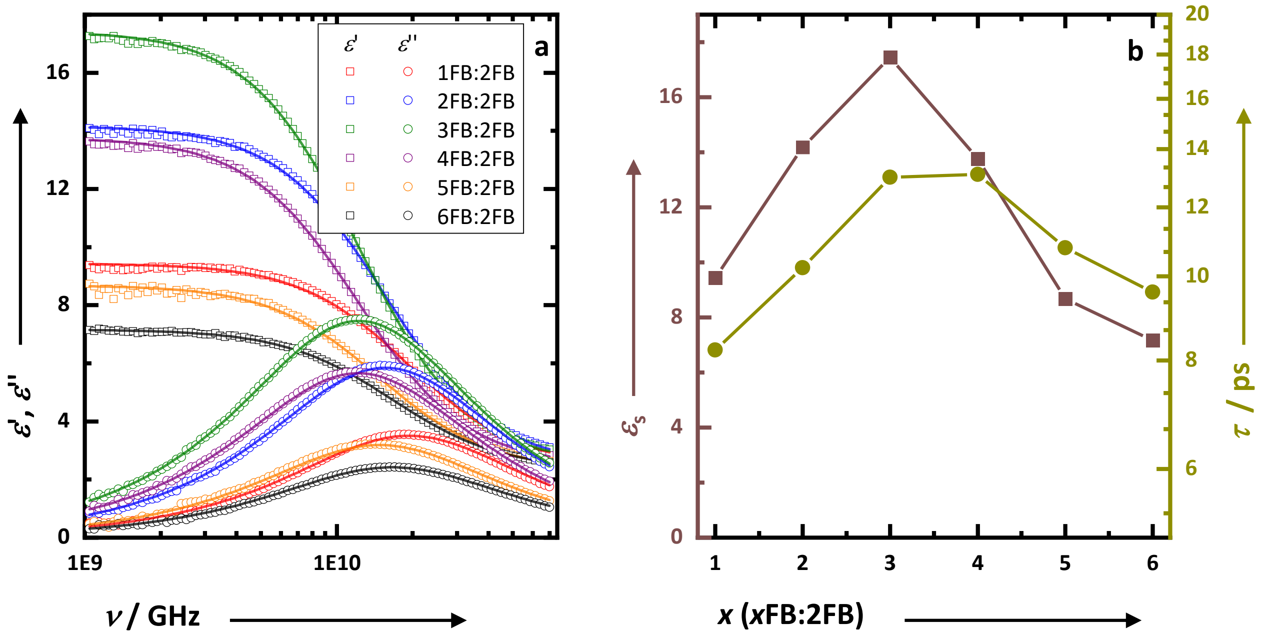

Supplementary Figure 106: (a) Complex permittivity spectra of 1:1 mixtures of 1FB, 2FB, 3FB, 4FB, 5FB, and 6FB with 2FB at 298 K. Symbols show experimental data and solid lines show the fits of eq. (S8) to the data. (b) Static dielectric constant (solid squares, left-hand axis) and relaxations time (solid circles, right-hand axis) as a function of the number of fluorine atoms of the co-solvent, as obtained from fitting eq. (S8) to the experimental spectra. Data in (b) show the average of the parameters obtained from fitting the spectra of three independent experiments.

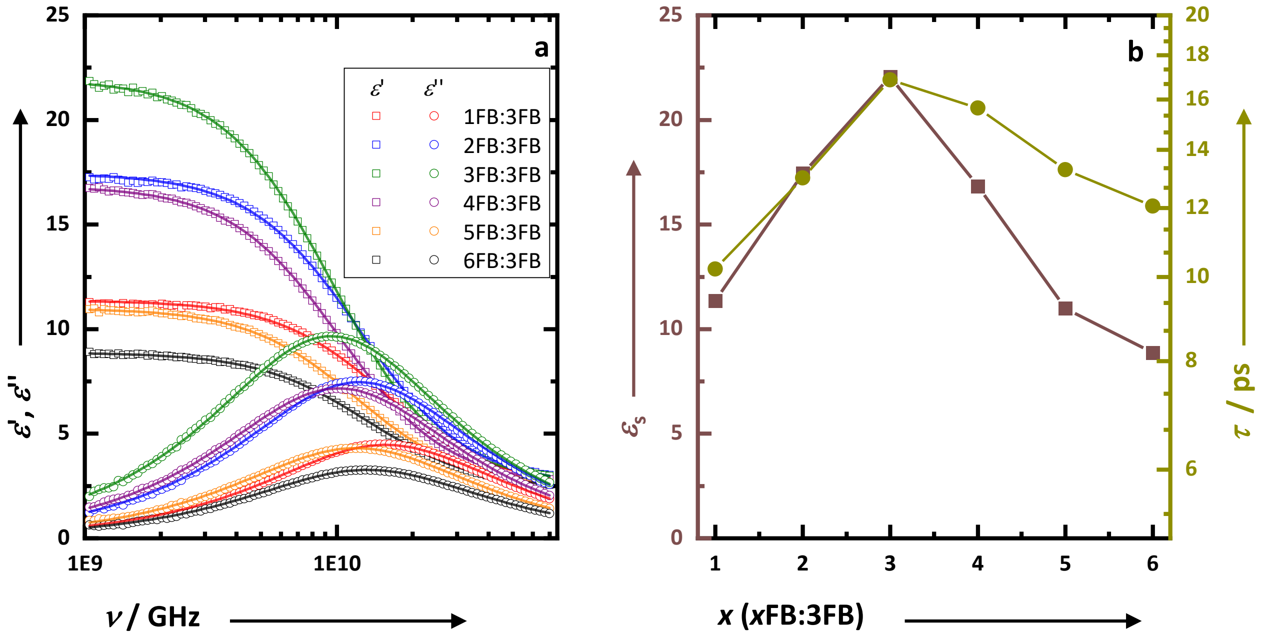

Supplementary Figure 107: (a) Complex permittivity spectra of 1:1 mixtures of 1FB, 2FB, 3FB, 4FB, 5FB, and 6FB with 3FB at 298 K. Symbols show experimental data and solid lines show the fits of eq. (S8) to the data. (b) Static dielectric constant (solid squares, left-hand axis) and relaxations time (solid circles, right-hand axis) as a function of the number of fluorine atoms of the co-solvent, as obtained from fitting eq. (S8) to the experimental spectra. Data in (b) show the average of the parameters obtained from fitting the spectra of three independent experiments.

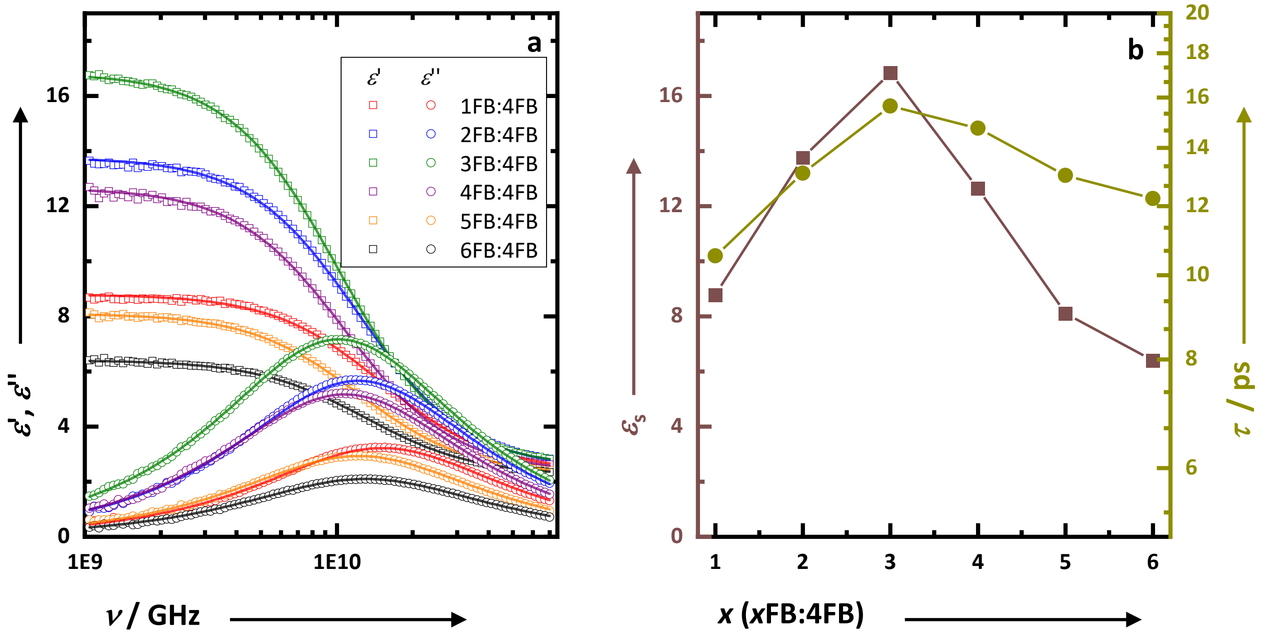

Supplementary Figure 108: (a) Complex permittivity spectra of 1:1 mixtures of 1FB, 2FB, 3FB, 4FB, 5FB, and 6FB with 4FB at 298 K. Symbols show experimental data and solid lines show the fits of eq. (S8) to the data. (b) Static dielectric constant (solid squares, left-hand axis) and relaxations time (solid circles, right-hand axis) as a function of the number of fluorine atoms of the co-solvent, as obtained from fitting eq. (S8) to the experimental spectra. Data in (b) show the average of the parameters obtained from fitting the spectra of three independent experiments.

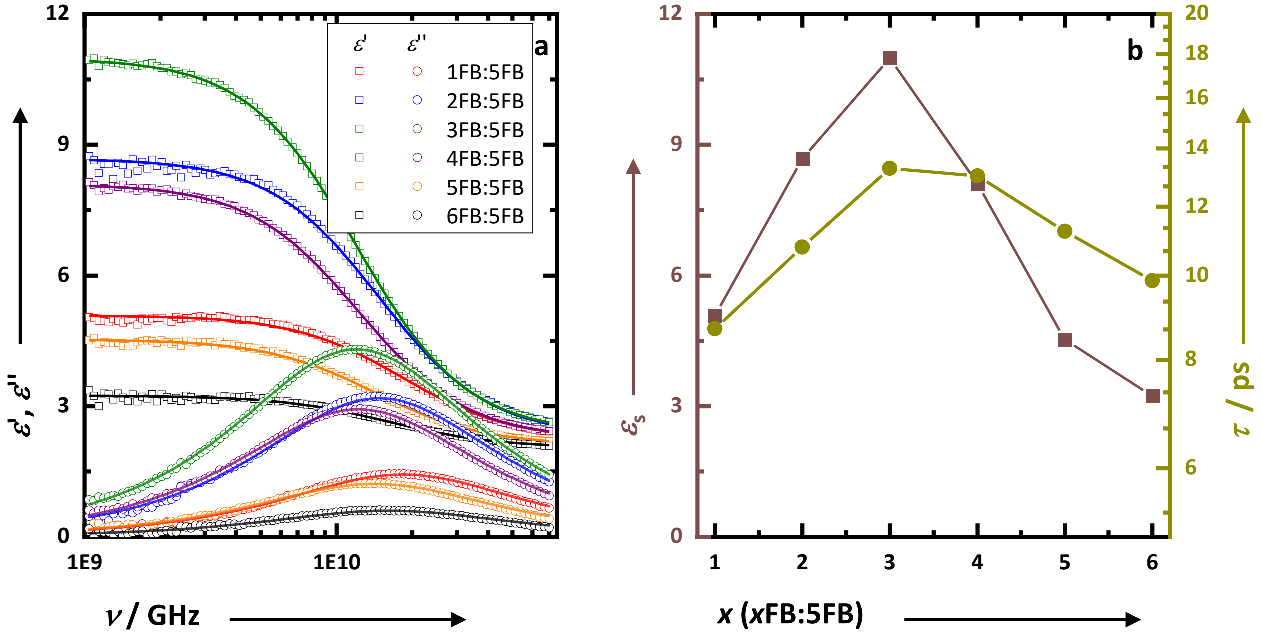

Supplementary Figure 109: (a) Complex permittivity spectra of 1:1 mixtures of 1FB, 2FB, 3FB, 4FB, 5FB, and 6FB with 5FB at 298 K. Symbols show experimental data and solid lines show the fits of eq. (S8) to the data. (b) Static dielectric constant (solid squares, left-hand axis) and relaxations time (solid circles, right-hand axis) as a function of the number of fluorine atoms of the co-solvent, as obtained from fitting eq. (S8) to the experimental spectra. Data in (b) show the average of the parameters obtained from fitting the spectra of three independent experiments.

The trends for the relaxation parameters of the mixtures parallel those found for the neat solvents: Upon increasing fluorine substitution for one mixture component with a constant second component we find both the static permittivity and the relaxation time to peak for mixtures with 3FB or 4FB, irrespective of the second component (Supplementary Figure 105b - Supplementary Figure 109b). Therefore, also the data for the mixtures support the notion that the molecular dipole moment of the varied component determines the magnitude of the static dielectric constant. Also, the relaxation time peaks for mixtures with 3FB or 4FB the

second varied component. This supports the above notion that the exact value of the relaxation time is markedly influenced by dipolar correlations: Dipolar interactions scale with the magnitude of the molecular dipole moment and are, as such, strongest for mixtures with highly dipolar 3FB and 4FB.

To analyse dipolar correlations in the mixtures quantitatively we use eq. (S9) to determine the apparent average dipole moment of the mixtures $\mu_{eff,av}$, by using the relaxation parameters of the mixtures (ε_S, ε_∞). We take the concentration in eq. (S9) as the total concentration of both mixture components $c = c_{xFB} + c_{yFB}$ assuming ideal mixing. We compare these apparent values to what would be expected for an ideal mixture of dipoles with dipole moments μ_{eff} as determined from the spectra of the neat fluorobenzene: $\mu_{eff,id} = \sqrt{0.5\mu_{eff}(xFB)^2 + 0.5\mu_{eff}(yFB)^2}$ in Supplementary Figure 110.

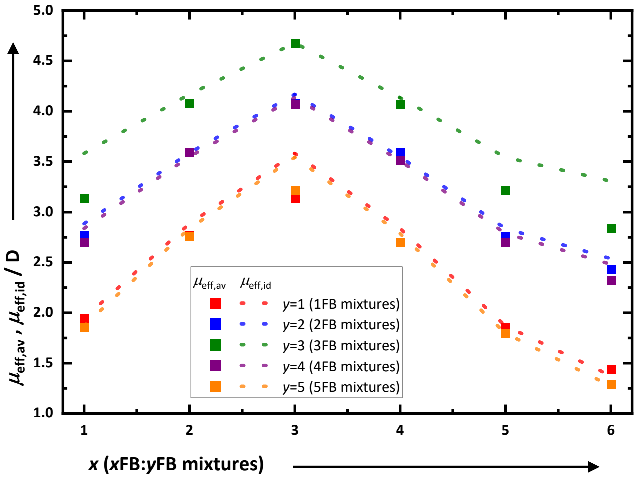

Supplementary Figure 110: Average effective dipole moments, $\boldsymbol{\mu_{eff,av}}$, (solid symbols) for binary xFB:yFB mixtures calculated using eq. (S9) assuming $\boldsymbol{c = c_{xFB} + c_{yFB}}$ compared to the ideal effective dipole moments $\boldsymbol{\mu_{eff,id} = \sqrt{0.5\mu_{eff}(xFB)^2 + 0.5\mu_{eff}(yFB)^2}}$ (dotted lines). Effective dipole moments $\boldsymbol{\mu_{eff}}$ were determined from the dielectric spectra of the neat solvents (see text).

As can be seen from this comparison, $\mu_{eff,id}$ is significantly higher than $\mu_{eff,av}$ when fluorobenzenes with a high dipole moment (e.g. 3FB) are mixed with weakly dipolar fluorobenzenes (e.g. 1FB, 5FB, or 6FB). This observation can again be explained by – preferential parallel – dipolar correlations for the highly dipolar fluorobenzenes: When highly dipolar fluorobenzenes are mixed with weakly dipolar fluorobenzenes, the dipolar correlations are reduced and $\mu_{eff,av} < \mu_{eff,id}$. As such, dilution of highly dipolar fluorobenzenes with weakly dipolar fluorobenzenes can weaken dipole-dipole interactions – presumably by increasing the average dipole-dipole distance between the highly dipolar species.

Despite these dipolar correlations, we find for all mixtures a monotonic decrease of the static permittivity with increasing temperature (Supplementary Figure 111). The temperature coefficients $d\,\varepsilon_S/d\,T$ correlate with the relaxation strength (~squared dipole moment of the mixtures components), consistent with the dielectric response being dominated by orientational polarization.

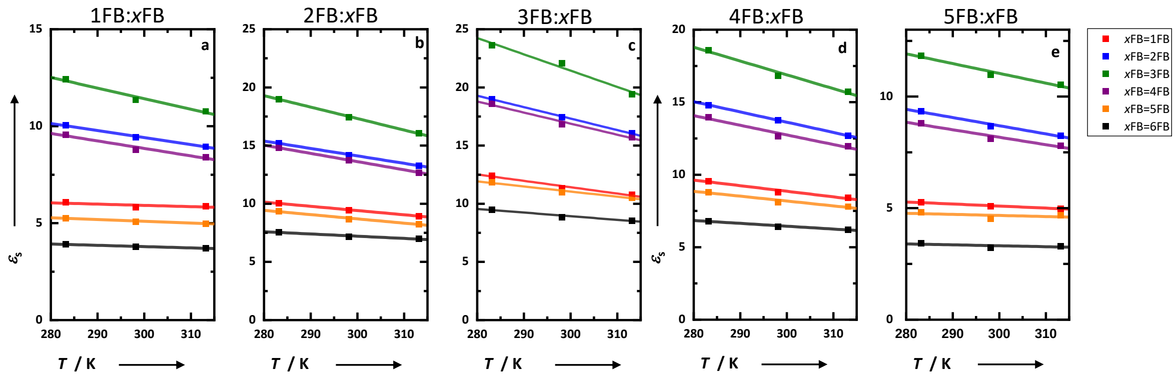

Supplementary Figure 111: Static permittivity, ε_s, of the binary solvent mixtures with (a) 1FB, (b) 2FB, (c) 3FB, (d) 4FB and (e) 5FB as a function of temperature. Symbols correspond to the parameters obtained from fitting eq. (S8) to the experimental spectra. Solid lines are linear fits. All data show the average of the parameters obtained from fitting the spectra of three independent experiments.

Similar to our findings for the neat solvents, the thermal activation of the relaxation time (Supplementary Figure 112) is very similar for all studied mixtures.

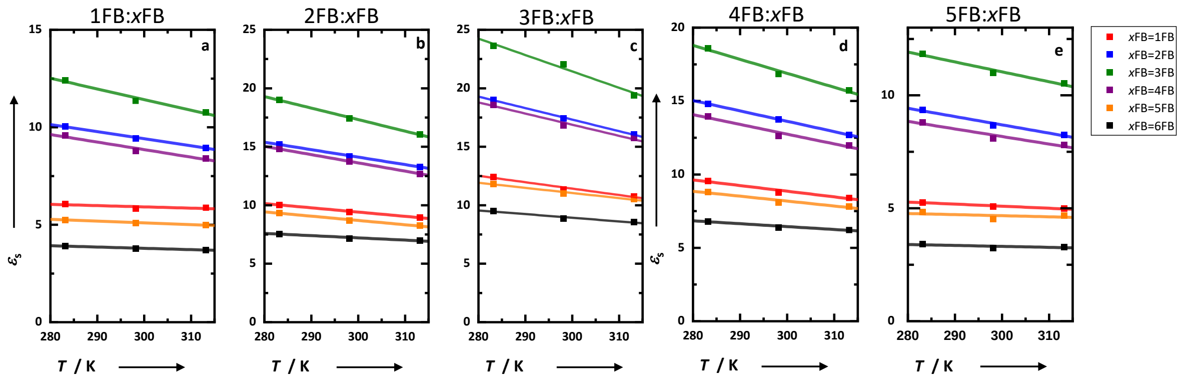

Supplementary Figure 112: Relaxation time, τ, of the binary solvent mixtures of 1FB-6FB with (a) 1FB, (b) 2FB, (c) 3FB, (d) 4FB and (e) 5FB as a function of inverse temperature, $1/T$. Symbols correspond to the parameter obtained from fitting eq. (S8) to the experimental spectra. Solid lines are Arrhenius fits (eq. (S10)). All data show the average of the parameters obtained from fitting the spectra of three independent experiments.

6 Supplementary Note 6

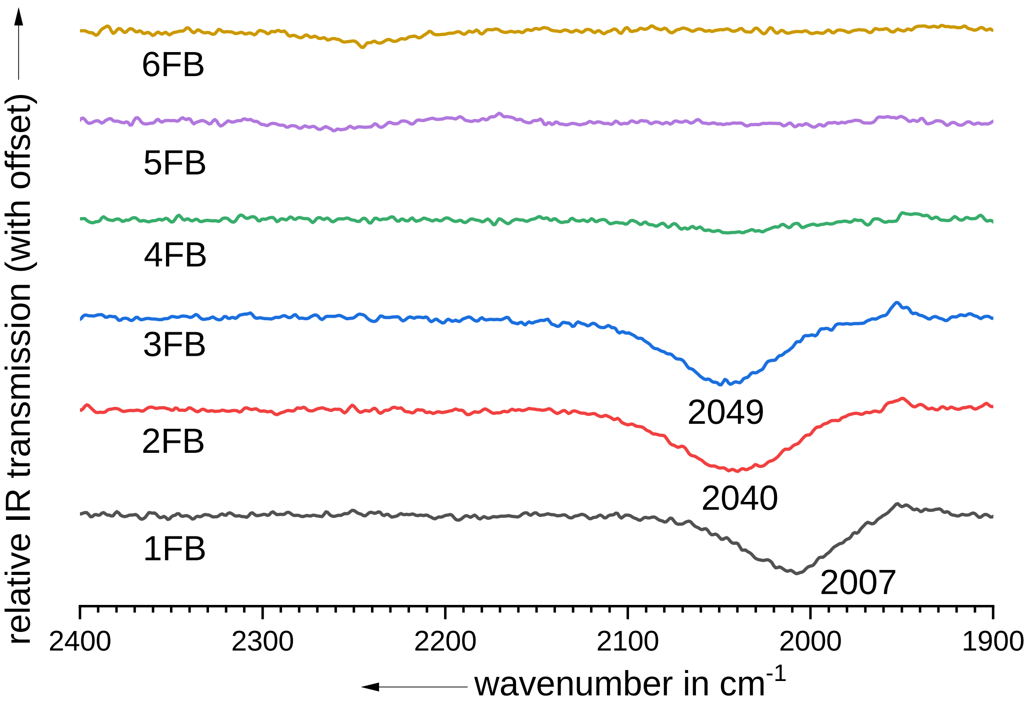

Supplementary Figure 113: FTIR-spectra (ZnSe, ATR) of $NO^+[pf]^-$ in xFB (x = 1-6) solutions, neat xFB was taken as background spectrum.

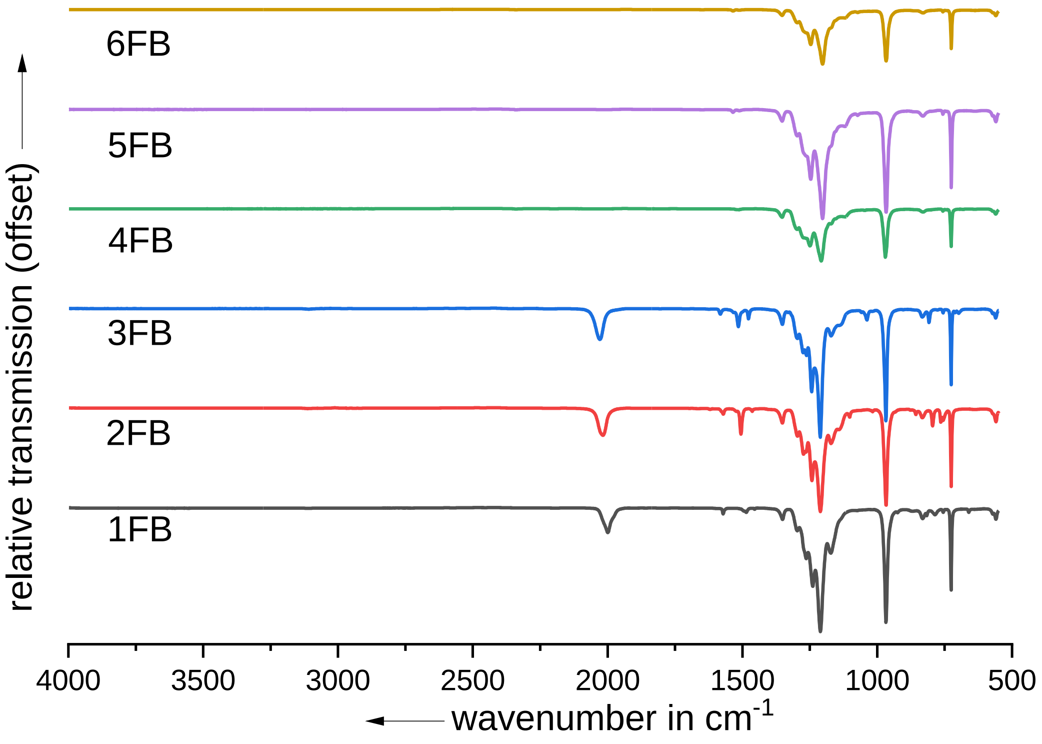

Supplementary Figure 114: FTIR-spectra (ZnSe, ATR) of $NO^+[pf]^-$ from dried xFB (x = 1-6) solutions in solid-state.

7 Supplementary Note 7

Supplementary Table 36: Crystallographic data for complexes $[Ag(2FB)_3]^+[pf]^-$, $[Ag(3FB)]^+[pf]^-$, $[Ag(4FB)]^+[pf]^-$.

	$[Ag(2FB)_3]^+[pf]^-$	$[Ag(3FB)]^+[pf]^-$	$[Ag(4FB)]^+[pf]^-$
**CCDC number**	2303021	2303022	2303023
**Empirical formula**	$C_{34}H_{12}AgAlF_{42}O_4$	$C_{22}H_3AgAlF_{39}O_4$	$C_{22}H_2AgAlF_{40}O_4$
**Formula weight**	1417.29	1207.09	1225.09
**Temperature [K]**	100(2)	100(2)	100(2)
**Crystal system**	orthorhombic	triclinic	monoclinic
**Space group (number)**	$Pca2_1$ (29)	$P\overline{1}$ (2)	$P\,2_1/n$ (14)
***a* [Å]**	29.578(10)	10.715(3)	20.8861(10)[a]
***b* [Å]**	10.489(4)	16.356(4)	16.8908(8)
***c* [Å]**	28.741(13)	19.628(5)	21.1315(10)
**α [°]**	90	90.728(7)	90
**β [°]**	90	91.616(12)	112.3370(10)
**γ [°]**	90	94.382(7)	90
**Volume [Å³]**	8916(6)	3428.2(16)	6895.5(6)
Z	8	4	8
**ρ_{calc} [gcm⁻³]**	2.112	2.339	2.360
**μ [mm⁻¹]**	0.689	0.861	0.862
***F*(000)**	5488	2312	4688
**Crystal size [mm³]**	0.264×0.224×0.111	0.30×0.13×0.05	0.24×0.20×0.12
**Crystal colour**	colourless	colourless	colourless
**Crystal shape**	block	plate	block
**Radiation**	MoK_α (λ=0.71073 Å)	MoK_α (λ=0.71073 Å)	MoK_α (λ=0.71073 Å)
**2Θ range [°]**	2.83 to 57.47 (0.74 Å)	3.22 to 51.58 (0.82 Å)	3.19 to 57.48 (0.74 Å)
**Index ranges**	−39 ≤ h ≤ 37<br>−14 ≤ k ≤ 14<br>−38 ≤ l ≤ 38	–13 ≤ h ≤ 13; –19 ≤ k ≤ 19; 0 ≤ l ≤ 23	-28 ≤ h ≤ 28<br>-22 ≤ k ≤ 22<br>-28 ≤ l ≤ 28
**Reflections collected**	240121	13714	294758
**Independent reflections**	23069<br>R_{int} = 0.0491<br>R_{sigma} = 0.0256	13714; R_{int} = 0.0889; R_{sigma} = 0.0584	17870<br>R_{int} = 0.0401<br>R_{sigma} = 0.0143
**Completeness to Θ = 25.242°**	99.9 %	100.0 %	100.0 %
**Data / Restraints / Parameters**	23069/18814/1805	13714/10195/1226	17870/15789/1473
**Goodness-of-fit on F^2**	1.165	1.142	1.067
**Final *R* indexes [I≥2σ(I)]**	R_1 = 0.0306<br>wR_2 = 0.0691	R_1 = 0.0784; wR_2 = 0.1919	R_1 = 0.0245<br>wR_2 = 0.0633
**Final *R* indexes [all data]**	R_1 = 0.0364<br>wR_2 = 0.0731	R_1 = 0.0886; wR_2 = 0.1983	R_1 = 0.0278<br>wR_2 = 0.0653
**Flack X parameter**	0.004(4)	-	-
**Largest peak/hole [eÅ⁻³]**	0.69/-0.38	1.59/–1.27	1.01/-0.41

[a] Rows of reflexes with alternating intensity were observed along the a*-axis. Both the intense and less intense reflexes were considered resulting in a larger cell (supercell). The molecular moieties in the asymetric unit differ slightly in their conformation.

Supplementary Table 37: Crystallographic data for [2FB···NO]$^+$[*pf*]$^-$, [anthracene^F]$^{+\cdot}$[*al-f-al*]$^-$, [anthracene^F]$^{+\cdot}$[*al-f-al*]$^-$·5FB and [phenanthrene^F]$^{+\cdot}$[*al-f-al*]$^-$

	**[2FB···NO]$^+$[*pf*]$^-$**	**[anthracene^F]$^{+\cdot}$[*al-f-al*]$^-$**	**[anthracene^F]$^{+\cdot}$[*al-f-al*]$^-$ ·5FB**	**[phenanthrene^F]$^{+\cdot}$[*al-f-al*]$^-$**
**CCDC number**	2303024	23030325	2303026	2177466
**Empirical formula**	$C_{22}H_4AlF_{38}NO_5$	$C_{38}Al_2F_{65}O_6$	$C_{44}HAl_2F_{70}O_6$	$C_{38}Al_2F_{65}O_6$
**Formula weight**	1111.24	1841.34	2009.41	1841.34
**Temperature [K]**	100(2)	100(2)	100(2)	100(2)
**Crystal system**	monoclinic	monoclinic	triclinic	monoclinic
**Space group (number)**	$P\,2_1/c$ (14)	$P2_1/c$ (14)	$P\overline{1}$ (2)	$P\,2_1/c$ (14)
***a* [Å]**	10.288(2)	11.957(4)	13.533(3)	23.166(3)
***b* [Å]**	10.8467(14)	21.937(2)	13.741(3)	18.144(3)
***c* [Å]**	30.651(11)	10.8451(11)	16.944(5)	13.3847(14)
**α [°]**	90	90	91.36(2)	90
**β [°]**	98.924(15)	107.170(17)	106.811(14)	105.719(5)
**γ [°]**	90	90	92.517(19)	90
**Volume [Å^3]**	3379.0(15)	2718.0(11)	3011.2(12)	5415.6(12)
Z	4	2	2	4
**ρ_{calc} [gcm^{-3}]**	2.184	2.250	2.216	2.258
**μ [mm^{-1}]**	0.311	0.327	0.316	0.328
***F*(000)**	2152	1774	1938	3548
**Crystal size [mm^3]**	0.253×0.210×0.179	0.305×0.220×0.058	0.153×0.101×0.095	0.242×0.160×0.094
**Crystal colour**	red	blue	blue	green
**Crystal shape**	block	plate	prism	block
**Radiation**	MoK_α (λ=0.71073 Å)	MoK_α (λ=0.71073 Å)	MoK_α (λ=0.71073 Å)	MoK_α (λ=0.71073 Å)
**2Θ range [°]**	2.69 to 57.49 (0.74 Å)	3.57 to 61.16 (0.70 Å)	2.51 to 55.11 (0.77 Å)	2.89 to 59.27 (0.72 Å)
**Index ranges**	−13 ≤ h ≤ 13<br>−14 ≤ k ≤ 14<br>−41 ≤ l ≤ 41	−17 ≤ h ≤ 17<br>−31 ≤ k ≤ 31<br>−15 ≤ l ≤ 15	−17 ≤ h ≤ 17<br>−17 ≤ k ≤ 17<br>−22 ≤ l ≤ 22	−32 ≤ h ≤ 32<br>−25 ≤ k ≤ 25<br>−18 ≤ l ≤ 17
**Reflections collected**	73682	209733	79431	209632
**Independent reflections**	8744<br>R_{int} = 0.0479<br>R_{sigma} = 0.0246	8330<br>R_{int} = 0.0834<br>R_{sigma} = 0.0189	13829<br>R_{int} = 0.0434<br>R_{sigma} = 0.0287	15256<br>R_{int} = 0.0485<br>R_{sigma} = 0.0188
**Completeness to Θ = 25.242°**	100.0 %	100.0 %	99.7 %	100.0 %
**Data / Restraints / Parameters**	8744/9305/1058	8330/6723/883	13829/11425/1456	15256/5901/1003
**Goodness-of-fit on *F*^{2}**	1.053	1.126	1.013	1.000
**Final *R* indexes [*I*≥2σ(*I*)]**	R_1 = 0.0326<br>wR_2 = 0.0733	R_1 = 0.0290<br>wR_2 = 0.0722	R_1 = 0.0362<br>wR_2 = 0.0905	R_1 = 0.0327<br>wR_2 = 0.0830
**Final *R* indexes [all data]**	R_1 = 0.0491<br>wR_2 = 0.0835	R_1 = 0.0454<br>wR_2 = 0.0828	R_1 = 0.0508<br>wR_2 = 0.1014	R_1 = 0.0403<br>wR_2 = 0.0898
**Largest peak/hole [eÅ^{-3}]**	0.32/-0.34	0.32/-0.35	0.50/-0.30	0.55/-0.34

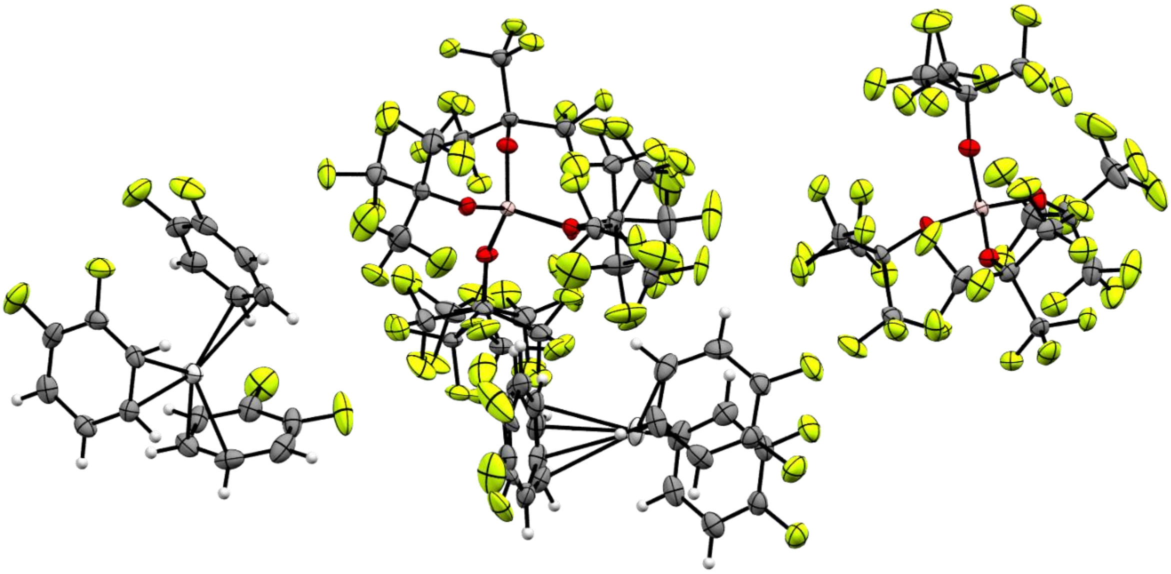

Supplementary Figure 115: ORTEP drawing of the asymmetric unit including disorders of $[Ag(2FB)_3]^+[pf]^-$ (CCDC 2303021). Thermal ellisoids drawn at 50 % probability, color code: silver – gray, aluminium – rose, fluorine – bright green, oxygen – red, carbon – dark gray.

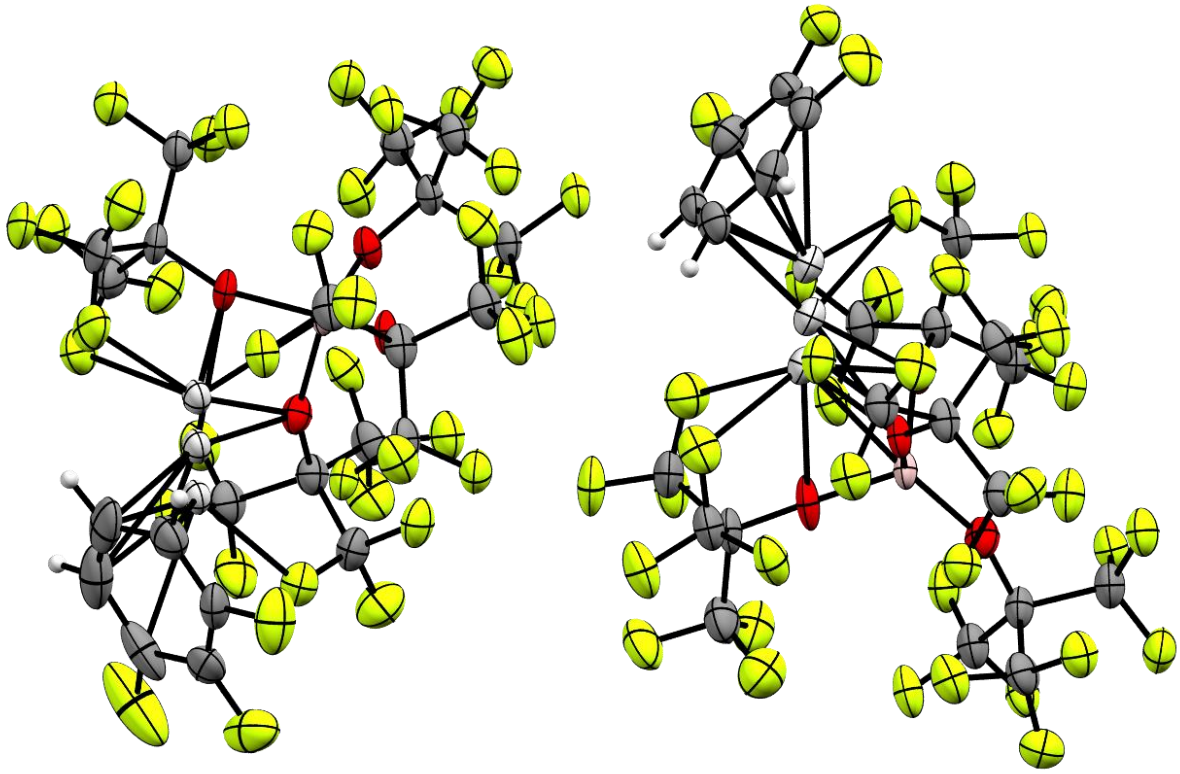

Supplementary Figure 116: ORTEP drawing of the asymmetric unit including disorders of $\{Ag(3FB)[pf]\}_{ip}$ (CCDC 2303022). Thermal ellisoids drawn at 50 % probability, color code: silver – gray, aluminium – rose, fluorine – bright green, oxygen – red, carbon – dark gray.

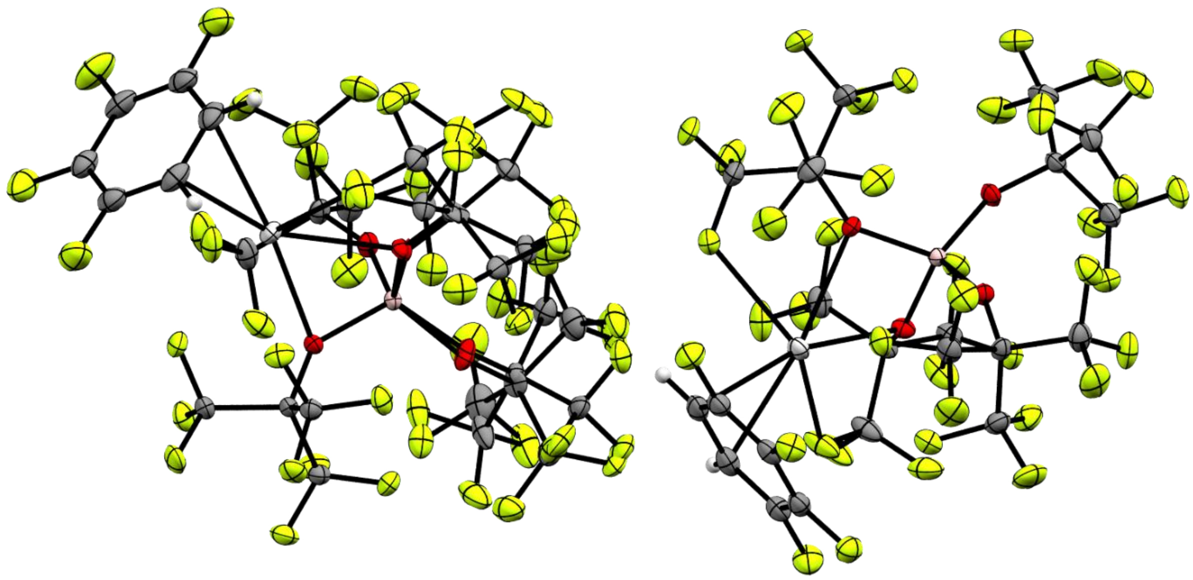

Supplementary Figure 117: ORTEP drawing of the asymmetric unit including disorders of {Ag(4FB)[*pf*]}$_{ip}$ (CCDC 2303023). Thermal ellisoids drawn at 50 % probability, color code: silver – gray, aluminium – rose, fluorine – bright green, oxygen – red, carbon – dark gray.

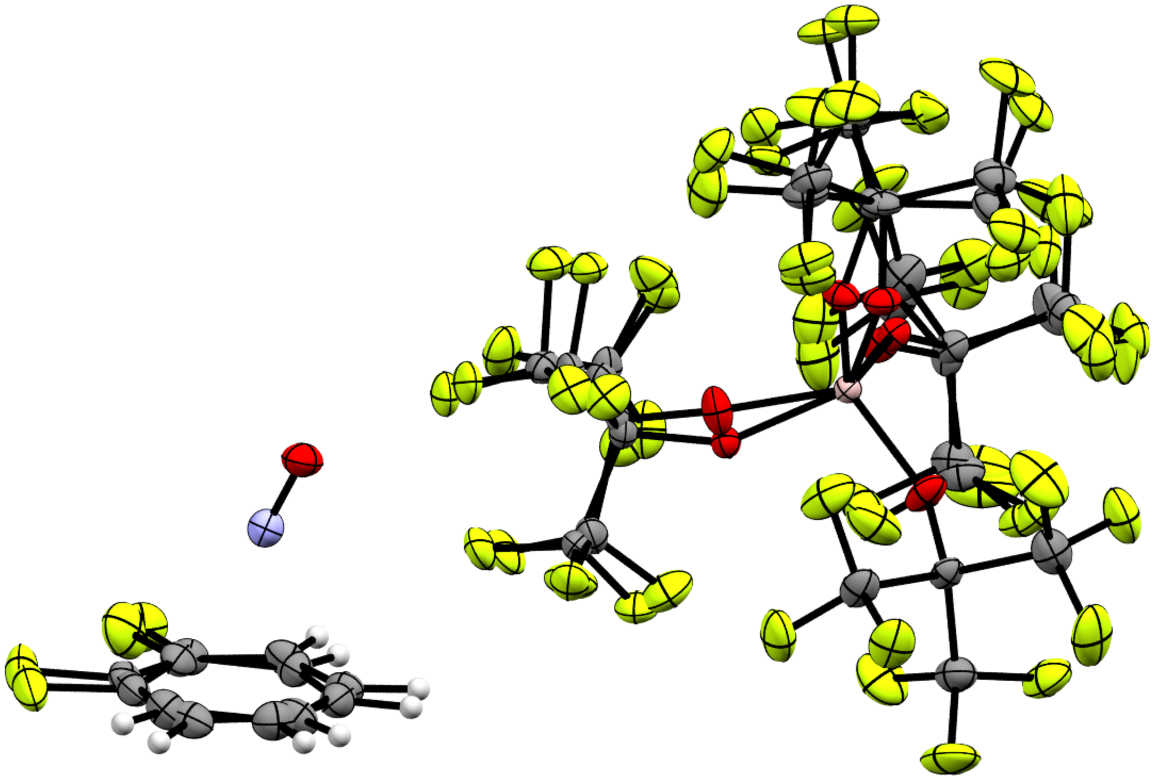

Supplementary Figure 118: ORTEP drawing of the asymmetric unit including disorders of $[NO(2FB)]^{+}$[*pf*]$^{-}$ (CCDC 2303024). Thermal ellisoids drawn at 50 % probability, color code: aluminium – rose, fluorine – bright green, oxygen – red, nitrogen – blue, carbon – dark gray.

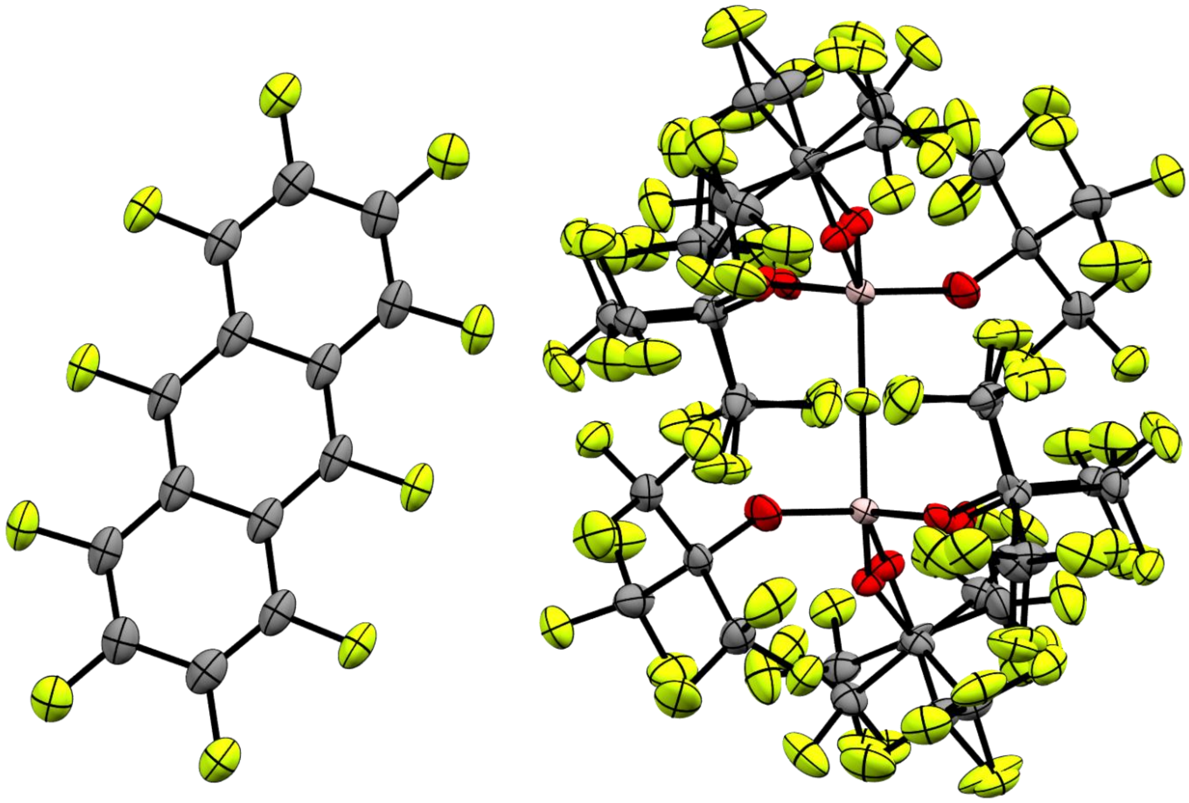

Supplementary Figure 119: ORTEP drawing of the asymmetric unit (molecular fragments grown for clarity) including disorders of [anthracene^{F}]$^{+}$[*alfal*]$^{-}$ (CCDC 2303025). Thermal ellisoids drawn at 50 % probability, color code: aluminium – rose, fluorine – bright green, oxygen – red, carbon – dark gray.

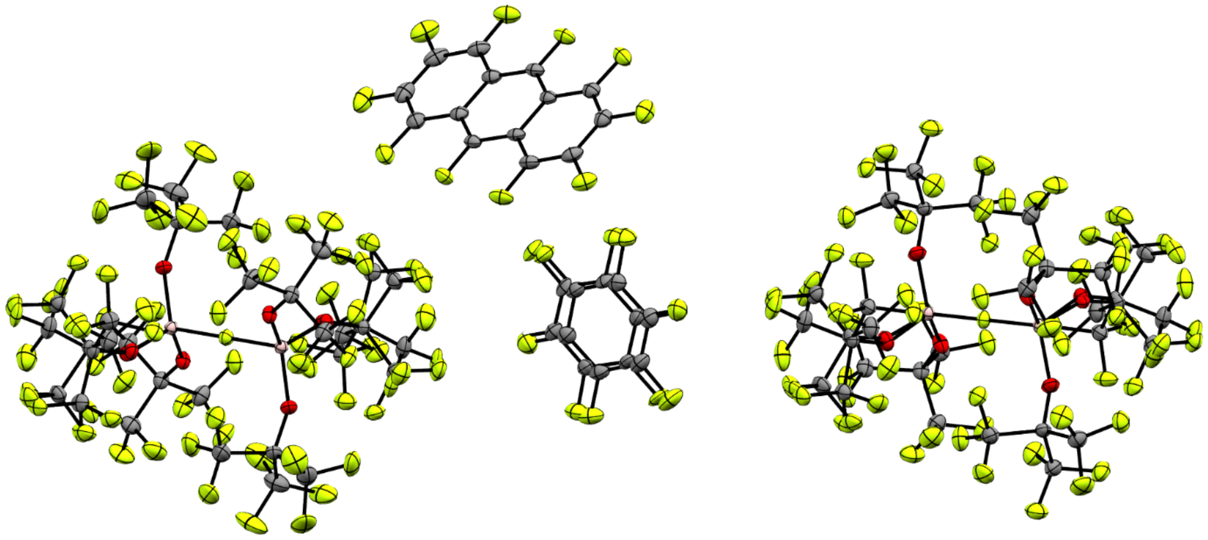

Supplementary Figure 120: ORTEP drawing of the asymmetric unit (molecular fragments grown for clarity) including disorders of [anthracene^{F}]$^{+}$[*alfal*]$^{-}$·5FB (CCDC 2303026). Thermal ellisoids drawn at 50 % probability, color code: aluminium – rose, fluorine – bright green, oxygen – red, carbon – dark gray.

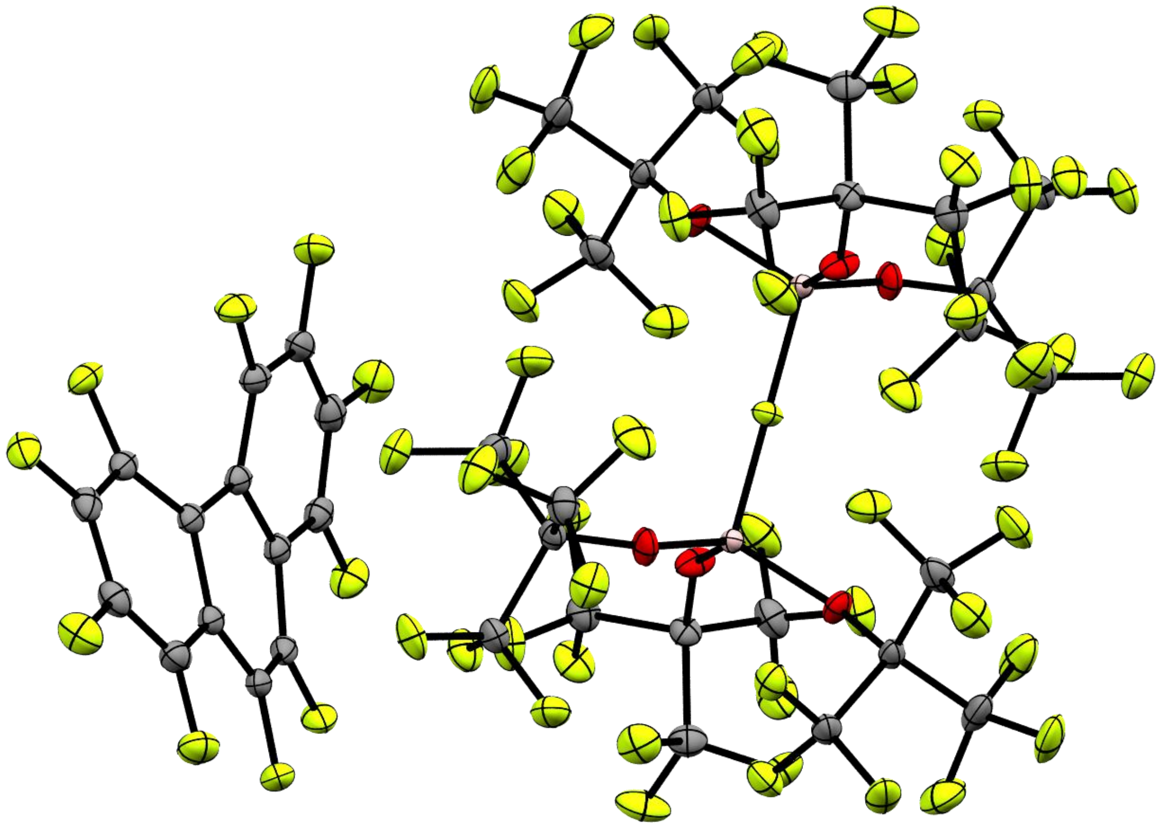

Supplementary Figure 121: ORTEP drawing of the asymmetric unit (molecular fragments grown for clarity) including disorders of [phenanthrene^{F}]$^{+}$[*alfal*]$^{-}$ (CCDC 2177466). Thermal ellisoids drawn at 50 % probability, color code: aluminium – rose, fluorine – bright green, oxygen – red, carbon – dark gray.

8 Supplementary Note 8

8.1 Original Measurement data

#EXPORTTYPE:DATA ALL
#FILE:1,2,3 Trifluorbenzol.ngb-sd7
#FORMAT:NETZSCH5
#FTYPE:ANSI
#IDENTITY:1,2,3 Trifluorbenzol
#DECIMAL:POINT
#SEPARATOR:SEMICOLON
#MTYPE:DSC
#INSTRUMENT:NETZSCH DSC 204F1 Phoenix
#PROJECT:
#DATE/TIME:08.06.2021 10:41
#CORR. FILE:
#TEMPCAL:09-02-2021 12:34
#SENSITIVITY:02-06-2021 08:41
#LABORATORY:Messlabor 00036
#OPERATOR:A.Warmbold
#REMARK:
#SAMPLE:1,2,3 Trifluorbenzol
#SAMPLE MASS /mg:17.8
#MATERIAL:1,2,3 Trifluorbenzol
#REFERENCE:
#REFERENCE MASS /mg:0
#TYPE OF CRUCIBLE:Low pressure Al, closed
#SAMPLE CRUCIBLE MASS /mg:0
#REFERENCE CRUCIBLE MASS /mg:0
#M.RANGE /µV:5000
#TAU-R:---
#CORR. CODE:000
#EXO:-1
#RANGE:-30°C....25°C/-10.0....0.5K/min
#SEGMENT:S1-4/4
#SEG. 1:25°C/10.0(K/min)/-30°C
#SEG. 2:-30°C/00:05/-30°C
#SEG. 3:-30°C/0.5(K/min)/10°C
#SEG. 4:10°C/00:05/10°C

##Temp./°C; Time/min; DSC/(mW/mg); Gas Flow(purge2)/(ml/min); Gas Flow(protective)/(ml/min); Sensit./(uV/mW); Segment

24.952	0	0.02569	40	40	3.48305	1
23.71585	0.5	−0.05884	40	40	3.48333	1
21.4443	1	−0.08887	40	40	3.4838	1
18.75436	1.5	−0.10928	40	40	3.48429	1
15.62397	2	−0.1281	40	40	3.48476	1
11.93138	2.5	−0.15485	40	40	3.48516	1
7.6011	3	−0.18166	40	40	3.48544	1
2.56589	3.5	−0.21338	40	40	3.48549	1
−3.27413	4	−0.24552	40	40	3.4852	1
−9.83061	4.5	−0.2731	40	40	3.48441	1
−17.129875	5	−0.29948	40	40	3.48296	1
−25.0327	5.5	−2.89703	40	40	3.48071	2
−33.155256	6	−0.43489	40	40	3.47768	2
−41.4686	6.5	−0.28682	40	40	3.4738	2
−48.403827	7	−0.17388	40	40	3.46999	2
−46.15154	7.5	0.16173	40	40	3.47128	2
−42.56735	8	0.17447	40	40	3.47323	2
−39.59196	8.5	0.14624	40	40	3.47474	2
−37.29337	9	0.11779	40	40	3.47584	2
−35.54658	9.5	0.09556	40	40	3.47664	2
−34.22928	10	0.07825	40	40	3.47722	2
−33.22216	10.5	0.06537	40	40	3.47765	3
−32.32959	11	0.06351	40	40	3.47802	3
−31.52602	11.5	0.06024	40	40	3.47834	3
−30.87762	12	0.05373	40	40	3.4786	3
−30.33896	12.5	0.04867	40	40	3.47881	3
−29.77171	13	0.04509	40	40	3.47903	3
−29.26986	13.5	0.042	40	40	3.47922	3

−28.83098	14	0.04027	40	40	3.47938	3
−28.4394	14.5	0.03841	40	40	3.47953	3
−28.08304	15	0.03708	40	40	3.47966	3
−27.75314	15.5	0.03625	40	40	3.47978	3
−27.44258	16	0.03545	40	40	3.47989	3
−27.14698	16.5	0.03522	40	40	3.47999	3
−26.86222	17	0.03458	40	40	3.48009	3
−26.58571	17.5	0.03439	40	40	3.48019	3
−26.31541	18	0.03385	40	40	3.48028	3
−26.05022	18.5	0.0338	40	40	3.48037	3
−25.78876	19	0.03364	40	40	3.48046	3
−25.52989	19.5	0.03348	40	40	3.48055	3
−25.27345	20	0.03316	40	40	3.48063	3
−25.01854	20.5	0.03313	40	40	3.48072	3
−24.76479	21	0.0332	40	40	3.4808	3
−24.51209	21.5	0.03308	40	40	3.48088	3
−24.26014	22	0.03285	40	40	3.48096	3
−24.00854	22.5	0.03306	40	40	3.48104	3
−23.75713	23	0.03284	40	40	3.48112	3
−23.50574	23.5	0.03302	40	40	3.4812	3
−23.25487	24	0.03286	40	40	3.48128	3
−23.0044	24.5	0.03285	40	40	3.48136	3
−22.75449	25	0.03321	40	40	3.48143	3
−22.50439	25.5	0.03305	40	40	3.48151	3
−22.25434	26	0.03283	40	40	3.48158	3
−22.00406	26.5	0.03295	40	40	3.48166	3
−21.75407	27	0.03323	40	40	3.48173	3
−21.50441	27.5	0.03304	40	40	3.4818	3
−21.25446	28	0.03326	40	40	3.48187	3
−21.00409	28.5	0.03312	40	40	3.48195	3
−20.75335	29	0.03349	40	40	3.48202	3
−20.50297	29.5	0.03349	40	40	3.48209	3
−20.2528	30	0.03357	40	40	3.48216	3
−20.00266	30.5	0.034	40	40	3.48222	3
−19.75243	31	0.03414	40	40	3.48229	3
−19.50237	31.5	0.03431	40	40	3.48236	3
−19.25236	32	0.03501	40	40	3.48242	3
−19.00214	32.5	0.0354	40	40	3.48249	3
−18.75222	33	0.03601	40	40	3.48256	3
−18.5023	33.5	0.03693	40	40	3.48262	3
−18.25224	34	0.03805	40	40	3.48268	3
−18.0022	34.5	0.03947	40	40	3.48275	3
−17.75222	35	0.04179	40	40	3.48281	3
−17.50216	35.5	0.04511	40	40	3.48287	3
−17.25221	36	0.05028	40	40	3.48293	3
−17.00243	36.5	0.05779	40	40	3.48299	3
−16.75258	37	0.07032	40	40	3.48305	3
−16.50278	37.5	0.091	40	40	3.48311	3
−16.2529	38	0.12478	40	40	3.48317	3
−16.00352	38.5	0.17526	40	40	3.48322	3
−15.75408	39	0.24636	40	40	3.48328	3
−15.50419	39.5	0.33696	40	40	3.48333	3
−15.25348	40	0.44276	40	40	3.48339	3
−15.00284	40.5	0.5568	40	40	3.48344	3
−14.75241	41	0.66307	40	40	3.4835	3
−14.50197	41.5	0.11269	40	40	3.48355	3
−14.25159	42	0.03337	40	40	3.4836	3
−14.00095	42.5	0.03116	40	40	3.48365	3
−13.75049	43	0.0312	40	40	3.48371	3
−13.50034	43.5	0.03131	40	40	3.48376	3
−13.25108	44	0.03114	40	40	3.4838	3
−13.0016	44.5	0.0309	40	40	3.48385	3
−12.75207	45	0.03125	40	40	3.4839	3
−12.50215	45.5	0.03097	40	40	3.48395	3
−12.25227	46	0.03097	40	40	3.48399	3
−12.00257	46.5	0.03102	40	40	3.48404	3
−11.75236	47	0.03079	40	40	3.48409	3
−11.50229	47.5	0.03082	40	40	3.48413	3
−11.25189	48	0.03079	40	40	3.48417	3
−11.00192	48.5	0.03067	40	40	3.48422	3
−10.75205	49	0.03068	40	40	3.48426	3

−10.50244	49.5	0.03066	40	40	3.4843	3
−10.25272	50	0.03054	40	40	3.48434	3
−10.00239	50.5	0.03057	40	40	3.48438	3
−9.7524	51	0.03046	40	40	3.48442	3
−9.50212	51.5	0.0306	40	40	3.48446	3
−9.25204	52	0.03073	40	40	3.4845	3
−9.00159	52.5	0.03032	40	40	3.48454	3
−8.75089	53	0.03043	40	40	3.48457	3
−8.50034	53.5	0.03044	40	40	3.48461	3
−8.25002	54	0.03038	40	40	3.48464	3
−7.99993	54.5	0.03063	40	40	3.48468	3
−7.75016	55	0.03068	40	40	3.48471	3
−7.5004	55.5	0.03046	40	40	3.48475	3
−7.25069	56	0.03057	40	40	3.48478	3
−7.00103	56.5	0.03035	40	40	3.48481	3
−6.75157	57	0.03053	40	40	3.48484	3
−6.50189	57.5	0.03053	40	40	3.48487	3
−6.25193	58	0.03034	40	40	3.4849	3
−6.00225	58.5	0.03036	40	40	3.48493	3
−5.75224	59	0.03043	40	40	3.48496	3
−5.50228	59.5	0.03042	40	40	3.48499	3
−5.25245	60	0.0304	40	40	3.48501	3
−5.00241	60.5	0.03035	40	40	3.48504	3
−4.75236	61	0.03012	40	40	3.48506	3
−4.50239	61.5	0.03009	40	40	3.48509	3
−4.25242	62	0.03007	40	40	3.48511	3
−4.00248	62.5	0.03003	40	40	3.48514	3
−3.75248	63	0.03017	40	40	3.48516	3
−3.50234	63.5	0.03017	40	40	3.48518	3
−3.25221	64	0.02999	40	40	3.4852	3
−3.0022	64.5	0.03001	40	40	3.48522	3
−2.75216	65	0.03005	40	40	3.48524	3
−2.50216	65.5	0.02991	40	40	3.48526	3
−2.2522	66	0.02986	40	40	3.48528	3
−2.00216	66.5	0.0297	40	40	3.4853	3
−1.75207	67	0.02966	40	40	3.48531	3
−1.50214	67.5	0.02983	40	40	3.48533	3
−1.25215	68	0.02971	40	40	3.48535	3
−1.0023	68.5	0.02966	40	40	3.48536	3
−0.75237	69	0.02966	40	40	3.48537	3
−0.50237	69.5	0.02936	40	40	3.48539	3
−0.25226	70	0.02951	40	40	3.4854	3
−0.00225	70.5	0.02962	40	40	3.48541	3
0.24773	71	0.02935	40	40	3.48542	3
0.49761	71.5	0.02944	40	40	3.48543	3
0.74731	72	0.02942	40	40	3.48544	3
0.9974	72.5	0.02937	40	40	3.48545	3
1.24719	73	0.02932	40	40	3.48546	3
1.49703	73.5	0.02917	40	40	3.48547	3
1.74692	74	0.02931	40	40	3.48548	3
1.99686	74.5	0.0293	40	40	3.48548	3
2.24674	75	0.02914	40	40	3.48549	3
2.49681	75.5	0.02934	40	40	3.48549	3
2.74704	76	0.02905	40	40	3.4855	3
2.99708	76.5	0.02917	40	40	3.4855	3
3.24721	77	0.02917	40	40	3.4855	3
3.49733	77.5	0.02886	40	40	3.48551	3
3.74746	78	0.02902	40	40	3.48551	3
3.99756	78.5	0.02909	40	40	3.48551	3
4.24795	79	0.02887	40	40	3.48551	3
4.4981	79.5	0.0291	40	40	3.48551	3
4.74785	80	0.02894	40	40	3.48551	3
4.99768	80.5	0.02901	40	40	3.4855	3
5.24743	81	0.02902	40	40	3.4855	3
5.49734	81.5	0.02867	40	40	3.4855	3
5.74695	82	0.02884	40	40	3.48549	3
5.99651	82.5	0.02904	40	40	3.48549	3
6.24612	83	0.02866	40	40	3.48548	3
6.49597	83.5	0.02892	40	40	3.48548	3
6.74628	84	0.02895	40	40	3.48547	3
6.99665	84.5	0.02878	40	40	3.48546	3

7.24685	85	0.0289	40	40	3.48545	3
7.49707	85.5	0.02863	40	40	3.48544	3
7.74737	86	0.02882	40	40	3.48543	3
7.99778	86.5	0.02867	40	40	3.48542	3
8.248	87	0.02859	40	40	3.48541	3
8.49788	87.5	0.02877	40	40	3.4854	3
8.74773	88	0.02859	40	40	3.48539	3
8.99761	88.5	0.02858	40	40	3.48537	3
9.2476	89	0.02859	40	40	3.48536	3
9.49757	89.5	0.02843	40	40	3.48534	3
9.74741	90	0.0285	40	40	3.48533	3
9.99782	90.5	0.02831	40	40	3.48531	4
10.1397	91	0.01973	40	40	3.4853	4
10.06811	91.5	0.01405	40	40	3.48531	4
10.02135	92	0.01536	40	40	3.48531	4
9.99917	92.5	0.01657	40	40	3.48531	4
9.99173	93	0.01696	40	40	3.48531	4
9.98676	93.5	0.01698	40	40	3.48531	4
9.99253	94	0.01734	40	40	3.48531	4
9.99765	94.5	0.01752	40	40	3.48531	4
9.99547	95	0.01708	40	40	3.48531	4

8.2 DSC Diagram

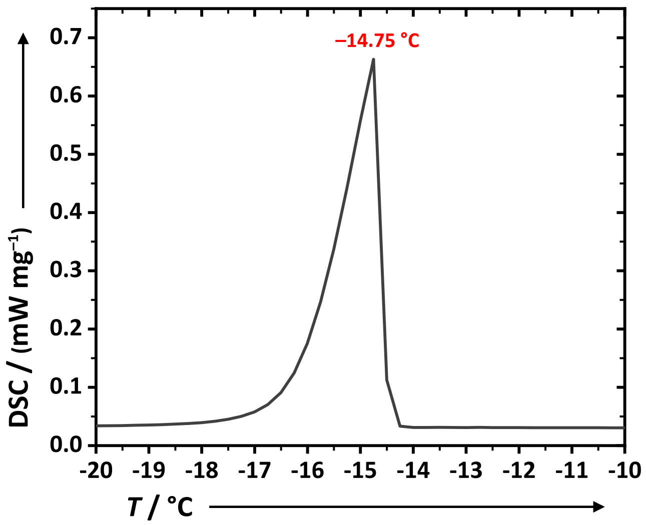

Supplementary Figure 122: DSC diagram of neat 1,2,3-Trifluorobenzene (3FB) shown in a temperature range of –20 °C up to –10 °C. Melting point of the solvent is highlighted in red**.**

9 Supplementary Note 9

The measurements were performed using a Ubbelohde-Viscometer, type no. 501 00 and capillary no. 0 for the determination of kinematic viscosity in accordance with DIN 51 562, part 1 and a device constant of K = 0.001048 $mm^2\ s^{-2}$ at a temperature of 23.1±0.2 °C. The lead time [s] through the Ubberlohde-viscometer of each neat solvent (1FB, 2FB, 3FB, 4FB, 5FB and 6FB) were measured five times and is given with an error of two seconds in Supplementary Table 38.

Supplementary Table 38: Lead time of 1FB, 2FB, 3FB, 4FB, 5FB and 6FB in seconds, together with the mean value of the lead time for each solvent. The error for each lead time is given with 2 seconds, the error for the mean value of the lead time is calculated according to Gaussian error propagation.

	lead time / s					
**#**	**1FB**	**2FB**	**3FB**	**4FB**	**5FB**	**6FB**
1	529 ± 2	512 ± 2	515 ± 2	533 ± 2	516 ± 2	533 ± 2
2	529 ± 2	513 ± 2	514 ± 2	532 ± 2	515 ± 2	534 ± 2
3	528 ± 2	513 ± 2	513 ± 2	533 ± 2	517 ± 2	535 ± 2
4	529 ± 2	514 ± 2	515 ± 2	533 ± 2	516 ± 2	533 ± 2
5	528 ± 2	514 ± 2	514 ± 2	534 ± 2	514 ± 2	533 ± 2
$\bar{t}$/ s	528.6 ± 0.9	513.2 ± 0.9	514.2 ± 0.9	533.0 ± 0.9	515.6 ± 0.9	533.6 ± 0.9

The mean value of the lead time for each solvent was used together with the device constant K for the calculation of the kinematic viscosity ν [$mm^2\ s^{-1}$] of each fluoroarene using equation (S11).

$$\nu = K \cdot \bar{t} \qquad \text{(S11)}$$

The kinematic viscosity ν [$mm^2\ s^{-1}$] was then used for the calculation of the dynamic viscosity η [mPa s] (eq. (S12)) together with the corresponding density ρ for each solvent.

$$\eta = \nu \cdot \rho \qquad \text{(S12)}$$

Due to the lack of density values for the measurement temperature, the density value of each solvent was used, with the closest temperature compared to the measurement temperature. We are aware that, this procedure leads to a systematic error of the dynamic viscosity, whereby it should be noted that this error can be neglected compared to the time error of the lead time and the temperature error. Nevertheless, the absolute values of the dynamic viscosity η should be taken under significant consideration, but the pattern of the dynamic viscosity from 1FB to 6FB should be able to show the right development. The results for the calculation of the kinematic and dynamic viscosity, together with the used density for each solvent can be found in Supplementary Table 39 below. The error for the kinematic and dynamic viscosity are calculated according to the *Gaussian* error propagation.

Supplementary Table 39: Summarized values for the kinematic and dynamic viscosity, including the corresponding errors together with the used density and the corresponding temperature for the density.

	**1FB**	**2FB**	**3FB**	**4FB**	**5FB**	**6FB**
ν / mm^2 s^{-1}	0.5540	0.5378	0.5389	0.5586	0.5403	0.5592
s_ν / mm^2 s^{-1}	0.0009	0.0009	0.0009	0.0009	0.0009	0.0009
η / mPa s	0.5662	0.6217	0.6962	0.7826	0.8165	0.8982
s_η / mPa s	0.0010	0.0011	0.0012	0.0013	0.0014	0.0015
ρ / g cm^{-3}	1.022[83]	1.156[83]	1.292[83]	1.401[88]	1.511[83]	1.6062[89]
T_ρ / °C	23.1	23.1	22.9	23.0	23.8	25.0

The summarized result in the table above shows for the dynamic viscosity of the used fluoroarenes a significant increase of the values from 1FB up to 6FB. The different values for the kinematic viscosity show in contrast no trend through the solvents from 1FB up to 6FB.

10 Supplementary Note 10

10.1 (RI-)BP86(D3BJ)/def2-TZVPP level of theory

10.1.1 Solvent structures

Fluorobenzene (1FB)

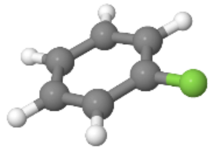

```
Method: (RI-)BP86(D3BJ)/def2-TZVPP
Symmetry: c2v

Cartesian coordinates in Ångström:
C          1.20932        0.00000        0.72050
C          0.00000        0.00000        1.42085
C         -1.20932        0.00000        0.72050
C         -1.21919        0.00000       -0.67668
C          0.00000        0.00000       -1.34668
C          1.21919        0.00000       -0.67668
F          0.00000        0.00000       -2.70609
H          2.15507        0.00000        1.26190
H          0.00000        0.00000        2.51006
H         -2.15507        0.00000        1.26190
H         -2.14787        0.00000       -1.24480
H          2.14787        0.00000       -1.24480

SCF energy GEOOPT =  -331.6471294505 H
ZPE =   235.4 kJ/mol
FREEH energy =   249.34 kJ/mol
FREEH entropy =   0.30411 kJ/mol

$vibrational spectrum
#  mode     symmetry     wave number   IR intensity    selection rules
#                         cm**(-1)        km/mol         IR     RAMAN
     1                       -0.00         0.00000        -       -
     2                       -0.00         0.00000        -       -
     3                       -0.00         0.00000        -       -
     4                       -0.00         0.00000        -       -
     5                        0.00         0.00000        -       -
     6                        0.00         0.00000        -       -
     7        b2            227.76         0.02777       YES     YES
     8        b1            394.16         1.70365       YES     YES
     9        a2            409.04         0.00000       NO      YES
    10        b2            492.57        11.20771       YES     YES
    11        a1            510.30         5.05547       YES     YES
    12        b1            608.37         0.18874       YES     YES
    13        b2            675.16        18.02908       YES     YES
    14        b2            741.05        66.87331       YES     YES
    15        a2            800.81         0.00000       NO      YES
    16        a1            803.84        26.01419       YES     YES
    17        b2            874.70         6.53201       YES     YES
    18        a2            936.78         0.00000       NO      YES
    19        b2            951.32         0.01544       YES     YES
    20        a1            996.39         0.00086       YES     YES
    21        a1           1014.80         3.63085       YES     YES
    22        b1           1062.96         7.69192       YES     YES
    23        a1           1142.65        14.93704       YES     YES
    24        b1           1148.12         0.14816       YES     YES
    25        a1           1211.92        97.60907       YES     YES
    26        b1           1289.16         0.69698       YES     YES
    27        b1           1343.93         0.04303       YES     YES
    28        b1           1446.63         0.91522       YES     YES
    29        a1           1481.10        77.85124       YES     YES
    30        a1           1589.24        44.50453       YES     YES
    31        b1           1598.42         8.73755       YES     YES
```

32	a1	3104.17	0.24493	YES	YES
33	b1	3112.49	8.97261	YES	YES
34	a1	3125.50	16.23399	YES	YES
35	b1	3134.21	5.00542	YES	YES
36	a1	3136.18	0.15981	YES	YES

$end

1,2-difluorobenzene (2FB)

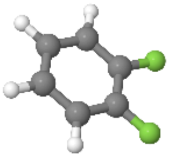

Method: (RI-)BP86(D3BJ)/def2-TZVPP
Symmetry: c2v

Cartesian coordinates in Ångström:

C	0.69820	0.00000	1.24912
C	-0.69820	0.00000	1.24912
C	-1.40249	0.00000	0.04179
C	-0.69888	0.00000	-1.15669
C	0.69888	0.00000	-1.15669
C	1.40249	0.00000	0.04179
F	1.35794	0.00000	-2.33562
F	-1.35794	0.00000	-2.33562
H	1.24685	0.00000	2.18970
H	-1.24685	0.00000	2.18970
H	-2.49103	0.00000	0.01181
H	2.49103	0.00000	0.01181

SCF energy GEOOPT = -430.9297538055 H
ZPE = 214.5 kJ/mol
FREEH energy = 230.60 kJ/mol
FREEH entropy = 0.32344 kJ/mol

$vibrational spectrum

# mode	symmetry	wave number cm**(-1)	IR intensity km/mol	selection rules IR	RAMAN
1		0.00	0.00000	-	-
2		0.00	0.00000	-	-
3		0.00	0.00000	-	-
4		0.00	0.00000	-	-
5		0.00	0.00000	-	-
6		0.00	0.00000	-	-
7	a2	180.09	0.00000	NO	YES
8	a1	278.05	0.17651	YES	YES
9	b2	281.79	0.08666	YES	YES
10	b1	430.03	0.04997	YES	YES
11	b2	445.25	3.94483	YES	YES
12	b1	535.67	3.09141	YES	YES
13	a2	546.11	0.00000	NO	YES
14	a1	565.15	4.19109	YES	YES
15	a2	672.10	0.00000	NO	YES
16	b2	736.47	77.94351	YES	YES
17	a1	755.62	31.60411	YES	YES
18	a2	816.59	0.00000	NO	YES
19	b1	839.09	18.72235	YES	YES
20	b2	904.97	4.29070	YES	YES
21	a2	929.71	0.00000	NO	YES
22	a1	1022.81	6.12954	YES	YES
23	b1	1092.84	23.02551	YES	YES
24	a1	1143.41	1.81188	YES	YES
25	b1	1184.06	37.74667	YES	YES
26	b1	1248.15	3.34025	YES	YES
27	a1	1258.83	137.62549	YES	YES
28	a1	1342.65	0.60567	YES	YES
29	b1	1447.70	10.12263	YES	YES
30	a1	1495.08	181.23140	YES	YES
31	b1	1593.62	8.36582	YES	YES
32	a1	1599.58	19.47433	YES	YES

```
    33        b1           3113.75          0.95386       YES     YES
    34        a1           3124.70          8.25162       YES     YES
    35        b1           3132.76          3.35472       YES     YES
    36        a1           3138.67          2.01443       YES     YES
$end
```

1,2,3-trifluorobenzene (3FB)

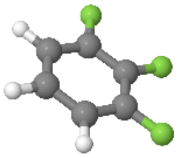

```
Method: (RI-)BP86(D3BJ)/def2-TZVPP
Symmetry: c2v

Cartesian coordinates in Ångström:
C          0.00000        0.00000        1.43848
C         -1.21481        0.00000        0.74970
C         -1.20234        0.00000       -0.64085
C          0.00000        0.00000       -1.35317
C          1.20234        0.00000       -0.64085
C          1.21481        0.00000        0.74970
F          2.35831        0.00000       -1.33591
F          0.00000        0.00000       -2.69597
F         -2.35831        0.00000       -1.33591
H          0.00000        0.00000        2.52692
H         -2.17114        0.00000        1.26887
H          2.17114        0.00000        1.26887

SCF energy GEOOPT =  -530.2114794915 H
ZPE =   193.6 kJ/mol
FREEH energy =   212.07 kJ/mol
FREEH entropy =   0.34270 kJ/mol

$vibrational spectrum
#  mode     symmetry     wave number   IR intensity    selection rules
#                         cm**(-1)        km/mol         IR     RAMAN
     1                       -0.00          0.00000        -       -
     2                       -0.00          0.00000        -       -
     3                        0.00          0.00000        -       -
     4                        0.00          0.00000        -       -
     5                        0.00          0.00000        -       -
     6                        0.00          0.00000        -       -
     7        b2            146.27          0.14572       YES     YES
     8        a2            243.81          0.00000       NO      YES
     9        b1            269.59          0.02745       YES     YES
    10        b2            289.70          0.19666       YES     YES
    11        a1            296.47          1.10526       YES     YES
    12        a1            469.80          0.11972       YES     YES
    13        b1            492.39          1.97884       YES     YES
    14        b2            529.21          0.11030       YES     YES
    15        a2            559.31          0.00000       NO      YES
    16        b1            563.63          2.76855       YES     YES
    17        b2            664.73          7.39693       YES     YES
    18        a1            683.60         21.72939       YES     YES
    19        b2            740.76         61.03888       YES     YES
    20        a1            816.14         11.31634       YES     YES
    21        a2            846.18          0.00000       NO      YES
    22        b2            911.60          0.42876       YES     YES
    23        b1           1005.29        136.97027       YES     YES
    24        a1           1053.94         10.33716       YES     YES
    25        b1           1144.03          2.04140       YES     YES
    26        a1           1211.42         21.11008       YES     YES
    27        b1           1230.22         42.44505       YES     YES
    28        a1           1287.79         95.05366       YES     YES
    29        b1           1341.52          0.45311       YES     YES
    30        b1           1467.38         85.87368       YES     YES
    31        a1           1497.19        196.87643       YES     YES
    32        b1           1596.62         71.47666       YES     YES
    33        a1           1601.87          0.10039       YES     YES
```

```
    34        a1           3124.52          3.47522       YES      YES
    35        b1           3139.07          1.03699       YES      YES
    36        a1           3145.13          0.20060       YES      YES
$end
```

1,2,3,4-tetrafluorobenzene (4FB)

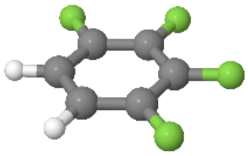

```
Method: (RI-)BP86(D3BJ)/def2-TZVPP
Symmetry: c2v

Cartesian coordinates in Ångström:
C         -0.69876        0.00000        1.25327
C         -1.38629        0.00000        0.04648
C         -0.69855        0.00000       -1.17106
C          0.69855        0.00000       -1.17106
C          1.38629        0.00000        0.04648
C          0.69876        0.00000        1.25327
F          2.73489        0.00000        0.02242
F          1.36502        0.00000       -2.33477
F         -1.36502        0.00000       -2.33477
F         -2.73489        0.00000        0.02242
H         -1.26254        0.00000        2.18365
H          1.26254        0.00000        2.18365

SCF energy GEOOPT =  -629.4915925303 H
ZPE =   172.7 kJ/mol
FREEH energy =   193.55 kJ/mol
FREEH entropy =   0.36206 kJ/mol

$vibrational spectrum
#  mode     symmetry     wave number   IR intensity    selection rules
#                         cm**(-1)        km/mol         IR     RAMAN
     1                       0.00          0.00000        -        -
     2                       0.00          0.00000        -        -
     3                       0.00          0.00000        -        -
     4                       0.00          0.00000        -        -
     5                       0.00          0.00000        -        -
     6                       0.00          0.00000        -        -
     7        a2            148.94          0.00000       NO       YES
     8        b2            149.44          1.10365       YES      YES
     9        a1            268.29          0.03050       YES      YES
    10        b1            274.32          0.22252       YES      YES
    11        b2            276.29          0.01114       YES      YES
    12        a1            314.54          1.59486       YES      YES
    13        a2            359.49          0.00000       NO       YES
    14        a1            446.27          0.04433       YES      YES
    15        b1            474.11          0.01095       YES      YES
    16        a2            527.23          0.00000       NO       YES
    17        b2            581.49          8.17753       YES      YES
    18        b1            591.98          1.79101       YES      YES
    19        a2            672.22          0.00000       NO       YES
    20        a1            674.89         17.88031       YES      YES
    21        b1            732.82         17.51330       YES      YES
    22        b2            774.93         40.42525       YES      YES
    23        a2            877.99          0.00000       NO       YES
    24        b1            969.23        142.59030       YES      YES
    25        a1           1034.52         70.58958       YES      YES
    26        a1           1147.41         24.33165       YES      YES
    27        b1           1215.05         70.64919       YES      YES
    28        b1           1237.82         24.76064       YES      YES
    29        a1           1309.44         33.25058       YES      YES
    30        a1           1336.14          0.04583       YES      YES
    31        a1           1488.39        166.64296       YES      YES
    32        b1           1491.64        289.49291       YES      YES
    33        a1           1603.97          5.92195       YES      YES
    34        b1           1605.85         12.35615       YES      YES
```

35	b1	3137.51	0.75794	YES	YES
36	a1	3149.16	0.62620	YES	YES

$end

Pentafluorobenzene (5FB)

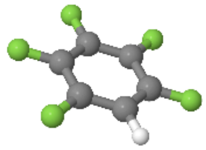

Method: (RI-)BP86(D3BJ)/def2-TZVPP
Symmetry: c2v

Cartesian coordinates in Ångström:

C	1.19662	0.00000	0.71977
C	0.00000	0.00000	1.42836
C	-1.19662	0.00000	0.71977
C	-1.21294	0.00000	-0.67669
C	0.00000	0.00000	-1.37188
C	1.21294	0.00000	-0.67669
F	0.00000	0.00000	-2.71134
F	2.37030	0.00000	1.37937
H	0.00000	0.00000	2.51570
F	-2.37030	0.00000	1.37937
F	-2.37134	0.00000	-1.35287
F	2.37134	0.00000	-1.35287

SCF energy GEOOPT = -728.7705916587 H
ZPE = 151.8 kJ/mol
FREEH energy = 175.10 kJ/mol
FREEH entropy = 0.38152 kJ/mol

$vibrational spectrum

# mode	symmetry	wave number	IR intensity	selection rules	
#		cm**(-1)	km/mol	IR	RAMAN
1		-0.00	0.00000	-	-
2		-0.00	0.00000	-	-
3		0.00	0.00000	-	-
4		0.00	0.00000	-	-
5		0.00	0.00000	-	-
6		0.00	0.00000	-	-
7	a2	126.47	0.00000	NO	YES
8	b2	151.92	0.22791	YES	YES
9	b2	197.78	1.47941	YES	YES
10	a1	263.77	0.00019	YES	YES
11	b1	267.69	0.03556	YES	YES
12	b1	295.40	0.58778	YES	YES
13	b2	307.62	0.00067	YES	YES
14	a1	318.90	1.54214	YES	YES
15	a2	372.43	0.00000	NO	YES
16	b1	425.16	0.26494	YES	YES
17	a1	462.21	0.55285	YES	YES
18	b2	540.73	3.37179	YES	YES
19	a1	566.49	0.00314	YES	YES
20	a2	642.92	0.00000	NO	YES
21	b2	666.66	0.03296	YES	YES
22	b1	673.13	2.19073	YES	YES
23	a1	704.70	21.48824	YES	YES
24	b2	795.01	27.39809	YES	YES
25	b1	936.76	168.33054	YES	YES
26	a1	1053.95	161.11038	YES	YES
27	b1	1115.86	15.43069	YES	YES
28	b1	1163.55	71.77848	YES	YES
29	a1	1252.25	13.96395	YES	YES
30	b1	1328.59	3.20905	YES	YES
31	a1	1382.72	3.76836	YES	YES
32	a1	1482.32	174.57829	YES	YES
33	b1	1502.00	407.47000	YES	YES
34	a1	1611.12	34.10140	YES	YES
35	b1	1612.72	6.11433	YES	YES

```
    36        a1           3154.21         4.61115        YES      YES
$end
```

Dichloromethane (DCM)

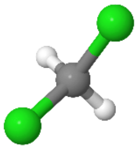

```
Method: (RI-)BP86(D3BJ)/def2-TZVPP
Symmetry: c2v

Cartesian coordinates in Ångström:
C          0.00000         0.00000         0.14914
Cl         0.00000        -1.48892        -0.83424
Cl         0.00000         1.48892        -0.83424
H         -0.90458         0.00000         0.75967
H          0.90458         0.00000         0.75967

SCF energy GEOOPT =  -959.8604993009 H
ZPE =   74.53 kJ/mol
FREEH energy =    84.05 kJ/mol
FREEH entropy =   0.27153 kJ/mol

$vibrational spectrum
#  mode     symmetry     wave number   IR intensity    selection rules
#                         cm**(-1)        km/mol          IR      RAMAN
     1                       -0.00         0.00000         -        -
     2                        0.00         0.00000         -        -
     3                        0.00         0.00000         -        -
     4                        0.00         0.00000         -        -
     5                        0.00         0.00000         -        -
     6                        0.00         0.00000         -        -
     7        a1            270.14         0.37685        YES      YES
     8        a1            687.31        11.35957        YES      YES
     9        b2            695.55       155.65944        YES      YES
    10        b1            871.73         1.82369        YES      YES
    11        a2           1130.25         0.00000        NO       YES
    12        b2           1237.99        36.37420        YES      YES
    13        a1           1410.53         0.04215        YES      YES
    14        a1           3039.63         8.10995        YES      YES
    15        b1           3117.02         0.04152        YES      YES
$end
```

1,2-Dichloroethane (DCE)

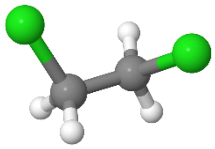

```
Method: (RI-)BP86(D3BJ)/def2-TZVPP
Symmetry: c2

Cartesian coordinates in Ångström:
C    -0.7478001   -0.1087974   -0.1308849
C     0.7478001    0.1087974   -0.1308849
Cl   -1.5644588    0.7181534    1.2430388
Cl    1.5644588   -0.7181534    1.2430388
H    -1.1767349    0.3086358   -1.0507137
H    -0.9984993   -1.1723574   -0.0617885
H     1.1767349   -0.3086358   -1.0507137
H     0.9984993    1.1723574   -0.0617885

SCF energy GEOOPT =  -999.1975286286 H
ZPE =   147.7 kJ/mol
FREEH energy =   160.40 kJ/mol
```

FREEH entropy =   0.30160 kJ/mol

$vibrational spectrum

# mode	symmetry	wave number cm**(-1)	IR intensity km/mol	selection rules IR	RAMAN
1		-0.00	0.00000	-	-
2		-0.00	0.00000	-	-
3		0.00	0.00000	-	-
4		0.00	0.00000	-	-
5		0.00	0.00000	-	-
6		0.00	0.00000	-	-
7	a	110.78	0.73285	YES	YES
8	a	253.96	0.95989	YES	YES
9	b	398.14	8.26989	YES	YES
10	a	641.79	17.29102	YES	YES
11	b	661.10	25.45349	YES	YES
12	b	863.96	18.56147	YES	YES
13	a	921.58	11.37947	YES	YES
14	a	1016.94	1.55159	YES	YES
15	b	1122.42	0.76420	YES	YES
16	a	1182.88	0.97909	YES	YES
17	b	1265.59	34.47096	YES	YES
18	a	1290.23	15.17534	YES	YES
19	b	1418.38	12.22814	YES	YES
20	a	1419.23	0.83259	YES	YES
21	b	2996.39	3.25277	YES	YES
22	a	3003.92	23.68600	YES	YES
23	a	3057.13	0.62559	YES	YES
24	b	3071.73	5.20849	YES	YES

$end

Acetonitrile (AN)

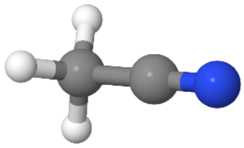

Method: (RI-)BP86(D3BJ)/def2-TZVPP
Symmetry: c3v

Cartesian coordinates in Ångström:

C	0.0000000	0.0000000	0.4884984
C	-0.0000000	0.0000000	-0.9665190
N	0.0000000	0.0000000	-2.1284481
H	-0.5147777	0.8916212	0.8688219
H	-0.5147777	-0.8916212	0.8688219
H	1.0295554	0.0000000	0.8688219

SCF energy GEOOPT = -132.8168669873 H
ZPE =   115.4 kJ/mol
FREEH energy =   124.94 kJ/mol
FREEH entropy =   0.24343 kJ/mol

$vibrational spectrum

# mode	symmetry	wave number cm**(-1)	IR intensity km/mol	selection rules IR	RAMAN
1		0.00	0.00000	-	-
2		0.00	0.00000	-	-
3		0.00	0.00000	-	-
4		0.00	0.00000	-	-
5		0.00	0.00000	-	-
6		0.00	0.00000	-	-
7	e	370.27	0.16143	YES	YES
8	e	370.27	0.16143	YES	YES
9	a1	919.33	0.42979	YES	YES
10	e	1022.07	2.03958	YES	YES
11	e	1022.07	2.03958	YES	YES
12	a1	1360.94	2.99941	YES	YES
13	e	1425.42	10.61080	YES	YES
14	e	1425.42	10.61080	YES	YES

```
    15        a1          2279.26          9.07131       YES      YES
    16        a1          2981.91          2.81399       YES      YES
    17        e           3054.17          0.66688       YES      YES
    18        e           3054.17          0.66688       YES      YES
$end
```

Dimethylformamide (DMF)

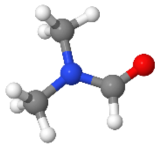

```
Method: (RI-)BP86(D3BJ)/def2-TZVPP
Symmetry: cs

Cartesian coordinates in Ångström:
H         -1.95848       -0.98784        0.00000
C         -1.52362        0.03800        0.00000
O         -2.21186        1.05059        0.00000
N         -0.15720        0.00261        0.00000
C          0.60729        1.23675        0.00000
C          0.57042       -1.24963        0.00000
H         -0.10082        2.07197        0.00000
H          1.24794        1.29674        0.89311
H          1.24794        1.29674       -0.89311
H         -0.14260       -2.08360        0.00000
H          1.21049       -1.33616       -0.89225
H          1.21049       -1.33616        0.89225

SCF energy GEOOPT =  -248.6415531155 H
ZPE =   260.2 kJ/mol
FREEH energy =   276.40 kJ/mol
FREEH entropy =   0.31903 kJ/mol

$vibrational spectrum
#  mode     symmetry     wave number   IR intensity    selection rules
#                         cm**(-1)        km/mol         IR     RAMAN
     1                      0.00         0.00000         -        -
     2                      0.00         0.00000         -        -
     3                      0.00         0.00000         -        -
     4                      0.00         0.00000         -        -
     5                      0.00         0.00000         -        -
     6                      0.00         0.00000         -        -
     7        a"          131.44         0.19316       YES      YES
     8        a"          176.85         0.06341       YES      YES
     9        a"          229.47         1.13664       YES      YES
    10        a'          308.71        11.24174       YES      YES
    11        a"          336.52        12.85035       YES      YES
    12        a'          380.59         1.73175       YES      YES
    13        a'          643.06         6.36606       YES      YES
    14        a'          855.04         1.15892       YES      YES
    15        a"          968.11         0.42816       YES      YES
    16        a'         1043.57         2.04996       YES      YES
    17        a'         1061.70       103.75051       YES      YES
    18        a"         1090.22         0.14943       YES      YES
    19        a"         1137.02         2.07381       YES      YES
    20        a'         1242.73        25.37435       YES      YES
    21        a'         1363.58        54.32724       YES      YES
    22        a'         1377.81        20.86254       YES      YES
    23        a'         1385.06         8.28948       YES      YES
    24        a'         1414.36         5.33718       YES      YES
    25        a"         1427.63         3.52253       YES      YES
    26        a"         1449.61        14.61509       YES      YES
    27        a'         1454.31        12.59228       YES      YES
    28        a'         1492.95        18.27102       YES      YES
    29        a'         1707.73       413.75570       YES      YES
    30        a'         2847.73        98.48802       YES      YES
    31        a'         2937.58        53.12349       YES      YES
    32        a'         2947.88        57.13634       YES      YES
    33        a"         2983.24        45.60881       YES      YES
```

34	a"	2996.92	18.20498	YES	YES
35	a'	3042.96	13.96798	YES	YES
36	a'	3073.09	1.60063	YES	YES

$end

Tetrahydrofuran (THF)

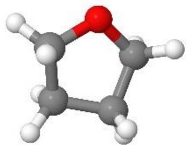

Method: (RI-)BP86(D3BJ)/def2-TZVPP
Symmetry: c2

Cartesian coordinates in Ångström:

C	1.1750836	0.0606725	0.6658763
C	0.7170222	-0.2745276	-0.7554096
C	-0.7170222	0.2745276	-0.7554096
C	-1.1750836	-0.0606725	0.6658763
O	0.0000000	0.0000000	1.4974442
H	1.6041703	1.0775627	0.7162346
H	1.9180971	-0.6468399	1.0613423
H	0.7090689	-1.3629780	-0.9108266
H	1.3566251	0.1760954	-1.5244685
H	-0.7090689	1.3629780	-0.9108266
H	-1.3566251	-0.1760954	-1.5244685
H	-1.6041703	-1.0775627	0.7162346
H	-1.9180971	0.6468399	1.0613423

SCF energy GEOOPT = -232.5645056424 H
ZPE = 296.9 kJ/mol
FREEH energy = 310.19 kJ/mol
FREEH entropy = 0.29962 kJ/mol

$vibrational spectrum

# mode	symmetry	wave number	IR intensity	selection rules	
#		cm**(-1)	km/mol	IR	RAMAN
1		-0.00	0.00000	-	-
2		0.00	0.00000	-	-
3		0.00	0.00000	-	-
4		0.00	0.00000	-	-
5		0.00	0.00000	-	-
6		0.00	0.00000	-	-
7	b	42.60	3.82107	YES	YES
8	a	244.85	0.00679	YES	YES
9	b	561.21	0.70342	YES	YES
10	a	654.81	3.24558	YES	YES
11	a	819.59	6.36403	YES	YES
12	b	855.60	9.08335	YES	YES
13	a	881.86	23.08155	YES	YES
14	b	885.92	4.62982	YES	YES
15	a	910.30	4.57872	YES	YES
16	b	942.48	0.49850	YES	YES
17	a	1010.42	3.93302	YES	YES
18	b	1043.47	104.90992	YES	YES
19	a	1131.80	0.01292	YES	YES
20	b	1142.22	3.46817	YES	YES
21	a	1150.48	6.25017	YES	YES
22	a	1219.26	0.19620	YES	YES
23	b	1222.39	6.37633	YES	YES
24	b	1273.21	0.96367	YES	YES
25	a	1295.16	0.43869	YES	YES
26	b	1319.11	0.08134	YES	YES
27	a	1349.53	2.21223	YES	YES
28	b	1440.00	3.92521	YES	YES
29	a	1448.36	4.92054	YES	YES
30	b	1472.85	0.71007	YES	YES
31	a	1484.23	0.00603	YES	YES
32	b	2910.82	150.34779	YES	YES
33	a	2916.30	2.21365	YES	YES

34	a	2973.08	21.08105	YES	YES
35	b	2977.07	4.80951	YES	YES
36	b	2991.49	111.69777	YES	YES
37	a	2992.69	0.72368	YES	YES
38	a	3032.06	17.76006	YES	YES
39	b	3040.45	44.62771	YES	YES

$end

10.1.2 $[NO(S)_1]^+$ structures

$[NO(1FB)_1]^+$

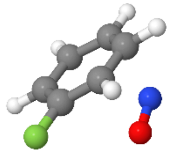

Method: (RI-)BP86(D3BJ)/def2-TZVPP
Symmetry: cs

Cartesian coordinates in Ångström:

C	0.68941	-0.19134	-1.23258
C	1.12448	-0.74654	0.00000
C	0.68941	-0.19134	1.23258
C	-0.26316	0.81286	1.23824
C	-0.74534	1.27980	0.00000
H	1.89357	-1.51762	0.00000
H	1.09004	-0.57442	2.17027
H	-0.63984	1.25457	2.15957
C	-0.26316	0.81286	-1.23824
N	-0.68344	-2.17627	0.00000
O	-1.67815	-1.67348	0.00000
H	1.09004	-0.57442	-2.17027
H	-0.63984	1.25457	-2.15957
F	-1.67055	2.22729	0.00000

SCF energy GEOOPT = -461.3363306677 H
ZPE = 254.1 kJ/mol
FREEH energy = 275.99 kJ/mol
FREEH entropy = 0.37699 kJ/mol

$vibrational spectrum

# mode	symmetry	wave number cm**(-1)	IR intensity km/mol	selection rules IR	RAMAN
1		-0.00	0.00000	-	-
2		-0.00	0.00000	-	-
3		-0.00	0.00000	-	-
4		-0.00	0.00000	-	-
5		0.00	0.00000	-	-
6		0.00	0.00000	-	-
7	a'	74.92	0.84984	YES	YES
8	a"	84.17	0.03468	YES	YES
9	a"	141.24	0.11902	YES	YES
10	a'	206.97	3.43736	YES	YES
11	a'	244.52	14.11772	YES	YES
12	a'	388.85	7.10735	YES	YES
13	a"	393.86	0.21382	YES	YES
14	a"	404.28	4.31928	YES	YES
15	a'	476.11	12.39292	YES	YES
16	a'	502.18	8.03962	YES	YES
17	a"	590.38	0.32343	YES	YES
18	a'	665.57	10.04942	YES	YES
19	a'	804.98	22.61768	YES	YES
20	a'	812.69	62.79243	YES	YES
21	a"	814.97	0.19676	YES	YES
22	a'	929.96	0.00937	YES	YES
23	a"	976.71	0.01051	YES	YES
24	a'	984.06	7.89286	YES	YES
25	a'	987.94	0.40808	YES	YES
26	a'	993.11	0.40609	YES	YES

27	a"	1069.59	3.39582	YES	YES
28	a'	1148.48	19.47234	YES	YES
29	a"	1156.74	0.30082	YES	YES
30	a'	1275.43	104.14460	YES	YES
31	a"	1293.20	2.45968	YES	YES
32	a"	1367.43	3.64066	YES	YES
33	a'	1463.32	165.90294	YES	YES
34	a"	1464.04	13.02677	YES	YES
35	a"	1518.63	14.28644	YES	YES
36	a'	1577.57	92.77748	YES	YES
37	a'	1991.19	612.19455	YES	YES
38	a'	3124.87	0.05901	YES	YES
39	a"	3131.12	0.00524	YES	YES
40	a'	3137.31	4.62345	YES	YES
41	a"	3143.60	17.50625	YES	YES
42	a'	3145.20	0.03390	YES	YES

$end

$[NO(2FB)_1]^+$

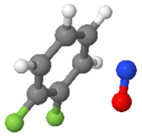

Method: (RI-)BP86(D3BJ)/def2-TZVPP
Symmetry: cs

Cartesian coordinates in Ångström:

C	-0.57227	-1.04873	0.71195
C	0.30855	-0.23175	1.42471
C	1.14649	0.62118	0.71257
C	1.14649	0.62118	-0.71257
C	0.30855	-0.23175	-1.42471
H	0.34367	-0.23015	2.51328
F	1.97750	1.43622	1.33710
F	1.97750	1.43622	-1.33710
C	-0.57227	-1.04873	-0.71195
N	-2.18715	0.65441	0.00000
O	-1.71057	1.66170	0.00000
H	-1.23683	-1.72562	1.24757
H	-1.23683	-1.72562	-1.24757
H	0.34367	-0.23015	-2.51328

SCF energy GEOOPT = -560.6148899666 H
ZPE = 232.6 kJ/mol
FREEH energy = 257.04 kJ/mol
FREEH entropy = 0.40190 kJ/mol

$vibrational spectrum

# mode	symmetry	wave number	IR intensity	selection rules	
#		cm**(-1)	km/mol	IR	RAMAN
1		-0.00	0.00000	-	-
2		-0.00	0.00000	-	-
3		-0.00	0.00000	-	-
4		-0.00	0.00000	-	-
5		0.00	0.00000	-	-
6		0.00	0.00000	-	-
7	a"	48.45	0.06743	YES	YES
8	a'	67.92	1.68809	YES	YES
9	a"	102.68	0.00744	YES	YES
10	a"	185.69	0.25712	YES	YES
11	a'	216.74	8.19861	YES	YES
12	a'	284.24	0.62331	YES	YES
13	a'	305.97	4.13386	YES	YES
14	a'	373.60	6.01515	YES	YES
15	a"	425.30	0.07677	YES	YES
16	a'	445.75	4.72162	YES	YES
17	a"	510.54	0.30393	YES	YES
18	a"	535.84	5.12333	YES	YES
19	a'	553.23	7.41594	YES	YES

20	a"	696.30	0.43970	YES	YES
21	a'	757.19	17.49161	YES	YES
22	a'	804.45	81.50646	YES	YES
23	a"	843.65	2.78446	YES	YES
24	a"	849.66	8.88710	YES	YES
25	a'	941.21	0.50887	YES	YES
26	a"	954.94	0.03957	YES	YES
27	a'	998.89	4.90064	YES	YES
28	a"	1099.76	11.14676	YES	YES
29	a'	1152.34	7.22985	YES	YES
30	a"	1214.03	19.45646	YES	YES
31	a"	1274.40	19.35352	YES	YES
32	a'	1300.04	116.33678	YES	YES
33	a'	1369.74	5.58499	YES	YES
34	a"	1453.64	30.45467	YES	YES
35	a'	1489.13	371.00209	YES	YES
36	a'	1532.54	42.37620	YES	YES
37	a"	1561.50	13.26104	YES	YES
38	a'	1991.28	667.82279	YES	YES
39	a"	3125.87	0.03771	YES	YES
40	a'	3133.47	1.88846	YES	YES
41	a"	3140.77	22.28799	YES	YES
42	a'	3143.74	4.82776	YES	YES

$end

$[NO(3FB)_1]^+$

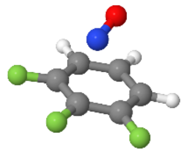

Method: (RI-)BP86(D3BJ)/def2-TZVPP
Symmetry: cs

Cartesian coordinates in Ångström:

C	-0.18624	-0.65986	-1.23089
C	-0.59746	0.66425	-1.23002
C	-0.77765	1.31785	0.00000
C	-0.59746	0.66425	1.23002
C	-0.18624	-0.65986	1.23089
C	0.07454	-1.33068	0.00000
F	0.45367	-2.58874	0.00000
F	-0.00222	-1.33879	-2.35209
H	-0.78083	1.16900	-2.17720
H	-1.10072	2.35801	0.00000
H	-0.78083	1.16900	2.17720
F	-0.00222	-1.33879	2.35209
N	2.18970	-0.26733	0.00000
O	2.29397	0.84171	0.00000

SCF energy GEOOPT = -659.8907433019 H
ZPE = 211.3 kJ/mol
FREEH energy = 238.36 kJ/mol
FREEH entropy = 0.42284 kJ/mol

$vibrational spectrum

# mode	symmetry	wave number cm**(-1)	IR intensity km/mol	selection rules IR	RAMAN
1		-0.00	0.00000	-	-
2		-0.00	0.00000	-	-
3		0.00	0.00000	-	-
4		0.00	0.00000	-	-
5		0.00	0.00000	-	-
6		0.00	0.00000	-	-
7	a"	35.50	1.15319	YES	YES
8	a'	87.23	1.45823	YES	YES
9	a"	97.30	0.00044	YES	YES
10	a'	140.87	0.79787	YES	YES
11	a'	165.11	9.59512	YES	YES
12	a"	239.99	0.01630	YES	YES

13	a"	273.71	0.10642	YES	YES
14	a'	296.21	3.64880	YES	YES
15	a'	307.83	0.47545	YES	YES
16	a'	395.11	17.29486	YES	YES
17	a'	462.89	1.47464	YES	YES
18	a"	485.58	5.53186	YES	YES
19	a'	506.74	6.01566	YES	YES
20	a"	559.58	3.15643	YES	YES
21	a"	568.03	2.28983	YES	YES
22	a'	673.23	2.42822	YES	YES
23	a'	692.63	18.64012	YES	YES
24	a'	790.00	47.71959	YES	YES
25	a'	812.77	3.86289	YES	YES
26	a"	889.52	0.02213	YES	YES
27	a'	969.26	0.26262	YES	YES
28	a"	1030.05	83.47913	YES	YES
29	a'	1044.49	2.28088	YES	YES
30	a"	1153.52	1.64272	YES	YES
31	a"	1260.89	44.56281	YES	YES
32	a'	1262.72	17.02087	YES	YES
33	a'	1317.27	45.64636	YES	YES
34	a"	1350.75	1.99572	YES	YES
35	a'	1466.58	348.92066	YES	YES
36	a"	1482.24	185.09181	YES	YES
37	a"	1525.30	82.28076	YES	YES
38	a'	1572.89	25.15130	YES	YES
39	a'	1996.40	674.44631	YES	YES
40	a'	3132.72	0.03000	YES	YES
41	a"	3142.86	13.26852	YES	YES
42	a'	3147.18	16.13190	YES	YES

$end

$[NO(4FB)_1]^+$

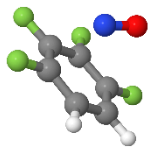

Method: (RI-)BP86(D3BJ)/def2-TZVPP
Symmetry: c1

Cartesian coordinates in Ångström:

H	-5.13616	4.08217	6.23066
C	-4.97973	3.08486	5.82219
F	-7.18480	2.47426	6.44754
C	-6.03759	2.15263	5.88053
C	-3.77435	2.70661	5.24919
C	-5.86993	0.81305	5.42584
H	-2.93604	3.39781	5.17654
F	-6.85504	-0.05410	5.54625
C	-4.65182	0.44093	4.85349
C	-3.61857	1.40174	4.75466
F	-4.46802	-0.78725	4.40937
F	-2.47736	1.02770	4.20815
N	-6.52311	2.77325	3.64585
O	-5.73726	2.68557	2.85932

SCF energy GEOOPT = -759.1688100338 H
ZPE =   190.7 kJ/mol
FREEH energy =   219.88 kJ/mol
FREEH entropy =   0.43586 kJ/mol

$vibrational spectrum

# mode	symmetry	wave number	IR intensity	selection rules	
#		cm**(-1)	km/mol	IR	RAMAN
1		-0.00	0.00000	-	-
2		-0.00	0.00000	-	-
3		-0.00	0.00000	-	-
4		0.00	0.00000	-	-
5		0.00	0.00000	-	-

6		0.00	0.00000	-	-
7	a	68.45	1.41591	YES	YES
8	a	73.36	0.35866	YES	YES
9	a	99.52	0.42068	YES	YES
10	a	139.60	3.63843	YES	YES
11	a	148.97	4.34240	YES	YES
12	a	194.78	3.89739	YES	YES
13	a	267.38	0.08451	YES	YES
14	a	276.11	0.77696	YES	YES
15	a	291.46	0.29502	YES	YES
16	a	324.11	3.92687	YES	YES
17	a	350.15	4.69402	YES	YES
18	a	390.28	6.85679	YES	YES
19	a	437.24	2.18255	YES	YES
20	a	464.18	1.84040	YES	YES
21	a	550.08	1.78183	YES	YES
22	a	592.71	7.54544	YES	YES
23	a	597.41	5.37677	YES	YES
24	a	672.73	16.49426	YES	YES
25	a	692.28	1.82299	YES	YES
26	a	739.11	11.75924	YES	YES
27	a	803.10	29.90408	YES	YES
28	a	933.31	0.04538	YES	YES
29	a	987.71	68.03587	YES	YES
30	a	1059.88	44.85864	YES	YES
31	a	1173.32	35.24186	YES	YES
32	a	1234.44	87.60571	YES	YES
33	a	1294.61	13.03153	YES	YES
34	a	1331.23	26.43875	YES	YES
35	a	1359.85	14.90167	YES	YES
36	a	1470.42	448.80424	YES	YES
37	a	1502.12	263.82149	YES	YES
38	a	1525.59	37.23391	YES	YES
39	a	1570.60	44.68169	YES	YES
40	a	1988.45	680.49694	YES	YES
41	a	3138.06	4.29649	YES	YES
42	a	3147.07	28.95136	YES	YES

$end

$[NO(5FB)_1]^+$

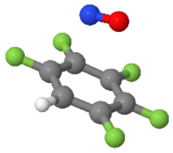

Method: (RI-)BP86(D3BJ)/def2-TZVPP
Symmetry: c1

Cartesian coordinates in Ångström:

C	-7.85561	2.41498	-0.03095
C	-7.80584	0.99457	-0.18567
C	-6.59830	0.32383	-0.38948
C	-5.42878	1.07478	-0.40741
C	-5.44492	2.48837	-0.25452
C	-6.66464	3.15903	-0.09561
F	-6.69597	4.47257	0.02627
F	-9.01573	3.02056	0.10969
F	-4.25804	0.49056	-0.58301
F	-4.31797	3.16667	-0.29325
F	-8.94985	0.33792	-0.17474
H	-6.57628	-0.75588	-0.52943
N	-7.43569	1.36817	2.21666
O	-6.37362	1.47806	2.53945

SCF energy GEOOPT = -858.4439080116 H
ZPE = 169.4 kJ/mol
FREEH energy = 201.22 kJ/mol
FREEH entropy = 0.46015 kJ/mol

$vibrational spectrum

# mode	symmetry	wave number	IR intensity	selection rules	
#		cm**(-1)	km/mol	IR	RAMAN
1		-0.00	0.00000	-	-
2		0.00	0.00000	-	-
3		0.00	0.00000	-	-
4		0.00	0.00000	-	-
5		0.00	0.00000	-	-
6		0.00	0.00000	-	-
7	a	38.99	0.31674	YES	YES
8	a	68.16	1.12989	YES	YES
9	a	97.38	0.13650	YES	YES
10	a	124.53	0.04404	YES	YES
11	a	145.58	4.17371	YES	YES
12	a	155.90	9.12436	YES	YES
13	a	233.51	0.30772	YES	YES
14	a	266.39	0.12510	YES	YES
15	a	266.93	0.06614	YES	YES
16	a	296.71	2.17620	YES	YES
17	a	312.65	0.26992	YES	YES
18	a	329.21	4.58299	YES	YES
19	a	350.80	6.18020	YES	YES
20	a	410.49	0.04390	YES	YES
21	a	444.61	6.00436	YES	YES
22	a	452.10	2.37911	YES	YES
23	a	557.31	8.16215	YES	YES
24	a	571.88	0.23311	YES	YES
25	a	631.88	1.26552	YES	YES
26	a	678.98	1.07142	YES	YES
27	a	684.32	1.45693	YES	YES
28	a	705.17	16.31444	YES	YES
29	a	828.99	20.70969	YES	YES
30	a	956.45	87.39045	YES	YES
31	a	1083.76	117.21076	YES	YES
32	a	1167.46	49.98742	YES	YES
33	a	1185.33	44.32977	YES	YES
34	a	1303.42	3.77505	YES	YES
35	a	1346.16	3.04920	YES	YES
36	a	1417.69	72.38337	YES	YES
37	a	1478.00	524.12716	YES	YES
38	a	1492.45	278.16781	YES	YES
39	a	1535.49	117.33495	YES	YES
40	a	1568.77	26.48081	YES	YES
41	a	1988.61	679.15425	YES	YES
42	a	3145.41	33.79077	YES	YES

$end

$[NO(DCM)_1]^+$

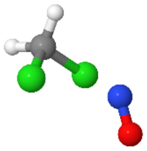

Method: (RI-)BP86(D3BJ)/def2-TZVPP
Symmetry: cs

Cartesian coordinates in Ångström:

O	1.21465	-2.46278	0.00000
N	1.31549	-1.36555	0.00000
Cl	-0.35168	-0.08018	1.46768
Cl	-0.35168	-0.08018	-1.46768
C	-0.53480	0.93621	0.00000
H	0.25087	1.69413	0.00000
H	-1.54285	1.35837	0.00000

SCF energy GEOOPT = -1089.527755817 H
ZPE = 92.66 kJ/mol
FREEH energy = 109.80 kJ/mol
FREEH entropy = 0.34587 kJ/mol

$vibrational spectrum

# mode	symmetry	wave number	IR intensity	selection rules	
#		cm**(-1)	km/mol	IR	RAMAN
1		-0.00	0.00000	-	-
2		-0.00	0.00000	-	-
3		-0.00	0.00000	-	-
4		-0.00	0.00000	-	-
5		0.00	0.00000	-	-
6		0.00	0.00000	-	-
7	a'	68.38	8.34359	YES	YES
8	a"	108.05	9.88551	YES	YES
9	a'	179.52	21.05101	YES	YES
10	a'	281.91	1.02603	YES	YES
11	a"	318.73	5.87165	YES	YES
12	a'	328.94	22.62137	YES	YES
13	a"	655.68	59.55668	YES	YES
14	a'	670.53	17.75669	YES	YES
15	a'	876.82	1.03127	YES	YES
16	a"	1114.84	0.46557	YES	YES
17	a"	1230.13	1.77514	YES	YES
18	a'	1402.44	1.26755	YES	YES
19	a'	2076.27	663.71890	YES	YES
20	a'	3043.86	8.73940	YES	YES
21	a'	3134.80	18.08112	YES	YES

$end

$[NO(DCE)_1]^+$

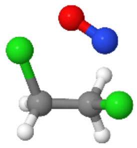

Method: (RI-)BP86(D3BJ)/def2-TZVPP
Symmetry: c1

Cartesian coordinates in Ångström:

C	-0.7724144	-0.9278683	-0.1004017
C	0.7234706	-0.8346834	-0.0226068
Cl	-1.4638320	0.5049799	-0.9503234
Cl	1.2469740	0.5928871	1.0056342
H	-1.0525436	-1.8059547	-0.6986415
H	-1.2491929	-0.9883114	0.8825716
H	1.1631878	-1.6979278	0.4962982
H	1.2038573	-0.6882000	-0.9943718
N	0.5333048	2.8692590	0.5260670
O	-0.3328117	2.9758195	-0.1442257

SCF energy GEOOPT = -1128.872817734 H
ZPE = 165.0 kJ/mol
FREEH energy = 185.68 kJ/mol
FREEH entropy = 0.37696 kJ/mol

$vibrational spectrum

# mode	symmetry	wave number	IR intensity	selection rules	
#		cm**(-1)	km/mol	IR	RAMAN
1		-0.00	0.00000	-	-
2		-0.00	0.00000	-	-
3		-0.00	0.00000	-	-
4		-0.00	0.00000	-	-
5		0.00	0.00000	-	-
6		0.00	0.00000	-	-
7	a	40.65	10.30355	YES	YES
8	a	120.62	5.79824	YES	YES
9	a	130.84	6.63872	YES	YES
10	a	144.54	1.12274	YES	YES
11	a	215.33	31.51373	YES	YES
12	a	260.64	0.29465	YES	YES
13	a	384.09	32.08146	YES	YES
14	a	413.39	1.72958	YES	YES
15	a	578.98	25.20779	YES	YES
16	a	643.16	14.34445	YES	YES

17	a	835.25	21.76327	YES	YES
18	a	918.48	8.80733	YES	YES
19	a	1006.92	0.73188	YES	YES
20	a	1106.23	0.81275	YES	YES
21	a	1173.32	0.23021	YES	YES
22	a	1239.22	3.10683	YES	YES
23	a	1288.03	14.30804	YES	YES
24	a	1404.82	18.68448	YES	YES
25	a	1413.54	8.86219	YES	YES
26	a	2084.71	601.74103	YES	YES
27	a	3002.68	17.25501	YES	YES
28	a	3007.17	12.06984	YES	YES
29	a	3074.85	5.22383	YES	YES
30	a	3092.79	4.49708	YES	YES

$end

$[NO(AN)_1]^+$

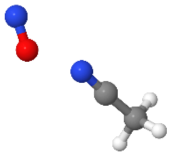

Method: (RI-)BP86(D3BJ)/def2-TZVPP
Symmetry: cs

Cartesian coordinates in Ångström:

C	-1.66125	0.01328	0.00000
C	-0.21810	0.06869	0.00000
H	-2.01293	-0.51669	-0.89602
H	-2.07300	1.03191	0.00000
H	-2.01293	-0.51669	0.89602
N	0.94156	0.16384	0.00000
O	3.14058	-0.50945	0.00000
N	3.89607	0.26511	0.00000

SCF energy GEOOPT = -262.4741228115 H
ZPE = 131.4 kJ/mol
FREEH energy = 150.31 kJ/mol
FREEH entropy = 0.36485 kJ/mol

$vibrational spectrum

# mode	symmetry	wave number	IR intensity	selection rules	
#		cm**(-1)	km/mol	IR	RAMAN
1		-0.00	0.00000	-	-
2		-0.00	0.00000	-	-
3		0.00	0.00000	-	-
4		0.00	0.00000	-	-
5		0.00	0.00000	-	-
6		0.00	0.00000	-	-
7	a"	9.57	0.65035	YES	YES
8	a"	58.98	19.02988	YES	YES
9	a'	60.63	18.15316	YES	YES
10	a'	188.28	24.78753	YES	YES
11	a'	289.22	12.39974	YES	YES
12	a"	373.53	1.39511	YES	YES
13	a'	378.49	2.60184	YES	YES
14	a'	932.33	1.29773	YES	YES
15	a"	995.86	8.39706	YES	YES
16	a'	1005.84	11.10250	YES	YES
17	a'	1348.94	11.42529	YES	YES
18	a'	1389.84	17.78672	YES	YES
19	a"	1397.91	16.36194	YES	YES
20	a'	2201.00	920.21874	YES	YES
21	a'	2251.66	48.03762	YES	YES
22	a'	2975.44	48.42415	YES	YES
23	a"	3053.35	8.72167	YES	YES
24	a'	3054.77	7.48436	YES	YES

$end

$[NO(DMF)_1]^+$

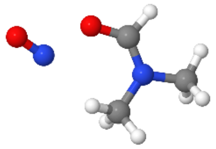

Method: (RI-)BP86(D3BJ)/def2-TZVPP
Symmetry: c1

Cartesian coordinates in Ångström:

N	-0.51978	-0.02008	-1.50678
O	-1.61536	-0.18182	-1.42539
O	-0.09532	1.25567	-0.17871
C	1.07082	1.72409	0.05341
N	2.22998	1.10505	-0.05482
C	3.47894	1.81917	0.24625
C	2.35740	-0.29271	-0.47471
H	1.10913	2.76062	0.41927
H	4.11613	1.83116	-0.64676
H	3.25801	2.84684	0.54931
H	4.00416	1.29962	1.05713
H	3.20612	-0.73681	0.05549
H	1.44584	-0.84309	-0.21713
H	2.53664	-0.35585	-1.55547

SCF energy GEOOPT = -378.3473008735 H
ZPE = 281.5 kJ/mol
FREEH energy = 304.76 kJ/mol
FREEH entropy = 0.38900 kJ/mol

$vibrational spectrum

# mode	symmetry	wave number	IR intensity	selection rules	
#		cm**(-1)	km/mol	IR	RAMAN
1		-0.00	0.00000	-	-
2		-0.00	0.00000	-	-
3		0.00	0.00000	-	-
4		0.00	0.00000	-	-
5		0.00	0.00000	-	-
6		0.00	0.00000	-	-
7	a	28.46	3.41896	YES	YES
8	a	89.86	1.83869	YES	YES
9	a	112.46	0.97114	YES	YES
10	a	177.88	0.80153	YES	YES
11	a	207.43	4.57088	YES	YES
12	a	257.45	22.37101	YES	YES
13	a	276.59	2.07270	YES	YES
14	a	327.50	13.20022	YES	YES
15	a	393.03	20.12945	YES	YES
16	a	416.13	6.22692	YES	YES
17	a	542.03	107.03590	YES	YES
18	a	680.29	7.48127	YES	YES
19	a	842.31	2.33799	YES	YES
20	a	952.95	2.92250	YES	YES
21	a	1028.88	5.94616	YES	YES
22	a	1077.89	0.93109	YES	YES
23	a	1093.90	13.03642	YES	YES
24	a	1129.66	4.56095	YES	YES
25	a	1202.41	4.27240	YES	YES
26	a	1320.16	106.18111	YES	YES
27	a	1383.09	26.07972	YES	YES
28	a	1400.00	26.00636	YES	YES
29	a	1412.97	41.60180	YES	YES
30	a	1423.86	8.56781	YES	YES
31	a	1437.59	31.78308	YES	YES
32	a	1444.98	3.29109	YES	YES
33	a	1466.01	36.69576	YES	YES
34	a	1598.29	320.69831	YES	YES
35	a	2034.75	675.33511	YES	YES
36	a	2985.90	2.85337	YES	YES
37	a	2988.17	0.45429	YES	YES
38	a	3024.47	1.14515	YES	YES

39	a	3060.84	0.56678	YES	YES
40	a	3064.90	2.66996	YES	YES
41	a	3085.05	2.89311	YES	YES
42	a	3094.67	0.67744	YES	YES

$end

10.1.3 $[NO(S)_2]^+$ structures

$[NO(1FB)_2]^+$

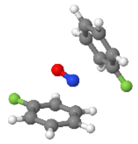

Method: (RI-)BP86(D3BJ)/def2-TZVPP
Symmetry: c1

Cartesian coordinates in Ångström:

C	-5.58258	0.50164	0.34952
C	-4.38517	0.58595	1.09816
C	-3.82780	1.84381	1.40632
C	-4.51172	3.00579	1.08189
C	-5.73686	2.88901	0.40871
H	-3.84215	-0.32225	1.35375
H	-2.86442	1.89511	1.90906
H	-4.12312	3.99566	1.31395
H	-5.12227	4.34725	5.14388
C	-5.00187	3.26734	5.22001
H	-6.83369	3.00260	6.34529
C	-5.97345	2.50400	5.90171
C	-3.86901	2.66454	4.67890
C	-5.82018	1.12616	6.02935
H	-3.08328	3.24349	4.19851
H	-6.55596	0.53336	6.57020
C	-4.72774	0.49054	5.42730
C	-3.75748	1.27974	4.77728
H	-4.57654	-0.58579	5.49626
F	-2.67947	0.66622	4.26112
C	-6.27135	1.65412	0.00728
N	-5.83714	0.72767	3.09786
O	-6.44425	1.67270	3.07735
H	-5.97228	-0.47306	0.05916
H	-7.20271	1.63023	-0.55575
F	-6.40591	4.00266	0.10476

SCF energy GEOOPT = -793.0165633090 H
ZPE = 493.9 kJ/mol
FREEH energy = 533.84 kJ/mol
FREEH entropy = 0.53387 kJ/mol

$vibrational spectrum

# mode	symmetry	wave number	IR intensity	selection rules	
#		cm**(-1)	km/mol	IR	RAMAN
1		-0.00	0.00000	-	-
2		0.00	0.00000	-	-
3		0.00	0.00000	-	-
4		0.00	0.00000	-	-
5		0.00	0.00000	-	-
6		0.00	0.00000	-	-
7	a	20.09	0.72193	YES	YES
8	a	27.47	1.10336	YES	YES
9	a	61.12	0.20470	YES	YES
10	a	77.48	1.48461	YES	YES
11	a	86.61	2.20641	YES	YES
12	a	94.62	0.84249	YES	YES
13	a	131.86	1.58381	YES	YES
14	a	169.54	1.28932	YES	YES
15	a	197.44	30.00320	YES	YES

16	a	229.40	5.21913	YES	YES
17	a	244.90	0.66222	YES	YES
18	a	271.14	8.28478	YES	YES
19	a	364.75	16.91681	YES	YES
20	a	393.49	1.82797	YES	YES
21	a	400.21	1.06850	YES	YES
22	a	402.90	2.84636	YES	YES
23	a	406.10	1.08262	YES	YES
24	a	485.75	14.71942	YES	YES
25	a	489.79	20.38437	YES	YES
26	a	506.10	11.12671	YES	YES
27	a	508.04	5.60746	YES	YES
28	a	596.94	0.58797	YES	YES
29	a	600.77	3.73299	YES	YES
30	a	666.06	19.39306	YES	YES
31	a	671.87	9.35983	YES	YES
32	a	758.99	59.47940	YES	YES
33	a	787.98	78.90772	YES	YES
34	a	802.19	10.38202	YES	YES
35	a	808.65	20.23951	YES	YES
36	a	812.59	1.27061	YES	YES
37	a	826.57	3.71537	YES	YES
38	a	910.20	6.67321	YES	YES
39	a	911.60	0.69500	YES	YES
40	a	969.23	0.08278	YES	YES
41	a	970.41	0.50824	YES	YES
42	a	982.19	2.22026	YES	YES
43	a	987.58	2.10262	YES	YES
44	a	991.66	14.02617	YES	YES
45	a	995.80	2.65346	YES	YES
46	a	1000.49	0.91330	YES	YES
47	a	1007.69	0.12987	YES	YES
48	a	1069.03	5.10357	YES	YES
49	a	1070.09	3.10348	YES	YES
50	a	1146.01	16.37504	YES	YES
51	a	1147.19	44.19120	YES	YES
52	a	1154.48	0.12083	YES	YES
53	a	1159.55	2.04846	YES	YES
54	a	1233.84	98.74770	YES	YES
55	a	1257.73	106.73962	YES	YES
56	a	1293.23	1.37281	YES	YES
57	a	1294.95	2.24840	YES	YES
58	a	1358.81	1.64217	YES	YES
59	a	1363.67	4.05347	YES	YES
60	a	1443.45	21.14702	YES	YES
61	a	1461.56	5.79613	YES	YES
62	a	1468.76	129.03378	YES	YES
63	a	1478.77	96.81313	YES	YES
64	a	1541.13	11.52947	YES	YES
65	a	1560.41	74.13594	YES	YES
66	a	1574.24	17.27442	YES	YES
67	a	1587.91	140.06337	YES	YES
68	a	1909.82	157.15094	YES	YES
69	a	3122.64	0.03944	YES	YES
70	a	3124.90	0.10897	YES	YES
71	a	3129.38	0.79657	YES	YES
72	a	3133.83	0.58016	YES	YES
73	a	3136.55	0.78095	YES	YES
74	a	3138.95	0.37342	YES	YES
75	a	3143.30	1.55675	YES	YES
76	a	3144.41	4.47332	YES	YES
77	a	3146.80	0.74536	YES	YES
78	a	3149.30	0.46638	YES	YES

$end

$[NO(2FB)_2]^+$

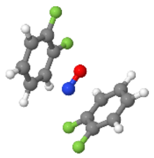

```
Method: (RI-)BP86(D3BJ)/def2-TZVPP
Symmetry: c1

Cartesian coordinates in Ångström:
C         -4.96738        0.60778        1.04888
C         -3.86636        1.41488        1.37542
C         -3.93434        2.77170        1.11776
C         -5.07250        3.32085        0.47897
C         -6.13042        2.50530        0.06920
H         -2.97403        0.99634        1.83505
F         -2.93255        3.58834        1.45373
F         -5.09953        4.62746        0.24418
H         -3.95669        3.84494        5.48186
C         -4.36653        2.83945        5.40167
H         -6.12204        3.30286        6.58570
C         -5.59342        2.53261        6.02695
C         -3.64333        1.85890        4.72179
C         -6.12729        1.25331        5.94489
H         -2.67078        2.06881        4.28074
H         -7.06693        0.98143        6.42324
C         -5.44150        0.28634        5.20044
C         -4.18594        0.58470        4.61314
F         -5.92294       -0.94745        5.08284
F         -3.53078       -0.39387        3.98055
C         -6.08081        1.14872        0.36376
N         -6.29957        1.42005        3.05481
O         -6.22166        2.54108        3.10392
H         -4.92735       -0.45989        1.25760
H         -6.90227        0.49766        0.06974
H         -6.97146        2.95620       -0.45504

SCF energy GEOOPT =  -991.5777155623 H
ZPE =   452.0 kJ/mol
FREEH energy =   496.48 kJ/mol
FREEH entropy =   0.57085 kJ/mol

$vibrational spectrum
#  mode     symmetry     wave number   IR intensity    selection rules
#                         cm**(-1)        km/mol         IR     RAMAN
     1                        0.00         0.00000         -       -
     2                        0.00         0.00000         -       -
     3                        0.00         0.00000         -       -
     4                        0.00         0.00000         -       -
     5                        0.00         0.00000         -       -
     6                        0.00         0.00000         -       -
     7         a             12.90         2.00781        YES     YES
     8         a             28.32         2.15239        YES     YES
     9         a             55.76         0.44982        YES     YES
    10         a             75.98         0.74047        YES     YES
    11         a             77.36         0.59868        YES     YES
    12         a             85.47         3.52443        YES     YES
    13         a            110.28         0.58560        YES     YES
    14         a            154.45         3.43782        YES     YES
    15         a            180.17        11.64964        YES     YES
    16         a            193.15         0.33900        YES     YES
    17         a            207.94        12.78528        YES     YES
    18         a            269.73         2.07065        YES     YES
    19         a            282.19         0.32787        YES     YES
    20         a            287.95         3.08714        YES     YES
    21         a            302.89         4.23913        YES     YES
    22         a            307.80         5.31866        YES     YES
    23         a            354.04        13.72848        YES     YES
    24         a            429.52         0.37952        YES     YES
    25         a            430.06         0.20944        YES     YES
    26         a            452.08         9.52529        YES     YES
    27         a            461.27        17.86551        YES     YES
    28         a            520.88         0.81638        YES     YES
    29         a            526.17         2.59072        YES     YES
    30         a            536.16         5.65128        YES     YES
    31         a            536.95         5.93677        YES     YES
    32         a            557.40        20.18734        YES     YES
    33         a            559.90         1.37811        YES     YES
    34         a            686.79         0.76311        YES     YES
    35         a            695.69         0.30406        YES     YES
```

36	a	754.31	23.16002	YES	YES
37	a	757.87	26.09170	YES	YES
38	a	762.26	57.87800	YES	YES
39	a	783.32	78.95379	YES	YES
40	a	841.51	5.50176	YES	YES
41	a	844.45	11.37646	YES	YES
42	a	847.65	3.20213	YES	YES
43	a	851.22	6.84875	YES	YES
44	a	939.53	1.57563	YES	YES
45	a	945.52	2.16730	YES	YES
46	a	966.31	0.28998	YES	YES
47	a	978.48	0.08613	YES	YES
48	a	1006.92	11.43198	YES	YES
49	a	1012.92	0.63889	YES	YES
50	a	1100.31	11.96503	YES	YES
51	a	1106.91	18.62916	YES	YES
52	a	1148.41	25.50268	YES	YES
53	a	1153.08	6.61159	YES	YES
54	a	1200.16	28.64738	YES	YES
55	a	1206.27	15.19172	YES	YES
56	a	1262.24	1.94296	YES	YES
57	a	1264.23	17.06258	YES	YES
58	a	1281.23	254.61265	YES	YES
59	a	1288.00	34.05286	YES	YES
60	a	1356.06	3.31760	YES	YES
61	a	1361.92	3.36276	YES	YES
62	a	1453.29	75.05334	YES	YES
63	a	1456.00	30.94297	YES	YES
64	a	1480.39	272.99961	YES	YES
65	a	1489.46	224.28087	YES	YES
66	a	1543.46	57.98487	YES	YES
67	a	1553.14	43.65566	YES	YES
68	a	1580.98	31.17786	YES	YES
69	a	1587.02	67.80543	YES	YES
70	a	1903.24	154.36060	YES	YES
71	a	3128.66	0.76034	YES	YES
72	a	3129.48	0.28944	YES	YES
73	a	3136.23	0.44290	YES	YES
74	a	3137.96	1.33313	YES	YES
75	a	3142.63	5.52510	YES	YES
76	a	3145.01	6.05465	YES	YES
77	a	3147.43	2.12346	YES	YES
78	a	3152.45	2.92985	YES	YES

$end

$[NO(3FB)_2]^+$

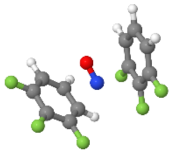

Method: (RI-)BP86(D3BJ)/def2-TZVPP
Symmetry: c1

Cartesian coordinates in Ångström:

C	-5.66807	0.46435	0.91461
C	-4.37221	0.68081	1.42906
C	-3.73694	1.89678	1.17809
C	-4.39465	2.90186	0.46924
C	-5.67740	2.66416	-0.05298
H	-3.88578	-0.12989	1.96761
H	-2.72947	2.06572	1.55109
H	-3.92667	3.86475	0.26987
H	-5.03931	4.16293	6.24846
C	-4.95964	3.14363	5.87451
H	-6.89074	2.52802	6.67376
C	-5.99896	2.24358	6.11874
C	-3.79540	2.76111	5.18274
C	-5.88732	0.95016	5.62435
H	-2.97443	3.45803	5.02472

```
F         -6.84752        0.05043        5.80667
C         -4.73501        0.54486        4.89991
C         -3.67940        1.45928        4.72706
F         -4.62803       -0.70549        4.46987
F         -2.57961        1.03885        4.08875
C         -6.32613        1.44367        0.14878
N         -6.15522        1.70655        3.07951
O         -6.00174        2.82004        3.03177
F         -6.28113       -0.69307        1.12699
F         -7.53835        1.21644       -0.34199
F         -6.29927        3.61093       -0.74937

SCF energy GEOOPT =  -1190.133106219 H
ZPE =   409.4 kJ/mol
FREEH energy =   458.97 kJ/mol
FREEH entropy =   0.61159 kJ/mol

$vibrational spectrum
#  mode     symmetry     wave number   IR intensity    selection rules
#                         cm**(-1)        km/mol         IR     RAMAN
     1                       -0.00         0.00000         -       -
     2                       -0.00         0.00000         -       -
     3                       -0.00         0.00000         -       -
     4                        0.00         0.00000         -       -
     5                        0.00         0.00000         -       -
     6                        0.00         0.00000         -       -
     7         a             15.03         0.24588        YES     YES
     8         a             23.21         0.66115        YES     YES
     9         a             45.69         0.44052        YES     YES
    10         a             61.12         1.29928        YES     YES
    11         a             67.16         0.88354        YES     YES
    12         a             73.05         4.48368        YES     YES
    13         a            105.79         0.93105        YES     YES
    14         a            140.09         1.42710        YES     YES
    15         a            149.95         3.66672        YES     YES
    16         a            154.10         4.45157        YES     YES
    17         a            176.31        13.33060        YES     YES
    18         a            232.66         3.53390        YES     YES
    19         a            249.86         1.09207        YES     YES
    20         a            267.74         1.57812        YES     YES
    21         a            272.87         0.04536        YES     YES
    22         a            274.84         2.43489        YES     YES
    23         a            298.68         1.27887        YES     YES
    24         a            299.68         6.28478        YES     YES
    25         a            315.33         1.46040        YES     YES
    26         a            331.62         0.78017        YES     YES
    27         a            352.13        60.81373        YES     YES
    28         a            465.60         5.73278        YES     YES
    29         a            466.13         0.30448        YES     YES
    30         a            487.72         2.23799        YES     YES
    31         a            489.91         4.76365        YES     YES
    32         a            509.83         2.98600        YES     YES
    33         a            529.15         0.86071        YES     YES
    34         a            560.01         3.79410        YES     YES
    35         a            563.10         6.83816        YES     YES
    36         a            568.01         2.47538        YES     YES
    37         a            579.09         2.48045        YES     YES
    38         a            680.34         9.02561        YES     YES
    39         a            683.46         5.08490        YES     YES
    40         a            686.12        12.86952        YES     YES
    41         a            691.70        18.90075        YES     YES
    42         a            771.85        65.78704        YES     YES
    43         a            781.81        45.54315        YES     YES
    44         a            814.50        10.03114        YES     YES
    45         a            824.02        10.00964        YES     YES
    46         a            879.75         0.15930        YES     YES
    47         a            890.68         1.39164        YES     YES
    48         a            962.91         0.24122        YES     YES
    49         a            964.23         2.70662        YES     YES
    50         a           1020.77        84.42726        YES     YES
    51         a           1024.51        71.45893        YES     YES
    52         a           1048.02         5.18346        YES     YES
    53         a           1061.51         9.10631        YES     YES
    54         a           1149.17         4.89803        YES     YES
```

55	a	1151.84	1.29312	YES	YES
56	a	1238.74	82.11131	YES	YES
57	a	1239.89	5.88248	YES	YES
58	a	1244.07	69.29759	YES	YES
59	a	1250.83	26.30430	YES	YES
60	a	1300.15	174.02790	YES	YES
61	a	1309.85	63.19596	YES	YES
62	a	1351.61	1.50968	YES	YES
63	a	1358.56	14.31658	YES	YES
64	a	1450.77	78.87253	YES	YES
65	a	1467.74	391.52895	YES	YES
66	a	1479.77	99.60259	YES	YES
67	a	1504.31	339.59713	YES	YES
68	a	1551.78	36.61153	YES	YES
69	a	1554.95	95.43977	YES	YES
70	a	1575.27	58.60213	YES	YES
71	a	1587.63	49.53751	YES	YES
72	a	1902.94	170.08269	YES	YES
73	a	3133.73	0.29922	YES	YES
74	a	3136.26	2.36213	YES	YES
75	a	3144.41	4.93850	YES	YES
76	a	3145.21	4.52593	YES	YES
77	a	3149.76	9.17497	YES	YES
78	a	3154.16	5.98908	YES	YES

$end

$[NO(4FB)_2]^+$

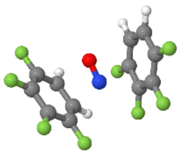

Method: (RI-)BP86(D3BJ)/def2-TZVPP
Symmetry: c1

Cartesian coordinates in Ångström:

C	-5.66438	0.48180	1.09517
C	-4.32044	0.69040	1.45427
C	-3.69641	1.87748	1.10034
C	-4.40902	2.83862	0.37409
C	-5.73805	2.61223	-0.05075
H	-3.79390	-0.09382	1.99313
H	-2.66012	2.07617	1.36571
F	-3.83433	3.98364	0.03067
H	-5.06536	4.08844	6.35099
C	-4.96454	3.09528	5.91677
F	-7.15292	2.53193	6.64252
C	-6.01717	2.19972	6.02875
C	-3.77790	2.71487	5.26330
C	-5.91635	0.91822	5.45763
H	-2.94092	3.40680	5.18620
F	-6.91033	0.05175	5.54329
C	-4.71024	0.52782	4.82158
C	-3.64613	1.43480	4.74446
F	-4.59944	-0.70858	4.34990
F	-2.51472	1.04296	4.14042
C	-6.36909	1.42363	0.30443
N	-6.22634	1.92681	3.07664
O	-5.80742	2.96842	2.98713
F	-6.27672	-0.65202	1.40937
F	-7.61861	1.18254	-0.06184
F	-6.37756	3.52857	-0.76237

SCF energy GEOOPT = -1388.687324234 H
ZPE = 367.1 kJ/mol
FREEH energy = 421.56 kJ/mol
FREEH entropy = 0.65082 kJ/mol

$vibrational spectrum
mode symmetry wave number IR intensity selection rules

#		cm**(-1)	km/mol	IR	RAMAN
1		-0.00	0.00000	-	-
2		0.00	0.00000	-	-
3		0.00	0.00000	-	-
4		0.00	0.00000	-	-
5		0.00	0.00000	-	-
6		0.00	0.00000	-	-
7	a	13.22	0.15586	YES	YES
8	a	18.38	0.75476	YES	YES
9	a	40.56	0.06876	YES	YES
10	a	58.95	0.36882	YES	YES
11	a	62.49	1.28388	YES	YES
12	a	66.29	3.01620	YES	YES
13	a	98.83	0.58905	YES	YES
14	a	132.76	2.58222	YES	YES
15	a	144.24	0.04069	YES	YES
16	a	145.79	11.95196	YES	YES
17	a	151.34	2.11610	YES	YES
18	a	164.40	4.36070	YES	YES
19	a	193.14	8.06260	YES	YES
20	a	244.85	2.49207	YES	YES
21	a	267.82	0.46350	YES	YES
22	a	271.38	0.20763	YES	YES
23	a	274.95	0.52257	YES	YES
24	a	277.07	0.33406	YES	YES
25	a	302.36	0.23066	YES	YES
26	a	311.79	9.77484	YES	YES
27	a	320.86	2.06288	YES	YES
28	a	324.01	4.79515	YES	YES
29	a	343.39	47.72234	YES	YES
30	a	373.09	0.54507	YES	YES
31	a	383.31	3.41270	YES	YES
32	a	441.62	6.43209	YES	YES
33	a	442.49	1.13705	YES	YES
34	a	464.21	0.36471	YES	YES
35	a	468.56	1.12053	YES	YES
36	a	507.30	0.57690	YES	YES
37	a	547.97	2.26873	YES	YES
38	a	592.58	14.13230	YES	YES
39	a	595.14	1.12413	YES	YES
40	a	596.08	5.85434	YES	YES
41	a	598.79	0.31873	YES	YES
42	a	674.89	11.20991	YES	YES
43	a	677.12	16.03649	YES	YES
44	a	687.89	0.03747	YES	YES
45	a	694.60	1.55046	YES	YES
46	a	735.78	18.34719	YES	YES
47	a	739.00	11.67527	YES	YES
48	a	802.15	41.07482	YES	YES
49	a	816.58	35.28403	YES	YES
50	a	921.99	0.01954	YES	YES
51	a	934.45	2.17286	YES	YES
52	a	978.13	81.15489	YES	YES
53	a	984.60	75.75701	YES	YES
54	a	1046.25	36.91458	YES	YES
55	a	1054.72	47.87939	YES	YES
56	a	1161.23	61.30654	YES	YES
57	a	1169.89	31.61541	YES	YES
58	a	1227.88	80.13128	YES	YES
59	a	1233.06	52.20406	YES	YES
60	a	1271.65	15.50165	YES	YES
61	a	1280.97	12.61755	YES	YES
62	a	1309.90	125.60610	YES	YES
63	a	1328.19	103.55053	YES	YES
64	a	1346.29	5.81118	YES	YES
65	a	1354.89	8.36176	YES	YES
66	a	1456.17	230.24729	YES	YES
67	a	1472.30	579.33261	YES	YES
68	a	1497.60	314.21511	YES	YES
69	a	1504.27	307.75719	YES	YES
70	a	1536.82	17.61618	YES	YES
71	a	1567.03	1.40952	YES	YES
72	a	1580.66	5.22958	YES	YES
73	a	1593.07	72.02525	YES	YES

74	a	1898.23	165.18310	YES	YES
75	a	3138.10	0.82553	YES	YES
76	a	3146.28	1.91930	YES	YES
77	a	3147.19	17.46040	YES	YES
78	a	3157.01	14.60145	YES	YES

$end

$[NO(5FB)_2]^+$

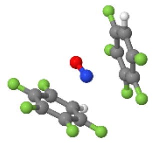

Method: (RI-)BP86(D3BJ)/def2-TZVPP
Symmetry: c1

Cartesian coordinates in Ångström:

C	-5.72351	0.43648	0.79765
C	-4.39964	0.56496	1.29752
C	-3.71275	1.77000	1.22010
C	-4.36371	2.85990	0.65128
C	-5.66780	2.75285	0.10513
F	-3.82283	-0.51858	1.80468
H	-2.69086	1.85784	1.58171
F	-3.75518	4.03583	0.57318
F	-5.17684	4.47283	6.03976
C	-5.04129	3.19423	5.71814
H	-6.90739	2.58655	6.62676
C	-6.02229	2.27229	6.07697
C	-3.86604	2.80641	5.03037
C	-5.84912	0.94964	5.69664
F	-2.93821	3.70576	4.73034
F	-6.75675	0.02935	5.99478
C	-4.69302	0.53166	4.98337
C	-3.68052	1.46652	4.70026
F	-4.52187	-0.74526	4.68648
F	-2.56311	1.06945	4.09675
C	-6.33424	1.52971	0.15598
N	-6.24049	1.45643	3.09311
O	-6.13664	2.58086	3.03647
F	-6.34615	-0.72719	0.85860
F	-7.55604	1.40287	-0.34244
F	-6.24209	3.80707	-0.45174

SCF energy GEOOPT = -1587.241780649 H
ZPE = 325.4 kJ/mol
FREEH energy = 384.59 kJ/mol
FREEH entropy = 0.68030 kJ/mol

$vibrational spectrum

# mode	symmetry	wave number cm**(-1)	IR intensity km/mol	selection rules IR	RAMAN
1		-0.00	0.00000	-	-
2		-0.00	0.00000	-	-
3		-0.00	0.00000	-	-
4		0.00	0.00000	-	-
5		0.00	0.00000	-	-
6		0.00	0.00000	-	-
7	a	17.98	0.15347	YES	YES
8	a	20.48	0.17299	YES	YES
9	a	45.12	0.20961	YES	YES
10	a	50.13	0.35516	YES	YES
11	a	55.15	0.84076	YES	YES
12	a	66.03	0.93023	YES	YES
13	a	98.98	0.27751	YES	YES
14	a	122.61	0.02882	YES	YES
15	a	127.69	0.24723	YES	YES
16	a	139.05	0.84173	YES	YES
17	a	147.24	9.82852	YES	YES
18	a	157.33	3.85254	YES	YES

19	a	168.08	8.23421	YES	YES
20	a	223.53	1.07142	YES	YES
21	a	234.36	6.26079	YES	YES
22	a	261.02	0.72609	YES	YES
23	a	266.26	0.52024	YES	YES
24	a	266.80	0.35753	YES	YES
25	a	268.08	0.21904	YES	YES
26	a	273.01	0.45900	YES	YES
27	a	297.04	2.45223	YES	YES
28	a	301.36	4.20576	YES	YES
29	a	315.57	0.31860	YES	YES
30	a	323.72	0.98131	YES	YES
31	a	327.94	3.73523	YES	YES
32	a	328.72	2.72832	YES	YES
33	a	347.13	22.97104	YES	YES
34	a	415.67	0.07630	YES	YES
35	a	417.33	1.60819	YES	YES
36	a	421.20	12.42450	YES	YES
37	a	426.53	2.50808	YES	YES
38	a	457.36	11.34946	YES	YES
39	a	458.40	0.54334	YES	YES
40	a	554.25	5.33467	YES	YES
41	a	556.74	7.36915	YES	YES
42	a	570.63	0.33489	YES	YES
43	a	570.93	0.11810	YES	YES
44	a	643.44	0.48027	YES	YES
45	a	645.55	2.63208	YES	YES
46	a	677.77	0.97547	YES	YES
47	a	678.47	1.13009	YES	YES
48	a	687.99	3.38685	YES	YES
49	a	688.87	3.06216	YES	YES
50	a	705.35	19.20870	YES	YES
51	a	706.75	12.40493	YES	YES
52	a	828.22	25.23823	YES	YES
53	a	830.38	23.41813	YES	YES
54	a	950.91	15.12260	YES	YES
55	a	953.72	165.20543	YES	YES
56	a	1070.07	65.52929	YES	YES
57	a	1076.93	157.97904	YES	YES
58	a	1153.02	37.63732	YES	YES
59	a	1156.17	17.18564	YES	YES
60	a	1178.15	71.24656	YES	YES
61	a	1179.40	48.97547	YES	YES
62	a	1286.62	7.74927	YES	YES
63	a	1292.72	5.71854	YES	YES
64	a	1341.39	3.78713	YES	YES
65	a	1342.81	2.39862	YES	YES
66	a	1394.90	415.13630	YES	YES
67	a	1415.72	17.24324	YES	YES
68	a	1467.52	93.09189	YES	YES
69	a	1480.95	705.06806	YES	YES
70	a	1495.30	347.65278	YES	YES
71	a	1501.68	293.37368	YES	YES
72	a	1550.38	81.08380	YES	YES
73	a	1554.40	119.34007	YES	YES
74	a	1589.19	32.50960	YES	YES
75	a	1597.64	58.55387	YES	YES
76	a	1871.29	147.60735	YES	YES
77	a	3149.80	26.57904	YES	YES
78	a	3156.52	19.10225	YES	YES

$end

$[NO(DCM)_2]^+$

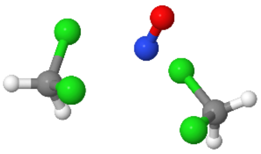

Method: (RI-)BP86(D3BJ)/def2-TZVPP
Symmetry: c1

```
Cartesian coordinates in Ångström:
O         -0.26101        2.35419        0.52101
N         -0.20505        1.29995        0.18357
Cl        -0.40357       -0.62005        2.09065
Cl         2.11384       -0.02920        0.64879
C          1.38566       -0.69849        2.14281
H          1.73184       -0.10492        2.99042
H          1.67401       -1.74923        2.21567
Cl         0.11161        0.94492       -2.46139
Cl        -2.40616        0.35000       -1.03115
C         -1.21743       -0.23284       -2.23579
H         -0.79005       -1.16896       -1.87290
H         -1.73368       -0.34536       -3.19170

SCF energy GEOOPT =  -2049.415188076 H
ZPE =   169.1 kJ/mol
FREEH energy =   201.11 kJ/mol
FREEH entropy =   0.49395 kJ/mol

$vibrational spectrum
#  mode     symmetry     wave number   IR intensity    selection rules
#                         cm**(-1)        km/mol         IR     RAMAN
     1                      -0.00         0.00000         -       -
     2                       0.00         0.00000         -       -
     3                       0.00         0.00000         -       -
     4                       0.00         0.00000         -       -
     5                       0.00         0.00000         -       -
     6                       0.00         0.00000         -       -
     7        a             30.41         0.16544        YES     YES
     8        a             34.75         1.20055        YES     YES
     9        a             40.43         0.04013        YES     YES
    10        a             62.10         5.21895        YES     YES
    11        a             77.15         2.17865        YES     YES
    12        a             89.03        11.65715        YES     YES
    13        a            107.55         0.41166        YES     YES
    14        a            126.12        34.52778        YES     YES
    15        a            147.46        16.06071        YES     YES
    16        a            280.46         6.90208        YES     YES
    17        a            289.96         3.96621        YES     YES
    18        a            325.42        10.73377        YES     YES
    19        a            342.41        85.20854        YES     YES
    20        a            664.98        32.14788        YES     YES
    21        a            669.44        97.00788        YES     YES
    22        a            672.70        22.28820        YES     YES
    23        a            675.01        13.31134        YES     YES
    24        a            871.62         0.90371        YES     YES
    25        a            876.12         0.71734        YES     YES
    26        a           1120.46         0.24180        YES     YES
    27        a           1123.04         0.74062        YES     YES
    28        a           1231.79         3.88065        YES     YES
    29        a           1233.81         2.88788        YES     YES
    30        a           1402.38         2.91944        YES     YES
    31        a           1406.49         0.11814        YES     YES
    32        a           2016.31       433.42292        YES     YES
    33        a           3046.33         6.44858        YES     YES
    34        a           3047.45         3.28769        YES     YES
    35        a           3132.90        14.80871        YES     YES
    36        a           3134.42         9.13538        YES     YES
$end
```

$[NO(DCE)_2]^+$

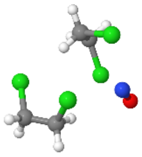

```
Method: (RI-)BP86(D3BJ)/def2-TZVPP
Symmetry: c1
```

Cartesian coordinates in Ångström:

C	-0.0703186	2.0147335	0.8663505
C	1.0007458	2.3424757	-0.1377464
Cl	0.6323271	1.4769628	2.4542567
Cl	1.9757646	0.8886280	-0.5821380
H	-0.6646381	2.9038242	1.1151924
H	-0.7319370	1.2082071	0.5334732
H	0.5300153	2.6931609	-1.0648742
H	1.7076795	3.0945048	0.2249021
N	1.3576491	-0.9625500	2.2846969
O	2.2374300	-1.0841607	1.6180091
C	-1.5298791	-1.7609784	-1.4667602
C	-0.4047496	-2.1062217	-0.5177797
Cl	-1.7496960	0.0073476	-1.6686280
Cl	-0.8047779	-1.5970517	1.1879019
H	-1.2738519	-2.1695879	-2.4531506
H	-2.4882031	-2.1792333	-1.1432770
H	-0.2456604	-3.1892618	-0.4743146
H	0.5221004	-1.5807993	-0.7761144

SCF energy GEOOPT = -2128.101875085 H
ZPE = 316.0 kJ/mol
FREEH energy = 353.72 kJ/mol
FREEH entropy = 0.53076 kJ/mol

$vibrational spectrum

# mode	symmetry	wave number	IR intensity	selection rules	
#		cm**(-1)	km/mol	IR	RAMAN
1		-0.00	0.00000	-	-
2		-0.00	0.00000	-	-
3		0.00	0.00000	-	-
4		0.00	0.00000	-	-
5		0.00	0.00000	-	-
6		0.00	0.00000	-	-
7	a	28.58	1.30264	YES	YES
8	a	41.22	1.54826	YES	YES
9	a	53.06	0.20698	YES	YES
10	a	59.28	1.48471	YES	YES
11	a	72.65	6.25029	YES	YES
12	a	79.46	0.16362	YES	YES
13	a	107.95	6.69159	YES	YES
14	a	120.93	4.18436	YES	YES
15	a	124.36	1.36012	YES	YES
16	a	165.34	29.79517	YES	YES
17	a	202.18	16.44615	YES	YES
18	a	259.99	6.78758	YES	YES
19	a	263.88	0.65576	YES	YES
20	a	342.06	19.03136	YES	YES
21	a	361.19	24.27895	YES	YES
22	a	398.74	7.26597	YES	YES
23	a	403.67	5.09445	YES	YES
24	a	597.13	20.20280	YES	YES
25	a	606.53	19.12046	YES	YES
26	a	641.79	16.53854	YES	YES
27	a	658.76	17.09036	YES	YES
28	a	842.13	13.89598	YES	YES
29	a	849.71	23.88012	YES	YES
30	a	914.17	9.04577	YES	YES
31	a	920.37	7.99875	YES	YES
32	a	1005.76	2.55723	YES	YES
33	a	1010.73	0.79343	YES	YES
34	a	1118.69	0.22090	YES	YES
35	a	1124.47	0.57491	YES	YES
36	a	1180.34	0.28788	YES	YES
37	a	1181.78	2.58313	YES	YES
38	a	1251.19	6.50462	YES	YES
39	a	1262.33	31.28858	YES	YES
40	a	1290.89	3.34675	YES	YES
41	a	1294.74	19.94126	YES	YES
42	a	1403.27	10.57285	YES	YES
43	a	1409.67	10.30149	YES	YES
44	a	1416.74	5.94686	YES	YES
45	a	1417.17	11.71831	YES	YES
46	a	2005.30	519.08281	YES	YES

47	a	3004.10	3.57145	YES	YES
48	a	3005.07	3.60944	YES	YES
49	a	3008.03	4.11775	YES	YES
50	a	3014.18	3.78848	YES	YES
51	a	3068.47	12.38059	YES	YES
52	a	3070.64	1.90503	YES	YES
53	a	3082.27	1.19086	YES	YES
54	a	3086.20	5.57228	YES	YES

$end

[NO(AN)$_2$]$^+$

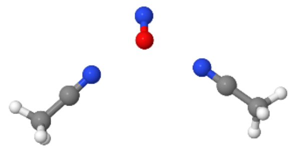

Method: (RI-)BP86(D3BJ)/def2-TZVPP
Symmetry: c1

Cartesian coordinates in Ångström:

N	-1.8832496	0.3321127	0.0510430
C	-2.7692858	-0.0376493	-0.6022402
C	-3.8929022	-0.4794383	-1.4024417
H	-3.9410979	0.1009395	-2.3329931
H	-3.7811196	-1.5436146	-1.6482505
H	-4.8276380	-0.3382821	-0.8442088
N	2.3392825	0.5047247	0.0086401
C	3.2388402	0.2085497	-0.6633974
C	4.3780531	-0.1397587	-1.4873598
H	5.2519564	0.4526888	-1.1863796
H	4.6118838	-1.2060364	-1.3705860
H	4.1536582	0.0653682	-2.5422439
N	0.2031850	1.4823472	1.9002223
O	0.2290539	0.6773486	1.1801057

SCF energy GEOOPT = -395.3210056072 H
ZPE = 249.6 kJ/mol
FREEH energy = 283.32 kJ/mol
FREEH entropy = 0.52768 kJ/mol/K

$vibrational spectrum

# mode	symmetry	wave number	IR intensity	selection rules	
#		cm**(-1)	km/mol	IR	RAMAN
1		-0.00	0.00000	-	-
2		0.00	0.00000	-	-
3		0.00	0.00000	-	-
4		0.00	0.00000	-	-
5		0.00	0.00000	-	-
6		0.00	0.00000	-	-
7	a	11.64	0.07755	YES	YES
8	a	12.64	0.00156	YES	YES
9	a	13.75	0.00088	YES	YES
10	a	50.39	4.42003	YES	YES
11	a	51.33	0.38422	YES	YES
12	a	56.93	13.38846	YES	YES
13	a	63.22	30.94488	YES	YES
14	a	125.07	9.62631	YES	YES
15	a	167.87	15.35188	YES	YES
16	a	169.91	48.90613	YES	YES
17	a	268.99	78.10464	YES	YES
18	a	376.59	0.41853	YES	YES
19	a	377.73	1.45993	YES	YES
20	a	378.92	1.19209	YES	YES
21	a	379.99	1.94541	YES	YES
22	a	928.84	6.01564	YES	YES
23	a	928.90	1.89800	YES	YES
24	a	1010.08	2.11431	YES	YES
25	a	1010.25	9.04683	YES	YES
26	a	1013.00	1.27909	YES	YES
27	a	1013.44	9.92931	YES	YES

28	a	1356.00	2.76847	YES	YES
29	a	1356.42	4.65381	YES	YES
30	a	1407.78	22.03244	YES	YES
31	a	1407.92	10.75846	YES	YES
32	a	1410.73	5.26201	YES	YES
33	a	1410.81	24.70331	YES	YES
34	a	2213.82	243.28821	YES	YES
35	a	2266.81	328.06559	YES	YES
36	a	2272.16	70.63238	YES	YES
37	a	2983.84	19.91410	YES	YES
38	a	2984.02	6.17630	YES	YES
39	a	3061.04	2.71750	YES	YES
40	a	3061.58	2.76242	YES	YES
41	a	3061.62	2.67825	YES	YES
42	a	3062.16	2.86733	YES	YES

$end

$[NO(DMF)_2]^+$

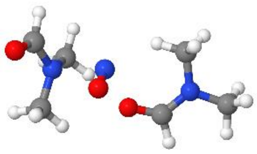

Method: (RI-)BP86(D3BJ)/def2-TZVPP
Symmetry: c1

Cartesian coordinates in Ångström:

N	-0.74940	-0.33313	-1.27952
O	-1.18661	0.27591	-2.11446
O	-0.10141	1.13020	0.15309
C	1.03991	1.65212	0.25943
N	2.21823	1.08375	0.01797
C	3.45829	1.85641	0.12506
C	2.34096	-0.31092	-0.39979
H	1.10078	2.69895	0.60277
H	3.96002	1.89835	-0.85047
H	3.23254	2.87574	0.45396
H	4.12901	1.38087	0.85177
H	3.20464	-0.75697	0.10716
H	1.43718	-0.85805	-0.12071
H	2.49855	-0.38137	-1.48517
O	-3.64532	-1.65997	-1.65721
C	-2.91527	-1.86926	-0.71621
N	-2.46093	-0.83953	0.14183
C	-1.82404	-1.21608	1.40299
C	-3.18982	0.42594	0.10530
H	-2.48115	-2.85750	-0.46030
H	-2.58220	-1.32143	2.19299
H	-1.30348	-2.17237	1.27913
H	-1.11014	-0.43837	1.69086
H	-2.56656	1.21071	0.54346
H	-3.45396	0.65942	-0.93230
H	-4.12794	0.33167	0.67227

SCF energy GEOOPT = -627.0131730862 H
ZPE = 544.8 kJ/mol
FREEH energy = 588.45 kJ/mol
FREEH entropy = 0.56036 kJ/mol

$vibrational spectrum

# mode	symmetry	wave number	IR intensity	selection rules	
#		cm**(-1)	km/mol	IR	RAMAN
1		0.00	0.00000	-	-
2		0.00	0.00000	-	-
3		0.00	0.00000	-	-
4		0.00	0.00000	-	-
5		0.00	0.00000	-	-
6		0.00	0.00000	-	-
7	a	16.37	0.27979	YES	YES
8	a	31.41	2.00949	YES	YES

9	a	39.40	0.50018	YES	YES
10	a	69.00	2.94048	YES	YES
11	a	92.66	1.99238	YES	YES
12	a	104.43	2.39344	YES	YES
13	a	131.25	1.29131	YES	YES
14	a	140.37	2.01039	YES	YES
15	a	156.58	18.69343	YES	YES
16	a	164.25	7.79637	YES	YES
17	a	172.69	6.21086	YES	YES
18	a	192.67	1.23172	YES	YES
19	a	217.48	6.85420	YES	YES
20	a	246.81	0.91798	YES	YES
21	a	256.48	2.68260	YES	YES
22	a	301.57	2.27452	YES	YES
23	a	308.33	8.77617	YES	YES
24	a	348.34	53.15813	YES	YES
25	a	377.79	11.29990	YES	YES
26	a	384.58	3.44923	YES	YES
27	a	396.83	41.85300	YES	YES
28	a	403.26	41.53630	YES	YES
29	a	450.11	8.80134	YES	YES
30	a	620.52	3.33767	YES	YES
31	a	641.35	30.03946	YES	YES
32	a	817.64	30.66326	YES	YES
33	a	846.47	0.31264	YES	YES
34	a	960.12	2.33678	YES	YES
35	a	975.69	18.70480	YES	YES
36	a	1001.16	127.27039	YES	YES
37	a	1026.71	6.82342	YES	YES
38	a	1036.15	11.40317	YES	YES
39	a	1068.78	2.63881	YES	YES
40	a	1082.90	4.93012	YES	YES
41	a	1088.90	41.77041	YES	YES
42	a	1124.22	7.68936	YES	YES
43	a	1132.48	3.28881	YES	YES
44	a	1209.93	22.90452	YES	YES
45	a	1215.97	10.39030	YES	YES
46	a	1277.10	79.95787	YES	YES
47	a	1351.15	22.06966	YES	YES
48	a	1356.83	3.18271	YES	YES
49	a	1388.54	11.73649	YES	YES
50	a	1390.06	3.07005	YES	YES
51	a	1399.49	23.99193	YES	YES
52	a	1412.82	4.97409	YES	YES
53	a	1420.76	60.27777	YES	YES
54	a	1424.98	2.59819	YES	YES
55	a	1429.95	3.72529	YES	YES
56	a	1432.41	17.58585	YES	YES
57	a	1443.32	18.23600	YES	YES
58	a	1452.21	5.06010	YES	YES
59	a	1454.37	34.04712	YES	YES
60	a	1464.27	24.49707	YES	YES
61	a	1481.40	66.26058	YES	YES
62	a	1620.33	849.50136	YES	YES
63	a	1723.52	189.89323	YES	YES
64	a	1941.24	411.83608	YES	YES
65	a	2915.42	32.83329	YES	YES
66	a	2970.42	5.34451	YES	YES
67	a	2974.92	1.43995	YES	YES
68	a	2976.61	8.50104	YES	YES
69	a	2978.88	7.25467	YES	YES
70	a	2983.00	13.39287	YES	YES
71	a	3047.53	6.26801	YES	YES
72	a	3048.27	0.75624	YES	YES
73	a	3050.12	0.06443	YES	YES
74	a	3050.81	2.93306	YES	YES
75	a	3083.79	2.56199	YES	YES
76	a	3087.66	1.51309	YES	YES
77	a	3099.00	1.79563	YES	YES
78	a	3100.36	4.75894	YES	YES

$end

10.1.4 Ag^+ related structures

$[Ag(1FB)_1]^+$ (Isomer 1.1 – gasphase structure)

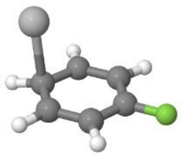

Method: (RI-)BP86(D3BJ)/def2-TZVPP
Symmetry: cs

Cartesian coordinates in Ångström:

Ag	1.8391002	-1.8574672	0.0000000
C	-0.2115804	-0.5575945	-1.2338624
H	-0.3483004	-1.0871537	-2.1762591
H	0.0392541	1.3914227	2.1544052
C	-0.3090120	-1.2755273	0.0000000
H	-0.7175184	-2.2901429	0.0000000
F	0.2160037	2.8058601	0.0000000
C	-0.2115804	-0.5575945	1.2338624
H	-0.3483004	-1.0871537	2.1762591
C	-0.0220504	0.8200800	1.2298147
C	0.0578865	1.4838190	0.0000000
C	-0.0220504	0.8200800	-1.2298147
H	0.0392541	1.3914227	-2.1544052

SCF energy GEOOPT = -478.5196525201 H
ZPE = 238.2 kJ/mol
FREEH energy = 257.52 kJ/mol
FREEH entropy = 0.37495 kJ/mol/K

$vibrational spectrum

# mode	symmetry	wave number	IR intensity	selection rules	
#		cm**(-1)	km/mol	IR	RAMAN
1		0.00	0.00000	-	-
2		0.00	0.00000	-	-
3		0.00	0.00000	-	-
4		0.00	0.00000	-	-
5		0.00	0.00000	-	-
6		0.00	0.00000	-	-
7	a'	60.00	0.13933	YES	YES
8	a"	61.73	0.23557	YES	YES
9	a'	174.89	0.38946	YES	YES
10	a'	314.41	2.35261	YES	YES
11	a"	401.93	3.06170	YES	YES
12	a"	408.83	0.40499	YES	YES
13	a'	502.47	14.51941	YES	YES
14	a'	514.89	4.62589	YES	YES
15	a"	597.43	0.17541	YES	YES
16	a'	682.57	9.06524	YES	YES
17	a'	807.35	34.50219	YES	YES
18	a'	815.09	65.21074	YES	YES
19	a"	823.64	0.10818	YES	YES
20	a'	901.82	18.34176	YES	YES
21	a'	947.38	22.50141	YES	YES
22	a"	962.84	1.14714	YES	YES
23	a'	978.15	2.23847	YES	YES
24	a'	995.81	0.68073	YES	YES
25	a"	1049.70	3.96738	YES	YES
26	a"	1140.44	0.51285	YES	YES
27	a'	1149.11	26.40614	YES	YES
28	a'	1261.95	113.77110	YES	YES
29	a"	1292.45	1.28655	YES	YES
30	a"	1352.86	0.17973	YES	YES
31	a"	1436.69	11.86523	YES	YES
32	a'	1466.39	87.30870	YES	YES
33	a"	1536.44	3.95696	YES	YES
34	a'	1575.43	186.05751	YES	YES
35	a'	3078.71	4.41870	YES	YES
36	a"	3123.65	0.78114	YES	YES

```
    37        a'           3123.86         2.37209       YES     YES
    38        a'           3144.22         0.40799       YES     YES
    39        a"           3144.28        12.46470       YES     YES
$end
```

$[Ag(1FB)_1]^+$ (Isomer 1.2 – structure in solution)

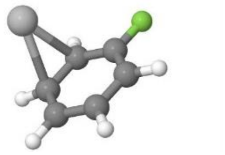

```
Method: (RI-)BP86(D3BJ)/def2-TZVPP
Symmetry: c1

Cartesian coordinates in Ångström:
Ag    1.7615595   -1.7948353    0.0154405
C    -0.3444969   -1.0545553   -0.7426352
H    -0.6811974   -1.9432471   -1.2793282
H     0.2573017    2.2628062    1.2398659
C    -0.3289181   -1.0697056    0.6946676
H    -0.6905787   -1.9320229    1.2573144
H     0.2588588    2.2630165   -1.2580068
C    -0.0968491    0.1503968    1.3685598
F    -0.1181001    0.1470974    2.7041653
C     0.1112774    1.3401519    0.6802285
C     0.1067024    1.3277828   -0.7209049
C    -0.1044901    0.1468417   -1.4357470
H    -0.1310594    0.1562628   -2.5236300

SCF energy GEOOPT =  -478.5170686260 H
ZPE =   237.9 kJ/mol
FREEH energy =   257.34 kJ/mol
FREEH entropy =   0.37640 kJ/mol/K

$vibrational spectrum
#  mode     symmetry     wave number   IR intensity    selection rules
#                         cm**(-1)        km/mol         IR     RAMAN
     1                      -0.00         0.00000         -       -
     2                      -0.00         0.00000         -       -
     3                      -0.00         0.00000         -       -
     4                       0.00         0.00000         -       -
     5                       0.00         0.00000         -       -
     6                       0.00         0.00000         -       -
     7        a             37.78         0.72050       YES     YES
     8        a             81.38         0.03382       YES     YES
     9        a            189.49         0.86602       YES     YES
    10        a            274.61         0.93293       YES     YES
    11        a            394.32         1.90569       YES     YES
    12        a            413.51         0.25373       YES     YES
    13        a            504.88         4.23005       YES     YES
    14        a            513.73         7.94100       YES     YES
    15        a            595.42         0.31272       YES     YES
    16        a            670.89         6.83863       YES     YES
    17        a            770.92        67.82997       YES     YES
    18        a            803.19        14.75311       YES     YES
    19        a            862.95        12.21685       YES     YES
    20        a            894.23        10.67307       YES     YES
    21        a            948.12         2.35523       YES     YES
    22        a            979.06         9.78113       YES     YES
    23        a            989.95         0.28338       YES     YES
    24        a           1005.49         1.96063       YES     YES
    25        a           1046.02         9.06820       YES     YES
    26        a           1141.00         4.73228       YES     YES
    27        a           1160.85         4.04962       YES     YES
    28        a           1235.65        90.83394       YES     YES
    29        a           1289.03         6.33340       YES     YES
    30        a           1340.59         7.09365       YES     YES
    31        a           1420.52         7.78286       YES     YES
    32        a           1464.70       104.05633       YES     YES
    33        a           1549.13        26.43105       YES     YES
```

34	a	1567.63	63.81807	YES	YES
35	a	3102.89	1.47856	YES	YES
36	a	3114.42	11.11025	YES	YES
37	a	3128.51	0.03628	YES	YES
38	a	3140.35	5.83553	YES	YES
39	a	3145.85	2.38512	YES	YES

$end

[Ag(1FB)$_2$]$^+$ (Isomer 2.1 – gasphase structure)

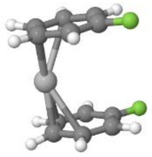

Method: (RI-)BP86(D3BJ)/def2-TZVPP
Symmetry: c1

Cartesian coordinates in Ångström:

C	-2.1952303	2.0253134	-0.7223295
C	-1.2021927	2.8754306	-0.2338130
C	-0.5575528	2.5926916	1.0022637
C	-0.9380144	1.4328138	1.7337079
C	-1.9294446	0.5823924	1.2414853
C	-2.5446897	0.9011966	0.0289383
C	1.6721999	-2.1621531	-0.0585411
C	1.1555148	-1.7094231	-1.2744821
C	1.6470577	-0.5173886	-1.8091106
C	2.6455433	0.2232808	-1.1166777
C	3.1377454	-0.2680405	0.1241949
C	2.6467367	-1.4615286	0.6552793
F	-3.5039968	0.0911381	-0.4359912
F	1.2081998	-3.3117917	0.4470905
Ag	1.0318738	1.3914469	-0.0570124
H	-2.7109150	2.2301154	-1.6586956
H	-0.9528970	3.7868854	-0.7758808
H	0.0712380	3.3506091	1.4733653
H	-0.4878650	1.2363419	2.7060952
H	-2.2447431	-0.3032762	1.7899456
H	0.4010097	-2.2983485	-1.7926713
H	1.3021585	-0.1770463	-2.7848076
H	3.1620452	1.0425605	-1.6204240
H	3.9372873	0.2657863	0.6365436
H	3.0191314	-1.8625060	1.5961272

SCF energy GEOOPT = -810.2268189600 H
ZPE = 477.0 kJ/mol
FREEH energy = 515.24 kJ/mol
FREEH entropy = 0.55316 kJ/mol/K

$vibrational spectrum

# mode	symmetry	wave number	IR intensity	selection rules	
#		cm**(-1)	km/mol	IR	RAMAN
1		-0.00	0.00000	-	-
2		-0.00	0.00000	-	-
3		-0.00	0.00000	-	-
4		-0.00	0.00000	-	-
5		0.00	0.00000	-	-
6		0.00	0.00000	-	-
7	a	9.39	0.08602	YES	YES
8	a	19.30	0.03225	YES	YES
9	a	32.07	0.67064	YES	YES
10	a	55.77	0.07453	YES	YES
11	a	58.10	0.68054	YES	YES
12	a	60.44	0.08901	YES	YES
13	a	76.52	0.28751	YES	YES
14	a	163.17	0.01426	YES	YES
15	a	191.38	0.15036	YES	YES
16	a	298.87	0.77503	YES	YES
17	a	310.24	2.25277	YES	YES
18	a	399.57	3.81106	YES	YES

19	a	399.67	0.94361	YES	YES
20	a	409.49	0.61226	YES	YES
21	a	410.08	0.08063	YES	YES
22	a	505.77	11.14309	YES	YES
23	a	506.64	9.30626	YES	YES
24	a	512.31	21.52723	YES	YES
25	a	513.32	0.29293	YES	YES
26	a	601.88	0.21103	YES	YES
27	a	602.05	0.01411	YES	YES
28	a	683.47	10.03566	YES	YES
29	a	683.84	1.26070	YES	YES
30	a	796.80	180.49229	YES	YES
31	a	800.41	7.30469	YES	YES
32	a	810.71	17.32245	YES	YES
33	a	811.20	20.92542	YES	YES
34	a	823.52	0.01934	YES	YES
35	a	825.11	0.21403	YES	YES
36	a	892.79	2.22880	YES	YES
37	a	898.18	4.27952	YES	YES
38	a	954.88	15.74362	YES	YES
39	a	957.21	8.67731	YES	YES
40	a	959.74	1.65815	YES	YES
41	a	960.33	2.44870	YES	YES
42	a	984.84	4.43893	YES	YES
43	a	985.52	0.93740	YES	YES
44	a	999.37	2.21524	YES	YES
45	a	999.86	1.09666	YES	YES
46	a	1053.50	1.28244	YES	YES
47	a	1053.80	5.33297	YES	YES
48	a	1141.12	0.38873	YES	YES
49	a	1141.24	0.33076	YES	YES
50	a	1146.42	13.49642	YES	YES
51	a	1147.67	17.74443	YES	YES
52	a	1246.66	57.74438	YES	YES
53	a	1248.84	145.31944	YES	YES
54	a	1291.19	1.24874	YES	YES
55	a	1291.35	0.29299	YES	YES
56	a	1345.85	0.05901	YES	YES
57	a	1346.34	0.54875	YES	YES
58	a	1433.23	3.25046	YES	YES
59	a	1433.36	12.89912	YES	YES
60	a	1468.98	28.75473	YES	YES
61	a	1470.55	127.41665	YES	YES
62	a	1553.73	7.31855	YES	YES
63	a	1553.82	2.46423	YES	YES
64	a	1574.33	62.30772	YES	YES
65	a	1576.53	157.62004	YES	YES
66	a	3097.54	0.88093	YES	YES
67	a	3097.64	2.96860	YES	YES
68	a	3122.63	0.19591	YES	YES
69	a	3122.65	0.03212	YES	YES
70	a	3124.36	0.77200	YES	YES
71	a	3124.39	0.80150	YES	YES
72	a	3143.77	1.86585	YES	YES
73	a	3143.86	1.53484	YES	YES
74	a	3143.93	7.16483	YES	YES
75	a	3143.94	1.59287	YES	YES

$end

$[Ag(1FB)_2]^+$ (Isomer 2.2 – structure in solution)

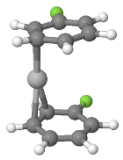

Method: (RI-)BP86(D3BJ)/def2-TZVPP
Symmetry: c1

Cartesian coordinates in Ångström:
C   0.7536794   2.1155076   -1.4850005

```
C     1.7456050    1.7850654   -0.5535045
C     1.4480853    1.6742062    0.8061648
C     0.1304704    1.9118473    1.2576976
C    -0.8953276    2.2266073    0.3198749
C    -0.5475583    2.3220164   -1.0403012
F    -1.5062713    2.6423696   -1.9198276
Ag   -0.8249414   -0.1238384    0.7551858
C    -0.6633818   -2.3301300   -1.3068654
C    -1.6239231   -2.0928945   -0.3056296
C     0.0194488   -2.8657901    1.3492861
C     0.9334997   -3.0979154    0.3200568
C     0.5990105   -2.8346809   -1.0138027
F    -0.9974944   -2.0783446   -2.5806011
C    -1.2701611   -2.3779311    1.0452538
H     0.9798484    2.2210036   -2.5446850
H     2.7666052    1.6276479   -0.8978839
H     2.2313207    1.4481261    1.5271192
H    -0.0787716    1.9891053    2.3257617
H    -1.8910327    2.5289705    0.6433337
H    -2.6450620   -1.8378132   -0.5880653
H     0.2778310   -3.0964460    2.3809845
H     1.9186581   -3.4997906    0.5518431
H     1.2995012   -3.0265119   -1.8248587
H    -2.0399383   -2.3435866    1.8176636

SCF energy GEOOPT =  -810.2239910214 H
ZPE =   476.7 kJ/mol
FREEH energy =   515.14 kJ/mol
FREEH entropy =   0.57022 kJ/mol/K

$vibrational spectrum
#  mode     symmetry     wave number   IR intensity    selection rules
#                         cm**(-1)        km/mol         IR     RAMAN
     1                        0.00         0.00000        -       -
     2                        0.00         0.00000        -       -
     3                        0.00         0.00000        -       -
     4                        0.00         0.00000        -       -
     5                        0.00         0.00000        -       -
     6                        0.00         0.00000        -       -
     7        a               1.44         0.27479       YES     YES
     8        a              19.33         0.05887       YES     YES
     9        a              29.64         0.18604       YES     YES
    10        a              34.86         0.52013       YES     YES
    11        a              50.16         0.04344       YES     YES
    12        a              75.65         0.15733       YES     YES
    13        a              91.82         0.22132       YES     YES
    14        a             195.14         0.20714       YES     YES
    15        a             209.20         1.12195       YES     YES
    16        a             249.62         0.35647       YES     YES
    17        a             266.84         0.31196       YES     YES
    18        a             395.76         1.31041       YES     YES
    19        a             396.27         2.57454       YES     YES
    20        a             419.45         0.03809       YES     YES
    21        a             420.74         0.22274       YES     YES
    22        a             507.07         0.84165       YES     YES
    23        a             507.48         7.52735       YES     YES
    24        a             514.23        15.52987       YES     YES
    25        a             516.22         2.41516       YES     YES
    26        a             599.58         0.05682       YES     YES
    27        a             599.87         0.21034       YES     YES
    28        a             676.27        21.91013       YES     YES
    29        a             677.48         0.50544       YES     YES
    30        a             769.14       138.42570       YES     YES
    31        a             772.65         0.38643       YES     YES
    32        a             803.99         4.45164       YES     YES
    33        a             804.76        24.87644       YES     YES
    34        a             848.85        10.04239       YES     YES
    35        a             855.17         2.27867       YES     YES
    36        a             893.79        11.91838       YES     YES
    37        a             897.45         0.46306       YES     YES
    38        a             948.17         0.55406       YES     YES
    39        a             952.26         3.09563       YES     YES
    40        a             981.66         1.47177       YES     YES
    41        a             982.26         0.69858       YES     YES
```

42	a	984.35	6.00230	YES	YES
43	a	985.83	4.67101	YES	YES
44	a	1005.81	1.29299	YES	YES
45	a	1006.37	0.68301	YES	YES
46	a	1051.06	3.50926	YES	YES
47	a	1052.06	9.39486	YES	YES
48	a	1138.85	2.55362	YES	YES
49	a	1139.83	6.18019	YES	YES
50	a	1156.66	2.29351	YES	YES
51	a	1157.12	4.81769	YES	YES
52	a	1231.34	31.27461	YES	YES
53	a	1236.35	141.70508	YES	YES
54	a	1288.69	0.75928	YES	YES
55	a	1289.33	4.96746	YES	YES
56	a	1335.94	9.16440	YES	YES
57	a	1338.39	2.74425	YES	YES
58	a	1425.62	3.44511	YES	YES
59	a	1427.53	8.48216	YES	YES
60	a	1467.72	15.08968	YES	YES
61	a	1469.49	166.97136	YES	YES
62	a	1558.50	7.56031	YES	YES
63	a	1560.63	78.05694	YES	YES
64	a	1572.11	6.95544	YES	YES
65	a	1572.81	43.84675	YES	YES
66	a	3104.59	0.99937	YES	YES
67	a	3105.53	0.93693	YES	YES
68	a	3126.80	0.36595	YES	YES
69	a	3127.11	0.74323	YES	YES
70	a	3127.74	5.16916	YES	YES
71	a	3128.00	6.59360	YES	YES
72	a	3139.30	1.67547	YES	YES
73	a	3139.46	3.05082	YES	YES
74	a	3144.74	0.46492	YES	YES
75	a	3144.85	1.57965	YES	YES

$end

[Ag(1FB)$_3$]$^+$

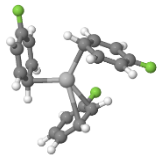

Method: (RI-)BP86(D3BJ)/def2-TZVPP
Symmetry: c1

Cartesian coordinates in Ångström:

C	1.4775885	2.7642858	-1.4874722
C	2.4294923	1.7721156	-1.2442730
C	2.2500498	0.5161346	-1.8237184
C	1.1203668	0.2562556	-2.6341258
C	0.1720722	1.2837001	-2.8532164
C	0.3503185	2.5450884	-2.2777157
C	-0.4080345	-3.1883743	-2.8532311
C	-1.0228751	-2.9142867	-1.6256030
C	-0.2569273	-2.8834845	-0.4344772
C	1.1232047	-3.1419554	-0.5273924
C	1.7444490	-3.4164915	-1.7409275
C	0.9652680	-3.4436580	-2.9031709
C	-2.2190838	-0.7003433	1.9612073
C	-1.9368482	0.3627918	1.0988288
C	-0.6257604	0.8911015	1.0198087
C	0.3993024	0.3263577	1.8156240
C	0.1227404	-0.7376204	2.6730317
C	-1.1844051	-1.2244408	2.7349811
F	-1.4541902	-2.2424038	3.5701372
F	1.6550161	3.9767592	-0.9351100
F	1.8518750	-3.1449755	0.6058304
Ag	-0.1500437	-0.5206338	-0.8007844
H	3.2988875	1.9989996	-0.6298144
H	3.0009448	-0.2596386	-1.6800012

```
H    -0.6750520    1.1068440   -3.5150734
H    -0.3562430    3.3553820   -2.4480829
H    -1.0056919   -3.2270326   -3.7620809
H    -2.1032036   -2.7851692   -1.5651394
H     2.8115556   -3.6310144   -1.7625119
H     1.4394811   -3.6803565   -3.8548529
H    -3.2237114   -1.1088954    2.0539397
H    -2.7417521    0.8229708    0.5267416
H     1.4020641    0.7498900    1.7834342
H     0.8940602   -1.1831692    3.2980214
H     1.0598387   -0.6754367   -3.1987829
H    -0.7284204   -2.8291674    0.5473456
H    -0.4421330    1.8188712    0.4757264

SCF energy GEOOPT =  -1141.903878213 H
ZPE =   714.9 kJ/mol
FREEH energy =   772.40 kJ/mol
FREEH entropy =   0.74174 kJ/mol/K

$vibrational spectrum
#  mode     symmetry     wave number   IR intensity    selection rules
#                         cm**(-1)        km/mol         IR     RAMAN
     1                      -0.00         0.00000        -        -
     2                       0.00         0.00000        -        -
     3                       0.00         0.00000        -        -
     4                       0.00         0.00000        -        -
     5                       0.00         0.00000        -        -
     6                       0.00         0.00000        -        -
     7                       0.46         0.52326        -        -
     8         a             9.30         0.02083       YES      YES
     9         a            18.62         0.39468       YES      YES
    10         a            33.30         0.26890       YES      YES
    11         a            34.59         0.98957       YES      YES
    12         a            45.36         0.39667       YES      YES
    13         a            60.22         0.44395       YES      YES
    14         a            62.54         0.04459       YES      YES
    15         a            68.17         0.31533       YES      YES
    16         a            74.33         0.13889       YES      YES
    17         a            77.02         0.49630       YES      YES
    18         a            85.38         0.01064       YES      YES
    19         a           155.35         0.51421       YES      YES
    20         a           159.80         2.76401       YES      YES
    21         a           163.34         1.17971       YES      YES
    22         a           248.78         1.18371       YES      YES
    23         a           271.25         2.31666       YES      YES
    24         a           276.07         0.73892       YES      YES
    25         a           394.93         1.70397       YES      YES
    26         a           398.24         0.21652       YES      YES
    27         a           398.38         4.09491       YES      YES
    28         a           409.92         0.33630       YES      YES
    29         a           410.58         0.05101       YES      YES
    30         a           415.50         0.11465       YES      YES
    31         a           501.76        17.04350       YES      YES
    32         a           502.43         9.30214       YES      YES
    33         a           503.96         4.66536       YES      YES
    34         a           508.46         7.41473       YES      YES
    35         a           509.21         2.08607       YES      YES
    36         a           511.32         8.70382       YES      YES
    37         a           603.66         0.70047       YES      YES
    38         a           604.01         0.36124       YES      YES
    39         a           604.42         0.06183       YES      YES
    40         a           680.99         9.56697       YES      YES
    41         a           682.28         9.94329       YES      YES
    42         a           684.85        13.19443       YES      YES
    43         a           758.94        82.53633       YES      YES
    44         a           783.85       148.28288       YES      YES
    45         a           786.60        60.38389       YES      YES
    46         a           804.02        14.61905       YES      YES
    47         a           809.41        22.05149       YES      YES
    48         a           809.58        11.90404       YES      YES
    49         a           820.08         0.90435       YES      YES
    50         a           822.76         1.01738       YES      YES
    51         a           835.13        10.55117       YES      YES
    52         a           891.85         5.96343       YES      YES
```

53	a	892.68	10.01807	YES	YES
54	a	894.39	9.85052	YES	YES
55	a	953.96	2.21368	YES	YES
56	a	955.93	0.23551	YES	YES
57	a	957.10	0.38996	YES	YES
58	a	959.91	10.67070	YES	YES
59	a	962.67	6.90468	YES	YES
60	a	982.42	0.22779	YES	YES
61	a	987.86	3.85278	YES	YES
62	a	988.57	2.08960	YES	YES
63	a	990.23	0.83953	YES	YES
64	a	1002.83	1.59598	YES	YES
65	a	1002.93	4.27072	YES	YES
66	a	1010.80	0.88699	YES	YES
67	a	1054.51	5.51852	YES	YES
68	a	1057.19	0.72125	YES	YES
69	a	1057.78	5.31189	YES	YES
70	a	1139.51	6.76017	YES	YES
71	a	1140.91	1.76552	YES	YES
72	a	1143.20	4.98378	YES	YES
73	a	1145.37	23.64063	YES	YES
74	a	1146.12	11.25402	YES	YES
75	a	1155.91	1.88996	YES	YES
76	a	1224.55	59.11930	YES	YES
77	a	1237.54	171.37527	YES	YES
78	a	1238.84	63.64778	YES	YES
79	a	1288.93	1.10514	YES	YES
80	a	1290.46	1.29172	YES	YES
81	a	1290.98	0.90514	YES	YES
82	a	1338.75	1.19737	YES	YES
83	a	1344.43	0.37428	YES	YES
84	a	1346.18	1.12397	YES	YES
85	a	1434.37	3.31496	YES	YES
86	a	1436.47	6.06291	YES	YES
87	a	1436.86	3.64649	YES	YES
88	a	1471.87	109.08543	YES	YES
89	a	1472.56	30.45869	YES	YES
90	a	1473.97	88.86997	YES	YES
91	a	1564.02	7.28890	YES	YES
92	a	1564.83	6.54494	YES	YES
93	a	1565.79	15.36179	YES	YES
94	a	1578.23	129.50262	YES	YES
95	a	1579.31	55.82703	YES	YES
96	a	1581.40	19.42256	YES	YES
97	a	3098.57	1.74209	YES	YES
98	a	3099.62	0.65823	YES	YES
99	a	3107.94	2.41081	YES	YES
100	a	3117.98	1.67453	YES	YES
101	a	3120.20	0.28050	YES	YES
102	a	3120.63	0.22724	YES	YES
103	a	3122.69	0.25556	YES	YES
104	a	3123.32	0.33866	YES	YES
105	a	3125.58	0.35940	YES	YES
106	a	3136.89	1.08624	YES	YES
107	a	3140.94	1.44154	YES	YES
108	a	3141.31	0.16799	YES	YES
109	a	3141.32	2.23761	YES	YES
110	a	3141.89	0.43767	YES	YES
111	a	3145.62	1.63302	YES	YES

$end

{(1FB)Ag[*pf*]}$_{ip.}$

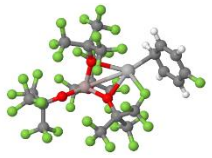

Method: (RI-)BP86(D3BJ)/def2-TZVPP
Symmetry: c1

Cartesian coordinates in Ångström:

Al	5.8616218	15.6216793	6.0846387
Ag	5.6387022	17.6605965	3.8350769
H	7.7865681	20.4964802	1.4187686
F	7.3773342	18.9221019	-0.5972946
H	5.1419053	17.6273197	-0.3867409
C	4.7578023	19.3473609	2.5644461
H	3.9893844	19.5940627	3.2988912
C	5.9647173	20.0897084	2.5273664
H	6.1871721	20.7953400	3.3251503
C	6.8512176	19.9423046	1.4627603
C	6.5206139	19.0602962	0.4343819
C	5.3475115	18.3106416	0.4343880
C	4.4607381	18.4580202	1.5017826
H	3.5200559	17.9113556	1.5037237
O	6.5372386	14.5657433	7.2525860
C	6.5021894	13.6347017	8.2459256
C	5.2143992	13.7854400	9.1537750
F	4.1483991	13.1876176	8.5807868
F	5.3832464	13.2367127	10.3757808
F	4.9089236	15.0879567	9.3148515
C	7.7899767	13.8149466	9.1485443
F	8.8609086	14.0896535	8.3824506
F	7.6260362	14.8417832	10.0131578
F	8.0656914	12.7084119	9.8737081
C	6.5044059	12.1796286	7.6316536
F	7.7262895	11.8653259	7.1487483
F	6.1664671	11.2397446	8.5412207
F	5.6248289	12.1078392	6.6123075
O	6.0692540	17.3776479	6.3508819
C	6.8437050	18.3468713	6.9329630
C	6.0423250	19.7097195	6.8976325
F	5.0666059	19.7201988	7.8206040
F	6.8352041	20.7808876	7.0994477
F	5.4447154	19.8787341	5.6858275
C	8.2017310	18.5068102	6.1449734
F	8.7504762	17.3024052	5.9167476
F	7.9700922	19.0775646	4.9239561
F	9.1013383	19.2727772	6.7847651
C	7.1743443	18.0082803	8.4399075
F	8.1599301	17.0915704	8.5070399
F	7.5787749	19.1025449	9.1232885
F	6.0889551	17.5088600	9.0490364
O	6.6770990	15.6072204	4.4662769
C	7.2957219	14.8383585	3.5058652
C	8.3384690	15.7309695	2.7158206
F	9.4503195	15.9338142	3.4421253
F	8.6886204	15.1853299	1.5373213
F	7.8168061	16.9615452	2.4441667
C	6.2120146	14.2807083	2.5049354
F	5.1698911	13.7759956	3.1832769
F	5.7386939	15.2961978	1.7280954
F	6.6911992	13.3271917	1.6857362
C	8.0854460	13.6301326	4.1434162
F	7.2329280	12.6434618	4.4795591
F	8.9935477	13.1171722	3.2842446
F	8.7304470	14.0319670	5.2497088
O	4.1732902	15.3661165	5.7688530
C	2.8601217	15.6822064	5.6871146
C	2.3897153	15.5911721	4.1791267
F	2.2715655	14.3140467	3.7811333
F	1.2182368	16.2192534	3.9496223
F	3.3173250	16.1735640	3.3634652
C	2.5867934	17.1364308	6.2335307
F	3.2393242	17.3340843	7.3879754
F	3.0508110	18.0660129	5.3416541
F	1.2806501	17.3925836	6.4325777
C	1.9985244	14.6512660	6.5284004
F	2.0712431	14.9343798	7.8452026
F	0.6916975	14.6815855	6.1723508
F	2.4550818	13.4045082	6.3377377

SCF energy GEOOPT = -5228.776567911 H
ZPE = 799.6 kJ/mol

FREEH energy =   970.77 kJ/mol
FREEH entropy =   1.53030 kJ/mol/K

$vibrational spectrum

# mode	symmetry	wave number cm**(-1)	IR intensity km/mol	selection rules IR	RAMAN
1		-0.00	0.00000	-	-
2		-0.00	0.00000	-	-
3		0.00	0.00000	-	-
4		0.00	0.00000	-	-
5		0.00	0.00000	-	-
6		0.00	0.00000	-	-
7	a	8.06	0.02990	YES	YES
8	a	16.72	0.08985	YES	YES
9	a	21.90	0.06469	YES	YES
10	a	23.24	0.04824	YES	YES
11	a	29.63	0.04477	YES	YES
12	a	30.12	0.08260	YES	YES
13	a	31.93	0.15355	YES	YES
14	a	33.81	0.22491	YES	YES
15	a	35.50	0.27747	YES	YES
16	a	37.24	0.59768	YES	YES
17	a	43.56	0.12045	YES	YES
18	a	48.09	0.05125	YES	YES
19	a	52.42	1.63095	YES	YES
20	a	57.96	1.25043	YES	YES
21	a	59.47	1.54900	YES	YES
22	a	61.43	0.50623	YES	YES
23	a	63.44	0.54995	YES	YES
24	a	66.28	0.63446	YES	YES
25	a	68.23	0.17557	YES	YES
26	a	70.47	0.73573	YES	YES
27	a	73.33	0.83039	YES	YES
28	a	74.93	0.52246	YES	YES
29	a	76.18	0.08164	YES	YES
30	a	79.04	0.45289	YES	YES
31	a	81.44	0.32708	YES	YES
32	a	84.13	0.88055	YES	YES
33	a	85.61	0.11162	YES	YES
34	a	87.05	1.75651	YES	YES
35	a	88.65	1.10124	YES	YES
36	a	91.83	0.34345	YES	YES
37	a	94.46	0.59993	YES	YES
38	a	95.62	0.03653	YES	YES
39	a	99.26	0.08022	YES	YES
40	a	101.36	0.27466	YES	YES
41	a	105.68	4.52002	YES	YES
42	a	108.49	0.17292	YES	YES
43	a	111.50	0.51112	YES	YES
44	a	121.67	0.70849	YES	YES
45	a	155.70	0.08888	YES	YES
46	a	159.74	0.17538	YES	YES
47	a	162.23	0.13696	YES	YES
48	a	165.88	0.83764	YES	YES
49	a	167.34	0.25813	YES	YES
50	a	167.99	0.22832	YES	YES
51	a	171.03	0.38994	YES	YES
52	a	172.94	0.39117	YES	YES
53	a	180.79	0.04792	YES	YES
54	a	194.69	4.36699	YES	YES
55	a	195.90	4.02278	YES	YES
56	a	201.43	3.71855	YES	YES
57	a	222.97	0.03794	YES	YES
58	a	258.23	2.16489	YES	YES
59	a	261.10	0.98778	YES	YES
60	a	269.15	1.32141	YES	YES
61	a	271.16	4.18566	YES	YES
62	a	273.34	1.70930	YES	YES
63	a	276.72	1.71063	YES	YES
64	a	279.44	0.01021	YES	YES
65	a	280.54	0.64816	YES	YES
66	a	280.72	0.89431	YES	YES
67	a	280.92	1.35086	YES	YES
68	a	281.80	0.33692	YES	YES

69	a	283.88	1.69787	YES	YES
70	a	292.30	0.98813	YES	YES
71	a	300.05	5.61067	YES	YES
72	a	300.96	3.25621	YES	YES
73	a	302.28	8.38016	YES	YES
74	a	305.42	1.33085	YES	YES
75	a	306.19	0.12400	YES	YES
76	a	309.08	0.58781	YES	YES
77	a	313.30	0.68004	YES	YES
78	a	315.52	1.50273	YES	YES
79	a	316.63	0.57902	YES	YES
80	a	317.54	1.55253	YES	YES
81	a	319.73	0.63334	YES	YES
82	a	320.17	0.70999	YES	YES
83	a	336.97	0.17172	YES	YES
84	a	339.74	1.47574	YES	YES
85	a	344.57	2.94012	YES	YES
86	a	345.66	6.44141	YES	YES
87	a	353.47	8.68820	YES	YES
88	a	355.95	7.77671	YES	YES
89	a	375.55	49.04885	YES	YES
90	a	379.93	8.74831	YES	YES
91	a	397.88	2.21689	YES	YES
92	a	412.69	25.46259	YES	YES
93	a	414.47	0.06334	YES	YES
94	a	422.40	53.08272	YES	YES
95	a	450.16	86.34664	YES	YES
96	a	503.79	3.80345	YES	YES
97	a	505.70	19.81862	YES	YES
98	a	508.00	1.97729	YES	YES
99	a	508.49	4.31320	YES	YES
100	a	509.97	1.11215	YES	YES
101	a	510.53	0.51078	YES	YES
102	a	510.85	3.47063	YES	YES
103	a	511.60	2.23490	YES	YES
104	a	512.57	0.77569	YES	YES
105	a	512.73	2.05587	YES	YES
106	a	514.09	3.20195	YES	YES
107	a	516.22	10.96979	YES	YES
108	a	518.29	3.63444	YES	YES
109	a	519.51	2.92849	YES	YES
110	a	520.35	0.52011	YES	YES
111	a	522.54	1.16021	YES	YES
112	a	531.22	20.34344	YES	YES
113	a	543.03	7.08477	YES	YES
114	a	543.85	6.85512	YES	YES
115	a	544.81	0.60927	YES	YES
116	a	545.33	1.88334	YES	YES
117	a	545.57	0.72171	YES	YES
118	a	545.81	0.15840	YES	YES
119	a	548.63	0.79904	YES	YES
120	a	548.96	1.14747	YES	YES
121	a	551.30	23.01321	YES	YES
122	a	605.22	0.15283	YES	YES
123	a	684.51	8.74932	YES	YES
124	a	695.15	3.31648	YES	YES
125	a	696.57	10.57964	YES	YES
126	a	697.14	15.47775	YES	YES
127	a	697.56	52.90276	YES	YES
128	a	698.13	26.70435	YES	YES
129	a	699.42	43.33132	YES	YES
130	a	700.67	30.62474	YES	YES
131	a	701.44	29.54061	YES	YES
132	a	710.53	35.72988	YES	YES
133	a	714.19	3.29899	YES	YES
134	a	722.09	2.12428	YES	YES
135	a	727.65	3.68683	YES	YES
136	a	761.36	10.03458	YES	YES
137	a	766.77	7.12129	YES	YES
138	a	784.96	135.68143	YES	YES
139	a	805.16	12.43181	YES	YES
140	a	808.70	22.45722	YES	YES
141	a	812.61	0.55958	YES	YES
142	a	846.11	14.62229	YES	YES

143	a	886.59	6.07080	YES	YES
144	a	918.52	89.99752	YES	YES
145	a	926.98	5.64047	YES	YES
146	a	928.82	5.60809	YES	YES
147	a	934.33	9.57381	YES	YES
148	a	935.16	227.58085	YES	YES
149	a	935.70	132.24347	YES	YES
150	a	937.15	169.98799	YES	YES
151	a	940.86	362.66898	YES	YES
152	a	953.75	4.02283	YES	YES
153	a	959.61	9.63449	YES	YES
154	a	988.13	0.84527	YES	YES
155	a	1003.44	2.69509	YES	YES
156	a	1038.46	2.87560	YES	YES
157	a	1046.50	2.55283	YES	YES
158	a	1051.12	5.86915	YES	YES
159	a	1056.04	4.39058	YES	YES
160	a	1062.38	1.02585	YES	YES
161	a	1082.56	27.04398	YES	YES
162	a	1089.92	4.64049	YES	YES
163	a	1097.59	95.26839	YES	YES
164	a	1100.69	5.21485	YES	YES
165	a	1103.96	6.36803	YES	YES
166	a	1111.26	8.42316	YES	YES
167	a	1119.26	19.29216	YES	YES
168	a	1122.10	19.56751	YES	YES
169	a	1134.40	18.85980	YES	YES
170	a	1137.18	20.93083	YES	YES
171	a	1137.65	18.69351	YES	YES
172	a	1140.64	21.25480	YES	YES
173	a	1144.46	139.09834	YES	YES
174	a	1146.43	46.74414	YES	YES
175	a	1147.96	57.91837	YES	YES
176	a	1151.62	250.15270	YES	YES
177	a	1154.05	24.10191	YES	YES
178	a	1158.01	69.09654	YES	YES
179	a	1158.76	15.91273	YES	YES
180	a	1165.28	8.90639	YES	YES
181	a	1168.28	404.66175	YES	YES
182	a	1173.36	290.95704	YES	YES
183	a	1177.06	156.25813	YES	YES
184	a	1182.54	242.91869	YES	YES
185	a	1191.45	971.31844	YES	YES
186	a	1194.96	1140.27153	YES	YES
187	a	1195.33	1287.42379	YES	YES
188	a	1199.38	128.98944	YES	YES
189	a	1199.75	178.77733	YES	YES
190	a	1201.73	130.30208	YES	YES
191	a	1210.04	749.50322	YES	YES
192	a	1219.30	133.55323	YES	YES
193	a	1221.35	603.64718	YES	YES
194	a	1221.84	546.99068	YES	YES
195	a	1234.23	105.85257	YES	YES
196	a	1259.41	5.43192	YES	YES
197	a	1268.11	26.30451	YES	YES
198	a	1292.18	1.21565	YES	YES
199	a	1300.46	262.27060	YES	YES
200	a	1314.39	125.56493	YES	YES
201	a	1349.33	0.28571	YES	YES
202	a	1437.33	4.86667	YES	YES
203	a	1472.59	78.78381	YES	YES
204	a	1566.84	6.07669	YES	YES
205	a	1581.79	100.53366	YES	YES
206	a	3102.49	0.19513	YES	YES
207	a	3133.54	0.30398	YES	YES
208	a	3134.39	0.15495	YES	YES
209	a	3146.04	0.23566	YES	YES
210	a	3147.70	0.62049	YES	YES

$end

$[Ag(2FB)_1]^+$

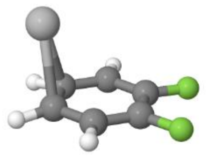

Method: (RI-)BP86(D3BJ)/def2-TZVPP
Symmetry: cs

Cartesian coordinates in Ångström:

Ag	1.7464320	-1.8986853	0.0000000
C	-0.3259106	-1.0859441	-0.7174912
H	-0.6698450	-1.9681961	-1.2598628
F	0.2696564	2.4436166	1.3405111
C	-0.3259106	-1.0859441	0.7174912
H	-0.6698450	-1.9681961	1.2598628
F	0.2696564	2.4436166	-1.3405111
C	-0.1061492	0.1177977	1.4207102
H	-0.1265203	0.1505996	2.5088544
C	0.0843181	1.2918766	0.7077225
C	0.0843181	1.2918766	-0.7077225
C	-0.1061492	0.1177977	-1.4207102
H	-0.1265203	0.1505996	-2.5088544

SCF energy GEOOPT = -577.7972800795 H
ZPE = 217.0 kJ/mol
FREEH energy = 238.62 kJ/mol
FREEH entropy = 0.39524 kJ/mol/K

$vibrational spectrum

# mode	symmetry	wave number	IR intensity	selection rules	
#		cm**(-1)	km/mol	IR	RAMAN
1		-0.00	0.00000	-	-
2		-0.00	0.00000	-	-
3		-0.00	0.00000	-	-
4		-0.00	0.00000	-	-
5		0.00	0.00000	-	-
6		0.00	0.00000	-	-
7	a"	40.05	0.79398	YES	YES
8	a'	63.03	0.33294	YES	YES
9	a"	176.90	0.32901	YES	YES
10	a'	201.81	0.40343	YES	YES
11	a'	283.68	0.26663	YES	YES
12	a'	347.45	0.35958	YES	YES
13	a"	428.92	0.02662	YES	YES
14	a'	444.49	1.63802	YES	YES
15	a"	536.30	3.75961	YES	YES
16	a"	554.02	2.61343	YES	YES
17	a'	555.42	8.62208	YES	YES
18	a"	691.44	0.57408	YES	YES
19	a'	758.23	14.55909	YES	YES
20	a'	816.72	75.24502	YES	YES
21	a"	836.93	11.16578	YES	YES
22	a"	837.62	10.45850	YES	YES
23	a'	918.56	8.43779	YES	YES
24	a"	940.88	4.03916	YES	YES
25	a'	979.93	20.74767	YES	YES
26	a"	1084.49	12.16650	YES	YES
27	a'	1136.69	6.94353	YES	YES
28	a"	1216.37	23.25462	YES	YES
29	a"	1260.24	13.27597	YES	YES
30	a'	1281.37	137.99748	YES	YES
31	a'	1356.04	4.15721	YES	YES
32	a"	1424.49	15.58770	YES	YES
33	a'	1493.25	289.10482	YES	YES
34	a'	1534.20	101.57387	YES	YES
35	a"	1574.57	36.93476	YES	YES
36	a"	3102.61	2.09284	YES	YES
37	a'	3112.76	6.34308	YES	YES
38	a"	3140.72	17.67577	YES	YES
39	a'	3142.04	1.74872	YES	YES

$end

[Ag(2FB)$_2$]$^+$

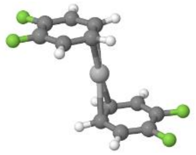

```
Method: (RI-)BP86(D3BJ)/def2-TZVPP
Symmetry: c2

Cartesian coordinates in Ångström:
C     2.1129996   -0.7397056    0.8633465
H     2.4101519    0.0873423    1.5084157
F     1.5479221   -4.0340676   -1.5671970
C     2.1571598   -0.5922436   -0.5514518
H     2.4888033    0.3479282   -0.9926870
F     1.4626874   -4.3127132    1.1078816
C     1.9559509   -1.7157653   -1.3828658
H     2.0087666   -1.6348625   -2.4672848
C     1.7295811   -2.9563140   -0.8053392
C     1.6850697   -3.1024164    0.5974675
C     1.8674126   -2.0089630    1.4311748
H     1.8521171   -2.1537368    2.5101060
Ag   -0.0000000   -0.0000000    0.1531260
C    -2.1571598    0.5922436   -0.5514518
H    -2.4888033   -0.3479282   -0.9926870
F    -1.4626874    4.3127132    1.1078816
C    -2.1129996    0.7397056    0.8633465
H    -2.4101519   -0.0873423    1.5084157
F    -1.5479221    4.0340676   -1.5671970
C    -1.8674126    2.0089630    1.4311748
H    -1.8521171    2.1537368    2.5101060
C    -1.6850697    3.1024164    0.5974675
C    -1.7295811    2.9563140   -0.8053392
C    -1.9559509    1.7157653   -1.3828658
H    -2.0087666    1.6348625   -2.4672848

SCF energy GEOOPT =  -1008.783601810 H
ZPE =   434.8 kJ/mol
FREEH energy =   477.71 kJ/mol
FREEH entropy =   0.59418 kJ/mol/K

$vibrational spectrum
#  mode     symmetry     wave number   IR intensity    selection rules
#                         cm**(-1)        km/mol          IR     RAMAN
     1                      -0.00         0.00000         -        -
     2                      -0.00         0.00000         -        -
     3                      -0.00         0.00000         -        -
     4                       0.00         0.00000         -        -
     5                       0.00         0.00000         -        -
     6                       0.00         0.00000         -        -
     7         a             2.74         1.41388        YES      YES
     8         a            21.59         0.93004        YES      YES
     9         b            22.72         1.86359        YES      YES
    10         b            45.41         0.01661        YES      YES
    11         a            45.49         0.01920        YES      YES
    12         a            50.25         0.20640        YES      YES
    13         b            85.21         0.74969        YES      YES
    14         a           180.26         0.00650        YES      YES
    15         b           183.88         0.00109        YES      YES
    16         a           189.41         0.08521        YES      YES
    17         b           220.70         0.77312        YES      YES
    18         b           281.84         0.49076        YES      YES
    19         a           281.91         0.00655        YES      YES
    20         a           335.76         0.00495        YES      YES
    21         b           336.94         0.27393        YES      YES
    22         a           430.25         0.24049        YES      YES
    23         b           430.57         0.00667        YES      YES
    24         b           441.54         3.34371        YES      YES
    25         a           443.28         0.00363        YES      YES
    26         a           536.42         6.64755        YES      YES
    27         b           536.45         0.06010        YES      YES
```

28	a	558.74	0.10196	YES	YES
29	b	559.12	11.38342	YES	YES
30	a	563.24	1.84498	YES	YES
31	b	564.59	0.03359	YES	YES
32	b	692.32	0.00374	YES	YES
33	a	693.71	0.12358	YES	YES
34	a	758.45	0.44340	YES	YES
35	b	758.53	37.39617	YES	YES
36	b	803.68	165.01302	YES	YES
37	a	807.93	0.00011	YES	YES
38	b	837.39	0.17945	YES	YES
39	a	839.28	27.97507	YES	YES
40	b	839.28	0.28936	YES	YES
41	a	841.38	3.67759	YES	YES
42	a	916.87	0.07522	YES	YES
43	b	917.17	10.58254	YES	YES
44	b	948.33	0.01723	YES	YES
45	a	951.13	1.81082	YES	YES
46	b	991.32	33.26569	YES	YES
47	a	993.66	0.14716	YES	YES
48	b	1084.35	0.21171	YES	YES
49	a	1084.69	19.67097	YES	YES
50	b	1137.71	6.67550	YES	YES
51	a	1138.20	0.00802	YES	YES
52	b	1210.96	0.68682	YES	YES
53	a	1211.02	56.46378	YES	YES
54	b	1255.74	0.06939	YES	YES
55	a	1255.91	22.47176	YES	YES
56	b	1271.65	342.43787	YES	YES
57	a	1275.49	2.30208	YES	YES
58	b	1349.79	12.16627	YES	YES
59	a	1351.37	0.00313	YES	YES
60	b	1428.09	0.26412	YES	YES
61	a	1428.41	33.34036	YES	YES
62	b	1490.97	548.20426	YES	YES
63	a	1493.65	5.32198	YES	YES
64	b	1548.70	116.78790	YES	YES
65	a	1550.82	0.97982	YES	YES
66	b	1579.81	0.61440	YES	YES
67	a	1579.84	48.33828	YES	YES
68	a	3110.61	1.25273	YES	YES
69	b	3110.85	0.05469	YES	YES
70	b	3121.24	3.20258	YES	YES
71	a	3121.45	0.06813	YES	YES
72	b	3140.22	0.32078	YES	YES
73	a	3140.24	18.81813	YES	YES
74	b	3141.81	2.45912	YES	YES
75	a	3141.88	0.18919	YES	YES

$end

$[Ag(2FB)_3]^+$

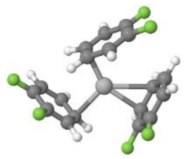

Method: (RI-)BP86(D3BJ)/def2-TZVPP
Symmetry: c1

Cartesian coordinates in Ångström:

Ag	-0.6454815	-0.4360756	0.0134288
F	1.5860328	2.5520625	-3.1180242
C	0.8288597	2.3481087	-2.0333378
C	-0.5631736	2.2772754	-2.1833864
F	-1.0935519	2.4042491	-3.4068381
C	-1.3809886	2.0572261	-1.0815409
H	-2.4618561	2.0515873	-1.2138401
C	-0.7919429	1.9001920	0.1958075
H	-1.4269532	1.8981647	1.0826057
C	0.6154738	1.9686977	0.3346117

```
H     1.0693213    1.9034353    1.3216381
C     1.4216518    2.1882485   -0.7849925
H     2.5051841    2.2527793   -0.7023902
F     1.4395512    0.4151724    3.7207223
C     0.2489042   -0.0368778    3.3027206
C    -0.9083998    0.6904592    3.6205011
F    -0.7896811    1.8118462    4.3395934
C    -2.1591167    0.2607576    3.1851756
H    -3.0391957    0.8408441    3.4588454
C    -2.2588890   -0.9101545    2.4287867
H    -3.2378754   -1.2716451    2.1191131
C    -1.1026723   -1.6535254    2.1170281
H    -1.1912739   -2.6387156    1.6603074
C     0.1639307   -1.2115341    2.5625097
H     1.0661353   -1.7933237    2.3786817
F     1.4385326   -3.3356767    0.1326430
C     1.2083831   -2.4862432   -0.8809542
C    -0.1028341   -2.3103125   -1.3589857
H    -0.8837210   -2.9868403   -1.0102221
C    -0.3224813   -1.4283416   -2.4476191
H    -1.3215591   -1.3429062   -2.8719215
C     0.7582401   -0.7542630   -3.0304096
H     0.5935379   -0.0984779   -3.8829492
C     2.0521844   -0.9393292   -2.5428844
H     2.9049906   -0.4292488   -2.9884498
C     2.2735643   -1.7922706   -1.4624907
F     3.5071694   -1.9653441   -0.9734832

SCF energy GEOOPT =  -1439.741958982 H
ZPE =   652.2 kJ/mol
FREEH energy =   716.64 kJ/mol
FREEH entropy =   0.76772 kJ/mol/K

$vibrational spectrum
#  mode     symmetry     wave number   IR intensity    selection rules
#                         cm**(-1)        km/mol         IR     RAMAN
     1                        0.00         0.00000        -       -
     2                        0.00         0.00000        -       -
     3                        0.00         0.00000        -       -
     4                        0.00         0.00000        -       -
     5                        0.00         0.00000        -       -
     6                        0.00         0.00000        -       -
     7         a              6.62         0.72864       YES     YES
     8         a             12.90         0.22564       YES     YES
     9         a             20.43         0.56181       YES     YES
    10         a             32.43         0.75411       YES     YES
    11         a             38.27         1.37159       YES     YES
    12         a             41.36         0.18771       YES     YES
    13         a             51.41         0.19445       YES     YES
    14         a             52.70         0.40189       YES     YES
    15         a             57.95         0.01939       YES     YES
    16         a             61.22         0.18243       YES     YES
    17         a             66.18         3.85586       YES     YES
    18         a             80.88         0.71275       YES     YES
    19         a            123.44         3.83469       YES     YES
    20         a            139.78         0.40991       YES     YES
    21         a            161.93         0.20450       YES     YES
    22         a            198.15         2.18109       YES     YES
    23         a            211.90         0.41009       YES     YES
    24         a            223.27         1.16306       YES     YES
    25         a            278.98         0.25631       YES     YES
    26         a            280.22         0.24433       YES     YES
    27         a            281.31         0.21805       YES     YES
    28         a            292.12         0.95552       YES     YES
    29         a            300.58         0.32636       YES     YES
    30         a            318.79         0.54092       YES     YES
    31         a            429.07         0.05767       YES     YES
    32         a            430.44         0.35773       YES     YES
    33         a            430.66         0.01608       YES     YES
    34         a            450.03         4.68327       YES     YES
    35         a            450.31         1.82406       YES     YES
    36         a            463.42         3.10380       YES     YES
    37         a            535.89         2.72034       YES     YES
    38         a            536.88         4.01601       YES     YES
```

39	a	537.97	2.57490	YES	YES
40	a	551.38	0.65086	YES	YES
41	a	554.74	0.11129	YES	YES
42	a	559.04	2.06683	YES	YES
43	a	561.90	6.30935	YES	YES
44	a	562.58	1.73271	YES	YES
45	a	563.17	1.52676	YES	YES
46	a	693.95	1.50322	YES	YES
47	a	696.78	0.57342	YES	YES
48	a	697.91	0.71674	YES	YES
49	a	753.43	18.39490	YES	YES
50	a	758.40	13.47185	YES	YES
51	a	759.59	35.83084	YES	YES
52	a	771.61	117.53233	YES	YES
53	a	782.54	122.47700	YES	YES
54	a	788.00	54.60696	YES	YES
55	a	837.21	5.08978	YES	YES
56	a	838.82	8.12514	YES	YES
57	a	841.64	21.20624	YES	YES
58	a	842.67	4.81440	YES	YES
59	a	848.27	15.71377	YES	YES
60	a	855.66	4.12425	YES	YES
61	a	918.85	5.53258	YES	YES
62	a	922.01	9.70060	YES	YES
63	a	927.02	7.33179	YES	YES
64	a	954.84	5.53059	YES	YES
65	a	957.78	1.22982	YES	YES
66	a	966.86	0.85644	YES	YES
67	a	1003.62	11.74698	YES	YES
68	a	1008.46	12.25656	YES	YES
69	a	1011.91	6.02377	YES	YES
70	a	1088.24	12.30841	YES	YES
71	a	1089.07	7.33232	YES	YES
72	a	1090.51	16.49001	YES	YES
73	a	1140.20	2.24239	YES	YES
74	a	1141.16	7.56117	YES	YES
75	a	1147.30	0.82320	YES	YES
76	a	1194.75	25.18371	YES	YES
77	a	1201.54	32.42074	YES	YES
78	a	1202.40	25.18176	YES	YES
79	a	1243.22	29.58939	YES	YES
80	a	1250.56	7.84428	YES	YES
81	a	1252.14	27.92383	YES	YES
82	a	1267.37	83.23828	YES	YES
83	a	1268.42	187.51057	YES	YES
84	a	1273.02	57.98257	YES	YES
85	a	1337.37	4.20275	YES	YES
86	a	1343.91	0.71015	YES	YES
87	a	1347.50	1.51362	YES	YES
88	a	1434.34	5.11985	YES	YES
89	a	1436.23	19.58658	YES	YES
90	a	1437.41	17.48798	YES	YES
91	a	1483.86	148.35427	YES	YES
92	a	1490.59	277.14951	YES	YES
93	a	1490.89	124.85028	YES	YES
94	a	1565.81	14.96149	YES	YES
95	a	1568.90	26.89863	YES	YES
96	a	1570.13	37.26220	YES	YES
97	a	1579.87	37.07823	YES	YES
98	a	1581.29	24.91886	YES	YES
99	a	1585.20	32.21268	YES	YES
100	a	3105.27	1.36445	YES	YES
101	a	3112.33	1.68335	YES	YES
102	a	3115.68	1.05662	YES	YES
103	a	3126.01	1.13290	YES	YES
104	a	3129.77	0.32737	YES	YES
105	a	3130.45	0.14392	YES	YES
106	a	3132.79	4.63864	YES	YES
107	a	3133.19	1.77968	YES	YES
108	a	3134.14	4.18331	YES	YES
109	a	3141.14	1.50883	YES	YES
110	a	3142.74	2.59671	YES	YES
111	a	3144.24	1.95816	YES	YES

$end

{(2FB)Ag[*pf*]}$_{ip.}$

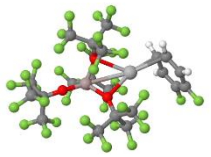

```
Method: (RI-)BP86(D3BJ)/def2-TZVPP
Symmetry: c1

Cartesian coordinates in Ångström:
Al     5.9042440   15.6625037    6.1167285
Ag     5.7176545   17.7121637    3.8629778
H      7.7623738   19.7762410    0.9129387
F      6.6187086   18.3512286   -0.9381321
F      4.0885281   17.5468371   -0.4363288
C      4.7974678   19.4222095    2.6143991
H      4.2528558   19.8415906    3.4607996
C      6.1236284   19.8398374    2.3426514
H      6.6454409   20.4831517    3.0481843
C      6.7421189   19.4748981    1.1420280
C      6.0418812   18.7107057    0.2175412
C      4.7281890   18.2916198    0.4781912
C      4.0995365   18.6419904    1.6632990
H      3.0732988   18.3263536    1.8362902
O      6.5965306   14.6361386    7.3040063
C      6.5556412   13.6302156    8.2220120
C      5.3654471   13.8325394    9.2420836
F      4.1955988   13.4438511    8.6893111
F      5.5388598   13.1293924   10.3812756
F      5.2447642   15.1339159    9.5661445
C      7.9212631   13.6206421    9.0231525
F      8.9536146   13.8506762    8.1924841
F      7.9337531   14.5864561    9.9681740
F      8.1336579   12.4379058    9.6431909
C      6.3686221   12.2294297    7.5120254
F      7.5280576   11.8062769    6.9629561
F      5.9463891   11.2720555    8.3649984
F      5.4586616   12.3373933    6.5215105
O      6.0319039   17.4239656    6.4138802
C      6.7858502   18.3661140    7.0657757
C      6.1343988   19.7875258    6.8318924
F      5.0069966   19.9292833    7.5474249
F      6.9710723   20.7954450    7.1409716
F      5.7886641   19.9501331    5.5210572
C      8.2597846   18.3593760    6.5065991
F      8.7163860   17.0957551    6.4344030
F      8.2780582   18.8666912    5.2401786
F      9.1178808   19.0797745    7.2483977
C      6.8176589   18.1046476    8.6247322
F      7.6573231   17.0937893    8.9148881
F      7.2297513   19.1962915    9.3090321
F      5.5916115   17.7757548    9.0603583
O      6.7466152   15.6796936    4.5091049
C      7.4352285   14.9706845    3.5480314
C      8.5770738   15.8875046    2.9450291
F      9.6044665   16.0057688    3.8020116
F      9.0455175   15.4218856    1.7733377
F      8.1088034   17.1470310    2.7130302
C      6.4361954   14.5460553    2.4032700
F      5.3315126   13.9973698    2.9277587
F      6.0470516   15.6519978    1.7011958
F      6.9725846   13.6775873    1.5295912
C      8.1153851   13.6788829    4.1421330
F      7.1912044   12.7201514    4.3463207
F      9.0540748   13.1805708    3.3083072
F      8.7016183   13.9637304    5.3142351
O      4.2274743   15.3702756    5.7779143
C      2.9170689   15.6757850    5.6353200
C      2.5117366   15.5392194    4.1128469
```

```
F     2.4426183   14.2504756    3.7435484
F     1.3352325   16.1317612    3.8189888
F     3.4568796   16.1285428    3.3239305
C     2.6085209   17.1422030    6.1295713
F     3.1930108   17.3761345    7.3113989
F     3.1169159   18.0545495    5.2395314
F     1.2919711   17.3958036    6.2463258
C     2.0309420   14.6614856    6.4733266
F     2.0453814   14.9867509    7.7826294
F     0.7401125   14.6652668    6.0623937
F     2.5080559   13.4149862    6.3436331

SCF energy GEOOPT =  -5328.056813192 H
ZPE =   778.3 kJ/mol
FREEH energy =   951.91 kJ/mol
FREEH entropy =   1.55581 kJ/mol/K

$vibrational spectrum
#  mode     symmetry     wave number   IR intensity    selection rules
#                         cm**(-1)        km/mol         IR     RAMAN
     1                      -0.00         0.00000         -       -
     2                       0.00         0.00000         -       -
     3                       0.00         0.00000         -       -
     4                       0.00         0.00000         -       -
     5                       0.00         0.00000         -       -
     6                       0.00         0.00000         -       -
     7        a              4.02         0.02448        YES     YES
     8        a             16.33         0.02290        YES     YES
     9        a             18.83         0.08346        YES     YES
    10        a             24.58         0.01882        YES     YES
    11        a             26.36         0.00767        YES     YES
    12        a             28.27         0.22657        YES     YES
    13        a             32.18         0.26926        YES     YES
    14        a             34.48         0.17063        YES     YES
    15        a             35.84         0.07856        YES     YES
    16        a             38.32         1.01157        YES     YES
    17        a             46.49         0.92450        YES     YES
    18        a             46.69         0.00527        YES     YES
    19        a             49.26         0.25605        YES     YES
    20        a             53.58         2.25839        YES     YES
    21        a             57.59         0.23041        YES     YES
    22        a             59.61         0.18353        YES     YES
    23        a             60.85         0.15572        YES     YES
    24        a             64.25         0.09562        YES     YES
    25        a             66.11         0.25685        YES     YES
    26        a             66.99         1.52653        YES     YES
    27        a             69.83         0.33605        YES     YES
    28        a             73.21         0.18437        YES     YES
    29        a             76.11         0.59130        YES     YES
    30        a             76.76         1.76307        YES     YES
    31        a             79.39         0.49402        YES     YES
    32        a             81.35         1.05719        YES     YES
    33        a             84.79         1.43469        YES     YES
    34        a             86.38         0.40361        YES     YES
    35        a             89.01         0.52875        YES     YES
    36        a             90.28         1.85673        YES     YES
    37        a             93.51         0.12506        YES     YES
    38        a             95.20         0.19791        YES     YES
    39        a             99.43         0.00859        YES     YES
    40        a            101.71         0.13549        YES     YES
    41        a            104.47         0.12247        YES     YES
    42        a            106.58         4.04885        YES     YES
    43        a            109.21         0.81285        YES     YES
    44        a            121.46         1.26408        YES     YES
    45        a            157.31         0.07065        YES     YES
    46        a            160.40         0.28859        YES     YES
    47        a            162.43         0.18630        YES     YES
    48        a            164.37         1.04154        YES     YES
    49        a            166.53         0.21172        YES     YES
    50        a            168.06         0.05280        YES     YES
    51        a            170.68         0.71762        YES     YES
    52        a            174.36         0.09197        YES     YES
    53        a            175.04         0.07877        YES     YES
    54        a            195.74         3.99424        YES     YES
```

55	a	196.01	4.25505	YES	YES
56	a	201.72	3.89247	YES	YES
57	a	216.89	0.05131	YES	YES
58	a	224.14	0.09678	YES	YES
59	a	257.77	1.87325	YES	YES
60	a	263.12	0.91513	YES	YES
61	a	268.51	1.35689	YES	YES
62	a	271.97	4.81836	YES	YES
63	a	273.92	1.15071	YES	YES
64	a	276.04	1.74329	YES	YES
65	a	278.83	1.12570	YES	YES
66	a	279.09	0.20949	YES	YES
67	a	280.27	0.15428	YES	YES
68	a	280.49	0.15334	YES	YES
69	a	280.60	0.07989	YES	YES
70	a	281.65	0.85180	YES	YES
71	a	284.51	1.78534	YES	YES
72	a	300.03	5.56799	YES	YES
73	a	301.06	3.94768	YES	YES
74	a	302.40	8.29999	YES	YES
75	a	305.59	1.11972	YES	YES
76	a	307.03	0.73974	YES	YES
77	a	309.45	0.11746	YES	YES
78	a	312.37	0.78712	YES	YES
79	a	314.47	1.24102	YES	YES
80	a	315.65	1.01017	YES	YES
81	a	316.33	0.85456	YES	YES
82	a	319.21	0.82161	YES	YES
83	a	319.70	0.83472	YES	YES
84	a	323.78	0.25291	YES	YES
85	a	336.59	0.14437	YES	YES
86	a	340.52	2.02326	YES	YES
87	a	343.57	2.44612	YES	YES
88	a	346.34	4.94040	YES	YES
89	a	353.52	8.95332	YES	YES
90	a	354.68	8.63814	YES	YES
91	a	375.82	38.74063	YES	YES
92	a	383.40	22.65469	YES	YES
93	a	417.44	26.61413	YES	YES
94	a	423.38	47.26421	YES	YES
95	a	430.63	0.47169	YES	YES
96	a	448.64	1.41834	YES	YES
97	a	451.22	85.32807	YES	YES
98	a	503.54	0.91082	YES	YES
99	a	508.05	2.49325	YES	YES
100	a	510.10	1.98265	YES	YES
101	a	510.73	3.24577	YES	YES
102	a	511.08	0.44344	YES	YES
103	a	512.12	0.47542	YES	YES
104	a	512.50	3.54358	YES	YES
105	a	512.79	3.54478	YES	YES
106	a	514.58	1.89978	YES	YES
107	a	516.33	9.42596	YES	YES
108	a	518.15	4.29604	YES	YES
109	a	518.87	2.32620	YES	YES
110	a	521.14	0.70346	YES	YES
111	a	523.09	1.56903	YES	YES
112	a	531.36	19.04456	YES	YES
113	a	536.84	4.06621	YES	YES
114	a	543.39	3.61933	YES	YES
115	a	544.04	3.73347	YES	YES
116	a	544.99	2.23668	YES	YES
117	a	545.10	2.00176	YES	YES
118	a	545.90	0.37176	YES	YES
119	a	546.71	0.17903	YES	YES
120	a	548.27	0.54110	YES	YES
121	a	548.70	0.54106	YES	YES
122	a	551.96	29.49793	YES	YES
123	a	560.44	5.86979	YES	YES
124	a	565.08	1.18369	YES	YES
125	a	686.36	1.08639	YES	YES
126	a	694.85	6.02123	YES	YES
127	a	696.75	14.92380	YES	YES
128	a	697.29	23.33856	YES	YES

129	a	697.98	38.36714	YES	YES
130	a	698.30	26.73484	YES	YES
131	a	699.80	45.00578	YES	YES
132	a	700.81	30.90633	YES	YES
133	a	701.23	23.61063	YES	YES
134	a	710.24	41.60397	YES	YES
135	a	713.80	3.40015	YES	YES
136	a	721.98	2.05717	YES	YES
137	a	728.02	3.51509	YES	YES
138	a	758.66	24.54343	YES	YES
139	a	760.71	10.20349	YES	YES
140	a	765.11	10.70062	YES	YES
141	a	783.12	122.83249	YES	YES
142	a	804.29	11.40482	YES	YES
143	a	832.82	14.23930	YES	YES
144	a	840.67	14.91190	YES	YES
145	a	844.61	14.09797	YES	YES
146	a	913.45	3.84817	YES	YES
147	a	918.48	99.39098	YES	YES
148	a	926.84	5.26323	YES	YES
149	a	928.21	6.80017	YES	YES
150	a	934.21	63.32796	YES	YES
151	a	934.89	238.93306	YES	YES
152	a	935.45	70.23706	YES	YES
153	a	937.87	180.25332	YES	YES
154	a	941.17	337.55947	YES	YES
155	a	949.95	17.90831	YES	YES
156	a	1001.97	9.62916	YES	YES
157	a	1036.84	3.45673	YES	YES
158	a	1044.97	7.19356	YES	YES
159	a	1048.99	5.15694	YES	YES
160	a	1062.15	0.61415	YES	YES
161	a	1081.45	34.79319	YES	YES
162	a	1088.49	4.86960	YES	YES
163	a	1089.76	6.40165	YES	YES
164	a	1097.55	99.52427	YES	YES
165	a	1101.20	7.15297	YES	YES
166	a	1104.71	6.59571	YES	YES
167	a	1111.76	5.98952	YES	YES
168	a	1119.83	16.25000	YES	YES
169	a	1124.87	32.96955	YES	YES
170	a	1134.22	22.55578	YES	YES
171	a	1136.33	8.15216	YES	YES
172	a	1137.09	13.53986	YES	YES
173	a	1141.22	85.71146	YES	YES
174	a	1144.17	134.06320	YES	YES
175	a	1146.64	51.89238	YES	YES
176	a	1150.05	231.64377	YES	YES
177	a	1153.60	19.63875	YES	YES
178	a	1156.78	96.61702	YES	YES
179	a	1158.45	11.56186	YES	YES
180	a	1164.26	54.14604	YES	YES
181	a	1166.93	357.96961	YES	YES
182	a	1171.68	342.67412	YES	YES
183	a	1177.11	99.66000	YES	YES
184	a	1182.42	193.45509	YES	YES
185	a	1191.34	1196.80157	YES	YES
186	a	1193.36	1113.28730	YES	YES
187	a	1196.74	979.75351	YES	YES
188	a	1199.25	75.38391	YES	YES
189	a	1200.19	331.95074	YES	YES
190	a	1202.25	269.83797	YES	YES
191	a	1203.59	260.26894	YES	YES
192	a	1210.95	567.12970	YES	YES
193	a	1219.08	258.13502	YES	YES
194	a	1221.36	446.59241	YES	YES
195	a	1222.15	448.39856	YES	YES
196	a	1251.37	12.40082	YES	YES
197	a	1259.26	10.68468	YES	YES
198	a	1265.00	16.18365	YES	YES
199	a	1268.37	144.98662	YES	YES
200	a	1298.39	246.39263	YES	YES
201	a	1313.41	124.70077	YES	YES
202	a	1349.71	0.25176	YES	YES

203	a	1435.14	15.81052	YES	YES
204	a	1490.89	209.07185	YES	YES
205	a	1567.34	45.36945	YES	YES
206	a	1585.18	24.72676	YES	YES
207	a	3112.09	0.22890	YES	YES
208	a	3132.60	0.05877	YES	YES
209	a	3144.81	1.01846	YES	YES
210	a	3149.78	2.22982	YES	YES

$end

$[Ag(3FB)_1]^+$

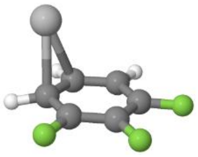

Method: (RI-)BP86(D3BJ)/def2-TZVPP
Symmetry: c1

Cartesian coordinates in Ångström:

Ag	1.7611916	-1.9326393	-0.1455221
C	-0.2981806	-1.1027003	-0.6761713
H	-0.6909799	-1.9560814	-1.2332631
F	0.2443332	2.4528003	1.3579029
C	-0.3348205	-1.0735097	0.7602278
H	-0.6552485	-1.9642026	1.3010450
F	0.2867146	2.4426581	-1.3342612
C	-0.1316604	0.1259976	1.4536448
H	-0.1692387	0.1705129	2.5403581
C	0.0698518	1.2985186	0.7304296
C	0.0974036	1.3081552	-0.6807030
C	-0.0853074	0.1106324	-1.3726338
F	-0.0940588	0.1198582	-2.7010537

SCF energy GEOOPT = -677.0733958544 H
ZPE = 195.9 kJ/mol
FREEH energy = 220.06 kJ/mol
FREEH entropy = 0.42035 kJ/mol/K

$vibrational spectrum

# mode	symmetry	wave number cm**(-1)	IR intensity km/mol	selection rules IR	RAMAN
1		0.00	0.00000	-	-
2		0.00	0.00000	-	-
3		0.00	0.00000	-	-
4		0.00	0.00000	-	-
5		0.00	0.00000	-	-
6		0.00	0.00000	-	-
7	a	17.93	0.66950	YES	YES
8	a	61.84	0.47775	YES	YES
9	a	135.72	0.31245	YES	YES
10	a	170.88	0.23415	YES	YES
11	a	272.01	0.08841	YES	YES
12	a	281.56	0.86600	YES	YES
13	a	301.16	1.51305	YES	YES
14	a	338.40	0.54596	YES	YES
15	a	463.02	0.03366	YES	YES
16	a	485.78	4.56173	YES	YES
17	a	535.68	1.93090	YES	YES
18	a	562.16	2.99555	YES	YES
19	a	584.66	0.39202	YES	YES
20	a	682.53	13.63247	YES	YES
21	a	696.12	1.98841	YES	YES
22	a	799.80	49.00446	YES	YES
23	a	817.14	15.39964	YES	YES
24	a	871.97	17.65713	YES	YES
25	a	929.37	8.43974	YES	YES
26	a	1017.11	43.52776	YES	YES
27	a	1031.14	66.91400	YES	YES
28	a	1144.16	2.09102	YES	YES

```
    29        a            1230.82        56.02726       YES     YES
    30        a            1249.08        38.92110       YES     YES
    31        a            1295.61        79.93541       YES     YES
    32        a            1354.18         1.57789       YES     YES
    33        a            1440.65        89.72118       YES     YES
    34        a            1497.59       317.41000       YES     YES
    35        a            1538.75        81.16440       YES     YES
    36        a            1575.25       115.34659       YES     YES
    37        a            3101.86        11.80467       YES     YES
    38        a            3119.94         8.22614       YES     YES
    39        a            3147.52        12.16238       YES     YES
$end
```

$[Ag(3FB)_2]^+$

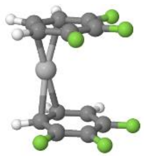

```
Method: (RI-)BP86(D3BJ)/def2-TZVPP
Symmetry: c1

Cartesian coordinates in Ångström:
C    -2.4982140    0.9878011   -0.7182021
C    -2.0855043    1.4651501    0.5276185
C    -2.4251732    0.7418730    1.6872858
C    -3.1583385   -0.4374484    1.6204006
C    -3.5806359   -0.9108766    0.3644045
C    -3.2454259   -0.2033366   -0.8268930
H    -3.7022031   -0.4498566   -1.7855598
H    -4.2753133   -1.7486988    0.3104795
H    -3.4190457   -0.9562929    2.5406486
F    -2.0132931    1.2165305    2.8602495
F    -1.3778078    2.5838984    0.6200339
F    -2.1919957    1.6829595   -1.8129177
C     1.7811803   -0.9532950   -1.0317750
C     1.7378345   -1.2889181    0.3352763
C     0.9786110   -2.3877971    0.7430296
C     0.2580715   -3.1587311   -0.1929616
C     0.3262642   -2.7995388   -1.5708770
C     1.0830849   -1.6877494   -1.9837215
H     1.1586210   -1.4091940   -3.0328818
H    -0.1051604   -3.4649295   -2.3182005
H    -0.1718245   -4.1072548    0.1295848
F     0.9544860   -2.7161213    2.0341139
F     2.4077456   -0.5656840    1.2236510
F     2.5075564    0.1021890   -1.3911535
Ag   -1.6255199   -1.8570788   -0.5933333

SCF energy GEOOPT =  -1207.338995808 H
ZPE =   392.8 kJ/mol
FREEH energy =   440.55 kJ/mol
FREEH entropy =   0.63424 kJ/mol/K

$vibrational spectrum
#  mode     symmetry     wave number   IR intensity    selection rules
#                         cm**(-1)        km/mol          IR     RAMAN
     1                       0.00         0.00000         -       -
     2                       0.00         0.00000         -       -
     3                       0.00         0.00000         -       -
     4                       0.00         0.00000         -       -
     5                       0.00         0.00000         -       -
     6                       0.00         0.00000         -       -
     7        a              5.55         0.19934       YES     YES
     8        a             11.84         0.00120       YES     YES
     9        a             19.59         0.39519       YES     YES
    10        a             37.99         0.07059       YES     YES
    11        a             42.21         0.28562       YES     YES
    12        a             50.94         2.35896       YES     YES
    13        a             82.20         0.00203       YES     YES
```

14	a	130.88	0.03720	YES	YES
15	a	139.29	0.61112	YES	YES
16	a	174.47	0.15049	YES	YES
17	a	204.25	0.00136	YES	YES
18	a	271.19	0.10372	YES	YES
19	a	271.45	0.07686	YES	YES
20	a	273.45	0.49244	YES	YES
21	a	282.12	0.03454	YES	YES
22	a	299.29	2.24368	YES	YES
23	a	299.46	0.56918	YES	YES
24	a	328.44	0.15715	YES	YES
25	a	330.42	0.63658	YES	YES
26	a	466.14	0.00482	YES	YES
27	a	466.23	0.04255	YES	YES
28	a	487.83	3.69144	YES	YES
29	a	488.18	0.62819	YES	YES
30	a	541.43	0.00818	YES	YES
31	a	542.95	2.07272	YES	YES
32	a	562.42	0.47375	YES	YES
33	a	562.72	4.72108	YES	YES
34	a	579.02	0.13670	YES	YES
35	a	580.93	1.03959	YES	YES
36	a	682.97	5.49722	YES	YES
37	a	683.76	21.92892	YES	YES
38	a	700.16	2.03577	YES	YES
39	a	700.25	4.10270	YES	YES
40	a	795.10	121.38468	YES	YES
41	a	799.72	0.65821	YES	YES
42	a	817.47	7.75239	YES	YES
43	a	818.07	12.30505	YES	YES
44	a	865.55	7.52226	YES	YES
45	a	867.12	2.60738	YES	YES
46	a	934.24	0.00332	YES	YES
47	a	938.18	9.41194	YES	YES
48	a	1021.89	34.15179	YES	YES
49	a	1022.26	95.23688	YES	YES
50	a	1029.71	55.86442	YES	YES
51	a	1030.45	7.36930	YES	YES
52	a	1142.14	3.75412	YES	YES
53	a	1142.32	0.39831	YES	YES
54	a	1230.60	0.09271	YES	YES
55	a	1231.63	96.57879	YES	YES
56	a	1242.28	2.24092	YES	YES
57	a	1244.24	56.47946	YES	YES
58	a	1289.84	51.04981	YES	YES
59	a	1295.89	84.77814	YES	YES
60	a	1342.31	7.30664	YES	YES
61	a	1344.75	0.04129	YES	YES
62	a	1445.34	10.06404	YES	YES
63	a	1445.80	152.08892	YES	YES
64	a	1491.67	62.44867	YES	YES
65	a	1499.03	447.65965	YES	YES
66	a	1551.96	87.70458	YES	YES
67	a	1553.05	30.93834	YES	YES
68	a	1577.74	1.49970	YES	YES
69	a	1579.85	126.69226	YES	YES
70	a	3118.28	1.89230	YES	YES
71	a	3118.36	3.48102	YES	YES
72	a	3127.41	5.61210	YES	YES
73	a	3127.53	17.52917	YES	YES
74	a	3147.58	12.71528	YES	YES
75	a	3147.64	5.66519	YES	YES

$end

$[Ag(3FB)_3]^+$

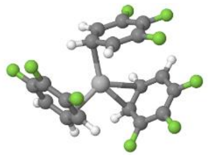

Method: (RI-)BP86(D3BJ)/def2-TZVPP
Symmetry: c1

Cartesian coordinates in Ångström:

Ag	-0.6315600	-0.4308427	0.0153163
F	1.2604708	2.6103269	-3.2980823
C	0.6157582	2.3847351	-2.1555763
C	-0.7821666	2.3062016	-2.1362782
F	-1.4422272	2.4519955	-3.2900091
C	-1.4742200	2.0659681	-0.9519563
H	-2.5625668	2.0685628	-0.9592359
C	-0.7433000	1.8999808	0.2464581
H	-1.2727434	1.8999085	1.1994962
C	0.6711278	1.9573905	0.2391545
H	1.2509302	1.8871185	1.1576377
C	1.3303014	2.1936416	-0.9654020
F	2.6642374	2.2413957	-1.0087354
F	1.4277736	0.4355051	3.7042384
C	0.2413928	-0.0348366	3.2998871
C	-0.9121304	0.6842347	3.6396741
F	-0.8224809	1.8060323	4.3506241
C	-2.1598888	0.2169005	3.2024039
F	-3.2493949	0.9242151	3.5194959
C	-2.2684562	-0.9484319	2.4476679
H	-3.2557212	-1.3058153	2.1611891
C	-1.1003486	-1.6676022	2.1287194
H	-1.1832095	-2.6509260	1.6674490
C	0.1657123	-1.2093248	2.5533482
H	1.0720346	-1.7819232	2.3645749
F	1.2391487	-3.4199312	0.1385211
C	1.1410728	-2.5358419	-0.8649244
C	-0.1221348	-2.2404735	-1.4168810
H	-0.9709987	-2.8721623	-1.1532170
C	-0.1899033	-1.3324343	-2.5040698
H	-1.1466630	-1.1578742	-2.9928684
C	0.9685561	-0.7343671	-3.0069834
H	0.9390728	-0.0574847	-3.8581859
C	2.1989216	-1.0401579	-2.4311581
F	3.3179891	-0.4843744	-2.8929600
C	2.3015215	-1.9304474	-1.3486069
F	3.4840725	-2.1988515	-0.8007053

SCF energy GEOOPT = -1737.577888171 H
ZPE =   589.0 kJ/mol
FREEH energy =   660.71 kJ/mol
FREEH entropy =   0.82978 kJ/mol/K

$vibrational spectrum

# mode	symmetry	wave number cm**(-1)	IR intensity km/mol	selection rules IR	RAMAN
1		-0.00	0.00000	-	-
2		0.00	0.00000	-	-
3		0.00	0.00000	-	-
4		0.00	0.00000	-	-
5		0.00	0.00000	-	-
6		0.00	0.00000	-	-
7	a	5.23	0.50314	YES	YES
8	a	10.05	0.50603	YES	YES
9	a	14.76	0.62824	YES	YES
10	a	27.02	0.13902	YES	YES
11	a	34.66	0.40231	YES	YES
12	a	35.97	0.08773	YES	YES
13	a	40.80	1.71862	YES	YES
14	a	49.42	0.06336	YES	YES
15	a	51.54	0.12245	YES	YES
16	a	52.91	0.05288	YES	YES
17	a	62.26	3.81828	YES	YES
18	a	70.80	0.47580	YES	YES
19	a	115.70	3.87110	YES	YES
20	a	132.20	0.31734	YES	YES
21	a	142.51	0.73963	YES	YES
22	a	154.74	1.08096	YES	YES
23	a	161.85	0.13459	YES	YES
24	a	198.07	0.22267	YES	YES

25	a	248.24	0.18986	YES	YES
26	a	251.88	0.18530	YES	YES
27	a	270.03	0.07043	YES	YES
28	a	271.40	0.06188	YES	YES
29	a	272.18	0.07768	YES	YES
30	a	284.97	2.21932	YES	YES
31	a	296.85	0.99860	YES	YES
32	a	297.89	0.64250	YES	YES
33	a	299.42	1.43384	YES	YES
34	a	309.40	0.40392	YES	YES
35	a	316.84	0.42761	YES	YES
36	a	329.55	0.42549	YES	YES
37	a	467.69	0.26201	YES	YES
38	a	468.03	0.23925	YES	YES
39	a	468.65	0.05304	YES	YES
40	a	489.79	1.30528	YES	YES
41	a	490.82	1.56603	YES	YES
42	a	490.93	1.84785	YES	YES
43	a	535.72	0.17559	YES	YES
44	a	541.32	0.74647	YES	YES
45	a	546.52	0.95266	YES	YES
46	a	562.99	1.91526	YES	YES
47	a	563.64	2.08423	YES	YES
48	a	563.68	1.03574	YES	YES
49	a	572.52	1.05774	YES	YES
50	a	573.24	0.72253	YES	YES
51	a	579.64	0.20525	YES	YES
52	a	684.35	13.51293	YES	YES
53	a	686.17	13.36599	YES	YES
54	a	686.84	19.49579	YES	YES
55	a	699.57	9.02838	YES	YES
56	a	700.41	12.31001	YES	YES
57	a	702.09	0.52429	YES	YES
58	a	780.40	119.52206	YES	YES
59	a	791.36	114.77057	YES	YES
60	a	793.40	27.65834	YES	YES
61	a	818.16	12.28152	YES	YES
62	a	818.97	8.69678	YES	YES
63	a	819.88	9.73877	YES	YES
64	a	858.82	1.14439	YES	YES
65	a	864.62	4.65902	YES	YES
66	a	865.39	1.95546	YES	YES
67	a	930.50	7.94989	YES	YES
68	a	936.63	6.51912	YES	YES
69	a	941.90	4.83120	YES	YES
70	a	1015.65	92.64495	YES	YES
71	a	1018.17	43.05719	YES	YES
72	a	1020.11	117.48104	YES	YES
73	a	1035.75	20.36018	YES	YES
74	a	1037.39	12.14041	YES	YES
75	a	1040.75	17.23723	YES	YES
76	a	1137.65	10.71970	YES	YES
77	a	1140.10	1.39578	YES	YES
78	a	1144.56	1.59925	YES	YES
79	a	1228.39	40.71643	YES	YES
80	a	1230.37	24.12248	YES	YES
81	a	1231.37	56.16691	YES	YES
82	a	1234.90	32.07862	YES	YES
83	a	1236.95	29.22489	YES	YES
84	a	1237.27	11.40012	YES	YES
85	a	1287.30	86.97152	YES	YES
86	a	1288.77	128.75381	YES	YES
87	a	1296.87	49.48984	YES	YES
88	a	1341.56	0.40508	YES	YES
89	a	1343.61	0.83813	YES	YES
90	a	1346.92	0.16974	YES	YES
91	a	1450.67	67.60844	YES	YES
92	a	1451.52	43.85379	YES	YES
93	a	1456.26	96.18083	YES	YES
94	a	1492.40	218.56596	YES	YES
95	a	1494.59	266.81648	YES	YES
96	a	1497.84	243.06731	YES	YES
97	a	1561.21	41.05451	YES	YES
98	a	1568.62	94.11346	YES	YES

99	a	1570.61	102.29831	YES	YES
100	a	1578.84	0.27474	YES	YES
101	a	1582.38	4.50646	YES	YES
102	a	1587.09	62.51411	YES	YES
103	a	3111.42	1.28285	YES	YES
104	a	3113.96	3.09399	YES	YES
105	a	3120.26	0.67279	YES	YES
106	a	3131.65	1.84327	YES	YES
107	a	3137.07	5.02906	YES	YES
108	a	3138.14	4.47972	YES	YES
109	a	3141.15	5.52821	YES	YES
110	a	3143.44	4.89487	YES	YES
111	a	3147.68	5.47490	YES	YES

$end

{(3FB)Ag[*pf*]}$_{ip.}$

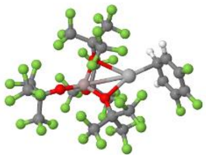

Method: (RI-)BP86(D3BJ)/def2-TZVPP
Symmetry: c1

Cartesian coordinates in Ångström:

Al	5.8826507	15.6498657	6.1149233
Ag	5.6636463	17.6921430	3.8707897
F	8.0278386	20.0678797	0.9854249
F	6.8635012	18.4527132	-0.8557125
F	4.3726327	17.4656602	-0.4386927
C	4.8200609	19.4251956	2.6068936
H	4.2173648	19.8195724	3.4253624
C	6.1206100	19.9399709	2.3885516
H	6.5879897	20.6210024	3.0960471
C	6.7918645	19.6027367	1.2175883
C	6.2016656	18.7730972	0.2572236
C	4.9170233	18.2670362	0.4875873
C	4.2160195	18.5832510	1.6459199
H	3.2067995	18.2026605	1.7805177
O	6.5851346	14.6269278	7.2981353
C	6.5515802	13.6274228	8.2238226
C	5.3555357	13.8228060	9.2383396
F	4.1928981	13.4115086	8.6869476
F	5.5360031	13.1338380	10.3848989
F	5.2154841	15.1258354	9.5483602
C	7.9143505	13.6386947	9.0297149
F	8.9460951	13.8786022	8.2011767
F	7.9106125	14.6084250	9.9708540
F	8.1398381	12.4615117	9.6554053
C	6.3833947	12.2205705	7.5220960
F	7.5492068	11.8097422	6.9776371
F	5.9720164	11.2631459	8.3801665
F	5.4733812	12.3110436	6.5295466
O	6.0179971	17.4135311	6.4017916
C	6.7826890	18.3614747	7.0334183
C	6.1078086	19.7762135	6.8261033
F	5.0069495	19.9064056	7.5833726
F	6.9445903	20.7929525	7.1028558
F	5.7106058	19.9342475	5.5286882
C	8.2377123	18.3721557	6.4265496
F	8.7078523	17.1147520	6.3415480
F	8.2069138	18.8767311	5.1586176
F	9.1097064	19.1055908	7.1378938
C	6.8682446	18.0949872	8.5890619
F	7.7277049	17.0923916	8.8478911
F	7.2909396	19.1891423	9.2624933
F	5.6607238	17.7514391	9.0632457
O	6.7070409	15.6618551	4.4984969
C	7.3781327	14.9446964	3.5312385
C	8.4749544	15.8721193	2.8648379

```
F     9.5252983   16.0413731    3.6834446
F     8.9147545   15.3869906    1.6912153
F     7.9662760   17.1144623    2.6140720
C     6.3518011   14.4655462    2.4335334
F     5.2742790   13.9154692    3.0112684
F     5.9207959   15.5397721    1.7087771
F     6.8774580   13.5775836    1.5726867
C     8.1117540   13.6860059    4.1345974
F     7.2229462   12.7027123    4.3747854
F     9.0485591   13.2017149    3.2907148
F     8.7118979   14.0111912    5.2892390
O     4.2010425   15.3643067    5.7935692
C     2.8897207   15.6726586    5.6662713
C     2.4632736   15.5285027    4.1500990
F     2.3831580   14.2384411    3.7890172
F     1.2864067   16.1254441    3.8683798
F     3.4014323   16.1084464    3.3445329
C     2.5920347   17.1429155    6.1559548
F     3.1948353   17.3828528    7.3271754
F     3.0901281   18.0487355    5.2520429
F     1.2786048   17.4016014    6.2900755
C     2.0115984   14.6660704    6.5215787
F     2.0485752   14.9965984    7.8290489
F     0.7146827   14.6751918    6.1306764
F     2.4802797   13.4167868    6.3887780

SCF energy GEOOPT =  -5427.336605281 H
ZPE =   757.2 kJ/mol
FREEH energy =   933.31 kJ/mol
FREEH entropy =   1.57732 kJ/mol/K

$vibrational spectrum
#  mode     symmetry     wave number   IR intensity    selection rules
#                         cm**(-1)        km/mol         IR     RAMAN
     1                      -0.00         0.00000        -       -
     2                       0.00         0.00000        -       -
     3                       0.00         0.00000        -       -
     4                       0.00         0.00000        -       -
     5                       0.00         0.00000        -       -
     6                       0.00         0.00000        -       -
     7        a              3.33         0.01617       YES     YES
     8        a             14.67         0.06733       YES     YES
     9        a             17.52         0.03978       YES     YES
    10        a             24.08         0.03285       YES     YES
    11        a             26.28         0.05029       YES     YES
    12        a             27.19         0.27965       YES     YES
    13        a             30.76         0.27104       YES     YES
    14        a             33.59         0.25621       YES     YES
    15        a             34.17         0.13379       YES     YES
    16        a             37.56         1.04682       YES     YES
    17        a             41.30         0.17714       YES     YES
    18        a             43.55         0.10595       YES     YES
    19        a             46.96         0.01781       YES     YES
    20        a             51.25         1.14184       YES     YES
    21        a             56.66         0.38118       YES     YES
    22        a             58.15         0.58621       YES     YES
    23        a             61.58         0.27187       YES     YES
    24        a             64.19         0.32865       YES     YES
    25        a             65.47         0.10718       YES     YES
    26        a             67.89         1.60972       YES     YES
    27        a             69.28         0.59786       YES     YES
    28        a             73.91         0.28025       YES     YES
    29        a             76.07         0.96236       YES     YES
    30        a             77.04         1.57469       YES     YES
    31        a             78.42         0.45594       YES     YES
    32        a             80.46         0.88860       YES     YES
    33        a             85.52         1.67725       YES     YES
    34        a             86.38         0.26106       YES     YES
    35        a             89.57         1.77957       YES     YES
    36        a             90.06         0.81439       YES     YES
    37        a             94.03         0.18108       YES     YES
    38        a             95.25         0.15495       YES     YES
    39        a             99.50         0.00291       YES     YES
    40        a            101.80         0.15729       YES     YES
```

41	a	105.03	0.11424	YES	YES
42	a	106.27	4.42614	YES	YES
43	a	110.00	0.41583	YES	YES
44	a	121.64	1.01342	YES	YES
45	a	136.83	1.24321	YES	YES
46	a	157.17	0.06182	YES	YES
47	a	161.04	0.14145	YES	YES
48	a	164.07	0.45646	YES	YES
49	a	165.26	0.82571	YES	YES
50	a	167.62	0.01379	YES	YES
51	a	170.07	0.54891	YES	YES
52	a	171.48	0.29356	YES	YES
53	a	173.72	0.18370	YES	YES
54	a	195.56	4.04078	YES	YES
55	a	195.77	4.19829	YES	YES
56	a	201.33	3.71633	YES	YES
57	a	208.88	0.36480	YES	YES
58	a	223.81	0.06612	YES	YES
59	a	253.74	0.11583	YES	YES
60	a	257.92	1.88952	YES	YES
61	a	263.17	0.90901	YES	YES
62	a	268.56	1.41489	YES	YES
63	a	272.13	1.24780	YES	YES
64	a	272.20	3.48826	YES	YES
65	a	273.97	1.22090	YES	YES
66	a	276.27	1.60978	YES	YES
67	a	279.15	0.09968	YES	YES
68	a	279.21	1.09219	YES	YES
69	a	280.47	0.17686	YES	YES
70	a	280.82	0.24805	YES	YES
71	a	281.67	0.88702	YES	YES
72	a	284.46	1.78331	YES	YES
73	a	297.13	0.88023	YES	YES
74	a	300.07	5.55947	YES	YES
75	a	301.19	3.93663	YES	YES
76	a	302.43	8.27663	YES	YES
77	a	305.62	1.23328	YES	YES
78	a	306.88	0.49838	YES	YES
79	a	309.54	0.12465	YES	YES
80	a	312.54	0.83118	YES	YES
81	a	314.76	1.25766	YES	YES
82	a	315.77	0.90434	YES	YES
83	a	316.49	1.14253	YES	YES
84	a	319.47	0.96264	YES	YES
85	a	320.32	0.72342	YES	YES
86	a	328.62	0.18809	YES	YES
87	a	336.65	0.15127	YES	YES
88	a	340.56	2.16827	YES	YES
89	a	343.66	2.42504	YES	YES
90	a	346.54	5.28965	YES	YES
91	a	353.81	9.09376	YES	YES
92	a	355.09	8.26747	YES	YES
93	a	376.31	38.69775	YES	YES
94	a	383.58	22.19256	YES	YES
95	a	417.16	26.85912	YES	YES
96	a	423.57	46.75171	YES	YES
97	a	451.40	82.67285	YES	YES
98	a	468.39	0.01878	YES	YES
99	a	490.28	1.34239	YES	YES
100	a	503.49	0.85776	YES	YES
101	a	507.84	2.49991	YES	YES
102	a	509.98	1.80709	YES	YES
103	a	510.58	2.86507	YES	YES
104	a	511.00	0.80720	YES	YES
105	a	512.17	0.87261	YES	YES
106	a	512.42	3.23816	YES	YES
107	a	512.83	3.45801	YES	YES
108	a	514.52	1.85303	YES	YES
109	a	516.32	9.66221	YES	YES
110	a	518.18	4.30752	YES	YES
111	a	518.94	2.20750	YES	YES
112	a	521.08	0.72631	YES	YES
113	a	522.93	1.24788	YES	YES
114	a	531.10	19.68078	YES	YES

115	a	543.30	2.47621	YES	YES
116	a	544.00	4.20226	YES	YES
117	a	544.90	2.18482	YES	YES
118	a	545.04	2.53772	YES	YES
119	a	545.74	0.34465	YES	YES
120	a	546.61	0.21287	YES	YES
121	a	548.43	0.66086	YES	YES
122	a	548.81	0.43342	YES	YES
123	a	551.63	4.15767	YES	YES
124	a	552.16	26.12830	YES	YES
125	a	562.67	1.00597	YES	YES
126	a	569.50	1.67594	YES	YES
127	a	683.67	28.27252	YES	YES
128	a	689.18	2.91893	YES	YES
129	a	694.75	6.88611	YES	YES
130	a	696.73	14.38323	YES	YES
131	a	697.31	24.27162	YES	YES
132	a	697.96	43.62035	YES	YES
133	a	698.26	24.41922	YES	YES
134	a	699.77	42.77756	YES	YES
135	a	700.91	27.97307	YES	YES
136	a	701.23	24.30716	YES	YES
137	a	710.24	41.33175	YES	YES
138	a	713.60	3.70912	YES	YES
139	a	721.73	2.07157	YES	YES
140	a	728.02	3.55595	YES	YES
141	a	761.11	10.48072	YES	YES
142	a	764.99	8.95277	YES	YES
143	a	788.74	112.49097	YES	YES
144	a	804.12	13.68508	YES	YES
145	a	817.46	12.46229	YES	YES
146	a	845.47	14.80499	YES	YES
147	a	857.69	0.46062	YES	YES
148	a	917.69	105.30945	YES	YES
149	a	926.55	5.52154	YES	YES
150	a	926.89	10.38736	YES	YES
151	a	931.40	24.14602	YES	YES
152	a	934.49	77.49095	YES	YES
153	a	935.05	140.60324	YES	YES
154	a	936.61	202.58622	YES	YES
155	a	938.81	117.95957	YES	YES
156	a	940.94	332.89665	YES	YES
157	a	1014.69	106.81731	YES	YES
158	a	1035.34	3.78896	YES	YES
159	a	1036.95	23.31055	YES	YES
160	a	1045.15	3.28728	YES	YES
161	a	1048.44	4.34436	YES	YES
162	a	1062.20	0.70451	YES	YES
163	a	1081.55	28.67848	YES	YES
164	a	1088.58	4.51594	YES	YES
165	a	1097.69	103.25246	YES	YES
166	a	1101.35	6.86333	YES	YES
167	a	1105.11	7.17621	YES	YES
168	a	1112.66	7.58894	YES	YES
169	a	1120.94	17.27079	YES	YES
170	a	1125.70	25.13846	YES	YES
171	a	1134.32	21.21871	YES	YES
172	a	1135.89	4.23190	YES	YES
173	a	1137.14	15.92730	YES	YES
174	a	1140.42	42.78199	YES	YES
175	a	1143.79	166.75140	YES	YES
176	a	1147.24	70.36584	YES	YES
177	a	1150.27	237.40571	YES	YES
178	a	1153.31	32.44311	YES	YES
179	a	1157.10	70.25566	YES	YES
180	a	1158.66	28.09143	YES	YES
181	a	1164.23	46.65973	YES	YES
182	a	1166.88	348.45509	YES	YES
183	a	1171.58	342.85037	YES	YES
184	a	1176.81	111.91804	YES	YES
185	a	1182.16	201.46584	YES	YES
186	a	1191.71	1166.05407	YES	YES
187	a	1193.92	1052.66190	YES	YES
188	a	1197.43	1083.60990	YES	YES

189	a	1200.56	392.64745	YES	YES
190	a	1202.51	256.30913	YES	YES
191	a	1203.47	196.72857	YES	YES
192	a	1211.09	577.44470	YES	YES
193	a	1219.44	268.91054	YES	YES
194	a	1221.81	410.20856	YES	YES
195	a	1222.44	460.46489	YES	YES
196	a	1228.53	69.16443	YES	YES
197	a	1231.29	27.41424	YES	YES
198	a	1259.49	7.77446	YES	YES
199	a	1264.79	14.79126	YES	YES
200	a	1287.69	106.94153	YES	YES
201	a	1298.46	240.22784	YES	YES
202	a	1313.36	121.19134	YES	YES
203	a	1348.01	0.02437	YES	YES
204	a	1452.63	77.85093	YES	YES
205	a	1494.70	263.33865	YES	YES
206	a	1570.27	91.43312	YES	YES
207	a	1585.88	4.92632	YES	YES
208	a	3114.42	0.40653	YES	YES
209	a	3147.71	3.54558	YES	YES
210	a	3155.09	4.26810	YES	YES

$end

$[Ag(4FB)_1]^+$

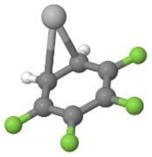

Method: (RI-)BP86(D3BJ)/def2-TZVPP
Symmetry: c1

Cartesian coordinates in Ångström:

C	0.7130902	1.0022604	0.0767927
C	1.3542696	-0.2197254	0.4757705
C	0.6140048	-1.2012768	1.1223567
C	-0.7470102	-0.9965219	1.4163842
C	-1.3867507	0.1990509	1.0438082
C	-0.6587631	1.1933637	0.3848277
F	-2.6659620	0.3762472	1.3313697
F	-1.4372882	-1.9308718	2.0400702
F	1.1784071	-2.3461083	1.4961720
Ag	0.6363391	0.1810739	-2.0147887
F	-1.2488641	2.3480168	0.0945439
H	1.3146142	1.8889064	-0.1418177
H	2.4310134	-0.3433151	0.3675107

SCF energy GEOOPT = -776.3478610592 H
ZPE = 174.9 kJ/mol
FREEH energy = 201.38 kJ/mol
FREEH entropy = 0.43538 kJ/mol/K

$vibrational spectrum

# mode	symmetry	wave number	IR intensity	selection rules	
#		cm**(-1)	km/mol	IR	RAMAN
1		-0.00	0.00000	-	-
2		-0.00	0.00000	-	-
3		0.00	0.00000	-	-
4		0.00	0.00000	-	-
5		0.00	0.00000	-	-
6		0.00	0.00000	-	-
7	a	27.68	0.04234	YES	YES
8	a	56.60	0.30490	YES	YES
9	a	120.71	1.13312	YES	YES
10	a	142.38	0.15060	YES	YES
11	a	227.12	0.86091	YES	YES
12	a	270.44	0.04776	YES	YES
13	a	277.05	0.53182	YES	YES
14	a	310.98	0.30591	YES	YES

15	a	317.05	2.04769	YES	YES
16	a	385.95	1.94491	YES	YES
17	a	440.73	0.81031	YES	YES
18	a	469.18	0.88043	YES	YES
19	a	544.15	1.10322	YES	YES
20	a	593.04	0.99764	YES	YES
21	a	623.00	3.74648	YES	YES
22	a	673.72	10.48972	YES	YES
23	a	698.50	0.54442	YES	YES
24	a	734.55	9.48531	YES	YES
25	a	825.21	46.44904	YES	YES
26	a	886.85	35.46654	YES	YES
27	a	987.00	90.86058	YES	YES
28	a	1033.97	56.66155	YES	YES
29	a	1158.58	33.43199	YES	YES
30	a	1217.93	74.82777	YES	YES
31	a	1276.22	55.95792	YES	YES
32	a	1282.07	10.38549	YES	YES
33	a	1354.03	0.45656	YES	YES
34	a	1468.06	280.44661	YES	YES
35	a	1488.95	343.21397	YES	YES
36	a	1550.78	101.22746	YES	YES
37	a	1574.53	47.42403	YES	YES
38	a	3088.43	16.81064	YES	YES
39	a	3133.06	17.12209	YES	YES

$end

$[Ag(4FB)_2]^+$

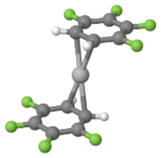

Method: (RI-)BP86(D3BJ)/def2-TZVPP
Symmetry: c2

Cartesian coordinates in Ångström:

C	2.4896487	2.5179445	-0.7420198
C	2.3688795	1.2899024	-1.4017918
F	2.6088134	3.6432678	-1.4312016
C	2.2394768	0.0951134	-0.6746078
C	2.2306156	0.1423638	0.7534348
C	2.3503346	1.3826845	1.4013225
C	2.4800785	2.5644301	0.6632274
Ag	0.0000000	-0.0000000	0.0259971
F	2.4018333	1.2637227	-2.7332334
F	2.5896972	3.7329635	1.2780903
F	2.3647983	1.4452901	2.7320085
H	2.3202913	-0.7693135	1.3441923
H	2.3376758	-0.8535591	-1.2024200
C	-2.4896487	-2.5179445	-0.7420198
C	-2.3688795	-1.2899024	-1.4017918
F	-2.6088134	-3.6432678	-1.4312016
C	-2.2394768	-0.0951134	-0.6746078
C	-2.2306156	-0.1423638	0.7534348
C	-2.3503346	-1.3826845	1.4013225
C	-2.4800785	-2.5644301	0.6632274
F	-2.4018333	-1.2637227	-2.7332334
F	-2.5896972	-3.7329635	1.2780903
F	-2.3647983	-1.4452901	2.7320085
H	-2.3202913	0.7693135	1.3441923
H	-2.3376758	0.8535591	-1.2024200

SCF energy GEOOPT = -1405.889684690 H
ZPE = 350.5 kJ/mol
FREEH energy = 403.09 kJ/mol
FREEH entropy = 0.66128 kJ/mol/K

$vibrational spectrum
\# mode symmetry wave number IR intensity selection rules

#		cm**(-1)	km/mol	IR	RAMAN
1		-0.00	0.00000	-	-
2		-0.00	0.00000	-	-
3		-0.00	0.00000	-	-
4		-0.00	0.00000	-	-
5		0.00	0.00000	-	-
6		0.00	0.00000	-	-
7	a	4.73	0.79198	YES	YES
8	a	19.92	0.01468	YES	YES
9	b	22.40	1.54034	YES	YES
10	b	36.66	0.00124	YES	YES
11	a	41.70	0.00018	YES	YES
12	a	42.93	0.11883	YES	YES
13	b	82.15	0.21282	YES	YES
14	a	108.64	0.00000	YES	YES
15	b	130.84	2.67840	YES	YES
16	b	145.10	0.00005	YES	YES
17	a	145.37	0.00239	YES	YES
18	a	218.20	0.00156	YES	YES
19	b	243.87	1.32622	YES	YES
20	b	270.15	0.12512	YES	YES
21	a	270.32	0.00001	YES	YES
22	a	276.18	1.03989	YES	YES
23	b	276.19	0.00105	YES	YES
24	a	315.33	0.00387	YES	YES
25	b	315.62	3.02935	YES	YES
26	a	324.74	0.00141	YES	YES
27	b	328.21	0.86727	YES	YES
28	b	355.60	0.00160	YES	YES
29	a	364.97	0.00508	YES	YES
30	a	442.54	0.00030	YES	YES
31	b	442.65	0.22297	YES	YES
32	a	469.92	0.03470	YES	YES
33	b	470.35	0.00029	YES	YES
34	a	542.99	0.16733	YES	YES
35	b	544.77	0.00041	YES	YES
36	b	592.02	0.00303	YES	YES
37	a	592.30	1.34372	YES	YES
38	b	619.07	12.19221	YES	YES
39	a	619.21	0.00050	YES	YES
40	a	674.36	0.02095	YES	YES
41	b	675.54	23.35997	YES	YES
42	a	695.77	0.51625	YES	YES
43	b	695.79	0.00161	YES	YES
44	b	735.89	0.02209	YES	YES
45	a	736.04	20.17110	YES	YES
46	b	817.32	96.58480	YES	YES
47	a	819.85	0.00025	YES	YES
48	b	885.65	0.03306	YES	YES
49	a	894.47	9.70934	YES	YES
50	b	983.91	0.21092	YES	YES
51	a	984.46	179.17307	YES	YES
52	b	1035.82	118.64773	YES	YES
53	a	1036.71	0.09992	YES	YES
54	b	1156.76	63.07178	YES	YES
55	a	1158.34	0.09476	YES	YES
56	b	1217.45	0.19019	YES	YES
57	a	1217.73	158.35333	YES	YES
58	b	1274.28	0.04791	YES	YES
59	a	1274.83	12.31884	YES	YES
60	b	1281.06	171.43964	YES	YES
61	a	1289.12	0.02326	YES	YES
62	b	1339.89	20.48711	YES	YES
63	a	1344.10	0.00156	YES	YES
64	b	1473.38	0.51201	YES	YES
65	a	1476.78	578.82256	YES	YES
66	b	1486.45	672.11814	YES	YES
67	a	1492.92	0.57913	YES	YES
68	b	1563.27	24.57921	YES	YES
69	a	1565.77	0.08815	YES	YES
70	b	1573.89	0.17571	YES	YES
71	a	1574.62	148.98892	YES	YES
72	a	3120.49	6.39135	YES	YES
73	b	3120.71	0.02955	YES	YES

```
    74        b            3128.80        35.73412       YES     YES
    75        a            3128.97         0.06541       YES     YES
$end
```

$[Ag(4FB)_3]^+$

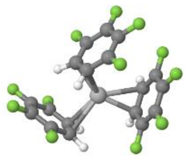

```
Method: (RI-)BP86(D3BJ)/def2-TZVPP
Symmetry: c1

Cartesian coordinates in Ångström:
Ag   -0.4186589   -0.5318373    0.0831094
F     1.3852531    2.6674457   -3.2640029
C     0.7390682    2.3859141   -2.1408860
C    -0.6632825    2.3794884   -2.1310773
F    -1.3330285    2.6530694   -3.2444004
C    -1.3435946    2.0887706   -0.9433389
F    -2.6786624    2.1275993   -0.9345491
C    -0.6383817    1.8009310    0.2393899
H    -1.1823504    1.8102102    1.1844939
C     0.7830048    1.8083135    0.2207833
H     1.3571713    1.6882518    1.1379902
C     1.4501077    2.0841794   -0.9696816
F     2.7862956    2.0819635   -1.0150918
F     1.3322443   -0.0052078    3.6806845
C     0.0720115   -0.2496421    3.2994420
C    -0.9282949    0.6623939    3.6608498
F    -0.6269566    1.7524480    4.3609788
C    -2.2544256    0.4354363    3.2641937
F    -3.2001334    1.3086610    3.5962268
C    -2.5677487   -0.7035921    2.5109478
F    -3.8374797   -0.9082836    2.1500473
C    -1.5732382   -1.6197771    2.1553009
H    -1.8670701   -2.5570224    1.6849191
C    -0.2348317   -1.3913103    2.5575027
H     0.5406908   -2.1371027    2.3864947
F     2.0955845   -3.1123100    0.1786967
C     1.6499650   -2.3756735   -0.8465881
C     0.2839254   -2.3940315   -1.1821475
H    -0.3562557   -3.1530373   -0.7329802
C    -0.1677297   -1.6276517   -2.2901647
H    -1.1971373   -1.6977519   -2.6377684
C     0.7507388   -0.8771816   -3.0237338
F     0.3463496   -0.1572020   -4.0756227
C     2.1105772   -0.8718298   -2.6869210
F     2.9765651   -0.1569690   -3.3930980
C     2.5617611   -1.6146576   -1.5844362
F     3.8479266   -1.5929950   -1.2555430

SCF energy GEOOPT =  -2035.407368123 H
ZPE =   525.3 kJ/mol
FREEH energy =   604.37 kJ/mol
FREEH entropy =   0.87696 kJ/mol/K

$vibrational spectrum
#  mode     symmetry     wave number   IR intensity    selection rules
#                         cm**(-1)        km/mol         IR     RAMAN
     1                      -0.00         0.00000        -       -
     2                      -0.00         0.00000        -       -
     3                      -0.00         0.00000        -       -
     4                      -0.00         0.00000        -       -
     5                       0.00         0.00000        -       -
     6                       0.00         0.00000        -       -
     7        a              7.45         0.52858       YES     YES
     8        a             11.92         0.09298       YES     YES
     9        a             20.20         0.38717       YES     YES
    10        a             27.18         0.08781       YES     YES
```

11	a	30.40	1.09653	YES	YES
12	a	32.37	0.47609	YES	YES
13	a	39.88	0.05426	YES	YES
14	a	40.90	0.01916	YES	YES
15	a	47.76	0.18579	YES	YES
16	a	53.46	0.35888	YES	YES
17	a	58.92	0.80560	YES	YES
18	a	67.64	0.59574	YES	YES
19	a	100.16	4.27835	YES	YES
20	a	109.00	1.56829	YES	YES
21	a	127.02	2.05335	YES	YES
22	a	147.14	0.00376	YES	YES
23	a	149.06	0.09260	YES	YES
24	a	150.18	0.00322	YES	YES
25	a	173.94	2.42504	YES	YES
26	a	202.12	0.27444	YES	YES
27	a	208.53	0.35276	YES	YES
28	a	269.15	0.02781	YES	YES
29	a	269.36	0.01313	YES	YES
30	a	269.79	0.02545	YES	YES
31	a	275.81	0.74634	YES	YES
32	a	275.88	0.29009	YES	YES
33	a	276.48	0.14240	YES	YES
34	a	301.76	0.28860	YES	YES
35	a	304.80	0.16784	YES	YES
36	a	309.85	0.13229	YES	YES
37	a	317.04	1.68763	YES	YES
38	a	317.21	1.70108	YES	YES
39	a	317.83	1.51494	YES	YES
40	a	361.02	0.14378	YES	YES
41	a	369.01	1.61429	YES	YES
42	a	373.38	0.85957	YES	YES
43	a	444.26	0.17915	YES	YES
44	a	444.56	0.05983	YES	YES
45	a	444.93	0.12052	YES	YES
46	a	471.48	0.17387	YES	YES
47	a	472.02	0.26308	YES	YES
48	a	472.31	0.03135	YES	YES
49	a	535.02	0.12865	YES	YES
50	a	542.77	0.09843	YES	YES
51	a	544.45	0.36158	YES	YES
52	a	592.93	0.71742	YES	YES
53	a	594.50	0.42446	YES	YES
54	a	594.88	0.77727	YES	YES
55	a	608.52	12.56012	YES	YES
56	a	611.31	5.29517	YES	YES
57	a	612.68	9.72570	YES	YES
58	a	675.21	8.91682	YES	YES
59	a	676.70	9.04139	YES	YES
60	a	676.78	10.50054	YES	YES
61	a	699.07	0.26763	YES	YES
62	a	704.46	1.08475	YES	YES
63	a	709.75	0.45394	YES	YES
64	a	736.21	6.40554	YES	YES
65	a	736.94	5.15645	YES	YES
66	a	737.95	14.70280	YES	YES
67	a	799.30	129.99432	YES	YES
68	a	803.00	66.22409	YES	YES
69	a	810.77	26.65712	YES	YES
70	a	885.40	11.70516	YES	YES
71	a	890.60	0.25075	YES	YES
72	a	894.13	9.52345	YES	YES
73	a	979.49	101.26202	YES	YES
74	a	980.36	36.62280	YES	YES
75	a	982.25	111.62273	YES	YES
76	a	1033.84	60.63078	YES	YES
77	a	1036.64	42.41699	YES	YES
78	a	1039.27	63.80140	YES	YES
79	a	1148.89	17.32060	YES	YES
80	a	1154.20	33.27508	YES	YES
81	a	1156.41	22.70913	YES	YES
82	a	1214.58	89.54759	YES	YES
83	a	1217.16	37.37733	YES	YES
84	a	1220.71	68.74667	YES	YES

```
    85        a           1265.48          9.92160       YES      YES
    86        a           1267.60          6.05063       YES      YES
    87        a           1269.61          9.91078       YES      YES
    88        a           1284.74         39.48375       YES      YES
    89        a           1289.27        119.89454       YES      YES
    90        a           1298.59         21.36674       YES      YES
    91        a           1336.55          0.43898       YES      YES
    92        a           1339.04          0.63737       YES      YES
    93        a           1342.85          0.31585       YES      YES
    94        a           1475.58        101.90302       YES      YES
    95        a           1476.37        148.90575       YES      YES
    96        a           1483.31         43.45047       YES      YES
    97        a           1485.54        354.73593       YES      YES
    98        a           1488.23        550.15210       YES      YES
    99        a           1495.79        282.45842       YES      YES
   100        a           1568.53          0.52608       YES      YES
   101        a           1571.36         53.18778       YES      YES
   102        a           1578.80         16.56928       YES      YES
   103        a           1580.11         13.39304       YES      YES
   104        a           1582.39         33.88443       YES      YES
   105        a           1582.89         42.12808       YES      YES
   106        a           3111.28          9.94720       YES      YES
   107        a           3121.50          1.89725       YES      YES
   108        a           3122.42          1.57745       YES      YES
   109        a           3131.64          8.65727       YES      YES
   110        a           3132.74          9.37447       YES      YES
   111        a           3135.47         12.06294       YES      YES
$end
```

{(4FB)Ag[*pf*]}*ip.*

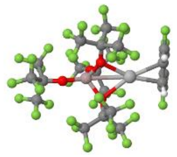

```
Method: (RI-)BP86(D3BJ)/def2-TZVPP
Symmetry: c1

Cartesian coordinates in Ångström:
Al   10.3018960   12.7157391    7.0901378
F    13.9092436    9.7048824    3.0921061
C    13.4937306    8.9632648    4.1252426
F    15.7086648    8.8979814    4.9655309
Ag   11.0666364   10.1665281    5.6498756
C    14.4293813    8.5356404    5.0657056
F    14.9244748    7.3124062    7.0247562
C    14.0250061    7.7119308    6.1263307
F    12.3307937    6.5258535    7.2526961
C    12.6882219    7.3284361    6.2408875
C    11.7395225    7.7606802    5.3125137
H    10.7333843    7.3497785    5.3568875
C    12.1423144    8.5986240    4.2391671
H    11.4763326    8.7908249    3.3984026
O     9.4114049   14.0595349    7.6604326
C     9.2057038   15.1885013    8.3935577
C     9.9349291   15.1203070    9.7988037
F    11.2326465   15.4754394    9.6862530
F     9.3642622   15.9372756   10.7094354
F     9.9023811   13.8632942   10.2815036
C     7.6491246   15.3507072    8.6224549
F     6.9785739   15.0499433    7.4957316
F     7.2221106   14.5089214    9.5914305
F     7.3128351   16.6068761    8.9879918
C     9.7506446   16.4545034    7.6198585
F     8.9397154   16.7799254    6.5907849
F     9.8511919   17.5363095    8.4225338
F    10.9729315   16.1932532    7.1134840
O    11.8978477   12.5548184    7.7553319
C    13.2508908   12.5359630    7.7160522
C    13.7809104   12.8673331    6.2676435
```

```
F    13.5389375   11.8070137    5.4342848
F    15.1005439   13.1176773    6.2227397
F    13.1298825   13.9252363    5.7625010
C    13.8372822   13.5954127    8.7346001
F    13.1957657   13.5136004    9.9100404
F    13.6842499   14.8460716    8.2548130
F    15.1574366   13.3920877    8.9598462
C    13.7651587   11.1025305    8.1491814
F    13.6808500   10.9233427    9.4752316
F    15.0333174   10.8550669    7.7648992
F    12.9810805   10.1371987    7.5765225
O    10.4797174   12.5696349    5.3083882
C     9.8796686   12.8993397    4.1155392
C     9.4833475   14.4275712    4.0685552
F     8.3660348   14.6477702    4.7874987
F     9.2540544   14.8471470    2.8047725
F    10.4744846   15.1703800    4.5856279
C    10.9107594   12.6204916    2.9510079
F    11.5078659   11.4097695    3.1294425
F    11.8867546   13.5422650    2.9412333
F    10.3273288   12.5970500    1.7380697
C     8.5840608   12.0264686    3.9014449
F     8.9325622   10.7197572    3.6880336
F     7.8424043   12.4240854    2.8552660
F     7.8175906   12.0524056    5.0035240
O     9.5352139   11.0918242    7.2862278
C     8.7893739   10.2861308    8.1147841
C     9.7470305    9.5607427    9.1353151
F    10.4832942    8.6202592    8.4812008
F     9.0871801    8.9473873   10.1341958
F    10.6063983   10.4431235    9.6701278
C     7.7005967   11.1134630    8.9031865
F     7.1234776   12.0066604    8.0821334
F     8.2647381   11.7786501    9.9273846
F     6.7348360   10.3145448    9.4080199
C     8.0264526    9.2074417    7.2416686
F     6.9680215    9.7459270    6.6151535
F     7.6071299    8.1602093    7.9723757
F     8.8438152    8.7087819    6.2655457

SCF energy GEOOPT =  -5526.614800451 H
ZPE =   736.1 kJ/mol
FREEH energy =   914.68 kJ/mol
FREEH entropy =   1.58014 kJ/mol/K

$vibrational spectrum
#  mode     symmetry     wave number   IR intensity    selection rules
#                         cm**(-1)        km/mol         IR     RAMAN
     1                       -0.00         0.00000        -       -
     2                       -0.00         0.00000        -       -
     3                       -0.00         0.00000        -       -
     4                        0.00         0.00000        -       -
     5                        0.00         0.00000        -       -
     6                        0.00         0.00000        -       -
     7        a              12.00         0.14847       YES     YES
     8        a              18.46         0.09359       YES     YES
     9        a              19.20         0.43581       YES     YES
    10        a              22.31         0.08850       YES     YES
    11        a              28.70         0.03025       YES     YES
    12        a              29.39         0.09559       YES     YES
    13        a              31.14         0.34610       YES     YES
    14        a              32.88         0.24439       YES     YES
    15        a              37.17         0.08639       YES     YES
    16        a              39.39         0.30220       YES     YES
    17        a              40.95         0.22356       YES     YES
    18        a              44.71         0.12805       YES     YES
    19        a              46.40         0.05277       YES     YES
    20        a              49.54         1.02346       YES     YES
    21        a              56.07         0.48026       YES     YES
    22        a              58.52         0.13354       YES     YES
    23        a              62.82         0.32535       YES     YES
    24        a              63.95         0.55266       YES     YES
    25        a              65.31         0.16228       YES     YES
    26        a              67.99         0.97208       YES     YES
```

27	a	69.87	0.41556	YES	YES
28	a	73.48	2.91496	YES	YES
29	a	74.86	0.07525	YES	YES
30	a	77.76	0.20852	YES	YES
31	a	79.92	0.56890	YES	YES
32	a	81.10	1.36774	YES	YES
33	a	82.97	0.20565	YES	YES
34	a	84.88	1.45277	YES	YES
35	a	86.47	0.13300	YES	YES
36	a	89.84	0.16373	YES	YES
37	a	91.15	1.19209	YES	YES
38	a	95.46	0.33405	YES	YES
39	a	97.38	0.76148	YES	YES
40	a	98.79	1.16083	YES	YES
41	a	102.41	1.23041	YES	YES
42	a	106.68	0.24383	YES	YES
43	a	108.54	0.23473	YES	YES
44	a	119.53	0.17695	YES	YES
45	a	126.34	5.80541	YES	YES
46	a	149.82	0.18337	YES	YES
47	a	154.88	0.10068	YES	YES
48	a	158.78	0.16793	YES	YES
49	a	164.94	0.07789	YES	YES
50	a	165.89	0.02970	YES	YES
51	a	169.18	1.06757	YES	YES
52	a	170.14	0.29849	YES	YES
53	a	171.33	0.32896	YES	YES
54	a	174.63	0.51608	YES	YES
55	a	193.50	3.49018	YES	YES
56	a	195.13	4.35517	YES	YES
57	a	196.24	3.15062	YES	YES
58	a	201.87	2.13734	YES	YES
59	a	222.90	0.09013	YES	YES
60	a	259.60	1.53380	YES	YES
61	a	262.12	1.32564	YES	YES
62	a	269.05	0.97648	YES	YES
63	a	270.68	0.04642	YES	YES
64	a	272.35	3.60507	YES	YES
65	a	274.34	1.90542	YES	YES
66	a	276.58	0.72292	YES	YES
67	a	276.83	1.26960	YES	YES
68	a	279.77	0.01118	YES	YES
69	a	280.07	0.23255	YES	YES
70	a	280.33	0.34296	YES	YES
71	a	280.69	0.30482	YES	YES
72	a	282.11	1.94381	YES	YES
73	a	282.85	1.96868	YES	YES
74	a	300.02	4.55528	YES	YES
75	a	300.31	5.32923	YES	YES
76	a	302.64	7.62139	YES	YES
77	a	306.29	1.56680	YES	YES
78	a	306.79	0.12068	YES	YES
79	a	309.11	0.42267	YES	YES
80	a	313.63	0.77256	YES	YES
81	a	314.85	0.59975	YES	YES
82	a	316.17	0.90467	YES	YES
83	a	316.54	2.09266	YES	YES
84	a	317.24	0.03068	YES	YES
85	a	318.62	1.38917	YES	YES
86	a	319.09	1.26910	YES	YES
87	a	320.48	0.23511	YES	YES
88	a	337.83	0.30230	YES	YES
89	a	339.06	1.83280	YES	YES
90	a	343.98	3.52446	YES	YES
91	a	347.72	8.64259	YES	YES
92	a	354.94	9.95589	YES	YES
93	a	357.62	8.57087	YES	YES
94	a	359.71	0.58536	YES	YES
95	a	377.90	40.23853	YES	YES
96	a	381.49	5.42414	YES	YES
97	a	416.53	23.33701	YES	YES
98	a	424.56	48.71610	YES	YES
99	a	445.00	0.27039	YES	YES
100	a	451.95	81.87505	YES	YES

101	a	473.01	0.05005	YES	YES
102	a	504.35	0.74722	YES	YES
103	a	507.08	2.07433	YES	YES
104	a	509.71	0.78734	YES	YES
105	a	510.41	2.14636	YES	YES
106	a	511.45	3.47256	YES	YES
107	a	511.51	1.75133	YES	YES
108	a	513.22	1.43058	YES	YES
109	a	513.36	2.88135	YES	YES
110	a	513.96	4.59316	YES	YES
111	a	516.49	7.50539	YES	YES
112	a	517.69	2.95217	YES	YES
113	a	519.16	2.38255	YES	YES
114	a	520.78	0.37467	YES	YES
115	a	522.42	1.83846	YES	YES
116	a	532.36	19.17078	YES	YES
117	a	540.33	0.27256	YES	YES
118	a	543.05	1.82815	YES	YES
119	a	544.19	9.02957	YES	YES
120	a	545.08	0.39441	YES	YES
121	a	545.48	0.96128	YES	YES
122	a	545.83	0.65979	YES	YES
123	a	546.08	1.04999	YES	YES
124	a	548.18	1.36442	YES	YES
125	a	548.77	0.56152	YES	YES
126	a	553.44	27.83496	YES	YES
127	a	591.13	1.22528	YES	YES
128	a	612.59	9.64711	YES	YES
129	a	675.79	15.76624	YES	YES
130	a	694.46	0.38698	YES	YES
131	a	695.38	1.94193	YES	YES
132	a	696.67	9.36106	YES	YES
133	a	697.24	21.87584	YES	YES
134	a	697.99	32.48256	YES	YES
135	a	698.19	49.65606	YES	YES
136	a	699.54	32.03926	YES	YES
137	a	701.07	23.31512	YES	YES
138	a	701.27	40.82637	YES	YES
139	a	711.51	38.56601	YES	YES
140	a	713.48	0.75308	YES	YES
141	a	722.32	2.33028	YES	YES
142	a	728.31	3.46692	YES	YES
143	a	735.01	11.08258	YES	YES
144	a	762.93	14.12196	YES	YES
145	a	764.39	4.23080	YES	YES
146	a	801.08	86.50220	YES	YES
147	a	805.16	7.27229	YES	YES
148	a	850.03	15.82093	YES	YES
149	a	890.55	12.39073	YES	YES
150	a	917.76	92.13556	YES	YES
151	a	926.51	3.41038	YES	YES
152	a	929.03	5.77726	YES	YES
153	a	934.12	43.32544	YES	YES
154	a	934.93	191.08630	YES	YES
155	a	936.13	259.69466	YES	YES
156	a	937.51	28.73424	YES	YES
157	a	941.72	358.23258	YES	YES
158	a	979.12	107.38629	YES	YES
159	a	1034.57	50.81038	YES	YES
160	a	1037.04	2.49895	YES	YES
161	a	1044.66	5.87478	YES	YES
162	a	1050.61	5.59780	YES	YES
163	a	1062.81	0.70507	YES	YES
164	a	1080.25	29.55419	YES	YES
165	a	1091.31	3.66072	YES	YES
166	a	1098.68	84.44765	YES	YES
167	a	1101.87	7.88778	YES	YES
168	a	1104.56	7.16882	YES	YES
169	a	1113.10	11.83898	YES	YES
170	a	1120.98	17.65907	YES	YES
171	a	1124.99	19.71317	YES	YES
172	a	1134.68	19.45716	YES	YES
173	a	1137.50	25.05564	YES	YES
174	a	1138.39	15.34329	YES	YES

175	a	1142.27	101.91296	YES	YES
176	a	1143.98	98.49754	YES	YES
177	a	1151.09	128.23662	YES	YES
178	a	1151.31	116.57034	YES	YES
179	a	1153.89	54.58074	YES	YES
180	a	1156.26	28.01770	YES	YES
181	a	1159.26	34.52213	YES	YES
182	a	1163.97	83.29461	YES	YES
183	a	1168.36	263.02047	YES	YES
184	a	1172.23	207.95840	YES	YES
185	a	1176.90	206.69310	YES	YES
186	a	1181.24	161.36447	YES	YES
187	a	1191.49	809.65276	YES	YES
188	a	1194.08	1198.93684	YES	YES
189	a	1196.03	1313.24315	YES	YES
190	a	1200.19	411.17460	YES	YES
191	a	1201.74	289.01584	YES	YES
192	a	1204.71	231.22010	YES	YES
193	a	1211.43	536.03308	YES	YES
194	a	1216.15	13.81511	YES	YES
195	a	1221.01	241.96573	YES	YES
196	a	1222.41	426.50287	YES	YES
197	a	1222.90	629.25226	YES	YES
198	a	1258.08	12.21099	YES	YES
199	a	1260.04	0.82006	YES	YES
200	a	1263.27	16.08824	YES	YES
201	a	1290.73	43.64065	YES	YES
202	a	1301.37	266.30257	YES	YES
203	a	1313.34	108.48409	YES	YES
204	a	1343.54	3.56194	YES	YES
205	a	1481.40	250.18289	YES	YES
206	a	1489.21	241.26175	YES	YES
207	a	1577.23	12.54931	YES	YES
208	a	1592.35	50.15987	YES	YES
209	a	3124.99	2.90983	YES	YES
210	a	3146.53	7.34270	YES	YES

$end

$[Ag(5FB)_1]^+$

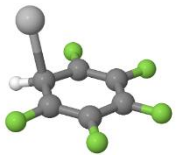

Method: (RI-)BP86(D3BJ)/def2-TZVPP
Symmetry: c1

Cartesian coordinates in Ångström:

Ag	1.8402194	-1.9818682	0.7360223
C	-0.3660772	-1.0324754	-0.7931239
F	-0.6999415	-2.1242714	-1.4727636
F	0.3699305	2.4382697	1.3520181
C	-0.2171647	-1.1169843	0.6279464
H	-0.7303760	-1.9337025	1.1470131
F	0.1794751	2.4901973	-1.3479718
C	-0.0025870	0.1008631	1.3487682
F	0.0047391	0.0713956	2.6770453
C	0.1435638	1.3152473	0.6868382
C	0.0323860	1.3446596	-0.7169595
C	-0.2217396	0.1775729	-1.4634008
F	-0.3324279	0.2510963	-2.7814320

SCF energy GEOOPT = -875.6217572200 H
ZPE = 154.1 kJ/mol
FREEH energy = 182.97 kJ/mol
FREEH entropy = 0.44883 kJ/mol/K

$vibrational spectrum

# mode	symmetry	wave number	IR intensity	selection rules	
#		cm**(-1)	km/mol	IR	RAMAN

1		-0.00	0.00000	-	-
2		-0.00	0.00000	-	-
3		-0.00	0.00000	-	-
4		0.00	0.00000	-	-
5		0.00	0.00000	-	-
6		0.00	0.00000	-	-
7	a	47.31	0.05528	YES	YES
8	a	60.27	0.08360	YES	YES
9	a	122.86	1.55976	YES	YES
10	a	125.86	0.00070	YES	YES
11	a	157.87	0.86769	YES	YES
12	a	236.43	1.58500	YES	YES
13	a	263.90	0.17580	YES	YES
14	a	270.40	0.16223	YES	YES
15	a	299.88	1.46093	YES	YES
16	a	317.58	2.30687	YES	YES
17	a	365.24	0.38507	YES	YES
18	a	404.08	3.31873	YES	YES
19	a	424.06	0.70068	YES	YES
20	a	455.07	2.56748	YES	YES
21	a	566.02	0.94815	YES	YES
22	a	595.25	0.38151	YES	YES
23	a	638.06	0.42827	YES	YES
24	a	680.39	0.82440	YES	YES
25	a	698.72	3.78785	YES	YES
26	a	709.11	2.97223	YES	YES
27	a	847.37	99.65375	YES	YES
28	a	946.45	107.53715	YES	YES
29	a	1081.66	126.26872	YES	YES
30	a	1142.62	32.10431	YES	YES
31	a	1163.75	49.44725	YES	YES
32	a	1270.48	30.77694	YES	YES
33	a	1344.26	3.92231	YES	YES
34	a	1347.61	43.28272	YES	YES
35	a	1474.63	440.82663	YES	YES
36	a	1482.00	387.45315	YES	YES
37	a	1556.61	82.22862	YES	YES
38	a	1597.24	65.23912	YES	YES
39	a	3071.39	31.30434	YES	YES

$end

$[Ag(5FB)_2]^+$

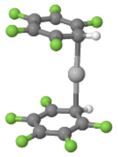

Method: (RI-)BP86(D3BJ)/def2-TZVPP
Symmetry: c1

Cartesian coordinates in Ångström:

C	2.4891506	1.0946765	0.0417090
C	2.5696129	1.0720279	-1.3508484
C	2.7458971	-0.1583500	-2.0059159
C	2.8378749	-1.3618364	-1.2864705
C	2.7552561	-1.3285189	0.1058337
C	2.5389501	-0.1067888	0.8031185
F	2.4130995	2.2657753	0.6729498
F	2.4888331	2.1931804	-2.0583530
F	2.8080763	-0.1860928	-3.3254500
F	3.0088524	-2.5076834	-1.9339434
F	2.9280412	-2.4522834	0.7978692
C	-2.1155070	0.4756888	2.0953827
C	-2.4673257	1.7638023	1.6910884
C	-2.6863127	2.0162308	0.3262530
C	-2.5548925	0.9994056	-0.6344097
C	-2.2036690	-0.2854884	-0.2197288
C	-1.9353486	-0.5714669	1.1484842
F	-1.9914868	0.2091573	3.3935340
F	-2.6042940	2.7441987	2.5751029

```
F    -3.0043926    3.2379599   -0.0636520
F    -2.7715399    1.2682616   -1.9169431
F    -2.1661328   -1.2732544   -1.1134188
Ag    0.2982302   -0.3219132    0.9527168
H     2.7944706   -0.0511707    1.8641681
H    -1.9802434   -1.6080180    1.4916234

SCF energy GEOOPT =  -1604.437312542 H
ZPE =   308.4 kJ/mol
FREEH energy =   365.94 kJ/mol
FREEH entropy =   0.70313 kJ/mol/K

$vibrational spectrum
#  mode     symmetry     wave number   IR intensity    selection rules
#                         cm**(-1)        km/mol          IR     RAMAN
     1                      -0.00         0.00000         -       -
     2                      -0.00         0.00000         -       -
     3                      -0.00         0.00000         -       -
     4                      -0.00         0.00000         -       -
     5                       0.00         0.00000         -       -
     6                       0.00         0.00000         -       -
     7        a              4.40         0.08279        YES     YES
     8        a             14.57         0.03632        YES     YES
     9        a             18.51         0.14549        YES     YES
    10        a             40.93         0.01323        YES     YES
    11        a             41.18         0.00524        YES     YES
    12        a             66.84         0.00531        YES     YES
    13        a             69.40         0.08854        YES     YES
    14        a            103.36         0.02460        YES     YES
    15        a            127.88         0.00630        YES     YES
    16        a            128.49         0.01816        YES     YES
    17        a            135.38         1.29453        YES     YES
    18        a            156.35         0.02096        YES     YES
    19        a            167.89         2.03865        YES     YES
    20        a            233.05         0.34151        YES     YES
    21        a            244.63         0.77946        YES     YES
    22        a            263.91         0.17127        YES     YES
    23        a            263.99         0.16011        YES     YES
    24        a            269.49         0.10867        YES     YES
    25        a            269.58         0.10642        YES     YES
    26        a            298.44         1.29464        YES     YES
    27        a            298.51         1.04619        YES     YES
    28        a            318.57         2.56265        YES     YES
    29        a            318.66         2.12614        YES     YES
    30        a            366.36         0.12142        YES     YES
    31        a            366.77         0.30027        YES     YES
    32        a            387.70        10.53258        YES     YES
    33        a            392.31         1.24502        YES     YES
    34        a            425.54         0.55140        YES     YES
    35        a            425.63         0.24698        YES     YES
    36        a            456.73         1.09968        YES     YES
    37        a            456.90         2.59152        YES     YES
    38        a            565.85         1.75505        YES     YES
    39        a            566.27         0.02234        YES     YES
    40        a            585.61         0.08875        YES     YES
    41        a            586.83         0.23228        YES     YES
    42        a            640.84         0.40855        YES     YES
    43        a            640.97         0.78388        YES     YES
    44        a            676.61         1.09640        YES     YES
    45        a            676.74         1.40741        YES     YES
    46        a            696.29         2.45955        YES     YES
    47        a            696.31         2.12454        YES     YES
    48        a            707.82         5.64792        YES     YES
    49        a            708.14         9.72325        YES     YES
    50        a            826.26       189.84551        YES     YES
    51        a            834.89        24.93929        YES     YES
    52        a            944.12        74.29258        YES     YES
    53        a            944.65       135.01897        YES     YES
    54        a           1076.55       105.82229        YES     YES
    55        a           1076.69       152.03842        YES     YES
    56        a           1139.20        25.34099        YES     YES
    57        a           1139.41        18.65115        YES     YES
    58        a           1163.29        71.53706        YES     YES
    59        a           1164.46        36.62482        YES     YES
```

60	a	1270.04	52.06605	YES	YES
61	a	1274.04	5.01947	YES	YES
62	a	1333.37	7.43395	YES	YES
63	a	1333.53	6.05057	YES	YES
64	a	1350.07	98.61084	YES	YES
65	a	1358.29	23.79908	YES	YES
66	a	1481.18	52.94488	YES	YES
67	a	1482.66	240.72525	YES	YES
68	a	1484.51	777.83849	YES	YES
69	a	1487.67	355.50664	YES	YES
70	a	1565.98	33.47368	YES	YES
71	a	1566.59	16.11800	YES	YES
72	a	1590.71	44.23436	YES	YES
73	a	1590.93	71.29434	YES	YES
74	a	3098.22	23.19600	YES	YES
75	a	3098.27	28.51285	YES	YES

$end

$[Ag(5FB)_3]^+$

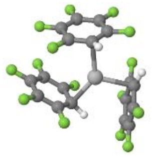

Method: (RI-)BP86(D3BJ)/def2-TZVPP
Symmetry: c1

Cartesian coordinates in Ångström:

C	1.4819886	2.0671898	-1.3442811
C	1.0829397	3.0800010	-0.4706520
C	-0.2606334	3.4833400	-0.4539071
C	-1.2018628	2.8931013	-1.3125014
C	-0.7835760	1.8912797	-2.1896941
C	0.5533394	1.4296047	-2.1985586
F	2.7740884	1.7301612	-1.4057375
F	1.9613428	3.6647784	0.3414996
F	-0.6483218	4.4259401	0.3910248
F	-2.4672651	3.2965796	-1.2883586
F	-1.6591065	1.3805655	-3.0630586
C	-1.3607739	-2.8135744	-1.3269229
C	-1.4794221	-3.5938859	-0.1745079
C	-2.1952353	-3.0893884	0.9223674
C	-2.7887028	-1.8180687	0.8752241
C	-2.6588566	-1.0527669	-0.2848038
C	-1.9256093	-1.5233419	-1.3954124
F	-0.7276498	-3.3134279	-2.3911102
F	-0.9255386	-4.8017193	-0.1060921
F	-2.3005661	-3.8190072	2.0259707
F	-3.4653435	-1.3619217	1.9245339
F	-3.2681782	0.1359331	-0.3541904
Ag	0.0888731	-0.3451243	-0.7049479
C	2.2742668	-1.2881207	0.8583112
C	3.2298653	-0.2982058	1.0954447
C	2.8661523	0.8416610	1.8282297
C	1.5576374	1.0022008	2.3121625
C	0.6149395	0.0029840	2.0701921
C	0.9440974	-1.1496778	1.3201536
F	2.6354007	-2.4083393	0.2279461
F	4.4718372	-0.4264386	0.6419150
F	3.7649632	1.7872715	2.0514571
F	1.2320984	2.0946330	2.9985103
F	-0.6101773	0.1183441	2.5944772
H	0.9151253	0.8346607	-3.0389370
H	-2.0271557	-1.0248518	-2.3600840
H	0.3050193	-2.0323686	1.3843377

SCF energy GEOOPT = -2333.232604967 H
ZPE = 462.0 kJ/mol
FREEH energy = 548.58 kJ/mol
FREEH entropy = 0.92036 kJ/mol/K

$vibrational spectrum

# mode	symmetry	wave number cm**(-1)	IR intensity km/mol	selection rules IR	selection rules RAMAN
1		-0.00	0.00000	-	-
2		-0.00	0.00000	-	-
3		-0.00	0.00000	-	-
4		0.00	0.00000	-	-
5		0.00	0.00000	-	-
6		0.00	0.00000	-	-
7	a	9.38	0.09014	YES	YES
8	a	15.65	0.05018	YES	YES
9	a	17.38	0.04867	YES	YES
10	a	26.88	0.07391	YES	YES
11	a	33.17	0.67975	YES	YES
12	a	38.09	0.01560	YES	YES
13	a	39.27	0.06411	YES	YES
14	a	47.85	0.16472	YES	YES
15	a	48.66	0.01776	YES	YES
16	a	49.90	0.39008	YES	YES
17	a	60.58	0.06415	YES	YES
18	a	67.00	0.02375	YES	YES
19	a	99.73	0.81476	YES	YES
20	a	111.19	5.22118	YES	YES
21	a	127.49	2.52221	YES	YES
22	a	128.15	0.08033	YES	YES
23	a	128.90	0.18278	YES	YES
24	a	137.93	0.47377	YES	YES
25	a	155.78	0.56709	YES	YES
26	a	159.17	0.22196	YES	YES
27	a	163.52	0.86244	YES	YES
28	a	213.85	1.34025	YES	YES
29	a	225.51	0.96502	YES	YES
30	a	228.69	0.12606	YES	YES
31	a	263.63	0.12598	YES	YES
32	a	264.17	0.11492	YES	YES
33	a	264.42	0.17224	YES	YES
34	a	268.78	0.07918	YES	YES
35	a	269.13	0.03104	YES	YES
36	a	269.33	0.09346	YES	YES
37	a	297.65	0.92970	YES	YES
38	a	297.91	0.66212	YES	YES
39	a	298.28	1.03400	YES	YES
40	a	318.74	2.30419	YES	YES
41	a	319.43	1.83693	YES	YES
42	a	319.56	0.99741	YES	YES
43	a	340.02	8.14947	YES	YES
44	a	350.87	3.39992	YES	YES
45	a	366.50	1.07054	YES	YES
46	a	368.61	0.86658	YES	YES
47	a	371.81	0.05974	YES	YES
48	a	376.88	0.05733	YES	YES
49	a	425.24	0.35214	YES	YES
50	a	425.98	0.68979	YES	YES
51	a	426.31	0.17108	YES	YES
52	a	458.89	2.03255	YES	YES
53	a	459.03	0.77274	YES	YES
54	a	459.33	1.27974	YES	YES
55	a	565.84	2.75305	YES	YES
56	a	566.35	0.47478	YES	YES
57	a	566.70	1.04113	YES	YES
58	a	569.43	1.11367	YES	YES
59	a	575.03	0.62563	YES	YES
60	a	576.34	2.59793	YES	YES
61	a	640.90	0.54666	YES	YES
62	a	642.04	0.23509	YES	YES
63	a	642.89	0.30406	YES	YES
64	a	674.64	1.33484	YES	YES
65	a	676.55	1.40278	YES	YES
66	a	676.94	0.38300	YES	YES
67	a	692.35	1.38980	YES	YES
68	a	694.15	1.15499	YES	YES
69	a	700.25	2.55951	YES	YES
70	a	707.68	5.30338	YES	YES

71	a	709.02	8.56433	YES	YES
72	a	709.70	6.64239	YES	YES
73	a	814.92	85.86013	YES	YES
74	a	818.70	160.22275	YES	YES
75	a	830.74	39.57275	YES	YES
76	a	940.85	52.18536	YES	YES
77	a	941.86	40.72457	YES	YES
78	a	945.64	198.75471	YES	YES
79	a	1069.30	145.18382	YES	YES
80	a	1071.53	137.59619	YES	YES
81	a	1072.39	77.99314	YES	YES
82	a	1130.39	19.27444	YES	YES
83	a	1132.25	5.29934	YES	YES
84	a	1135.58	19.33687	YES	YES
85	a	1160.87	108.82625	YES	YES
86	a	1165.48	36.00304	YES	YES
87	a	1168.34	16.35459	YES	YES
88	a	1272.22	33.11234	YES	YES
89	a	1273.25	7.62930	YES	YES
90	a	1274.29	10.91051	YES	YES
91	a	1328.39	3.51311	YES	YES
92	a	1328.77	0.95408	YES	YES
93	a	1332.11	6.11099	YES	YES
94	a	1353.81	66.41287	YES	YES
95	a	1354.48	34.65666	YES	YES
96	a	1366.19	34.92329	YES	YES
97	a	1479.76	35.88530	YES	YES
98	a	1481.51	331.13775	YES	YES
99	a	1486.59	149.44857	YES	YES
100	a	1487.05	112.62215	YES	YES
101	a	1490.48	437.67796	YES	YES
102	a	1495.71	777.35998	YES	YES
103	a	1574.20	17.06650	YES	YES
104	a	1575.62	3.32095	YES	YES
105	a	1578.28	3.13044	YES	YES
106	a	1593.34	58.38248	YES	YES
107	a	1594.14	42.77108	YES	YES
108	a	1594.65	32.84544	YES	YES
109	a	3101.29	21.54516	YES	YES
110	a	3112.88	19.71437	YES	YES
111	a	3119.65	18.90812	YES	YES

$end

{(5FB)Ag[*pf*]}$_{ip.}$

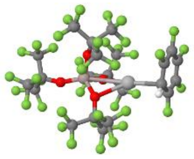

Method: (RI-)BP86(D3BJ)/def2-TZVPP
Symmetry: c1

Cartesian coordinates in Ångström:

Ag	0.8083290	9.1092529	6.1893926
Al	1.1947435	7.3469344	3.7605238
F	1.3634249	8.9560887	10.6637076
F	3.7486812	10.1399357	10.1047163
C	0.4767651	10.8960801	7.6801066
H	-0.4251677	11.2766060	7.2011807
C	0.3679901	10.0453771	8.8026153
F	-0.8194580	9.5183346	9.1213415
F	3.9916450	11.8785842	8.0252181
C	1.4665171	9.7842974	9.6222923
C	2.6905312	10.4031481	9.3418194
C	2.8193385	11.2875969	8.2633113
C	1.7101081	11.5463438	7.4588198
F	1.8074057	12.4642215	6.4944327
O	1.0732081	6.0353028	2.6703738
C	1.3699110	5.3349262	1.5409935
C	0.4864105	5.8411936	0.3324799

```
F    -0.7902043    5.4198985    0.4552660
F     0.9521466    5.4024365   -0.8570687
F     0.4733371    7.1895961    0.3061763
C     2.8935832    5.4883639    1.1345515
F     3.6668559    5.5464758    2.2358446
F     3.0944885    6.6281773    0.4395220
F     3.3235365    4.4616090    0.3706887
C     1.0558169    3.8091929    1.8126785
F     2.0389287    3.2482300    2.5538233
F     0.9413427    3.0975029    0.6703790
F    -0.0945255    3.6854666    2.4991269
O    -0.3299200    8.2124824    4.1391145
C    -1.6925511    8.0646712    4.2279827
C    -2.3329583    9.4971116    4.4206630
F    -2.2895337   10.2025352    3.2798491
F    -3.6070624    9.4429315    4.8473469
F    -1.6321966   10.2097922    5.3527423
C    -2.0606805    7.1469422    5.4565850
F    -1.3144349    6.0298888    5.4412611
F    -1.7808905    7.8003136    6.6238691
F    -3.3563288    6.7952475    5.4872055
C    -2.2936154    7.4284597    2.9127377
F    -2.0706708    6.1006080    2.8883469
F    -3.6273038    7.6286275    2.8235117
F    -1.7126178    7.9761559    1.8339548
O     2.3594400    8.5601145    3.3287830
C     2.7541830    9.8019068    2.9589702
C     3.5745408    9.7273823    1.6075064
F     2.7485810    9.5056754    0.5650194
F     4.2493692   10.8772541    1.3687722
F     4.4645729    8.7245778    1.6596551
C     3.7027885   10.4035572    4.0749993
F     3.1927858   10.1253664    5.3153678
F     4.9252433    9.8513442    4.0350816
F     3.8357225   11.7399242    3.9932023
C     1.5126474   10.7533324    2.7557879
F     0.9601302   11.0599482    3.9705989
F     1.8194860   11.9141832    2.1513494
F     0.5650438   10.1368748    2.0330313
O     1.5601410    6.9313994    5.4818507
C     2.2964798    6.1401393    6.3323241
C     3.6863242    6.8307109    6.6069123
F     3.4950207    7.9455377    7.3765164
F     4.5585588    6.0358366    7.2490161
F     4.2421575    7.2333156    5.4551823
C     2.5251341    4.6994114    5.7328202
F     1.3850327    4.2482754    5.1850562
F     3.4732567    4.7307016    4.7782634
F     2.9183771    3.8217546    6.6817929
C     1.5091286    5.9724865    7.6966795
F     0.4983680    5.0983235    7.5744983
F     2.3062232    5.5710703    8.7022252
F     0.9554480    7.1611248    8.0772535

SCF energy GEOOPT =  -5625.889532171 H
ZPE =   715.0 kJ/mol
FREEH energy =   896.07 kJ/mol
FREEH entropy =   1.59862 kJ/mol/K

$vibrational spectrum
#  mode     symmetry     wave number   IR intensity    selection rules
#                         cm**(-1)        km/mol         IR     RAMAN
     1                       -0.00         0.00000        -       -
     2                       -0.00         0.00000        -       -
     3                       -0.00         0.00000        -       -
     4                       -0.00         0.00000        -       -
     5                       -0.00         0.00000        -       -
     6                        0.00         0.00000        -       -
     7        a               8.04         0.05029       YES     YES
     8        a              17.88         0.06718       YES     YES
     9        a              20.57         0.06919       YES     YES
    10        a              22.00         0.09406       YES     YES
    11        a              27.58         0.09124       YES     YES
    12        a              28.11         0.18857       YES     YES
```

13	a	31.44	0.35859	YES	YES
14	a	34.10	0.42302	YES	YES
15	a	35.89	0.01904	YES	YES
16	a	38.27	0.32965	YES	YES
17	a	42.00	0.11728	YES	YES
18	a	46.53	0.48214	YES	YES
19	a	46.96	0.20676	YES	YES
20	a	47.40	0.22128	YES	YES
21	a	56.67	0.80671	YES	YES
22	a	58.06	0.20078	YES	YES
23	a	63.39	0.20300	YES	YES
24	a	65.56	0.13251	YES	YES
25	a	66.70	0.70542	YES	YES
26	a	68.14	0.16851	YES	YES
27	a	69.91	0.68097	YES	YES
28	a	73.33	1.04856	YES	YES
29	a	74.09	1.50754	YES	YES
30	a	78.31	0.75937	YES	YES
31	a	79.80	1.46181	YES	YES
32	a	81.28	0.73215	YES	YES
33	a	82.61	0.10994	YES	YES
34	a	83.57	1.12625	YES	YES
35	a	87.05	0.13369	YES	YES
36	a	90.92	0.39891	YES	YES
37	a	92.12	0.75263	YES	YES
38	a	95.81	0.20985	YES	YES
39	a	97.98	0.48011	YES	YES
40	a	98.27	0.53364	YES	YES
41	a	102.15	0.97230	YES	YES
42	a	107.23	0.35129	YES	YES
43	a	108.34	0.27185	YES	YES
44	a	119.59	0.20614	YES	YES
45	a	131.33	5.87018	YES	YES
46	a	136.41	0.21961	YES	YES
47	a	154.45	0.09345	YES	YES
48	a	158.88	0.23649	YES	YES
49	a	162.00	0.40157	YES	YES
50	a	165.20	0.04995	YES	YES
51	a	168.51	0.27486	YES	YES
52	a	169.36	0.96434	YES	YES
53	a	171.23	0.84190	YES	YES
54	a	171.60	0.06636	YES	YES
55	a	174.73	0.32072	YES	YES
56	a	194.70	4.79656	YES	YES
57	a	195.71	3.45540	YES	YES
58	a	200.96	3.71679	YES	YES
59	a	222.25	0.43959	YES	YES
60	a	226.63	1.18711	YES	YES
61	a	259.39	1.64113	YES	YES
62	a	261.71	1.19192	YES	YES
63	a	264.52	0.09742	YES	YES
64	a	269.30	0.17764	YES	YES
65	a	269.40	0.80207	YES	YES
66	a	272.28	3.86690	YES	YES
67	a	273.89	1.93017	YES	YES
68	a	276.64	1.49279	YES	YES
69	a	279.25	0.01480	YES	YES
70	a	280.27	0.55091	YES	YES
71	a	280.40	0.23410	YES	YES
72	a	280.94	0.09145	YES	YES
73	a	282.07	2.56101	YES	YES
74	a	283.58	1.39670	YES	YES
75	a	298.08	0.65742	YES	YES
76	a	300.10	5.04869	YES	YES
77	a	300.48	4.04148	YES	YES
78	a	302.73	8.50343	YES	YES
79	a	305.93	1.49979	YES	YES
80	a	306.98	0.19207	YES	YES
81	a	309.11	0.34698	YES	YES
82	a	313.99	0.43037	YES	YES
83	a	315.49	1.60075	YES	YES
84	a	316.90	0.98876	YES	YES
85	a	317.32	0.60297	YES	YES
86	a	318.36	2.27886	YES	YES

87	a	318.71	1.24846	YES	YES
88	a	319.97	0.39135	YES	YES
89	a	337.65	0.36140	YES	YES
90	a	339.09	1.43887	YES	YES
91	a	344.02	3.90231	YES	YES
92	a	347.50	8.24395	YES	YES
93	a	351.15	1.17167	YES	YES
94	a	354.88	9.98613	YES	YES
95	a	357.61	8.24255	YES	YES
96	a	371.16	0.15500	YES	YES
97	a	377.50	43.10384	YES	YES
98	a	381.84	3.97309	YES	YES
99	a	416.50	22.50635	YES	YES
100	a	425.17	51.28314	YES	YES
101	a	427.85	0.41925	YES	YES
102	a	451.09	83.08840	YES	YES
103	a	460.14	0.90989	YES	YES
104	a	503.98	0.72699	YES	YES
105	a	506.94	1.76537	YES	YES
106	a	509.36	1.10666	YES	YES
107	a	509.80	2.71946	YES	YES
108	a	511.29	3.75459	YES	YES
109	a	511.47	1.50829	YES	YES
110	a	513.06	3.12156	YES	YES
111	a	513.16	1.60423	YES	YES
112	a	514.14	3.96708	YES	YES
113	a	516.30	7.00541	YES	YES
114	a	517.54	3.05605	YES	YES
115	a	518.96	2.22092	YES	YES
116	a	520.61	0.19896	YES	YES
117	a	522.16	1.69322	YES	YES
118	a	532.52	20.56756	YES	YES
119	a	542.86	1.51909	YES	YES
120	a	543.98	7.34353	YES	YES
121	a	544.78	0.51027	YES	YES
122	a	545.42	1.55096	YES	YES
123	a	545.85	0.68738	YES	YES
124	a	546.08	1.17652	YES	YES
125	a	547.95	1.65211	YES	YES
126	a	548.36	0.55149	YES	YES
127	a	553.16	26.50029	YES	YES
128	a	566.46	2.27104	YES	YES
129	a	575.36	0.33715	YES	YES
130	a	641.01	0.52191	YES	YES
131	a	673.06	1.57506	YES	YES
132	a	690.03	0.10973	YES	YES
133	a	695.18	0.25906	YES	YES
134	a	696.57	10.85984	YES	YES
135	a	696.85	27.02340	YES	YES
136	a	697.88	22.02526	YES	YES
137	a	698.02	47.64036	YES	YES
138	a	699.42	34.66813	YES	YES
139	a	700.89	39.82565	YES	YES
140	a	701.31	26.94742	YES	YES
141	a	705.67	14.26626	YES	YES
142	a	711.34	39.34287	YES	YES
143	a	713.35	1.21224	YES	YES
144	a	722.13	2.82261	YES	YES
145	a	728.25	3.49984	YES	YES
146	a	762.92	11.76119	YES	YES
147	a	765.05	6.19071	YES	YES
148	a	805.39	12.22316	YES	YES
149	a	824.23	114.73856	YES	YES
150	a	850.31	13.55722	YES	YES
151	a	916.74	105.77204	YES	YES
152	a	926.57	2.46393	YES	YES
153	a	928.76	5.59519	YES	YES
154	a	933.50	21.23669	YES	YES
155	a	934.58	80.13220	YES	YES
156	a	935.86	200.93604	YES	YES
157	a	936.25	142.69382	YES	YES
158	a	939.46	385.16246	YES	YES
159	a	942.23	188.87160	YES	YES
160	a	1035.48	3.90941	YES	YES

161	a	1043.40	9.35918	YES	YES
162	a	1045.93	5.87429	YES	YES
163	a	1062.66	1.00213	YES	YES
164	a	1066.80	123.26822	YES	YES
165	a	1082.88	25.14961	YES	YES
166	a	1088.79	1.84763	YES	YES
167	a	1097.87	107.19326	YES	YES
168	a	1102.25	7.80119	YES	YES
169	a	1104.63	5.84324	YES	YES
170	a	1114.53	9.41089	YES	YES
171	a	1122.57	28.22914	YES	YES
172	a	1125.81	21.94524	YES	YES
173	a	1131.58	25.39306	YES	YES
174	a	1134.09	21.56242	YES	YES
175	a	1136.84	15.42236	YES	YES
176	a	1138.25	11.34632	YES	YES
177	a	1143.30	29.74587	YES	YES
178	a	1144.33	112.76861	YES	YES
179	a	1151.48	199.45733	YES	YES
180	a	1154.12	135.29989	YES	YES
181	a	1155.64	25.01072	YES	YES
182	a	1159.32	54.85339	YES	YES
183	a	1161.07	50.44491	YES	YES
184	a	1165.57	12.18613	YES	YES
185	a	1167.78	365.68172	YES	YES
186	a	1171.05	212.00326	YES	YES
187	a	1176.50	236.36429	YES	YES
188	a	1180.33	144.63060	YES	YES
189	a	1192.08	681.41533	YES	YES
190	a	1194.41	1121.49017	YES	YES
191	a	1196.66	1214.46403	YES	YES
192	a	1200.55	541.47630	YES	YES
193	a	1202.94	336.79205	YES	YES
194	a	1205.02	225.92575	YES	YES
195	a	1211.34	639.64946	YES	YES
196	a	1221.57	521.05687	YES	YES
197	a	1222.65	148.22232	YES	YES
198	a	1223.24	530.97296	YES	YES
199	a	1260.88	3.33667	YES	YES
200	a	1263.93	11.85340	YES	YES
201	a	1264.75	20.39765	YES	YES
202	a	1300.94	257.89338	YES	YES
203	a	1312.33	116.57980	YES	YES
204	a	1330.86	7.72067	YES	YES
205	a	1363.36	28.99605	YES	YES
206	a	1484.00	231.65071	YES	YES
207	a	1492.23	403.42864	YES	YES
208	a	1582.78	4.04385	YES	YES
209	a	1599.81	47.13322	YES	YES
210	a	3129.41	17.37456	YES	YES

$end

$[Ag(DCM)_1]^+$

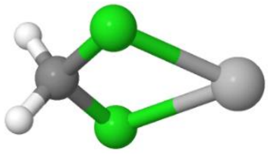

Method: (RI-)BP86(D3BJ)/def2-TZVPP
Symmetry: c2v

Cartesian coordinates in Ångström:

Ag	0.0000000	0.0000000	-2.5205569
Cl	0.0000000	-1.4799535	-0.3493477
Cl	0.0000000	1.4799535	-0.3493477
C	0.0000000	0.0000000	0.6756704
H	-0.9146146	0.0000000	1.2716933
H	0.9146146	0.0000000	1.2716933

SCF energy GEOOPT = -1106.721183053 H
ZPE = 76.31 kJ/mol

FREEH energy = 91.60 kJ/mol
FREEH entropy = 0.34501 kJ/mol/K

$vibrational spectrum

# mode	symmetry	wave number cm**(-1)	IR intensity km/mol	selection rules IR	RAMAN
1		-0.00	0.00000	-	-
2		-0.00	0.00000	-	-
3		-0.00	0.00000	-	-
4		0.00	0.00000	-	-
5		0.00	0.00000	-	-
6		0.00	0.00000	-	-
7	b1	34.94	7.91742	YES	YES
8	b2	102.52	0.68050	YES	YES
9	a1	154.98	4.09370	YES	YES
10	a1	316.35	1.38693	YES	YES
11	a1	664.95	18.36251	YES	YES
12	b2	666.02	76.82912	YES	YES
13	b1	877.49	1.13417	YES	YES
14	a2	1110.47	0.00000	NO	YES
15	b2	1232.77	10.34440	YES	YES
16	a1	1403.59	0.28983	YES	YES
17	a1	3050.73	5.72647	YES	YES
18	b1	3143.54	16.05319	YES	YES

$end

$[Ag(DCM)_2]^+$

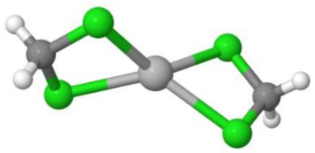

Method: (RI-)BP86(D3BJ)/def2-TZVPP
Symmetry: c1

Cartesian coordinates in Ångström:

Ag	-0.1276473	-0.0027146	0.0902964
Cl	-1.4264187	-2.2941792	0.1104574
Cl	-0.2903392	2.2038262	-1.4376671
Cl	1.5059228	-2.1438957	-0.1274852
Cl	0.0852992	2.2353689	1.4843983
C	-0.0493391	3.2449124	0.0019690
C	0.0957375	-3.2442448	-0.0292431
H	0.8858452	3.7938087	-0.1211377
H	-0.9212555	3.8923804	0.1097964
H	0.0466690	-3.8284105	-0.9497892
H	0.1955260	-3.8568517	0.8684048

SCF energy GEOOPT = -2066.631490833 H
ZPE = 152.6 kJ/mol
FREEH energy = 183.29 kJ/mol
FREEH entropy = 0.53252 kJ/mol/K

$vibrational spectrum

# mode	symmetry	wave number cm**(-1)	IR intensity km/mol	selection rules IR	RAMAN
1		0.00	0.00000	-	-
2		0.00	0.00000	-	-
3		0.00	0.00000	-	-
4		0.00	0.00000	-	-
5		0.00	0.00000	-	-
6		0.00	0.00000	-	-
7	a	5.93	0.38462	YES	YES
8	a	10.57	0.05670	YES	YES
9	a	12.45	0.47668	YES	YES
10	a	38.87	7.51689	YES	YES
11	a	39.66	7.50033	YES	YES
12	a	79.03	0.77883	YES	YES
13	a	82.72	1.06440	YES	YES
14	a	114.48	0.00336	YES	YES

15	a	170.64	11.77047	YES	YES
16	a	307.07	0.00910	YES	YES
17	a	311.53	4.28448	YES	YES
18	a	665.52	48.52395	YES	YES
19	a	667.54	52.60270	YES	YES
20	a	677.48	44.73799	YES	YES
21	a	680.15	46.20623	YES	YES
22	a	877.85	0.86296	YES	YES
23	a	877.88	0.86140	YES	YES
24	a	1110.92	0.00112	YES	YES
25	a	1111.29	0.00029	YES	YES
26	a	1233.02	9.52366	YES	YES
27	a	1233.20	10.46405	YES	YES
28	a	1406.56	0.20174	YES	YES
29	a	1406.66	0.13811	YES	YES
30	a	3052.17	3.87569	YES	YES
31	a	3052.23	0.18099	YES	YES
32	a	3141.95	9.63947	YES	YES
33	a	3141.97	9.68709	YES	YES

$end

$[Ag(DCM)_3]^+$

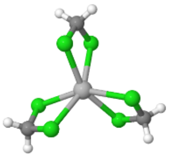

Method: (RI-)BP86(D3BJ)/def2-TZVPP
Symmetry: c3

Cartesian coordinates in Ångström:

Ag	0.0000000	0.0000000	-0.0360001
C	-0.3035012	3.3418675	-0.0063691
Cl	-0.8615620	2.2644006	1.3125483
Cl	0.4296418	2.3991988	-1.3437246
H	0.4600745	4.0082868	0.3975960
H	-1.1704424	3.8742696	-0.4004667
C	3.0458927	-1.4080940	-0.0063691
Cl	2.3918095	-0.3860657	1.3125483
Cl	1.8629462	-1.5716801	-1.3437246
H	3.2412410	-2.4025796	0.3975960
H	3.9404371	-0.9235020	-0.4004667
C	-2.7423915	-1.9337735	-0.0063691
Cl	-1.5302475	-1.8783349	1.3125483
Cl	-2.2925880	-0.8275187	-1.3437246
H	-3.7013154	-1.6057072	0.3975960
H	-2.7699947	-2.9507676	-0.4004667

SCF energy GEOOPT = -3026.522036894 H
ZPE =   228.6 kJ/mol
FREEH energy =   274.87 kJ/mol
FREEH entropy =   0.67358 kJ/mol/K

$vibrational spectrum

# mode	symmetry	wave number	IR intensity	selection rules	
#		cm**(-1)	km/mol	IR	RAMAN
1		-0.00	0.00000	-	-
2		-0.00	0.00000	-	-
3		0.00	0.00000	-	-
4		0.00	0.00000	-	-
5		0.00	0.00000	-	-
6		0.00	0.00000	-	-
7	a	11.72	0.64208	YES	YES
8	e	17.00	0.71297	YES	YES
9	e	17.00	0.71297	YES	YES
10	e	19.16	0.11095	YES	YES
11	e	19.16	0.11095	YES	YES
12	a	24.99	0.00060	YES	YES
13	e	38.39	7.90883	YES	YES
14	e	38.39	7.90883	YES	YES

15	a	39.21	3.36397	YES	YES
16	e	56.09	0.00446	YES	YES
17	e	56.09	0.00446	YES	YES
18	a	96.45	1.89002	YES	YES
19	a	106.01	0.00034	YES	YES
20	e	126.77	8.13986	YES	YES
21	e	126.77	8.13986	YES	YES
22	e	298.65	3.93523	YES	YES
23	e	298.65	3.93523	YES	YES
24	a	299.93	0.00016	YES	YES
25	e	670.00	39.34657	YES	YES
26	e	670.00	39.34657	YES	YES
27	e	675.79	17.84526	YES	YES
28	e	675.79	17.84526	YES	YES
29	a	675.85	7.49958	YES	YES
30	a	679.25	183.73699	YES	YES
31	a	876.34	0.38263	YES	YES
32	e	876.61	1.01731	YES	YES
33	e	876.61	1.01731	YES	YES
34	e	1116.59	0.00167	YES	YES
35	e	1116.59	0.00167	YES	YES
36	a	1116.71	0.00013	YES	YES
37	e	1232.07	4.09281	YES	YES
38	e	1232.07	4.09281	YES	YES
39	a	1236.11	22.73760	YES	YES
40	e	1407.67	0.57075	YES	YES
41	e	1407.67	0.57075	YES	YES
42	a	1407.89	0.00029	YES	YES
43	e	3052.95	0.57151	YES	YES
44	e	3052.95	0.57151	YES	YES
45	a	3053.01	0.00003	YES	YES
46	e	3140.14	7.47159	YES	YES
47	e	3140.14	7.47159	YES	YES
48	a	3140.15	3.47289	YES	YES

$end

$[Ag(DCM)_4]^+$

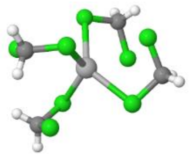

Method: (RI-)BP86(D3BJ)/def2-TZVPP
Symmetry: c1

Cartesian coordinates in Ångström:

Ag	-0.0042546	-0.0003722	0.0025802
C	0.1947928	-3.3978973	-0.5858354
H	-0.3141575	-3.5096846	-1.5440558
H	0.7114315	-4.3044484	-0.2667332
C	0.7446968	1.6269736	2.9543518
H	0.3570306	1.8428625	3.9512012
H	1.8183685	1.7977947	2.8660539
C	2.2103431	2.4133549	-1.0941725
H	2.9247984	2.6447893	-1.8857495
H	1.9049976	3.2908590	-0.5228138
C	-3.1481581	-0.6427281	-1.2741880
H	-3.4100914	-1.5867175	-0.7946052
H	-3.9874026	-0.1796741	-1.7955701
Cl	1.4458595	-2.1154917	-0.7857125
Cl	-1.0056926	-2.9565431	0.6480506
Cl	0.4500231	-0.1236393	2.6380860
Cl	-0.0951353	2.6569000	1.7751412
Cl	0.7351166	1.7476125	-1.8887926
Cl	2.9440487	1.2371921	0.0171576
Cl	-1.8403791	-0.9388818	-2.4397361
Cl	-2.6362361	0.4977394	0.0253420

SCF energy GEOOPT = -3986.407879765 H
ZPE = 306.8 kJ/mol

```
FREEH energy =   366.60 kJ/mol
FREEH entropy =   0.76518 kJ/mol/K

$vibrational spectrum
#  mode     symmetry     wave number   IR intensity    selection rules
#                         cm**(-1)        km/mol         IR     RAMAN
     1                      -0.00         0.00000         -       -
     2                      -0.00         0.00000         -       -
     3                      -0.00         0.00000         -       -
     4                      -0.00         0.00000         -       -
     5                       0.00         0.00000         -       -
     6                       0.00         0.00000         -       -
     7        a             22.70         0.00049       YES     YES
     8        a             24.39         0.00447       YES     YES
     9        a             25.89         0.00388       YES     YES
    10        a             33.83         0.90314       YES     YES
    11        a             34.06         0.86735       YES     YES
    12        a             40.87         0.00416       YES     YES
    13        a             42.24         1.06888       YES     YES
    14        a             44.15         0.01810       YES     YES
    15        a             50.10         0.00856       YES     YES
    16        a             62.92         1.42278       YES     YES
    17        a             63.47         1.28142       YES     YES
    18        a             64.28         0.27480       YES     YES
    19        a             66.49         0.00085       YES     YES
    20        a             81.89         0.39523       YES     YES
    21        a             83.89         0.01639       YES     YES
    22        a             87.03        13.42810       YES     YES
    23        a             88.26        13.61394       YES     YES
    24        a            109.28         7.32213       YES     YES
    25        a            120.36         0.00695       YES     YES
    26        a            135.75         4.82694       YES     YES
    27        a            136.41         4.81300       YES     YES
    28        a            288.55         2.16137       YES     YES
    29        a            288.56         2.13374       YES     YES
    30        a            290.44         0.00242       YES     YES
    31        a            292.33         3.98211       YES     YES
    32        a            652.03       158.56421       YES     YES
    33        a            653.36         2.74161       YES     YES
    34        a            654.69        38.57508       YES     YES
    35        a            655.16        38.80393       YES     YES
    36        a            697.94         1.22929       YES     YES
    37        a            700.67        92.39412       YES     YES
    38        a            701.10        97.90255       YES     YES
    39        a            701.84        15.45842       YES     YES
    40        a            871.49         3.21394       YES     YES
    41        a            871.54         3.18630       YES     YES
    42        a            873.38         0.04216       YES     YES
    43        a            873.69         0.00816       YES     YES
    44        a           1124.68         0.04335       YES     YES
    45        a           1124.74         0.21237       YES     YES
    46        a           1124.80         0.25373       YES     YES
    47        a           1125.19         0.08759       YES     YES
    48        a           1234.82         0.00920       YES     YES
    49        a           1235.86        17.95458       YES     YES
    50        a           1235.90        18.09684       YES     YES
    51        a           1238.34        29.50701       YES     YES
    52        a           1399.62         0.75700       YES     YES
    53        a           1399.77         4.40207       YES     YES
    54        a           1401.64         2.87274       YES     YES
    55        a           1401.75         2.85667       YES     YES
    56        a           3051.38         0.47636       YES     YES
    57        a           3051.43         0.47839       YES     YES
    58        a           3051.57         0.03207       YES     YES
    59        a           3051.62         0.01653       YES     YES
    60        a           3138.18        11.48953       YES     YES
    61        a           3138.25        11.39680       YES     YES
    62        a           3138.58         0.19706       YES     YES
    63        a           3138.65         0.12270       YES     YES
$end
```

$[Ag(DCE)_1]^+$

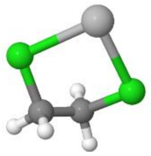

```
Method: (RI-)BP86(D3BJ)/def2-TZVPP
Symmetry: c2

Cartesian coordinates in Ångström:
Ag    0.0000000    0.0000000   -2.7282986
Cl    1.7434335   -0.2507910   -0.9013693
C     0.5679373   -0.4950970    0.4798593
C    -0.5679373    0.4950970    0.4798593
Cl   -1.7434335    0.2507910   -0.9013693
H     0.2369787   -1.5357761    0.4190931
H     1.1781577   -0.3453205    1.3787085
H    -0.2369787    1.5357761    0.4190931
H    -1.1781577    0.3453205    1.3787085

SCF energy GEOOPT =  -1146.078275655 H
ZPE =   150.3 kJ/mol
FREEH energy =   168.16 kJ/mol
FREEH entropy =   0.35765 kJ/mol/K

$vibrational spectrum
#  mode     symmetry     wave number   IR intensity    selection rules
#                         cm**(-1)        km/mol         IR      RAMAN
     1                      -0.00         0.00000         -        -
     2                      -0.00         0.00000         -        -
     3                       0.00         0.00000         -        -
     4                       0.00         0.00000         -        -
     5                       0.00         0.00000         -        -
     6                       0.00         0.00000         -        -
     7        b             66.38         6.53581        YES      YES
     8        a            120.04         0.85807        YES      YES
     9        b            161.00         2.30857        YES      YES
    10        a            220.87         5.43315        YES      YES
    11        a            265.60         0.00603        YES      YES
    12        b            402.21         9.61783        YES      YES
    13        a            589.00        25.98477        YES      YES
    14        b            610.31        22.45750        YES      YES
    15        b            851.40        13.85573        YES      YES
    16        a            914.12        14.90704        YES      YES
    17        a           1012.34         0.55871        YES      YES
    18        b           1122.56         0.00844        YES      YES
    19        a           1183.37         0.11414        YES      YES
    20        b           1256.32        12.49555        YES      YES
    21        a           1294.56        15.93450        YES      YES
    22        b           1410.42        22.51826        YES      YES
    23        a           1411.05         2.35562        YES      YES
    24        b           3021.42         7.75365        YES      YES
    25        a           3025.12         2.85719        YES      YES
    26        a           3088.30         6.70604        YES      YES
    27        b           3100.78         4.98008        YES      YES
$end
```

$[Ag(DCE)_2]^+$

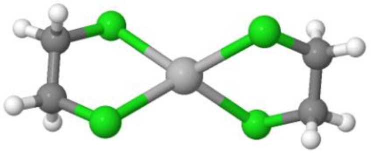

```
Method: (RI-)BP86(D3BJ)/def2-TZVPP
Symmetry: c2

Cartesian coordinates in Ångström:
C    -3.3485848   -0.6497376    0.1585469
C    -3.3131700    0.8141778   -0.1958043
```

Cl	-1.8668185	1.2571156	-1.2065266
Ag	-0.0000000	-0.0000000	0.1053560
Cl	-2.0051234	-1.1317829	1.2887965
Cl	2.0051234	1.1317829	1.2887965
C	3.3485848	0.6497376	0.1585469
C	3.3131700	-0.8141778	-0.1958043
Cl	1.8668185	-1.2571156	-1.2065266
H	-4.2707879	-0.8716286	0.7082094
H	-3.2664845	-1.3019982	-0.7156584
H	-3.2883822	1.4647371	0.6831579
H	-4.1797950	1.0598327	-0.8207570
H	3.2664845	1.3019982	-0.7156584
H	4.2707879	0.8716286	0.7082094
H	3.2883822	-1.4647371	0.6831579
H	4.1797950	-1.0598327	-0.8207570

SCF energy GEOOPT = -2145.335726354 H
ZPE = 300.8 kJ/mol
FREEH energy = 336.64 kJ/mol
FREEH entropy = 0.53326 kJ/mol/K

$vibrational spectrum

# mode	symmetry	wave number	IR intensity	selection rules	
#		cm**(-1)	km/mol	IR	RAMAN
1		-0.00	0.00000	-	-
2		0.00	0.00000	-	-
3		0.00	0.00000	-	-
4		0.00	0.00000	-	-
5		0.00	0.00000	-	-
6		0.00	0.00000	-	-
7	a	18.56	2.50710	YES	YES
8	a	20.38	0.02722	YES	YES
9	b	20.72	2.49337	YES	YES
10	b	56.46	5.78160	YES	YES
11	a	60.18	3.69902	YES	YES
12	a	93.03	0.00125	YES	YES
13	b	128.96	2.36193	YES	YES
14	a	131.86	3.24957	YES	YES
15	b	133.60	2.29239	YES	YES
16	a	196.32	0.00551	YES	YES
17	b	209.77	16.06561	YES	YES
18	a	260.23	0.00202	YES	YES
19	b	263.21	0.01285	YES	YES
20	b	403.91	9.32468	YES	YES
21	a	404.52	6.40584	YES	YES
22	b	606.61	59.62953	YES	YES
23	a	607.75	0.30777	YES	YES
24	a	627.85	24.06768	YES	YES
25	b	627.94	14.00827	YES	YES
26	b	854.30	3.10018	YES	YES
27	a	854.55	24.64913	YES	YES
28	b	920.18	35.14288	YES	YES
29	a	920.95	0.02892	YES	YES
30	b	1014.70	1.50612	YES	YES
31	a	1014.95	0.00549	YES	YES
32	a	1122.80	0.00843	YES	YES
33	b	1122.98	0.00186	YES	YES
34	b	1183.02	0.11479	YES	YES
35	a	1183.17	0.00005	YES	YES
36	b	1257.81	3.52865	YES	YES
37	a	1259.28	16.32007	YES	YES
38	b	1293.75	44.43656	YES	YES
39	a	1294.68	0.04443	YES	YES
40	b	1414.28	36.65880	YES	YES
41	a	1414.41	2.17006	YES	YES
42	a	1415.73	0.09262	YES	YES
43	b	1415.78	5.58178	YES	YES
44	a	3020.89	5.87774	YES	YES
45	b	3020.90	3.17592	YES	YES
46	b	3024.41	0.03332	YES	YES
47	a	3024.42	0.01906	YES	YES
48	b	3084.78	7.22944	YES	YES
49	a	3084.79	0.01877	YES	YES
50	b	3097.75	0.06422	YES	YES

```
    51         a            3097.75         3.26576       YES     YES
$end
```

$[Ag(DCE)_3]^+$

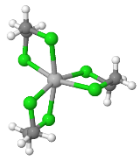

```
Method: (RI-)BP86(D3BJ)/def2-TZVPP
Symmetry: c1

Cartesian coordinates in Ångström:
Ag    3.0396243    4.1236351    1.3229776
Cl    4.5590316    4.5195961   -0.9404314
Cl    1.6044787    6.0695842    0.0224752
Cl    4.8404346    5.5690582    2.7868185
Cl    1.6316351    4.5370739    3.6193682
Cl    4.3773834    1.7795424    1.7619195
Cl    1.2457651    2.2833670    0.3515952
C     4.0825446    5.5417491    4.4337368
H     4.2959667    4.5573243    4.8603375
H     4.6048089    6.3167258    5.0069196
C     2.6067842    5.8506280    4.4020634
H     2.3833088    6.7730820    3.8581900
H     2.2264865    5.9221728    5.4277631
C     2.3076910    5.9731774   -1.6454080
H     1.9144536    5.0587698   -2.0993596
H     1.9174295    6.8492874   -2.1763576
C     3.8152650    6.0157724   -1.6444917
H     4.2094102    6.8595621   -1.0706788
H     4.1803217    6.0652808   -2.6769397
C     1.9687604    0.7153274    0.9049340
H     1.6980124    0.6018546    1.9587303
H     1.4747684   -0.0603672    0.3083560
C     3.4588590    0.6493181    0.6822410
H     3.7409131    0.8977945   -0.3451414
H     3.8218832   -0.3536962    0.9352925

SCF energy GEOOPT =  -3144.564314813 H
ZPE =   449.6 kJ/mol
FREEH energy =   504.69 kJ/mol
FREEH entropy =   0.74677 kJ/mol/K

$vibrational spectrum
#  mode     symmetry     wave number   IR intensity    selection rules
#                         cm**(-1)        km/mol         IR     RAMAN
     1                       -0.00         0.00000        -       -
     2                        0.00         0.00000        -       -
     3                        0.00         0.00000        -       -
     4                        0.00         0.00000        -       -
     5                        0.00         0.00000        -       -
     6                        0.00         0.00000        -       -
     7         a              1.69         1.91306       YES     YES
     8         a              8.56         1.83815       YES     YES
     9         a             21.53         0.26975       YES     YES
    10         a             22.59         0.53984       YES     YES
    11         a             25.22         0.18156       YES     YES
    12         a             27.36         0.02401       YES     YES
    13         a             43.09         3.05212       YES     YES
    14         a             47.81         7.79179       YES     YES
    15         a             49.50         5.36157       YES     YES
    16         a             77.07         0.00388       YES     YES
    17         a             78.08         0.07855       YES     YES
    18         a             87.62         0.01483       YES     YES
    19         a             99.42         2.75687       YES     YES
    20         a            101.05         3.06393       YES     YES
    21         a            131.09         6.64866       YES     YES
    22         a            168.73        11.69074       YES     YES
    23         a            171.95        12.49072       YES     YES
```

24	a	174.79	0.68738	YES	YES
25	a	255.64	0.95329	YES	YES
26	a	256.19	0.86442	YES	YES
27	a	257.68	0.15420	YES	YES
28	a	399.35	7.90758	YES	YES
29	a	399.95	8.30698	YES	YES
30	a	400.12	5.51746	YES	YES
31	a	616.45	42.60658	YES	YES
32	a	616.92	37.92299	YES	YES
33	a	617.93	2.32249	YES	YES
34	a	637.12	8.14423	YES	YES
35	a	637.47	8.59154	YES	YES
36	a	639.94	37.82674	YES	YES
37	a	857.18	11.99229	YES	YES
38	a	857.72	12.01368	YES	YES
39	a	857.90	13.25078	YES	YES
40	a	917.42	26.36811	YES	YES
41	a	917.72	30.18445	YES	YES
42	a	918.87	0.37698	YES	YES
43	a	1016.59	1.50037	YES	YES
44	a	1016.68	1.14574	YES	YES
45	a	1016.88	0.18647	YES	YES
46	a	1124.04	0.07748	YES	YES
47	a	1124.29	0.06692	YES	YES
48	a	1124.72	0.01346	YES	YES
49	a	1184.74	0.34049	YES	YES
50	a	1184.88	0.44712	YES	YES
51	a	1185.03	0.25201	YES	YES
52	a	1260.32	9.44633	YES	YES
53	a	1260.50	8.76870	YES	YES
54	a	1262.71	15.21320	YES	YES
55	a	1293.24	0.50686	YES	YES
56	a	1293.80	35.88515	YES	YES
57	a	1294.47	29.12329	YES	YES
58	a	1415.09	33.30962	YES	YES
59	a	1415.51	5.52057	YES	YES
60	a	1415.83	4.90756	YES	YES
61	a	1416.10	8.46722	YES	YES
62	a	1416.18	4.53273	YES	YES
63	a	1416.46	3.95068	YES	YES
64	a	3017.49	2.86169	YES	YES
65	a	3017.55	2.89677	YES	YES
66	a	3017.57	3.23912	YES	YES
67	a	3021.54	1.25676	YES	YES
68	a	3021.59	1.50800	YES	YES
69	a	3021.63	1.71695	YES	YES
70	a	3079.78	1.59619	YES	YES
71	a	3079.81	1.68155	YES	YES
72	a	3079.93	1.47174	YES	YES
73	a	3092.45	0.46708	YES	YES
74	a	3092.48	0.23357	YES	YES
75	a	3092.71	0.37727	YES	YES

$end

$[Ag(AN)_1]^+$

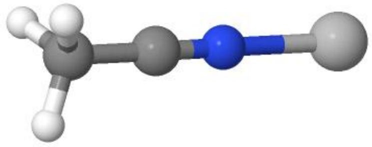

Method: (RI-)BP86(D3BJ)/def2-TZVPP
Symmetry: c3v

Cartesian coordinates in Ångström:

Ag	0.0000000	0.0000000	-3.5863239
N	0.0000000	0.0000000	-1.5132962
C	-0.0000000	0.0000000	-0.3556530
C	-0.0000000	0.0000000	1.0865801
H	-0.5172494	0.8959023	1.4563860
H	-0.5172494	-0.8959023	1.4563860

```
H     1.0344989    0.0000000    1.4563860

SCF energy GEOOPT =  -279.6972779020 H
ZPE =   118.3 kJ/mol
FREEH energy =   132.87 kJ/mol
FREEH entropy =   0.31091 kJ/mol/K

$vibrational spectrum
#  mode     symmetry     wave number   IR intensity    selection rules
#                         cm**(-1)        km/mol         IR     RAMAN
     1                      -0.00         0.00000         -       -
     2                      -0.00         0.00000         -       -
     3                      -0.00         0.00000         -       -
     4                       0.00         0.00000         -       -
     5                       0.00         0.00000         -       -
     6                       0.00         0.00000         -       -
     7         e            99.67        13.13565        YES     YES
     8         e            99.67        13.13565        YES     YES
     9         a1          270.62         1.31757        YES     YES
    10         e           389.96         1.32357        YES     YES
    11         e           389.96         1.32357        YES     YES
    12         a1          954.49         0.11452        YES     YES
    13         e          1008.13         6.22543        YES     YES
    14         e          1008.13         6.22543        YES     YES
    15         a1         1352.50        10.26236        YES     YES
    16         e          1399.73        16.88299        YES     YES
    17         e          1399.73        16.88299        YES     YES
    18         a1         2311.88       119.93103        YES     YES
    19         a1         2979.86        21.66438        YES     YES
    20         e          3059.47         9.49899        YES     YES
    21         e          3059.47         9.49899        YES     YES
$end
```

$[Ag(AN)_2]^+$

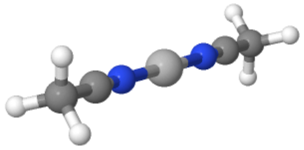

```
Method: (RI-)BP86(D3BJ)/def2-TZVPP
Symmetry: d3d

Cartesian coordinates in Ångström:
Ag    0.0000000    0.0000000    0.0000000
N     0.0000000    0.0000000   -2.0267674
C     0.0000000   -0.0000000   -3.1837631
C    -0.0000000   -0.0000000   -4.6274014
N     0.0000000    0.0000000    2.0267674
C     0.0000000    0.0000000    3.1837631
C    -0.0000000   -0.0000000    4.6274014
H     0.0000000   -1.0336515   -4.9984190
H     0.8951684    0.5168257   -4.9984190
H    -0.8951684    0.5168257   -4.9984190
H     0.0000000    1.0336515    4.9984190
H    -0.8951684   -0.5168257    4.9984190
H     0.8951684   -0.5168257    4.9984190

SCF energy GEOOPT =  -412.5955838589 H
ZPE =   239.0 kJ/mol
FREEH energy =   266.56 kJ/mol
FREEH entropy =   0.42475 kJ/mol/K

$vibrational spectrum
#  mode     symmetry     wave number   IR intensity    selection rules
#                         cm**(-1)        km/mol         IR     RAMAN
     1                      -0.00         0.00000         -       -
     2                      -0.00         0.00000         -       -
     3                       0.00         0.00000         -       -
     4                       0.00         0.00000         -       -
     5                       0.00         0.00000         -       -
```

6		0.00	0.00000	-	-
7	a1u	30.82	0.00000	NO	NO
8	eu	33.60	10.59739	YES	NO
9	eu	33.60	10.59739	YES	NO
10	eg	105.12	0.00000	NO	YES
11	eg	105.12	0.00000	NO	YES
12	eu	171.08	11.45957	YES	NO
13	eu	171.08	11.45957	YES	NO
14	a1g	248.75	0.00000	NO	YES
15	a2u	334.97	0.00143	YES	NO
16	eg	391.37	0.00000	NO	YES
17	eg	391.37	0.00000	NO	YES
18	eu	397.67	2.44523	YES	NO
19	eu	397.67	2.44523	YES	NO
20	a1g	958.96	0.00000	NO	YES
21	a2u	959.90	2.38190	YES	NO
22	eu	1010.87	11.01938	YES	NO
23	eu	1010.87	11.01938	YES	NO
24	eg	1010.94	0.00000	NO	YES
25	eg	1010.94	0.00000	NO	YES
26	a2u	1355.09	12.61011	YES	NO
27	a1g	1355.36	0.00000	NO	YES
28	eu	1404.62	31.18120	YES	NO
29	eu	1404.62	31.18120	YES	NO
30	eg	1404.64	0.00000	NO	YES
31	eg	1404.64	0.00000	NO	YES
32	a1g	2319.66	0.00000	NO	YES
33	a2u	2319.68	139.78453	YES	NO
34	a2u	2982.21	31.69457	YES	NO
35	a1g	2982.35	0.00000	NO	YES
36	eg	3061.18	0.00000	NO	YES
37	eg	3061.18	0.00000	NO	YES
38	eu	3061.19	12.43337	YES	NO
39	eu	3061.19	12.43337	YES	NO

$end

$[Ag(AN)_3]^+$

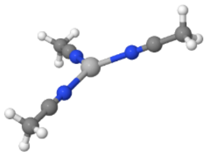

Method: (RI-)BP86(D3BJ)/def2-TZVPP
Symmetry: c1

Cartesian coordinates in Ångström:

C	3.8287237	-1.0717096	-2.1871127
C	2.6863153	-1.3950558	-1.3588992
H	1.7588117	-1.2349750	-1.9238884
H	2.6765415	-0.7559306	-0.4665135
H	2.7370572	-2.4460887	-1.0461779
C	7.1538060	-2.1639935	-6.7561916
C	7.4615043	-2.9666818	-7.9209361
H	7.7289628	-3.9855079	-7.6118101
H	8.3044441	-2.5259682	-8.4687792
H	6.5880254	-3.0125420	-8.5842439
C	8.3602893	2.2811423	-3.3225708
C	9.1952335	3.4170802	-2.9937108
H	8.9599928	3.7760837	-1.9834231
H	9.0199400	4.2288222	-3.7116890
H	10.2533032	3.1273858	-3.0336316
N	4.7425618	-0.8131094	-2.8500885
N	6.9072996	-1.5221256	-5.8241197
N	7.6923265	1.3723902	-3.5862464
Ag	6.4464313	-0.3226462	-4.0856174

SCF energy GEOOPT = -545.4369320339 H
ZPE = 354.4 kJ/mol
FREEH energy = 398.63 kJ/mol
FREEH entropy = 0.66544 kJ/mol/K

```
$vibrational spectrum
#  mode     symmetry     wave number   IR intensity    selection rules
#                         cm**(-1)        km/mol         IR     RAMAN
     1                       -0.00         0.00000        -       -
     2                       -0.00         0.00000        -       -
     3                        0.00         0.00000        -       -
     4                        0.00         0.00000        -       -
     5                        0.00         0.00000        -       -
     6                        0.00         0.00000        -       -
     7                        0.71         0.02305        -       -
     8        a              11.41         0.43462       YES     YES
     9        a              11.66         2.45352       YES     YES
    10        a              12.08         2.13616       YES     YES
    11        a              23.03         0.06112       YES     YES
    12        a              26.52        14.12851       YES     YES
    13        a              71.62         0.02501       YES     YES
    14        a              72.18         0.02470       YES     YES
    15        a              74.17         0.00533       YES     YES
    16        a              92.87        15.15062       YES     YES
    17        a              93.06        15.00374       YES     YES
    18        a             133.03        15.48577       YES     YES
    19        a             198.42         0.00029       YES     YES
    20        a             216.35         0.07623       YES     YES
    21        a             216.94         0.07209       YES     YES
    22        a             379.99         0.00017       YES     YES
    23        a             383.71         0.02557       YES     YES
    24        a             383.99         0.03156       YES     YES
    25        a             384.28         0.85934       YES     YES
    26        a             384.33         0.82711       YES     YES
    27        a             388.00         1.54931       YES     YES
    28        a             939.59         0.12574       YES     YES
    29        a             939.65         0.12902       YES     YES
    30        a             942.51         0.00023       YES     YES
    31        a            1014.50         0.58113       YES     YES
    32        a            1014.61         1.25952       YES     YES
    33        a            1014.68         4.57247       YES     YES
    34        a            1014.72         5.56263       YES     YES
    35        a            1014.72         1.84604       YES     YES
    36        a            1014.82        10.31614       YES     YES
    37        a            1357.93         4.87981       YES     YES
    38        a            1358.08         5.34467       YES     YES
    39        a            1358.61         0.09688       YES     YES
    40        a            1411.74         8.12567       YES     YES
    41        a            1411.84        14.55223       YES     YES
    42        a            1411.92        19.04543       YES     YES
    43        a            1412.54         2.07102       YES     YES
    44        a            1412.61        14.37338       YES     YES
    45        a            1412.68        25.51500       YES     YES
    46        a            2302.29        42.62048       YES     YES
    47        a            2302.34        42.48036       YES     YES
    48        a            2304.38         0.00959       YES     YES
    49        a            2984.89         6.13449       YES     YES
    50        a            2984.90         6.14066       YES     YES
    51        a            2984.95         0.02070       YES     YES
    52        a            3062.01         2.31274       YES     YES
    53        a            3062.08         2.04860       YES     YES
    54        a            3062.12         2.62827       YES     YES
    55        a            3062.28         2.05737       YES     YES
    56        a            3062.32         2.15492       YES     YES
    57        a            3062.37         2.15815       YES     YES
$end
```

$[Ag(AN)_4]^+$

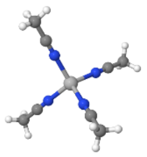

Method: (RI-)BP86(D3BJ)/def2-TZVPP

Symmetry: c1

Cartesian coordinates in Ångström:

Ag	-0.8911603	0.3121146	-0.0089111
N	-2.1966222	-1.0470515	-1.2298829
N	0.3145265	-0.9156588	1.4313655
N	0.4854425	1.4400036	-1.3742500
N	-2.1821930	1.7506807	1.1299331
C	-2.8717928	-1.7551571	-1.8506904
C	-2.8540361	2.4930833	1.7131140
C	1.1969264	2.0144175	-2.0859110
C	0.9333270	-1.5492611	2.1785472
C	-3.7165252	-2.6411984	-2.6273146
H	-3.9340804	-2.1922883	-3.6049538
H	-4.6621330	-2.8179555	-2.0988792
H	-3.2106606	-3.6028355	-2.7822529
C	-3.6945984	3.4220119	2.4426473
H	-4.4105909	3.8970998	1.7597724
H	-3.0776094	4.2007471	2.9090592
H	-4.2495161	2.8909060	3.2266334
C	2.0870849	2.7329638	-2.9763854
H	1.9849304	3.8142373	-2.8181654
H	1.8413233	2.5015908	-4.0207541
H	3.1275757	2.4420255	-2.7830922
C	1.7075040	-2.3419613	3.1134217
H	1.6662055	-3.4019923	2.8319034
H	1.3039847	-2.2274510	4.1276686
H	2.7546374	-2.0131710	3.1081373

SCF energy GEOOPT = -678.2744018457 H
ZPE =   471.0 kJ/mol
FREEH energy =   531.03 kJ/mol
FREEH entropy =   0.79621 kJ/mol/K

$vibrational spectrum

# mode	symmetry	wave number	IR intensity	selection rules	
#		cm**(-1)	km/mol	IR	RAMAN
1		-0.00	0.00000	-	-
2		-0.00	0.00000	-	-
3		-0.00	0.00000	-	-
4		0.00	0.00000	-	-
5		0.00	0.00000	-	-
6		0.00	0.00000	-	-
7	a	10.41	0.01577	YES	YES
8	a	15.34	0.06914	YES	YES
9	a	15.58	0.06323	YES	YES
10	a	15.71	0.19654	YES	YES
11	a	18.17	6.15693	YES	YES
12	a	18.53	6.35203	YES	YES
13	a	18.98	6.63892	YES	YES
14	a	22.06	0.01676	YES	YES
15	a	31.00	0.00097	YES	YES
16	a	52.14	0.00059	YES	YES
17	a	53.80	0.03850	YES	YES
18	a	54.23	0.00724	YES	YES
19	a	82.54	21.73151	YES	YES
20	a	83.16	21.61834	YES	YES
21	a	84.27	21.75889	YES	YES
22	a	90.54	0.20632	YES	YES
23	a	91.45	0.21789	YES	YES
24	a	171.63	0.06401	YES	YES
25	a	171.86	0.00405	YES	YES
26	a	172.34	0.06692	YES	YES
27	a	172.94	0.06557	YES	YES
28	a	377.40	0.00083	YES	YES
29	a	377.52	0.00024	YES	YES
30	a	377.55	0.00096	YES	YES
31	a	380.01	0.02151	YES	YES
32	a	380.08	0.06429	YES	YES
33	a	380.53	0.96820	YES	YES
34	a	380.65	0.98339	YES	YES
35	a	380.73	0.97456	YES	YES
36	a	932.99	0.00578	YES	YES
37	a	933.06	0.00791	YES	YES

38	a	933.12	0.00842	YES	YES
39	a	936.24	0.00035	YES	YES
40	a	1016.57	0.27604	YES	YES
41	a	1016.61	0.08678	YES	YES
42	a	1016.64	0.25254	YES	YES
43	a	1016.74	2.80426	YES	YES
44	a	1016.78	4.94318	YES	YES
45	a	1016.81	3.51968	YES	YES
46	a	1016.85	7.47878	YES	YES
47	a	1016.92	6.12588	YES	YES
48	a	1359.56	3.23949	YES	YES
49	a	1359.57	3.18030	YES	YES
50	a	1359.74	3.14112	YES	YES
51	a	1360.43	0.06620	YES	YES
52	a	1416.18	6.18906	YES	YES
53	a	1416.22	6.56011	YES	YES
54	a	1416.24	8.00544	YES	YES
55	a	1416.32	22.99972	YES	YES
56	a	1416.36	17.73917	YES	YES
57	a	1416.45	15.96555	YES	YES
58	a	1416.46	12.88859	YES	YES
59	a	1416.53	12.66106	YES	YES
60	a	2297.15	19.69761	YES	YES
61	a	2297.23	19.82855	YES	YES
62	a	2297.29	20.02576	YES	YES
63	a	2300.22	0.00027	YES	YES
64	a	2985.34	0.90321	YES	YES
65	a	2985.36	0.72818	YES	YES
66	a	2985.42	0.84256	YES	YES
67	a	2985.44	0.57833	YES	YES
68	a	3061.36	0.74480	YES	YES
69	a	3061.39	0.76310	YES	YES
70	a	3061.45	0.43245	YES	YES
71	a	3061.45	1.15874	YES	YES
72	a	3061.46	0.67471	YES	YES
73	a	3061.47	0.78515	YES	YES
74	a	3061.50	0.84093	YES	YES
75	a	3061.52	0.73852	YES	YES

$end

$[Ag(DMF)_1]^+$

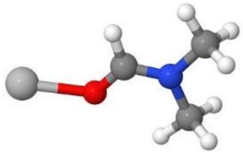

Method: (RI-)BP86(D3BJ)/def2-TZVPP
Symmetry: cs

Cartesian coordinates in Ångström:

Ag	-4.1140542	-0.3974635	0.0000000
C	-1.1319338	0.3259412	0.0000000
O	-2.0147637	-0.5900720	0.0000000
N	0.1646090	0.0679734	0.0000000
C	1.1495998	1.1510306	0.0000000
C	0.6808277	-1.3026135	0.0000000
H	-1.3943811	1.3931713	0.0000000
H	0.6403204	2.1201814	0.0000000
H	1.7820320	1.0741640	0.8934363
H	1.7820320	1.0741640	-0.8934363
H	-0.1539838	-2.0066274	0.0000000
H	1.2995947	-1.4566832	-0.8930148
H	1.2995947	-1.4566832	0.8930148

SCF energy GEOOPT = -395.5266141124 H
ZPE = 265.5 kJ/mol
FREEH energy = 287.01 kJ/mol
FREEH entropy = 0.38761 kJ/mol/K

$vibrational spectrum

```
#   mode     symmetry     wave number   IR intensity    selection rules
#                          cm**(-1)        km/mol         IR      RAMAN
      1                      -0.00         0.00000         -        -
      2                      -0.00         0.00000         -        -
      3                      -0.00         0.00000         -        -
      4                      -0.00         0.00000         -        -
      5                      -0.00         0.00000         -        -
      6                       0.00         0.00000         -        -
      7         a"           54.51         6.76441        YES      YES
      8         a'           99.10         3.40985        YES      YES
      9         a"           99.40         0.00750        YES      YES
     10         a"          164.88         0.12743        YES      YES
     11         a'          188.68         2.14417        YES      YES
     12         a"          256.40         0.09601        YES      YES
     13         a'          375.21        11.51258        YES      YES
     14         a"          382.74        15.52228        YES      YES
     15         a'          399.15         6.78348        YES      YES
     16         a'          677.36        18.84448        YES      YES
     17         a'          844.27         4.37819        YES      YES
     18         a"          961.45         2.50336        YES      YES
     19         a'         1033.69        10.81171        YES      YES
     20         a"         1084.53         0.28547        YES      YES
     21         a'         1103.19        27.76342        YES      YES
     22         a"         1136.93         0.86077        YES      YES
     23         a'         1219.30        15.25931        YES      YES
     24         a'         1351.26       144.95739        YES      YES
     25         a'         1397.79         1.49516        YES      YES
     26         a'         1404.18        15.66855        YES      YES
     27         a'         1418.48        63.82211        YES      YES
     28         a"         1427.25         3.34310        YES      YES
     29         a'         1445.51         8.79113        YES      YES
     30         a"         1448.67        27.27505        YES      YES
     31         a'         1483.32        39.03283        YES      YES
     32         a'         1640.40       647.51574        YES      YES
     33         a'         2982.90         6.72572        YES      YES
     34         a'         2988.54         5.67802        YES      YES
     35         a'         3020.75        26.75616        YES      YES
     36         a"         3052.72         5.14849        YES      YES
     37         a"         3054.81         0.19630        YES      YES
     38         a'         3083.13         2.78133        YES      YES
     39         a'         3112.05         0.50510        YES      YES
$end
```

$[Ag(DMF)_2]^+$

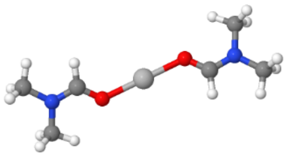

```
Method: (RI-)BP86(D3BJ)/def2-TZVPP
Symmetry: c2

Cartesian coordinates in Ångström:
Ag    0.0000000   -0.0000000   -0.1109862
O    -1.8654683   -0.9091791   -0.1380964
C    -2.9901400   -0.3771311    0.0805164
C     2.9901400    0.3771311    0.0805164
O     1.8654683    0.9091791   -0.1380964
N    -4.1349917   -1.0471186    0.0095155
C    -4.1716813   -2.4676051   -0.3349307
H    -3.0955394    0.6854546    0.3469502
H    -4.7750336   -2.6094409   -1.2409101
H    -4.6282984   -3.0329942    0.4877921
H    -3.1531115   -2.8220995   -0.5082160
C    -5.4122991   -0.3894794    0.2739291
H    -6.0573892   -0.4562916   -0.6118637
H    -5.2450763    0.6650904    0.5172845
H    -5.9165025   -0.8778560    1.1180475
N     4.1349917    1.0471186    0.0095155
H     3.0955394   -0.6854546    0.3469502
```

```
C     4.1716813    2.4676051   -0.3349307
H     4.7750336    2.6094409   -1.2409101
H     4.6282984    3.0329942    0.4877921
H     3.1531115    2.8220995   -0.5082160
C     5.4122991    0.3894794    0.2739291
H     6.0573892    0.4562916   -0.6118637
H     5.2450763   -0.6650904    0.5172845
H     5.9165025    0.8778560    1.1180475

SCF energy GEOOPT =  -644.2430045767 H
ZPE =   532.3 kJ/mol
FREEH energy =   574.15 kJ/mol
FREEH entropy =   0.56134 kJ/mol/K

$vibrational spectrum
#  mode     symmetry     wave number   IR intensity    selection rules
#                         cm**(-1)        km/mol         IR     RAMAN
     1                      -0.00         0.00000         -       -
     2                       0.00         0.00000         -       -
     3                       0.00         0.00000         -       -
     4                       0.00         0.00000         -       -
     5                       0.00         0.00000         -       -
     6                       0.00         0.00000         -       -
     7        a             17.98         5.15839        YES     YES
     8        a             21.23         0.83141        YES     YES
     9        b             25.68         3.49099        YES     YES
    10        b             60.48         0.06273        YES     YES
    11        a             85.99         0.21299        YES     YES
    12        a            106.54         0.10394        YES     YES
    13        b            107.95         0.00237        YES     YES
    14        a            126.29         4.37147        YES     YES
    15        b            141.16         2.03145        YES     YES
    16        a            154.08         0.00529        YES     YES
    17        b            167.88         0.04211        YES     YES
    18        a            170.06         0.73343        YES     YES
    19        b            227.16         3.79738        YES     YES
    20        b            254.64         0.01168        YES     YES
    21        a            255.88         0.05340        YES     YES
    22        a            372.61        19.72455        YES     YES
    23        b            373.18         1.31389        YES     YES
    24        a            374.34         9.28190        YES     YES
    25        b            389.23        10.81172        YES     YES
    26        a            396.56         0.00044        YES     YES
    27        b            412.10        28.19839        YES     YES
    28        a            681.69         0.02067        YES     YES
    29        b            693.68        57.34307        YES     YES
    30        a            850.14         0.00052        YES     YES
    31        b            850.65         3.66938        YES     YES
    32        a            965.58         3.38210        YES     YES
    33        b            965.63         0.21569        YES     YES
    34        b           1038.31        20.72565        YES     YES
    35        a           1038.40         0.64231        YES     YES
    36        b           1086.77         0.03005        YES     YES
    37        a           1086.80         0.60465        YES     YES
    38        b           1099.13       103.65754        YES     YES
    39        a           1100.00         0.00210        YES     YES
    40        b           1138.04         0.11706        YES     YES
    41        a           1138.05         1.80843        YES     YES
    42        a           1225.51         1.68973        YES     YES
    43        b           1225.75        32.22647        YES     YES
    44        b           1356.95       248.27659        YES     YES
    45        a           1359.34         0.77144        YES     YES
    46        b           1397.12         8.33399        YES     YES
    47        a           1397.14         0.48805        YES     YES
    48        a           1403.62         0.92937        YES     YES
    49        b           1403.93        13.89561        YES     YES
    50        b           1419.84       132.11147        YES     YES
    51        a           1421.39         0.20497        YES     YES
    52        a           1429.22         6.51326        YES     YES
    53        b           1429.24         0.45548        YES     YES
    54        b           1448.15        17.28752        YES     YES
    55        a           1448.43         1.45794        YES     YES
    56        b           1450.29         3.40351        YES     YES
    57        a           1450.42        43.20769        YES     YES
```

58	b	1488.46	73.05967	YES	YES
59	a	1490.42	1.91129	YES	YES
60	b	1637.90	1631.53570	YES	YES
61	a	1653.02	1.82041	YES	YES
62	b	2977.22	25.22058	YES	YES
63	a	2977.25	0.57813	YES	YES
64	b	2983.24	26.98609	YES	YES
65	a	2983.28	0.45427	YES	YES
66	b	3003.03	55.59495	YES	YES
67	a	3003.44	1.57595	YES	YES
68	b	3043.24	1.15902	YES	YES
69	a	3043.25	17.38505	YES	YES
70	b	3046.85	0.17050	YES	YES
71	a	3046.85	2.64833	YES	YES
72	a	3077.21	0.32073	YES	YES
73	b	3077.21	8.81528	YES	YES
74	b	3107.71	1.38700	YES	YES
75	a	3107.72	0.07840	YES	YES

$end

$[Ag(DMF)_3]^+$

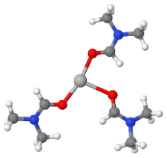

Method: (RI-)BP86(D3BJ)/def2-TZVPP
Symmetry: c1

Cartesian coordinates in Ångström:

Ag	-1.3861680	1.9844343	-0.1189804
O	-2.6654817	0.2716474	-0.0532795
C	-3.9190840	0.2982263	0.0383397
O	-0.5098716	3.9350873	-0.2127703
C	0.7377891	4.0875538	-0.1559376
O	0.9140345	0.9550520	-0.0352441
C	1.0363236	-0.2500167	0.2469885
N	-4.6901432	-0.7871046	-0.0473878
H	-4.4754249	1.2353514	0.2002305
C	-6.1404535	-0.6964105	0.0859426
H	-6.4798881	-1.2993431	0.9388936
H	-6.6265966	-1.0680742	-0.8259719
H	-6.4331551	0.3465838	0.2475495
C	-4.1186515	-2.1108545	-0.2742182
H	-4.5585383	-2.5520672	-1.1782707
H	-4.3378256	-2.7644055	0.5809194
H	-3.0374277	-2.0134540	-0.3985455
N	2.1714521	-0.9615930	0.1272433
H	0.1878096	-0.8493005	0.6298545
C	2.2293824	-2.3667970	0.5039525
H	2.9656459	-2.5200170	1.3054849
H	2.5171029	-2.9838497	-0.3588009
H	1.2457697	-2.6918455	0.8614650
C	3.3945994	-0.3499824	-0.3747349
H	3.7647571	-0.9113451	-1.2434096
H	4.1708713	-0.3542632	0.4033616
H	3.1740139	0.6793931	-0.6699218
N	1.3308880	5.2815973	-0.2151616
C	0.5576236	6.5120132	-0.3540390
H	-0.5048963	6.2601728	-0.3968946
H	0.8529284	7.0347535	-1.2736402
H	0.7491819	7.1704069	0.5038865
C	2.7814960	5.4048406	-0.1427154
H	3.2304263	4.4114882	-0.0369421
H	3.0668892	6.0207286	0.7210372
H	3.1682191	5.8781633	-1.0555780
H	1.4183621	3.2278500	-0.0537751

SCF energy GEOOPT = -892.9101395556 H
ZPE = 794.5 kJ/mol

FREEH energy = 858.86 kJ/mol
FREEH entropy = 0.79267 kJ/mol/K

$vibrational spectrum

# mode	symmetry	wave number cm**(-1)	IR intensity km/mol	selection rules IR	RAMAN
1		-0.00	0.00000	-	-
2		-0.00	0.00000	-	-
3		-0.00	0.00000	-	-
4		0.00	0.00000	-	-
5		0.00	0.00000	-	-
6		0.00	0.00000	-	-
7	a	2.96	0.17582	YES	YES
8	a	12.74	0.36050	YES	YES
9	a	15.55	3.79353	YES	YES
10	a	16.71	0.86605	YES	YES
11	a	21.84	2.16248	YES	YES
12	a	28.82	2.00747	YES	YES
13	a	45.74	1.66757	YES	YES
14	a	53.98	0.13749	YES	YES
15	a	76.11	3.20914	YES	YES
16	a	89.76	1.89475	YES	YES
17	a	95.25	5.50939	YES	YES
18	a	101.73	2.92025	YES	YES
19	a	109.76	0.62433	YES	YES
20	a	116.34	1.74924	YES	YES
21	a	120.24	2.98849	YES	YES
22	a	124.17	4.28192	YES	YES
23	a	146.83	0.69798	YES	YES
24	a	169.26	0.04342	YES	YES
25	a	170.35	0.02022	YES	YES
26	a	171.27	0.46937	YES	YES
27	a	210.51	4.58163	YES	YES
28	a	243.26	0.23450	YES	YES
29	a	249.76	0.01902	YES	YES
30	a	250.94	0.04889	YES	YES
31	a	327.61	24.86689	YES	YES
32	a	357.93	11.19190	YES	YES
33	a	363.10	9.70375	YES	YES
34	a	370.32	4.83725	YES	YES
35	a	374.22	15.57236	YES	YES
36	a	385.39	31.86508	YES	YES
37	a	387.37	0.77062	YES	YES
38	a	391.98	0.52879	YES	YES
39	a	398.00	22.88752	YES	YES
40	a	650.86	25.69576	YES	YES
41	a	670.54	1.12175	YES	YES
42	a	678.32	53.78784	YES	YES
43	a	852.43	0.20158	YES	YES
44	a	853.22	0.43834	YES	YES
45	a	853.50	1.49379	YES	YES
46	a	968.64	1.53727	YES	YES
47	a	972.79	0.83525	YES	YES
48	a	991.80	1.15064	YES	YES
49	a	1039.84	15.03726	YES	YES
50	a	1039.93	4.14908	YES	YES
51	a	1040.21	7.10125	YES	YES
52	a	1080.66	91.09713	YES	YES
53	a	1087.12	0.36299	YES	YES
54	a	1087.45	2.23921	YES	YES
55	a	1088.57	0.49542	YES	YES
56	a	1092.83	134.67784	YES	YES
57	a	1094.51	9.72022	YES	YES
58	a	1136.84	1.59584	YES	YES
59	a	1137.77	1.29200	YES	YES
60	a	1138.21	1.33306	YES	YES
61	a	1228.96	15.60491	YES	YES
62	a	1232.65	20.45795	YES	YES
63	a	1232.95	19.82168	YES	YES
64	a	1362.39	136.28001	YES	YES
65	a	1365.15	64.73676	YES	YES
66	a	1369.24	58.73489	YES	YES
67	a	1390.24	6.36283	YES	YES
68	a	1393.90	27.25804	YES	YES

69	a	1394.33	8.11705	YES	YES
70	a	1395.89	14.21598	YES	YES
71	a	1401.78	8.01651	YES	YES
72	a	1402.71	12.38549	YES	YES
73	a	1417.60	62.20183	YES	YES
74	a	1418.83	39.94621	YES	YES
75	a	1420.17	21.58769	YES	YES
76	a	1428.85	5.36716	YES	YES
77	a	1429.32	1.18058	YES	YES
78	a	1429.41	3.34966	YES	YES
79	a	1448.58	0.79524	YES	YES
80	a	1448.73	25.34142	YES	YES
81	a	1450.23	11.95487	YES	YES
82	a	1450.59	20.46940	YES	YES
83	a	1451.11	12.17296	YES	YES
84	a	1452.93	7.74282	YES	YES
85	a	1490.06	46.18320	YES	YES
86	a	1491.60	11.88115	YES	YES
87	a	1495.04	14.61230	YES	YES
88	a	1633.11	1477.63137	YES	YES
89	a	1642.19	377.88106	YES	YES
90	a	1661.15	488.75350	YES	YES
91	a	2926.14	48.71705	YES	YES
92	a	2963.86	30.84781	YES	YES
93	a	2969.65	16.92915	YES	YES
94	a	2970.39	37.38582	YES	YES
95	a	2972.62	15.17986	YES	YES
96	a	2976.74	24.46187	YES	YES
97	a	2977.66	18.54817	YES	YES
98	a	2988.76	36.56285	YES	YES
99	a	3002.44	37.72067	YES	YES
100	a	3022.18	19.53768	YES	YES
101	a	3028.42	3.92830	YES	YES
102	a	3030.60	14.20765	YES	YES
103	a	3035.73	13.94800	YES	YES
104	a	3037.22	4.43670	YES	YES
105	a	3039.55	1.25954	YES	YES
106	a	3063.97	8.79606	YES	YES
107	a	3072.63	6.89140	YES	YES
108	a	3073.92	5.12930	YES	YES
109	a	3091.94	2.49736	YES	YES
110	a	3101.20	0.89124	YES	YES
111	a	3101.40	1.26793	YES	YES

$end

$[Ag(DMF)_4]^+$

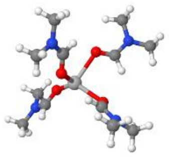

Method: (RI-)BP86(D3BJ)/def2-TZVPP
Symmetry: c1

Cartesian coordinates in Ångström:

Ag	-0.8939087	-0.2652034	-0.0068624
C	-3.6433372	-1.7737318	-0.1014624
O	-3.0616017	-0.6949604	0.1362864
C	0.2927903	2.6432700	-0.4062533
O	-0.0494397	1.9465739	0.5677169
C	1.5666362	-0.9592099	-1.8428798
O	0.5381538	-0.2593672	-1.9247391
C	1.3421345	-0.4589400	2.1907327
O	0.8900859	-1.2170365	1.3093712
N	-4.9686462	-1.9489765	0.0030313
C	-5.5884106	-3.2338051	-0.2903594
C	-5.8431050	-0.8562598	0.4135034
N	0.4787346	3.9754376	-0.3652685
C	0.8867651	4.7179118	-1.5482815
C	0.2662045	4.7275605	0.8639890

```
N     2.4015566   -1.2059157   -2.8681553
C     3.5892978   -2.0284094   -2.6927122
C     2.1443969   -0.6658118   -4.1965765
N     2.1194041   -0.8595698    3.2126388
C     2.6144677    0.0882781    4.1999806
C     2.4857354   -2.2597262    3.3781800
H    -3.0999584   -2.6806153   -0.4210367
H     0.4749225    2.1982284   -1.4021841
H     1.8684503   -1.4352667   -0.8921802
H     1.1285852    0.6253510    2.1859294
H    -6.3107552   -3.1306121   -1.1120379
H    -4.8187832   -3.9568085   -0.5828765
H    -6.1161692   -3.6142002    0.5952830
H    -6.3843923   -1.1336365    1.3283525
H    -5.2327553    0.0312702    0.6000216
H    -6.5744561   -0.6440263   -0.3783911
H     0.1335375    5.4763309   -1.8047676
H     1.8468094    5.2245632   -1.3742581
H     0.9968780    4.0284314   -2.3929449
H     1.2017482    5.2081060    1.1837336
H    -0.4901228    5.5078517    0.7012298
H    -0.0788091    4.0373173    1.6389077
H     3.5499039   -2.9063745   -3.3530449
H     4.4953735   -1.4531821   -2.9309372
H     3.6478999   -2.3692215   -1.6528730
H     2.9620602    0.0028765   -4.5007116
H     2.0713815   -1.4833030   -4.9271126
H     1.2025271   -0.1105789   -4.1707368
H     2.2581357   -0.1818245    5.2041679
H     3.7137598    0.0957635    4.2100326
H     2.2542429    1.0935824    3.9543848
H     3.5771125   -2.3772597    3.3203505
H     2.1463968   -2.6240001    4.3577468
H     2.0085621   -2.8408709    2.5840728

SCF energy GEOOPT =  -1141.572816218 H
ZPE =   1057. kJ/mol
FREEH energy =  1143.06 kJ/mol
FREEH entropy =   0.98485 kJ/mol/K

$vibrational spectrum
#  mode     symmetry     wave number   IR intensity    selection rules
#                         cm**(-1)        km/mol         IR     RAMAN
     1                      -0.00         0.00000        -       -
     2                      -0.00         0.00000        -       -
     3                      -0.00         0.00000        -       -
     4                      -0.00         0.00000        -       -
     5                      -0.00         0.00000        -       -
     6                      -0.00         0.00000        -       -
     7         a             4.07         1.95640       YES     YES
     8         a             6.16         0.60844       YES     YES
     9         a            10.09         0.63310       YES     YES
    10         a            16.70         0.66737       YES     YES
    11         a            18.94         1.01824       YES     YES
    12         a            20.05         0.25994       YES     YES
    13         a            29.41         0.86966       YES     YES
    14         a            29.69         0.83458       YES     YES
    15         a            38.41         0.99106       YES     YES
    16         a            46.08         2.82088       YES     YES
    17         a            57.91         2.97337       YES     YES
    18         a            65.10         8.19276       YES     YES
    19         a            68.57         5.41041       YES     YES
    20         a            74.98         2.97847       YES     YES
    21         a            86.13         4.92684       YES     YES
    22         a            88.37         7.85727       YES     YES
    23         a            93.26         8.65650       YES     YES
    24         a           104.50         2.45264       YES     YES
    25         a           105.48         2.56828       YES     YES
    26         a           117.40         0.07711       YES     YES
    27         a           125.51         0.10970       YES     YES
    28         a           131.67         0.53510       YES     YES
    29         a           132.30         0.48995       YES     YES
    30         a           135.60         1.08965       YES     YES
    31         a           166.66        14.11321       YES     YES
```

32	a	169.17	0.13690	YES	YES
33	a	172.83	0.54673	YES	YES
34	a	174.29	0.22870	YES	YES
35	a	175.79	0.99146	YES	YES
36	a	243.54	0.04695	YES	YES
37	a	243.62	0.03486	YES	YES
38	a	244.47	0.05788	YES	YES
39	a	245.96	0.09921	YES	YES
40	a	331.71	38.57841	YES	YES
41	a	333.12	39.49646	YES	YES
42	a	344.77	1.15203	YES	YES
43	a	353.40	18.57644	YES	YES
44	a	357.51	13.12066	YES	YES
45	a	363.96	6.10181	YES	YES
46	a	365.17	6.12112	YES	YES
47	a	371.02	30.79539	YES	YES
48	a	387.32	4.16884	YES	YES
49	a	387.55	3.18876	YES	YES
50	a	387.71	4.60294	YES	YES
51	a	389.23	4.99918	YES	YES
52	a	651.23	26.00865	YES	YES
53	a	651.69	22.44935	YES	YES
54	a	653.74	17.65282	YES	YES
55	a	665.11	21.65250	YES	YES
56	a	851.97	0.22488	YES	YES
57	a	852.91	0.74289	YES	YES
58	a	853.71	0.80397	YES	YES
59	a	853.89	1.04611	YES	YES
60	a	967.18	1.09164	YES	YES
61	a	987.75	1.58277	YES	YES
62	a	991.87	1.39495	YES	YES
63	a	993.48	1.42728	YES	YES
64	a	1040.88	7.76531	YES	YES
65	a	1041.12	7.93579	YES	YES
66	a	1041.18	7.17203	YES	YES
67	a	1041.28	6.13662	YES	YES
68	a	1078.66	137.38530	YES	YES
69	a	1079.46	126.05517	YES	YES
70	a	1085.15	124.51166	YES	YES
71	a	1087.67	0.32531	YES	YES
72	a	1088.46	23.42443	YES	YES
73	a	1088.85	1.35039	YES	YES
74	a	1088.96	0.31534	YES	YES
75	a	1089.00	0.63065	YES	YES
76	a	1137.51	1.35201	YES	YES
77	a	1137.66	2.23970	YES	YES
78	a	1137.67	1.14467	YES	YES
79	a	1137.70	1.83954	YES	YES
80	a	1231.69	18.35105	YES	YES
81	a	1234.80	24.69999	YES	YES
82	a	1235.01	17.62264	YES	YES
83	a	1235.27	14.57772	YES	YES
84	a	1363.15	90.44095	YES	YES
85	a	1365.14	99.50929	YES	YES
86	a	1365.87	101.67429	YES	YES
87	a	1367.82	21.28396	YES	YES
88	a	1389.19	4.93874	YES	YES
89	a	1389.68	21.87958	YES	YES
90	a	1389.78	24.57974	YES	YES
91	a	1392.74	6.75742	YES	YES
92	a	1396.30	20.18118	YES	YES
93	a	1396.96	19.66254	YES	YES
94	a	1397.63	9.12457	YES	YES
95	a	1398.83	17.11692	YES	YES
96	a	1418.00	24.47685	YES	YES
97	a	1418.27	21.91449	YES	YES
98	a	1418.59	61.22685	YES	YES
99	a	1419.66	3.35336	YES	YES
100	a	1428.78	3.98468	YES	YES
101	a	1429.70	3.54357	YES	YES
102	a	1429.77	2.19033	YES	YES
103	a	1429.99	2.71041	YES	YES
104	a	1450.37	12.61065	YES	YES
105	a	1450.53	7.52798	YES	YES

```
   106        a           1450.68         24.19448       YES      YES
   107        a           1450.81         18.27685       YES      YES
   108        a           1451.45          8.98663       YES      YES
   109        a           1452.77         12.83713       YES      YES
   110        a           1452.85         11.64234       YES      YES
   111        a           1453.23          2.95308       YES      YES
   112        a           1493.65         20.62997       YES      YES
   113        a           1493.81         10.29008       YES      YES
   114        a           1494.22         11.45680       YES      YES
   115        a           1495.21         11.37455       YES      YES
   116        a           1638.51       1254.31379       YES      YES
   117        a           1640.28        826.90046       YES      YES
   118        a           1643.47        957.29314       YES      YES
   119        a           1661.66         21.11486       YES      YES
   120        a           2944.48         24.44470       YES      YES
   121        a           2953.11         13.41292       YES      YES
   122        a           2954.68         34.61450       YES      YES
   123        a           2955.64          8.01592       YES      YES
   124        a           2961.26         35.62336       YES      YES
   125        a           2961.56         36.23983       YES      YES
   126        a           2961.83         35.90057       YES      YES
   127        a           2965.91         37.31629       YES      YES
   128        a           2967.75         46.17558       YES      YES
   129        a           2968.20         53.63806       YES      YES
   130        a           2968.62         34.72091       YES      YES
   131        a           2972.93         33.11619       YES      YES
   132        a           3017.78         18.70918       YES      YES
   133        a           3018.12         19.13293       YES      YES
   134        a           3018.31         27.68236       YES      YES
   135        a           3024.61          5.56743       YES      YES
   136        a           3024.90          9.86976       YES      YES
   137        a           3024.94         13.36606       YES      YES
   138        a           3025.27          6.04165       YES      YES
   139        a           3031.48          4.72022       YES      YES
   140        a           3063.53          8.67976       YES      YES
   141        a           3064.12          8.30159       YES      YES
   142        a           3064.83          7.86834       YES      YES
   143        a           3066.55          7.80570       YES      YES
   144        a           3089.99          1.45333       YES      YES
   145        a           3090.07          1.34213       YES      YES
   146        a           3090.15          1.55001       YES      YES
   147        a           3096.43          0.89343       YES      YES
$end
```

$[Ag(THF)_1]^+$

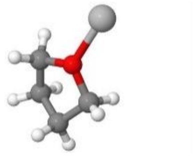

```
Method: (RI-)BP86(D3BJ)/def2-TZVPP
Symmetry: c1

Cartesian coordinates in Ångström:
C    -5.3473226    3.7148061    0.0294314
O    -5.8972081    2.3398012   -0.1278042
C    -4.9357063    1.3417226    0.4004499
C    -3.6213339    2.0969703    0.4383312
C    -4.0604673    3.5232289    0.8166115
H    -3.3107075    4.2757374    0.5481432
H    -4.2499589    3.5962881    1.8952055
H    -6.0997147    4.3217594    0.5453235
H    -5.1801070    4.0986581   -0.9847549
H    -4.9558740    0.4894847   -0.2879238
H    -5.2744163    1.0350089    1.3993752
H    -2.9293283    1.6664794    1.1709600
H    -3.1378685    2.0817194   -0.5468277
Ag   -7.9487165    1.9161156   -0.5130809

SCF energy GEOOPT =  -379.4366163956 H
```

```
ZPE =   301.0 kJ/mol
FREEH energy =   319.65 kJ/mol
FREEH entropy =   0.36833 kJ/mol/K

$vibrational spectrum
#  mode     symmetry     wave number   IR intensity    selection rules
#                         cm**(-1)        km/mol         IR     RAMAN
     1                      -0.00         0.00000         -       -
     2                      -0.00         0.00000         -       -
     3                       0.00         0.00000         -       -
     4                       0.00         0.00000         -       -
     5                       0.00         0.00000         -       -
     6                       0.00         0.00000         -       -
     7        a             48.30         2.46033        YES     YES
     8        a             89.64         5.00228        YES     YES
     9        a            116.33         0.60381        YES     YES
    10        a            206.58         4.19072        YES     YES
    11        a            248.40         0.17942        YES     YES
    12        a            551.85         1.90396        YES     YES
    13        a            667.59         0.53366        YES     YES
    14        a            809.17        37.72534        YES     YES
    15        a            817.21        98.75803        YES     YES
    16        a            831.92         3.63697        YES     YES
    17        a            901.33         8.60829        YES     YES
    18        a            908.38         2.11871        YES     YES
    19        a            944.70        25.50079        YES     YES
    20        a            973.67        40.45575        YES     YES
    21        a           1030.48         2.34773        YES     YES
    22        a           1119.77         5.15082        YES     YES
    23        a           1136.69         3.01390        YES     YES
    24        a           1145.76         2.52656        YES     YES
    25        a           1213.65         0.76187        YES     YES
    26        a           1235.60        10.81246        YES     YES
    27        a           1285.73         0.54666        YES     YES
    28        a           1313.37         0.12772        YES     YES
    29        a           1330.74         3.69760        YES     YES
    30        a           1345.80         9.07870        YES     YES
    31        a           1439.95         7.93721        YES     YES
    32        a           1445.77         8.96909        YES     YES
    33        a           1464.67         1.51144        YES     YES
    34        a           1474.88         6.34646        YES     YES
    35        a           2986.98        17.72328        YES     YES
    36        a           2997.76        13.40670        YES     YES
    37        a           3001.53         1.82174        YES     YES
    38        a           3007.62         8.08847        YES     YES
    39        a           3052.21         4.00719        YES     YES
    40        a           3053.82         3.26240        YES     YES
    41        a           3062.94         0.03280        YES     YES
    42        a           3070.42        11.06102        YES     YES
$end
```

$[Ag(THF)_2]^+$

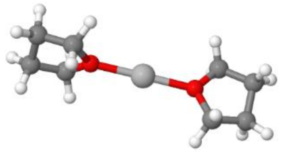

```
Method: (RI-)BP86(D3BJ)/def2-TZVPP
Symmetry: c2

Cartesian coordinates in Ångström:
Ag    0.0000000   -0.0000000    0.0018611
O     1.3855093   -1.5622140    0.0020810
C     2.6349424   -1.5810888    0.7893726
H     2.3875596   -1.2520001    1.8048665
H     3.3302809   -0.8700059    0.3230115
C     3.1171561   -3.0196030    0.6964652
H     2.6575183   -3.6333994    1.4821446
H     4.2057881   -3.0897468    0.7981269
C     2.6241649   -3.4559646   -0.6938819
```

```
H     2.5630459   -4.5450852   -0.7960643
H     3.2882585   -3.0728796   -1.4796444
C     1.2539407   -2.8045437   -0.7857002
H     0.9562580   -2.5190400   -1.8008948
H     0.4647700   -3.4097056   -0.3191557
O    -1.3855093    1.5622140    0.0020810
C    -2.6349424    1.5810888    0.7893726
H    -2.3875596    1.2520001    1.8048665
H    -3.3302809    0.8700059    0.3230115
C    -3.1171561    3.0196030    0.6964652
H    -2.6575183    3.6333994    1.4821446
H    -4.2057881    3.0897468    0.7981269
C    -2.6241649    3.4559646   -0.6938819
H    -3.2882585    3.0728796   -1.4796444
H    -2.5630459    4.5450852   -0.7960643
C    -1.2539407    2.8045437   -0.7857002
H    -0.9562580    2.5190400   -1.8008948
H    -0.4647700    3.4097056   -0.3191557

SCF energy GEOOPT =  -612.0698397463 H
ZPE =   603.8 kJ/mol
FREEH energy =   640.29 kJ/mol
FREEH entropy =   0.54045 kJ/mol/K

$vibrational spectrum
#  mode     symmetry     wave number   IR intensity    selection rules
#                         cm**(-1)        km/mol         IR     RAMAN
     1                      -0.00         0.00000         -       -
     2                      -0.00         0.00000         -       -
     3                      -0.00         0.00000         -       -
     4                      -0.00         0.00000         -       -
     5                      -0.00         0.00000         -       -
     6                      -0.00         0.00000         -       -
     7        a             10.50         0.07530        YES     YES
     8        b             15.91         2.30072        YES     YES
     9        a             16.50         2.45070        YES     YES
    10        b             57.19         0.21042        YES     YES
    11        a             62.51         0.67872        YES     YES
    12        a             79.31         2.92865        YES     YES
    13        b             80.06         2.89644        YES     YES
    14        a            145.37         0.44842        YES     YES
    15        b            147.54         0.03595        YES     YES
    16        a            162.60         0.00003        YES     YES
    17        b            245.31         1.15222        YES     YES
    18        a            246.65         0.00005        YES     YES
    19        b            264.58         7.10740        YES     YES
    20        b            555.35         2.92734        YES     YES
    21        a            555.43         0.01004        YES     YES
    22        a            675.84         0.00007        YES     YES
    23        b            681.79         0.23935        YES     YES
    24        a            827.29        21.12743        YES     YES
    25        b            827.78        17.42351        YES     YES
    26        b            831.55        70.99206        YES     YES
    27        a            833.58         0.10663        YES     YES
    28        a            846.53         0.00023        YES     YES
    29        b            848.91       213.48197        YES     YES
    30        a            901.14         0.68100        YES     YES
    31        b            901.48         9.46072        YES     YES
    32        a            909.12         0.00030        YES     YES
    33        b            909.26         2.98925        YES     YES
    34        a            949.78         4.79282        YES     YES
    35        b            949.98        13.07575        YES     YES
    36        a            985.18        43.73128        YES     YES
    37        b            985.93        75.14060        YES     YES
    38        b           1028.59        10.78707        YES     YES
    39        a           1028.89         0.00023        YES     YES
    40        b           1125.66         8.53866        YES     YES
    41        a           1125.72         0.00001        YES     YES
    42        b           1143.03         4.65150        YES     YES
    43        a           1143.12         3.43142        YES     YES
    44        b           1149.45         5.90574        YES     YES
    45        a           1149.51         0.00129        YES     YES
    46        a           1217.76         0.00002        YES     YES
    47        b           1217.79         0.66138        YES     YES
```

48	a	1234.94	14.09738	YES	YES
49	b	1235.21	5.17778	YES	YES
50	b	1285.54	0.20810	YES	YES
51	a	1285.54	0.47831	YES	YES
52	b	1312.29	0.04112	YES	YES
53	a	1312.41	0.00014	YES	YES
54	a	1332.31	2.52876	YES	YES
55	b	1332.36	4.20830	YES	YES
56	b	1347.87	17.47980	YES	YES
57	a	1348.41	0.00000	YES	YES
58	a	1442.09	14.26738	YES	YES
59	b	1442.09	0.82634	YES	YES
60	b	1448.29	22.15402	YES	YES
61	a	1448.35	0.17462	YES	YES
62	b	1467.54	1.93089	YES	YES
63	a	1467.59	0.01365	YES	YES
64	b	1477.72	9.99778	YES	YES
65	a	1478.15	0.00160	YES	YES
66	a	2985.92	8.09714	YES	YES
67	b	2986.09	65.40612	YES	YES
68	b	2987.65	0.40016	YES	YES
69	a	2987.77	0.01995	YES	YES
70	b	2998.15	8.31832	YES	YES
71	a	2998.15	0.00965	YES	YES
72	a	3004.94	13.39281	YES	YES
73	b	3004.96	8.00323	YES	YES
74	b	3048.81	0.07063	YES	YES
75	a	3048.81	23.28428	YES	YES
76	a	3049.84	0.07229	YES	YES
77	b	3049.87	0.59047	YES	YES
78	b	3060.21	2.30467	YES	YES
79	a	3060.22	0.00095	YES	YES
80	a	3068.07	10.97736	YES	YES
81	b	3068.08	16.91948	YES	YES

$end

$[Ag(THF)_3]^+$

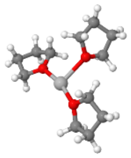

Method: (RI-)BP86(D3BJ)/def2-TZVPP
Symmetry: c1

Cartesian coordinates in Ångström:

C	-6.2165769	2.0337363	-0.7353951
C	-6.8433585	1.0382835	0.2551365
C	-5.8437526	-0.1074073	0.2434206
H	-7.8447758	0.7130645	-0.0480103
H	-6.9135400	1.4793543	1.2583153
O	-4.5416279	0.5416640	0.0388326
H	-6.0134339	-0.8024928	-0.5914540
H	-5.7841160	-0.6667364	1.1839778
C	-4.7287520	1.9191604	-0.4306144
H	-4.0812464	2.0681221	-1.3027306
H	-4.4123743	2.5919646	0.3793737
H	-6.5849151	3.0567288	-0.5996710
H	-6.4230647	1.7295120	-1.7700643
C	-0.8730574	-3.0696347	-0.4374045
O	-0.9909003	-1.8211437	0.3271726
C	0.5064392	-3.6048525	-0.0862040
H	-0.9650678	-2.8311996	-1.5070474
H	-1.7033124	-3.7160405	-0.1302584
C	1.3215412	-2.3149513	0.1064844
H	0.9098492	-4.2488407	-0.8756385
H	0.4701541	-4.1841795	0.8459208
C	0.3331414	-1.3971746	0.8117406
H	1.6151299	-1.8960469	-0.8655285
H	2.2293959	-2.4719298	0.6995697

```
H     0.4462036   -0.3334010    0.5739245
H     0.3375442   -1.5342381    1.9021677
C    -1.2759644    2.5864792    1.1945529
O    -1.2363713    1.6071254    0.1114144
C    -0.7892790    2.2523952   -1.1165118
C    -0.1939255    3.5853100   -0.6804895
H    -1.6576857    2.3932254   -1.7810006
H    -0.0738169    1.5800098   -1.6066791
C    -1.0668905    3.9443489    0.5319606
H    -0.2313383    4.3390443   -1.4750891
H     0.8544314    3.4586384   -0.3779038
H    -2.0261455    4.3668492    0.2017910
H    -0.5918345    4.6666162    1.2052131
H    -0.4700225    2.3439528    1.9034424
H    -2.2410892    2.4877706    1.7093674
Ag   -2.6194248   -0.4233068    0.1469562

SCF energy GEOOPT =  -844.6597437838 H
ZPE =   903.0 kJ/mol
FREEH energy =   958.50 kJ/mol
FREEH entropy =   0.73132 kJ/mol/K

$vibrational spectrum
#  mode     symmetry     wave number   IR intensity    selection rules
#                         cm**(-1)        km/mol         IR     RAMAN
     1                       -0.00         0.00000        -       -
     2                       -0.00         0.00000        -       -
     3                        0.00         0.00000        -       -
     4                        0.00         0.00000        -       -
     5                        0.00         0.00000        -       -
     6                        0.00         0.00000        -       -
     7         a              4.67         0.12100       YES     YES
     8         a             12.63         0.28343       YES     YES
     9         a             14.39         0.95102       YES     YES
    10         a             19.54         0.77926       YES     YES
    11         a             26.58         0.37333       YES     YES
    12         a             37.58         0.33648       YES     YES
    13         a             40.81         0.77442       YES     YES
    14         a             49.32         1.28143       YES     YES
    15         a             58.60         0.19474       YES     YES
    16         a             74.14         0.04069       YES     YES
    17         a             81.02         2.98179       YES     YES
    18         a             94.12         3.46130       YES     YES
    19         a            107.75         2.40585       YES     YES
    20         a            120.27         6.25577       YES     YES
    21         a            120.91         4.34953       YES     YES
    22         a            139.00         3.21395       YES     YES
    23         a            154.61         0.91952       YES     YES
    24         a            235.10         8.83505       YES     YES
    25         a            246.16         0.08055       YES     YES
    26         a            247.00         0.20883       YES     YES
    27         a            250.67         0.29231       YES     YES
    28         a            556.44         1.18278       YES     YES
    29         a            557.45         1.18180       YES     YES
    30         a            558.90         1.03935       YES     YES
    31         a            660.72         6.53096       YES     YES
    32         a            671.93         0.21894       YES     YES
    33         a            677.45         1.73839       YES     YES
    34         a            820.18        30.26728       YES     YES
    35         a            830.52         3.24228       YES     YES
    36         a            831.39         6.09856       YES     YES
    37         a            834.87        23.04138       YES     YES
    38         a            837.53        14.12326       YES     YES
    39         a            844.21        91.68622       YES     YES
    40         a            845.66        10.33232       YES     YES
    41         a            846.64       147.58853       YES     YES
    42         a            857.04        40.34996       YES     YES
    43         a            894.47         3.02655       YES     YES
    44         a            899.53         1.58487       YES     YES
    45         a            900.07         7.97505       YES     YES
    46         a            909.77         3.65494       YES     YES
    47         a            910.13         2.96230       YES     YES
    48         a            911.00         3.09545       YES     YES
    49         a            944.00         0.90386       YES     YES
```

50	a	948.41	2.54925	YES	YES
51	a	950.20	6.63226	YES	YES
52	a	994.29	41.38740	YES	YES
53	a	998.15	93.19212	YES	YES
54	a	1009.75	66.85226	YES	YES
55	a	1020.50	4.03769	YES	YES
56	a	1024.91	8.45954	YES	YES
57	a	1026.35	4.29429	YES	YES
58	a	1127.47	1.39511	YES	YES
59	a	1127.88	2.63876	YES	YES
60	a	1128.48	3.18037	YES	YES
61	a	1144.10	3.81102	YES	YES
62	a	1144.71	2.29152	YES	YES
63	a	1147.22	2.70509	YES	YES
64	a	1150.33	4.50904	YES	YES
65	a	1151.11	3.94942	YES	YES
66	a	1152.16	4.37988	YES	YES
67	a	1217.41	0.44006	YES	YES
68	a	1218.99	1.20066	YES	YES
69	a	1219.80	0.14670	YES	YES
70	a	1229.64	7.08743	YES	YES
71	a	1233.31	7.11956	YES	YES
72	a	1233.59	7.73929	YES	YES
73	a	1279.83	0.33437	YES	YES
74	a	1283.23	1.01542	YES	YES
75	a	1283.67	0.53932	YES	YES
76	a	1304.21	0.33412	YES	YES
77	a	1309.23	0.31316	YES	YES
78	a	1309.43	0.04000	YES	YES
79	a	1323.59	2.08352	YES	YES
80	a	1329.51	1.54956	YES	YES
81	a	1330.39	2.22169	YES	YES
82	a	1346.15	5.36719	YES	YES
83	a	1347.80	7.23806	YES	YES
84	a	1348.16	4.39675	YES	YES
85	a	1441.10	7.07883	YES	YES
86	a	1441.74	5.06689	YES	YES
87	a	1442.37	6.89746	YES	YES
88	a	1448.17	9.71763	YES	YES
89	a	1448.66	10.30221	YES	YES
90	a	1449.85	9.94804	YES	YES
91	a	1466.42	0.25911	YES	YES
92	a	1469.71	2.67117	YES	YES
93	a	1471.42	0.57002	YES	YES
94	a	1478.43	5.42567	YES	YES
95	a	1480.90	0.57715	YES	YES
96	a	1483.71	2.51483	YES	YES
97	a	2940.02	44.08732	YES	YES
98	a	2962.84	27.72365	YES	YES
99	a	2971.89	26.12120	YES	YES
100	a	2972.97	50.96082	YES	YES
101	a	2976.74	9.33916	YES	YES
102	a	2981.04	17.41247	YES	YES
103	a	2985.09	8.75422	YES	YES
104	a	2992.57	6.55955	YES	YES
105	a	2994.79	11.16293	YES	YES
106	a	2994.89	3.42803	YES	YES
107	a	3000.78	13.26051	YES	YES
108	a	3001.64	11.32422	YES	YES
109	a	3013.32	33.71975	YES	YES
110	a	3021.37	23.28699	YES	YES
111	a	3035.03	14.15010	YES	YES
112	a	3042.66	13.95902	YES	YES
113	a	3045.24	6.76308	YES	YES
114	a	3045.34	2.98758	YES	YES
115	a	3051.84	6.09548	YES	YES
116	a	3055.81	2.44893	YES	YES
117	a	3056.05	2.68312	YES	YES
118	a	3058.63	18.99377	YES	YES
119	a	3063.51	15.96450	YES	YES
120	a	3064.27	15.85425	YES	YES

$end

$[Ag(THF)_4]^+$

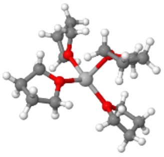

```
Method: (RI-)BP86(D3BJ)/def2-TZVPP
Symmetry: c1

Cartesian coordinates in Ångström:
Ag   -0.6297978    0.1268911    0.1840549
C    -3.0233638   -0.7752491   -3.4318800
C    -4.0426668    0.2178611   -2.8504880
C    -3.5116791    0.4260152   -1.4399616
O    -2.0577388    0.3593497   -1.5719469
C    -1.7051994   -0.2831525   -2.8440488
C    -0.4864837   -2.1061931    4.0848253
C     0.5053626   -0.9876213    4.4431908
C     0.7814644   -0.3523131    3.0870317
O    -0.4598592   -0.5025078    2.3341095
C    -1.3467699   -1.4507848    3.0119205
C     2.5439410   -3.0414846   -0.0011939
C     3.1232370   -1.6462235   -0.2844746
C     2.1122987   -1.0887316   -1.2748454
O     0.8229381   -1.5804149   -0.8092828
C     1.0313988   -2.7973773   -0.0295579
C     1.8444361    4.0571043   -0.2013689
C     0.6738179    4.2333835   -1.1813394
C     0.3767095    2.7966585   -1.5969574
O     0.7483180    1.9678982   -0.4496443
C     1.4506886    2.7825578    0.5342965
H    -3.0096063   -0.7814342   -4.5276130
H    -3.2433314   -1.7950640   -3.0879054
H    -5.0680058   -0.1690984   -2.8556567
H    -4.0290886    1.1625147   -3.4110931
H    -3.8429149   -0.3719474   -0.7573675
H    -3.7541776    1.4016857   -1.0018459
H    -0.9819004   -1.0788713   -2.6296174
H    -1.2287915    0.4817279   -3.4748693
H    -1.0794233   -2.4420051    4.9428811
H     0.0455008   -2.9763919    3.6759987
H     1.4185568   -1.3628765    4.9186671
H     0.0391884   -0.2605661    5.1214224
H     1.5837838   -0.8742428    2.5416606
H     1.0135375    0.7187421    3.1318685
H    -1.7295928   -2.1500250    2.2585202
H    -2.1849292   -0.8807517    3.4383508
H     2.8779767   -3.4612116    0.9547238
H     2.8375751   -3.7407770   -0.7949102
H     4.1413037   -1.6801336   -0.6892330
H     3.1377561   -1.0335857    0.6289360
H     2.2926686   -1.4693784   -2.2932486
H     2.0516797    0.0056624   -1.2975759
H     0.4700323   -3.6135831   -0.5032527
H     0.6193420   -2.6200910    0.9750340
H     1.9686731    4.9088396    0.4770941
H     2.7872301    3.9121291   -0.7462753
H     0.9242440    4.8698488   -2.0375773
H    -0.1922182    4.6747300   -0.6693275
H     0.9928733    2.4792107   -2.4521013
H    -0.6790257    2.6031653   -1.8218543
H     2.3028412    2.1958739    0.9010578
H     0.7671899    2.9922385    1.3726701

SCF energy GEOOPT =  -1077.248545952 H
ZPE =   1203. kJ/mol
FREEH energy =  1276.92 kJ/mol
FREEH entropy =   0.89394 kJ/mol/K

$vibrational spectrum
#  mode     symmetry     wave number   IR intensity    selection rules
```

#		cm**(-1)	km/mol	IR	RAMAN
1		-0.00	0.00000	-	-
2		-0.00	0.00000	-	-
3		0.00	0.00000	-	-
4		0.00	0.00000	-	-
5		0.00	0.00000	-	-
6		0.00	0.00000	-	-
7	a	4.17	0.11599	YES	YES
8	a	10.40	1.04734	YES	YES
9	a	15.67	0.17386	YES	YES
10	a	16.14	0.42028	YES	YES
11	a	20.48	0.13121	YES	YES
12	a	22.60	0.11864	YES	YES
13	a	27.37	0.03115	YES	YES
14	a	33.52	0.07873	YES	YES
15	a	36.74	0.37179	YES	YES
16	a	41.92	0.11289	YES	YES
17	a	51.21	0.48726	YES	YES
18	a	57.24	0.13816	YES	YES
19	a	60.91	1.00057	YES	YES
20	a	82.37	0.17864	YES	YES
21	a	83.86	3.75883	YES	YES
22	a	93.43	0.38757	YES	YES
23	a	95.95	1.38380	YES	YES
24	a	104.61	1.54997	YES	YES
25	a	110.15	4.15911	YES	YES
26	a	116.54	3.43301	YES	YES
27	a	119.83	2.03316	YES	YES
28	a	146.81	7.13582	YES	YES
29	a	147.97	6.63697	YES	YES
30	a	172.32	5.21297	YES	YES
31	a	192.40	14.10231	YES	YES
32	a	243.68	0.01264	YES	YES
33	a	247.71	0.09409	YES	YES
34	a	250.90	0.18672	YES	YES
35	a	268.71	3.86022	YES	YES
36	a	557.66	0.97740	YES	YES
37	a	558.64	0.44581	YES	YES
38	a	558.82	1.63976	YES	YES
39	a	562.94	1.42484	YES	YES
40	a	658.98	6.94681	YES	YES
41	a	662.11	7.89708	YES	YES
42	a	665.66	2.50147	YES	YES
43	a	669.73	4.45083	YES	YES
44	a	811.70	16.12260	YES	YES
45	a	821.26	47.36268	YES	YES
46	a	824.60	20.15799	YES	YES
47	a	827.62	17.75220	YES	YES
48	a	839.64	15.08826	YES	YES
49	a	842.18	24.72304	YES	YES
50	a	844.23	18.47984	YES	YES
51	a	844.81	32.43287	YES	YES
52	a	847.53	84.83182	YES	YES
53	a	849.84	88.12971	YES	YES
54	a	855.68	29.93833	YES	YES
55	a	864.21	28.44226	YES	YES
56	a	895.85	1.33618	YES	YES
57	a	896.17	4.63077	YES	YES
58	a	896.82	4.17545	YES	YES
59	a	897.36	6.70430	YES	YES
60	a	910.11	3.80943	YES	YES
61	a	910.79	7.64582	YES	YES
62	a	910.91	3.56121	YES	YES
63	a	911.19	2.41363	YES	YES
64	a	946.80	2.01930	YES	YES
65	a	947.15	1.53991	YES	YES
66	a	948.78	3.09220	YES	YES
67	a	949.57	1.12480	YES	YES
68	a	1002.33	45.08887	YES	YES
69	a	1006.70	26.20133	YES	YES
70	a	1009.02	107.56783	YES	YES
71	a	1009.40	85.40951	YES	YES
72	a	1020.31	6.05597	YES	YES
73	a	1021.16	6.27531	YES	YES

74	a	1022.65	7.53189	YES	YES
75	a	1023.70	2.30558	YES	YES
76	a	1128.45	0.35782	YES	YES
77	a	1128.72	1.54743	YES	YES
78	a	1129.01	2.92434	YES	YES
79	a	1130.54	1.60034	YES	YES
80	a	1143.08	3.03171	YES	YES
81	a	1145.45	4.08149	YES	YES
82	a	1147.66	2.26927	YES	YES
83	a	1148.51	1.94556	YES	YES
84	a	1150.56	4.59691	YES	YES
85	a	1151.91	7.24795	YES	YES
86	a	1152.43	2.40759	YES	YES
87	a	1166.39	2.59993	YES	YES
88	a	1214.31	0.73456	YES	YES
89	a	1216.81	0.52371	YES	YES
90	a	1219.69	1.03741	YES	YES
91	a	1220.14	0.96924	YES	YES
92	a	1229.98	8.69263	YES	YES
93	a	1230.79	3.76594	YES	YES
94	a	1232.59	5.36748	YES	YES
95	a	1236.14	3.85457	YES	YES
96	a	1280.48	0.72650	YES	YES
97	a	1280.52	0.80679	YES	YES
98	a	1281.39	0.56075	YES	YES
99	a	1281.50	0.88620	YES	YES
100	a	1301.57	1.21270	YES	YES
101	a	1305.15	0.45549	YES	YES
102	a	1305.37	0.18053	YES	YES
103	a	1306.04	0.32780	YES	YES
104	a	1321.47	0.69307	YES	YES
105	a	1323.94	0.60415	YES	YES
106	a	1325.61	1.92545	YES	YES
107	a	1327.73	2.91219	YES	YES
108	a	1344.80	5.42773	YES	YES
109	a	1346.26	2.01761	YES	YES
110	a	1347.71	2.21053	YES	YES
111	a	1348.78	5.65451	YES	YES
112	a	1439.71	4.59406	YES	YES
113	a	1440.62	6.55706	YES	YES
114	a	1440.83	7.05936	YES	YES
115	a	1442.00	6.92771	YES	YES
116	a	1447.84	10.22822	YES	YES
117	a	1447.99	8.92171	YES	YES
118	a	1449.54	9.31955	YES	YES
119	a	1450.11	8.47320	YES	YES
120	a	1466.11	0.67299	YES	YES
121	a	1470.90	0.73658	YES	YES
122	a	1471.79	0.36535	YES	YES
123	a	1473.21	0.47722	YES	YES
124	a	1479.01	1.46290	YES	YES
125	a	1483.23	2.35687	YES	YES
126	a	1483.77	1.11420	YES	YES
127	a	1484.40	0.88518	YES	YES
128	a	2946.77	48.18980	YES	YES
129	a	2947.83	37.17828	YES	YES
130	a	2950.89	29.05296	YES	YES
131	a	2956.35	36.31359	YES	YES
132	a	2959.78	22.86838	YES	YES
133	a	2964.53	35.11715	YES	YES
134	a	2968.77	26.12193	YES	YES
135	a	2972.86	25.91468	YES	YES
136	a	2976.84	26.17035	YES	YES
137	a	2987.99	15.70067	YES	YES
138	a	2988.07	3.35213	YES	YES
139	a	2989.21	7.02795	YES	YES
140	a	2994.91	11.80288	YES	YES
141	a	2996.20	13.14826	YES	YES
142	a	2996.35	11.65730	YES	YES
143	a	2998.12	10.96980	YES	YES
144	a	3013.99	28.01741	YES	YES
145	a	3018.78	26.53087	YES	YES
146	a	3028.62	26.88265	YES	YES
147	a	3030.73	6.36703	YES	YES

```
   148        a            3030.92         11.98446        YES      YES
   149        a            3033.27         22.26958        YES      YES
   150        a            3035.45          2.54797        YES      YES
   151        a            3038.31          3.47950        YES      YES
   152        a            3046.40          7.25541        YES      YES
   153        a            3050.44          6.77254        YES      YES
   154        a            3051.55          4.95631        YES      YES
   155        a            3052.93          5.59348        YES      YES
   156        a            3054.15         26.24953        YES      YES
   157        a            3057.94         17.82124        YES      YES
   158        a            3059.11         19.01218        YES      YES
   159        a            3060.33         18.64454        YES      YES
$end
```

10.1.5 Further structures

NO^+

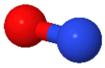

```
Method: (RI-)BP86(D3BJ)/def2-TZVPP
Symmetry: c6v

Cartesian coordinates in Ångström:
O          0.00000        0.00000       -0.53483
N          0.00000        0.00000        0.53483

SCF energy GEOOPT =  -129.6086651806 H
ZPE =   14.11 kJ/mol
FREEH energy =    20.31 kJ/mol
FREEH entropy =   0.19845 kJ/mol

$vibrational spectrum
#  mode     symmetry     wave number   IR intensity    selection rules
#                         cm**(-1)        km/mol          IR      RAMAN
     1                       -0.00         0.00000         -        -
     2                        0.00         0.00000         -        -
     3                        0.00         0.00000         -        -
     4                        0.00         0.00000         -        -
     5                        0.00         0.00000         -        -
     6        a1            2359.25        23.35239        YES      YES
$end
```

Ag^+

```
Method: (RI-)BP86(D3BJ)/def2-TZVPP
Symmetry: oh

Cartesian coordinates in Ångström:
Ag        -1.23058        0.18943        0.00000

SCF energy GEOOPT =  -146.7963177200 H
```

[*pf*]$^-$

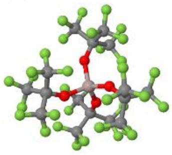

```
Method: (RI-)BP86(D3BJ)/def2-TZVPP
Symmetry: c1

Cartesian coordinates in Ångström:
```

```
Al   -0.0000099    0.0000134   -0.0298653
O     1.4385460    0.2794156    0.9244492
O     0.2850095   -1.4380580   -0.9867711
O    -1.4385757   -0.2794089    0.9244281
O    -0.2850155    1.4380960   -0.9867596
C     2.4273372   -0.2263858    1.6881238
C     0.2492758    2.4482442   -1.7029653
C    -2.4273545    0.2263764    1.6881279
C    -0.2492670   -2.4482275   -1.7029574
C     2.1317974   -1.7095490    2.1545515
F     2.8765205   -2.0805176    3.2289792
F     2.3862950   -2.5916362    1.1635630
F     0.8345583   -1.8389147    2.4915389
C     3.7843854   -0.2136788    0.8745246
F     4.7522427   -0.9702263    1.4582546
F     4.2704442    1.0449293    0.7636177
F     3.5903569   -0.6838540   -0.3697097
C     2.5829879    0.6891468    2.9696875
F     1.5942830    0.4371177    3.8591210
F     2.5035554    1.9892151    2.6349800
F     3.7667341    0.5017840    3.6118967
C    -0.9433574    3.2652915   -2.3453300
F    -1.5549973    4.0352811   -1.4151667
F    -1.8696613    2.4322675   -2.8519210
F    -0.5354149    4.0871160   -3.3488613
C     1.0995488    3.4193653   -0.7839074
F     1.2838822    4.6437308   -1.3487865
F     2.3229222    2.9031206   -0.5358342
F     0.4858556    3.6042885    0.3976744
C     1.1782370    1.9029562   -2.8602533
F     1.9639602    0.9127039   -2.3961678
F     1.9849736    2.8608390   -3.3862929
F     0.4406454    1.3955053   -3.8742357
C    -2.5829906   -0.6891711    2.9696822
F    -3.7667412   -0.5018292    3.6118908
F    -1.5942917   -0.4371391    3.8591202
F    -2.5035420   -1.9892346    2.6349593
C    -2.1318052    1.7095339    2.1545677
F    -2.3863144    2.5916310    1.1635916
F    -0.8345591    1.8388915    2.4915360
F    -2.8765085    2.0804933    3.2290125
C    -3.7844163    0.2136815    0.8745492
F    -3.5903960    0.6838404   -0.3696929
F    -4.7522509    0.9702552    1.4582848
F    -4.2705014   -1.0449174    0.7636633
C    -1.0995262   -3.4193501   -0.7838862
F    -1.2838114   -4.6437365   -1.3487357
F    -0.4858469   -3.6042220    0.3977097
F    -2.3229223   -2.9031404   -0.5358465
C     0.9433805   -3.2652653   -2.3453087
F     1.8696664   -2.4322339   -2.8519203
F     0.5354526   -4.0871202   -3.3488211
F     1.5550383   -4.0352215   -1.4151289
C    -1.1782353   -1.9029710   -2.8602533
F    -1.9639913   -0.9127397   -2.3961790
F    -0.4406493   -1.3955022   -3.8742309
F    -1.9849388   -2.8608798   -3.3862969

SCF energy GEOOPT =  -4750.100650386 H
ZPE =   556.0 kJ/mol
FREEH energy =   704.28 kJ/mol
FREEH entropy =   1.36743 kJ/mol/K

$vibrational spectrum
#  mode     symmetry     wave number   IR intensity    selection rules
#                         cm**(-1)        km/mol          IR     RAMAN
     1                      -0.00         0.00000         -        -
     2                       0.00         0.00000         -        -
     3                       0.00         0.00000         -        -
     4                       0.00         0.00000         -        -
     5                       0.00         0.00000         -        -
     6                       0.00         0.00000         -        -
     7        a             10.62         0.00071        YES      YES
     8        a             14.15         0.00617        YES      YES
```

9	a	15.06	0.00286	YES	YES
10	a	17.98	0.00053	YES	YES
11	a	28.48	0.07895	YES	YES
12	a	29.90	0.00031	YES	YES
13	a	32.12	0.00173	YES	YES
14	a	33.99	0.03724	YES	YES
15	a	38.43	0.09082	YES	YES
16	a	39.34	0.02229	YES	YES
17	a	43.01	0.07278	YES	YES
18	a	55.43	0.01698	YES	YES
19	a	58.59	0.00128	YES	YES
20	a	66.60	0.13030	YES	YES
21	a	66.88	0.21598	YES	YES
22	a	70.10	0.12177	YES	YES
23	a	70.88	0.07210	YES	YES
24	a	74.47	0.04756	YES	YES
25	a	75.08	0.00229	YES	YES
26	a	76.58	0.10572	YES	YES
27	a	78.54	0.00557	YES	YES
28	a	81.33	0.03852	YES	YES
29	a	81.45	0.06939	YES	YES
30	a	86.01	0.14832	YES	YES
31	a	86.84	0.00320	YES	YES
32	a	88.08	0.03500	YES	YES
33	a	92.36	0.62613	YES	YES
34	a	95.10	0.75889	YES	YES
35	a	95.86	1.02369	YES	YES
36	a	109.00	0.00727	YES	YES
37	a	153.99	0.17505	YES	YES
38	a	155.93	0.11804	YES	YES
39	a	158.68	0.24188	YES	YES
40	a	160.78	0.03929	YES	YES
41	a	161.17	0.66396	YES	YES
42	a	162.08	0.55035	YES	YES
43	a	162.97	0.19053	YES	YES
44	a	163.49	0.48380	YES	YES
45	a	191.92	3.39380	YES	YES
46	a	192.85	2.78075	YES	YES
47	a	193.50	2.56099	YES	YES
48	a	215.70	0.00053	YES	YES
49	a	257.11	1.03306	YES	YES
50	a	258.18	0.27662	YES	YES
51	a	265.60	2.77310	YES	YES
52	a	266.87	0.35643	YES	YES
53	a	270.55	2.95429	YES	YES
54	a	272.07	2.49277	YES	YES
55	a	274.04	2.44156	YES	YES
56	a	275.81	0.70550	YES	YES
57	a	278.26	0.03571	YES	YES
58	a	278.43	0.06606	YES	YES
59	a	278.91	0.03040	YES	YES
60	a	279.25	0.00488	YES	YES
61	a	296.13	7.33787	YES	YES
62	a	296.68	8.78883	YES	YES
63	a	297.17	7.10032	YES	YES
64	a	301.98	0.01991	YES	YES
65	a	305.55	0.28157	YES	YES
66	a	306.38	0.00186	YES	YES
67	a	310.49	0.19339	YES	YES
68	a	311.79	0.00239	YES	YES
69	a	313.48	1.43770	YES	YES
70	a	314.86	2.32437	YES	YES
71	a	315.80	1.92254	YES	YES
72	a	318.49	0.01137	YES	YES
73	a	336.29	0.72952	YES	YES
74	a	336.89	0.76988	YES	YES
75	a	342.40	2.24474	YES	YES
76	a	348.90	9.72146	YES	YES
77	a	349.81	0.41281	YES	YES
78	a	351.02	10.97371	YES	YES
79	a	365.30	31.35153	YES	YES
80	a	375.72	0.07896	YES	YES
81	a	424.09	51.71860	YES	YES
82	a	435.11	65.26626	YES	YES

83	a	435.49	57.35910	YES	YES
84	a	504.08	0.25487	YES	YES
85	a	508.12	1.63530	YES	YES
86	a	508.48	4.33708	YES	YES
87	a	508.77	1.52403	YES	YES
88	a	508.78	7.82921	YES	YES
89	a	509.53	2.63823	YES	YES
90	a	509.89	3.55695	YES	YES
91	a	509.96	7.37903	YES	YES
92	a	510.49	0.21622	YES	YES
93	a	514.58	2.45589	YES	YES
94	a	515.13	1.69764	YES	YES
95	a	515.33	1.95516	YES	YES
96	a	518.42	0.00839	YES	YES
97	a	533.56	21.97102	YES	YES
98	a	538.30	19.36850	YES	YES
99	a	539.34	22.16017	YES	YES
100	a	543.04	0.12077	YES	YES
101	a	543.11	0.15723	YES	YES
102	a	543.55	0.54975	YES	YES
103	a	544.02	0.02995	YES	YES
104	a	544.78	0.11445	YES	YES
105	a	545.65	2.69456	YES	YES
106	a	546.48	16.06728	YES	YES
107	a	547.24	11.35348	YES	YES
108	a	694.53	1.23928	YES	YES
109	a	694.80	1.91996	YES	YES
110	a	695.08	1.59971	YES	YES
111	a	695.46	3.05241	YES	YES
112	a	696.28	6.50338	YES	YES
113	a	696.96	75.33289	YES	YES
114	a	696.98	70.78605	YES	YES
115	a	697.39	72.23096	YES	YES
116	a	711.96	0.08940	YES	YES
117	a	722.07	2.64168	YES	YES
118	a	723.26	4.14581	YES	YES
119	a	723.46	4.79792	YES	YES
120	a	761.21	0.03286	YES	YES
121	a	797.34	8.31913	YES	YES
122	a	806.67	16.84831	YES	YES
123	a	807.72	16.23841	YES	YES
124	a	924.18	6.13230	YES	YES
125	a	925.42	21.57375	YES	YES
126	a	928.33	2.45634	YES	YES
127	a	929.25	1.10425	YES	YES
128	a	932.53	310.97235	YES	YES
129	a	933.34	23.87214	YES	YES
130	a	934.88	330.44420	YES	YES
131	a	936.07	325.66778	YES	YES
132	a	1055.42	11.85969	YES	YES
133	a	1055.60	11.37053	YES	YES
134	a	1057.67	8.69317	YES	YES
135	a	1064.11	0.52526	YES	YES
136	a	1073.36	15.36632	YES	YES
137	a	1075.38	11.95805	YES	YES
138	a	1076.04	1.34796	YES	YES
139	a	1077.74	15.70788	YES	YES
140	a	1079.99	6.65775	YES	YES
141	a	1083.13	16.12202	YES	YES
142	a	1084.43	18.19378	YES	YES
143	a	1084.75	11.80639	YES	YES
144	a	1140.83	29.72160	YES	YES
145	a	1142.49	12.82479	YES	YES
146	a	1143.51	48.39130	YES	YES
147	a	1149.32	0.00601	YES	YES
148	a	1150.96	2.24779	YES	YES
149	a	1155.73	0.96732	YES	YES
150	a	1157.22	29.23066	YES	YES
151	a	1159.00	81.90585	YES	YES
152	a	1168.03	1319.54216	YES	YES
153	a	1168.79	1117.40088	YES	YES
154	a	1168.82	1351.56988	YES	YES
155	a	1182.39	0.17983	YES	YES
156	a	1184.50	105.11608	YES	YES

157	a	1184.84	55.44855	YES	YES
158	a	1186.98	116.27873	YES	YES
159	a	1188.70	80.44084	YES	YES
160	a	1190.73	35.98292	YES	YES
161	a	1191.21	120.70187	YES	YES
162	a	1200.82	75.46599	YES	YES
163	a	1202.93	1.24469	YES	YES
164	a	1205.01	920.08852	YES	YES
165	a	1206.68	978.47597	YES	YES
166	a	1209.45	896.35219	YES	YES
167	a	1217.40	17.82459	YES	YES
168	a	1297.39	289.91713	YES	YES
169	a	1297.84	258.14533	YES	YES
170	a	1299.99	289.42702	YES	YES
171	a	1325.82	2.62259	YES	YES

$end

{Ag[*pf*]}*ip.*

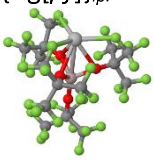

Method: (RI-)BP86(D3BJ)/def2-TZVPP
Symmetry: c1

Cartesian coordinates in Ångström:

Ag	1.1773493	9.2855463	5.8506443
Al	1.2860909	7.3945908	3.7223766
O	1.3603651	6.2261290	2.5050038
C	1.4023515	5.3581842	1.4640849
C	0.4462954	5.8555747	0.3063968
F	-0.8436201	5.5795995	0.5967318
F	0.7379959	5.2784841	-0.8779771
F	0.5515757	7.1915251	0.1636001
C	2.8812392	5.2599208	0.9116783
F	3.7572929	5.2376437	1.9340495
F	3.1774706	6.3340589	0.1471603
F	3.0729755	4.1523038	0.1648457
C	0.9308249	3.9295671	1.9520820
F	1.9111993	3.3140652	2.6484432
F	0.5872246	3.1290849	0.9208624
F	-0.1333973	4.0474297	2.7690634
O	-0.2760126	8.1523786	4.1026514
C	-1.5955237	7.9997191	4.4225893
C	-2.1629819	9.4258046	4.8019842
F	-2.2746650	10.2019126	3.7140331
F	-3.3501232	9.3717276	5.4235015
F	-1.3006535	10.0742062	5.6578219
C	-1.7594622	7.0199049	5.6463740
F	-1.0538881	5.8955926	5.4418523
F	-1.2654346	7.6074604	6.7744970
F	-3.0395203	6.6848123	5.8834391
C	-2.4095237	7.4465695	3.1845530
F	-2.2239429	6.1184691	3.0552469
F	-3.7355465	7.6730384	3.3123099
F	-1.9850254	8.0396423	2.0561164
O	2.2817325	8.8601320	3.5943043
C	2.6529622	9.9648561	2.8825584
C	3.5002131	9.5520604	1.6118900
F	2.6899300	9.1225826	0.6250263
F	4.2280324	10.5857562	1.1346008
F	4.3404295	8.5522800	1.9276607
C	3.5591852	10.8577094	3.8216870
F	2.9850639	10.9834401	5.0678331
F	4.7584773	10.2885517	4.0116533
F	3.7351230	12.1024943	3.3552565
C	1.3848004	10.7853800	2.4320785
F	0.7908514	11.3485200	3.5230906
F	1.6778523	11.7720249	1.5670070
F	0.4807670	9.9715600	1.8622567
O	1.7524191	6.8968324	5.3676740

```
C     2.6799595    6.2889476    6.1674017
C     4.0979825    6.9467986    5.9663010
F     4.0911977    8.2152511    6.4657315
F     5.0831211    6.2668528    6.5786090
F     4.3890435    7.0357169    4.6576014
C     2.7607289    4.7406786    5.8530092
F     1.5279028    4.2527548    5.6352379
F     3.4983186    4.5223790    4.7472837
F     3.3191547    4.0496901    6.8712060
C     2.2125052    6.4758370    7.6656034
F     1.1422991    5.7121500    7.9316891
F     3.1771226    6.2013163    8.5557878
F     1.8288643    7.7787311    7.8903950

SCF energy GEOOPT =  -4897.092911513 H
ZPE =   559.7 kJ/mol
FREEH energy =   713.16 kJ/mol
FREEH entropy =   1.40457 kJ/mol/K

$vibrational spectrum
#  mode     symmetry     wave number   IR intensity    selection rules
#                         cm**(-1)        km/mol         IR     RAMAN
     1                       -0.00         0.00000        -       -
     2                       -0.00         0.00000        -       -
     3                       -0.00         0.00000        -       -
     4                       -0.00         0.00000        -       -
     5                       -0.00         0.00000        -       -
     6                        0.00         0.00000        -       -
     7         a             12.14         0.10878       YES     YES
     8         a             13.98         0.03601       YES     YES
     9         a             18.53         0.03414       YES     YES
    10         a             20.89         0.07169       YES     YES
    11         a             21.32         0.10782       YES     YES
    12         a             31.97         0.05632       YES     YES
    13         a             32.50         0.08893       YES     YES
    14         a             35.51         0.00369       YES     YES
    15         a             42.28         0.07572       YES     YES
    16         a             42.46         0.02298       YES     YES
    17         a             46.01         0.00655       YES     YES
    18         a             59.38         0.06517       YES     YES
    19         a             62.45         1.82161       YES     YES
    20         a             63.28         1.52870       YES     YES
    21         a             65.46         0.11389       YES     YES
    22         a             66.33         0.97349       YES     YES
    23         a             67.07         0.90286       YES     YES
    24         a             68.21         0.87412       YES     YES
    25         a             68.72         1.17343       YES     YES
    26         a             74.23         0.89540       YES     YES
    27         a             75.51         3.30706       YES     YES
    28         a             76.06         2.45395       YES     YES
    29         a             81.32         0.43435       YES     YES
    30         a             82.46         0.56470       YES     YES
    31         a             83.84         0.08456       YES     YES
    32         a             87.94         0.00213       YES     YES
    33         a             92.26         0.31252       YES     YES
    34         a             92.43         0.34597       YES     YES
    35         a             95.75         0.00974       YES     YES
    36         a            106.99         0.34438       YES     YES
    37         a            107.36         0.30237       YES     YES
    38         a            110.41         5.12944       YES     YES
    39         a            120.87         0.03744       YES     YES
    40         a            156.01         0.04103       YES     YES
    41         a            156.59         0.04094       YES     YES
    42         a            162.21         0.13174       YES     YES
    43         a            164.60         0.67293       YES     YES
    44         a            164.99         0.68691       YES     YES
    45         a            169.06         0.30859       YES     YES
    46         a            169.82         0.54756       YES     YES
    47         a            169.84         0.26774       YES     YES
    48         a            193.16         5.58283       YES     YES
    49         a            193.37         5.65420       YES     YES
    50         a            200.76         3.79650       YES     YES
    51         a            222.50         0.14698       YES     YES
    52         a            259.73         1.01385       YES     YES
```

53	a	260.09	1.10656	YES	YES
54	a	271.85	1.68775	YES	YES
55	a	272.07	1.69957	YES	YES
56	a	274.73	1.31148	YES	YES
57	a	278.72	1.95736	YES	YES
58	a	279.25	0.54378	YES	YES
59	a	279.49	1.64513	YES	YES
60	a	280.75	0.39203	YES	YES
61	a	280.82	0.15959	YES	YES
62	a	280.88	0.20343	YES	YES
63	a	281.19	2.51220	YES	YES
64	a	300.54	3.29443	YES	YES
65	a	302.64	8.98521	YES	YES
66	a	302.79	8.87004	YES	YES
67	a	306.03	0.04141	YES	YES
68	a	307.67	0.17547	YES	YES
69	a	307.91	0.13449	YES	YES
70	a	314.59	0.91747	YES	YES
71	a	314.97	1.24261	YES	YES
72	a	316.64	2.27341	YES	YES
73	a	316.88	2.39643	YES	YES
74	a	317.33	1.95373	YES	YES
75	a	318.57	0.20373	YES	YES
76	a	338.66	0.24899	YES	YES
77	a	338.76	0.25415	YES	YES
78	a	347.59	12.96862	YES	YES
79	a	347.82	13.21074	YES	YES
80	a	355.57	1.86094	YES	YES
81	a	367.00	15.64156	YES	YES
82	a	370.13	17.14185	YES	YES
83	a	373.86	2.76979	YES	YES
84	a	416.21	51.34445	YES	YES
85	a	417.07	47.07151	YES	YES
86	a	442.82	74.22097	YES	YES
87	a	504.71	0.14925	YES	YES
88	a	507.07	1.18884	YES	YES
89	a	507.63	2.95168	YES	YES
90	a	507.86	2.62823	YES	YES
91	a	510.95	2.13742	YES	YES
92	a	511.16	1.72030	YES	YES
93	a	512.32	1.93454	YES	YES
94	a	512.48	2.37617	YES	YES
95	a	512.65	4.14493	YES	YES
96	a	515.72	8.47181	YES	YES
97	a	515.82	8.35979	YES	YES
98	a	517.62	2.05349	YES	YES
99	a	518.97	0.00466	YES	YES
100	a	523.37	7.73545	YES	YES
101	a	523.56	8.20228	YES	YES
102	a	542.51	4.02121	YES	YES
103	a	544.07	0.43240	YES	YES
104	a	544.15	0.40882	YES	YES
105	a	544.38	0.17531	YES	YES
106	a	545.40	0.67063	YES	YES
107	a	545.67	0.68703	YES	YES
108	a	546.42	0.02774	YES	YES
109	a	546.63	0.02380	YES	YES
110	a	551.74	26.88384	YES	YES
111	a	694.35	1.76169	YES	YES
112	a	695.73	27.33111	YES	YES
113	a	695.77	27.47282	YES	YES
114	a	697.99	28.84745	YES	YES
115	a	698.01	29.68444	YES	YES
116	a	700.08	18.59809	YES	YES
117	a	700.16	27.40058	YES	YES
118	a	700.21	60.45583	YES	YES
119	a	713.22	23.76186	YES	YES
120	a	717.21	1.58607	YES	YES
121	a	717.47	1.69431	YES	YES
122	a	728.83	1.06141	YES	YES
123	a	771.85	2.24456	YES	YES
124	a	779.27	14.12547	YES	YES
125	a	781.23	13.88529	YES	YES
126	a	872.63	1.66083	YES	YES

127	a	915.86	9.82193	YES	YES
128	a	917.20	7.76991	YES	YES
129	a	926.39	318.72241	YES	YES
130	a	932.50	74.64961	YES	YES
131	a	932.58	47.64320	YES	YES
132	a	932.86	36.63227	YES	YES
133	a	938.26	236.72718	YES	YES
134	a	938.54	239.67948	YES	YES
135	a	1034.46	9.90847	YES	YES
136	a	1035.01	16.83688	YES	YES
137	a	1036.35	13.97359	YES	YES
138	a	1063.68	0.06194	YES	YES
139	a	1089.27	2.02535	YES	YES
140	a	1090.06	1.58056	YES	YES
141	a	1102.11	76.93012	YES	YES
142	a	1106.36	9.76947	YES	YES
143	a	1107.25	8.84860	YES	YES
144	a	1121.89	4.63705	YES	YES
145	a	1124.41	13.59255	YES	YES
146	a	1124.69	14.71823	YES	YES
147	a	1134.10	55.20133	YES	YES
148	a	1134.43	60.63854	YES	YES
149	a	1135.41	14.48375	YES	YES
150	a	1146.39	28.96052	YES	YES
151	a	1147.16	26.22590	YES	YES
152	a	1150.71	8.48292	YES	YES
153	a	1157.02	5.80770	YES	YES
154	a	1157.18	2.10241	YES	YES
155	a	1165.41	4.67157	YES	YES
156	a	1165.88	4.25592	YES	YES
157	a	1170.88	258.89161	YES	YES
158	a	1172.00	365.47565	YES	YES
159	a	1172.41	375.92904	YES	YES
160	a	1178.16	240.94895	YES	YES
161	a	1196.12	1105.09056	YES	YES
162	a	1196.58	1077.68065	YES	YES
163	a	1199.61	438.79299	YES	YES
164	a	1205.07	516.25948	YES	YES
165	a	1207.11	585.73864	YES	YES
166	a	1207.54	575.32553	YES	YES
167	a	1209.50	765.08380	YES	YES
168	a	1221.32	556.89800	YES	YES
169	a	1221.54	542.30409	YES	YES
170	a	1229.15	4.48533	YES	YES
171	a	1271.01	24.17309	YES	YES
172	a	1272.92	33.52681	YES	YES
173	a	1282.18	48.00228	YES	YES
174	a	1336.81	338.12497	YES	YES

$end

10.2 EDA-NOCV results

Supplementary Table 40: EDA-NOCV results for $[(xFB)NO]^+$ complexes using NO^+ and xFB as interacting fragments at the BP86(D3BJ)/TZ2P level given in kJ mol^{-1}.

Energies	mesitylene	benzene	1FB	2FB	3FB	4FB	5FB	6FB
ΔE_{int}	−304.3	−239.7	−230.5	−222.1	−212.2	−204.6	−196.2	−188.1
ΔE_{Pauli}	205.9	158.5	156.5	152.9	149.4	144.5	140.9	138.7
ΔE_{disp}	−20.2	−16.2	−16.1	−16.1	−16.1	−16.2	−16.5	−16.5
ΔE_{elstat}	−112.2	−85.9	−72.6	−59.3	−45.6	−30.3	−11.9	1.0
ΔE_{orb}	−377.8	−296.1	−298.2	−299.7	−299.8	−302.6	−308.8	−311.3
$\Delta E_{orb(1)}$[a]	−179.5	−139.9	−158.9	−156.5	−139.6	−149.5	−166.5	−149.0
$\Delta E_{orb(2)}$[a]	−156.1	−127.7	−111.3	−115.1	−132.0	−124.6	−112.5	−132.0
$\Delta E_{orb(rest)}$	−42.0	−28.5	−28.2	−28.1	−28.2	−28.6	−29.7	−30.1

[a] $\Delta E_{orb(1-2)}$ contributions are π-donations from the arenes towards the π*-orbital of the NO^+ cation.

11 Supplementary References

1. Harris, R. K., Becker, E. D., Cabral de Menezes, S. M., Goodfellow, R. & Granger, P. NMR nomenclature. Nuclear spin properties and conventions for chemical shifts(IUPAC Recommendations 2001). *Pure Appl. Chem.* **73,** 1795–1818; 10.1351/pac200173111795 (2001).

2. Arnim, M. von & Ahlrichs, R. Performance of parallel TURBOMOLE for density functional calculations. *J. Comput. Chem.* **19,** 1746–1757; 10.1002/(SICI)1096-987X(19981130)19:15<1746::AID-JCC7>3.0.CO;2-N (1998).

3. Treutler, O. & Ahlrichs, R. Efficient molecular numerical integration schemes. *J. Chem. Phys.* **102,** 346–354; 10.1063/1.469408 (1995).

4. Perdew, J. P. Density-functional approximation for the correlation energy of the inhomogeneous electron gas. *Phys. Rev. B: Condens. Matter* **33,** 8822–8824; 10.1103/physrevb.33.8822 (1986).

5. Perdew, J. P. Erratum: Density-functional approximation for the correlation energy of the inhomogeneous electron gas. *Phys. Rev. B: Condens. Matter* **34,** 7406; 10.1103/PhysRevB.34.7406 (1986).

6. Becke, A. D. A new mixing of Hartree–Fock and local density-functional theories. *J. Chem. Phys.* **98,** 1372–1377; 10.1063/1.464304 (1993).

7. Becke, A. D. Density-functional thermochemistry. III. The role of exact exchange. *J. Chem. Phys.* **98,** 5648–5652; 10.1063/1.464913 (1993).

8. Lee, Yang & Parr. Development of the Colle-Salvetti correlation-energy formula into a functional of the electron density. *Phys. Rev. B: Condens. Matter* **37,** 785–789; 10.1103/physrevb.37.785 (1988).

9. Weigend, F. & Ahlrichs, R. Balanced basis sets of split valence, triple zeta valence and quadruple zeta valence quality for H to Rn: Design and assessment of accuracy. *Phys. Chem. Chem. Phys.* **7,** 3297–3305; 10.1039/B508541A (2005).

10. Ahlrichs, R. Efficient evaluation of three-center two-electron integrals over Gaussian functions. *Phys. Chem. Chem. Phys.* **6,** 5119; 10.1039/B413539C (2004).

11. Sierka, M., Hogekamp, A. & Ahlrichs, R. Fast evaluation of the Coulomb potential for electron densities using multipole accelerated resolution of identity approximation. *J. Chem. Phys.* **118,** 9136–9148; 10.1063/1.1567253 (2003).

12. Weigend, F. Accurate Coulomb-fitting basis sets for H to Rn. *Phys. Chem. Chem. Phys.* **8,** 1057–1065; 10.1039/B515623H (2006).

13. Grimme, S., Antony, J., Ehrlich, S. & Krieg, H. A consistent and accurate ab initio parametrization of density functional dispersion correction (DFT-D) for the 94 elements H-Pu. *J. Chem. Phys.* **132,** 154104; 10.1063/1.3382344 (2010).

14. Grimme, S., Ehrlich, S. & Goerigk, L. Effect of the damping function in dispersion corrected density functional theory. *J. Comput. Chem.* **32,** 1456–1465; 10.1002/jcc.21759 (2011).

15. Deglmann, P., Furche, F. & Ahlrichs, R. An efficient implementation of second analytical derivatives for density functional methods. *Chem. Phys. Lett.* **362,** 511–518; 10.1016/S0009-2614(02)01084-9 (2002).

16. Neese, F. The ORCA program system. *WIRES Comput. Mol. Sci.* **2,** 73–78; 10.1002/wcms.81 (2012).

17. Neese, F. Software update: the ORCA program system, version 4.0. *WIRES Comput. Mol. Sci.* **8**; 10.1002/wcms.1327 (2018).

18. Neese, F., Wennmohs, F., Becker, U. & Riplinger, C. The ORCA quantum chemistry program package. *J. Chem. Phys.* **152,** 224108; 10.1063/5.0004608 (2020).

19. Guo, Y. *et al.* An improved linear scaling perturbative triples correction for the domain based local pair-natural orbital based singles and doubles coupled cluster method DLPNO-CCSD(T). *J. Chem. Phys.* **148,** 11101; 10.1063/1.5011798 (2018).

20. Riplinger, C. & Neese, F. An efficient and near linear scaling pair natural orbital based local coupled cluster method. *J. Chem. Phys.* **138,** 34106; 10.1063/1.4773581 (2013).

21. Riplinger, C., Sandhoefer, B., Hansen, A. & Neese, F. Natural triple excitations in local coupled cluster calculations with pair natural orbitals. *J. Chem. Phys.* **139,** 134101; 10.1063/1.4821834 (2013).

22. Dunning, T. H. Gaussian basis sets for use in correlated molecular calculations. I. The atoms boron through neon and hydrogen. *J. Chem. Phys.* **90,** 1007–1023; 10.1063/1.456153 (1989).

23. Woon, D. E. & Dunning, T. H. Gaussian basis sets for use in correlated molecular calculations. III. The atoms aluminum through argon. *J. Chem. Phys.* **98,** 1358–1371; 10.1063/1.464303 (1993).

24. Wilson, A. K., Woon, D. E., Peterson, K. A. & Dunning, T. H. Gaussian basis sets for use in correlated molecular calculations. IX. The atoms gallium through krypton. *J. Chem. Phys.* **110,** 7667–7676; 10.1063/1.478678 (1999).

25. Weigend, F., Köhn, A. & Hättig, C. Efficient use of the correlation consistent basis sets in resolution of the identity MP2 calculations. *J. Chem. Phys.* **116,** 3175–3183; 10.1063/1.1445115 (2002).

26. Neese, F., Wennmohs, F., Hansen, A. & Becker, U. Efficient, approximate and parallel Hartree–Fock and hybrid DFT calculations. A 'chain-of-spheres' algorithm for the Hartree–Fock exchange. *Chem. Phys.* **356,** 98–109; 10.1016/j.chemphys.2008.10.036 (2009).

27. Izsák, R. & Neese, F. An overlap fitted chain of spheres exchange method. *J. Chem. Phys.* **135,** 144105; 10.1063/1.3646921 (2011).

28. Peterson, K. A. & Puzzarini, C. Systematically convergent basis sets for transition metals. II. Pseudopotential-based correlation consistent basis sets for the group 11 (Cu, Ag, Au) and 12 (Zn, Cd, Hg) elements. *Theor. Chem. Acc.* **114,** 283–296; 10.1007/s00214-005-0681-9 (2005).

29. Hättig, C. *unpublished results* **EMSL Basis Set Exchange (bse.pnl.gov)** (2017).

30. Figgen, D., Rauhut, G., Dolg, M. & Stoll, H. Energy-consistent pseudopotentials for group 11 and 12 atoms: adjustment to multi-configuration Dirac–Hartree–Fock data. *Chem. Phys.* **311,** 227–244; 10.1016/j.chemphys.2004.10.005 (2005).

31. Eckert, F. & Klamt, A. Fast solvent screening via quantum chemistry: COSMO-RS approach. *AIChE J.* **48,** 369–385; 10.1002/aic.690480220 (2002).

32. Klamt, A. Conductor-like Screening Model for Real Solvents: A New Approach to the Quantitative Calculation of Solvation Phenomena. *J. Phys. Chem.* **99,** 2224–2235; 10.1021/j100007a062 (1995).

33. Klamt, A., Jonas, V., Bürger, T. & Lohrenz, J. C. W. Refinement and Parametrization of COSMO-RS. *J. Phys. Chem. A* **102,** 5074–5085; 10.1021/jp980017s (1998).

34. Rappoport, D. & Furche, F. Property-optimized gaussian basis sets for molecular response calculations. *J. Chem. Phys.* **133,** 134105; 10.1063/1.3484283 (2010).

35. Eckert, F. & Klamt, A. *COSMOtherm Version C.30, Release 15.01* (COSMOlogic GmbH & Co. KG, Leverkusen, 2014).

36. Bruker. *SAINT, V8.40B* (Bruker AXS inc., Madison, Wisconsin, USA,).

37. Bruker. *SADABS, 2016/2* (Bruker AXS inc., Madison, Wisconsin, USA,).

38. Bruker. *TWINABS* (Bruker AXS inc., Madison, Wisconsin, USA,).

39. Krause, L., Herbst-Irmer, R., Sheldrick, G. M. & Stalke, D. Comparison of silver and molybdenum microfocus X-ray sources for single-crystal structure determination. *J. Appl. Crystallogr.* **48,** 3–10; 10.1107/S1600576714022985. (2015).

40. Sheldrick, G. M. SHELXT - integrated space-group and crystal-structure determination. *Acta Crystallogr. A* **71,** 3–8; 10.1107/S2053273314026370 (2015).

41. Sheldrick, G. M. Crystal structure refinement with SHELXL. *Acta Crystallogr. C* **71,** 3–8; 10.1107/S2053229614024218 (2015).

42. Hübschle, C. B., Sheldrick, G. M. & Dittrich, B. ShelXle: a Qt graphical user interface for SHELXL. *J. Appl. Crystallogr.* **44,** 1281–1284; 10.1107/S0021889811043202 (2011).

43. Kratzert, D., Holstein, J. J. & Krossing, I. DSR: enhanced modelling and refinement of disordered structures with SHELXL. *J. Appl. Crystallogr.* **48,** 933–938; 10.1107/S1600576715005580 (2015).

44. Kratzert, D. & Krossing, I. Recent improvements in DSR. *J. Appl. Crystallogr.* **51,** 928–934; 10.1107/S1600576718004508 (2018).

45. D. Kratzert. *FinalCif, V101-V103, https://dkratzert.de/finalcif.html ,*

46. Dolomanov, O. V., Bourhis, L. J., Gildea, R. J., Howard, J. A. K. & Puschmann, H. OLEX2 : a complete structure solution, refinement and analysis program. *J. Appl. Crystallogr.* **42,** 339–341; 10.1107/S0021889808042726 (2009).

47. Groom, C. R., Bruno, I. J., Lightfoot, M. P. & Ward, S. C. The Cambridge Structural Database. *Acta Crystallogr. B* **72,** 171–179; 10.1107/S2052520616003954 (2016).

48. Balos, V., Kim, H., Bonn, M. & Hunger, J. Dissecting Hofmeister Effects: Direct Anion-Amide Interactions Are Weaker than Cation-Amide Binding. *Angew. Chem. Int. Ed. Engl.* **55,** 8125–8128; 10.1002/anie.201602769 (2016).

49. Balos, V. *et al.* Macroscopic conductivity of aqueous electrolyte solutions scales with ultrafast microscopic ion motions. *Nat. Commun.* **11,** 1611; 10.1038/s41467-020-15450-2 (2020).

50. J. Barthel, R. Buchner, B. Wurm. *J. Mol. Liq.,* 51–69 (2002).

51. Ensing, W., Hunger, J., Ottosson, N. & Bakker, H. J. On the Orientational Mobility of Water Molecules in Proton and Sodium Terminated Nafion Membranes. *J. Phys. Chem. C* **117,** 12930–12935;

10.1021/jp312623p (2013).

52. Blackham, D. V. & Pollard, R. D. An improved technique for permittivity measurements using a coaxial probe. *IEEE Trans. Instrum. Meas.* **46,** 1093–1099; 10.1109/19.676718 (1997).

53. Gregory, A. P. & Clarke, R. N. Dielectric metrology with coaxial sensors. *Meas. Sci. Technol.* **18,** 1372–1386; 10.1088/0957-0233/18/5/026 (2007).

54. Buchner, R. & Hefter, G. Interactions and dynamics in electrolyte solutions by dielectric spectroscopy. *Phys. Chem. Chem. Phys.* **11,** 8984–8999; 10.1039/b906555p (2009).

55. Bard, A. J. & Faulkner, L. R. *Electrochemical Methods: Fundamentals and Applications. 2nd ed.* (John Wiley & Sons, Hoboken, N.J., 2001).

56. Scholz, F. *et al. Electroanalytical Methods* (Springer Berlin Heidelberg, Berlin, Heidelberg, 2010).

57. Rupp, A. *et al.* Size matters! On the way to ionic liquid systems without ion pairing. *Chem. Eur. J.* **20,** 9794–9804; 10.1002/chem.201400168 (2014).

58. O'Toole, T. R., Younathan, J. N., Sullivan, B. P. & Meyer, T. J. 1,2-Difluorobenzene: a relatively inert and noncoordinating solvent for electrochemical studies on transition-metal complexes. *Inorg. Chem.* **28,** 3923–3926; 10.1021/ic00319a032 (1989).

59. Eberson, L. & Schäfer, H. (eds.). *Organic Electrochemistry* (Springer, Berlin, Heidelberg, 1971).

60. Adams, R. N. *Electrochemistry at solid electrodes.* 6th ed. (Marcel Dekker Inc., New York, 1969).

61. Izutsu, K. *Electrochemistry in nonaqueous solutions* (Wiley-VCH, Weinheim, 2002).

62. Andreades, S. & Zahnow, E. W. Anodic cyanations of aromatic compounds. *J. Am. Chem. Soc.* **91,** 4181–4190; 10.1021/ja01043a028 (1969).

63. G. Cauquis & D. Serve. NM. *Bull. Soc. Chim. France,* 302 (1966).

64. Nelson, R. F. & Adams, R. N. Propylene carbonate: a versatile solvent for electrochemistry and EPR. *J. Electroanal. Chem. Interfacial Electrochemistry* **13,** 184–187; 10.1016/0022-0728(67)80114-1 (1967).

65. J. P. Billon. AN. *Bull. Soc. Chim. France,* 863 (1962).

66. J. Perichon, R. B. THF. *Bull. Soc. Chim. France,* 1279 (1968).

67. Connelly, N. G. & Geiger, W. E. Chemical Redox Agents for Organometallic Chemistry. *Chem. Rev.* **96,** 877–910; 10.1021/cr940053x (1996).

68. Lide, D. R. (ed.). *CRC Handbook of Chemistry and Physics.* 97th ed. (CRC Taylor & Francis, Boca Raton, FL, USA, 2017).

69. Maier, J. P. & Thommen, F. Fluorescence quantum yields and lifetimes of fluorobenzene cations in selected levels of their B̃ and C̃ states determined by photoelectron-photon coincidence spectroscopy. *Chem. Phys.* **57,** 319–332; 10.1016/0301-0104(81)80211-X (1981).

70. Kochi, J. K. Inner-sphere electron transfer in organic chemistry. Relevance to electrophilic aromatic nitration. *Acc. Chem. Res.* **25,** 39–47; 10.1021/ar00013a006 (1992).

71. Ligare, M. Classical thermodynamics of particles in harmonic traps. *Am. J. Phys.* **78,** 815–819; 10.1119/1.3417868 (2010).

72. York, D. M. & Karplus, M. A Smooth Solvation Potential Based on the Conductor-Like Screening Model. *J. Chem. Phys. A* **103,** 11060–11079; 10.1021/jp992097l (1999).

73. Barone, V. & Cossi, M. Quantum Calculation of Molecular Energies and Energy Gradients in Solution by a Conductor Solvent Model. *J. Phys. Chem. A* **102,** 1995–2001; 10.1021/jp9716997 (1998).

74. Klamt, A. & Schüürmann, G. COSMO: a new approach to dielectric screening in solvents with explicit expressions for the screening energy and its gradient. *J. Chem. Soc., Perkin Trans. 2,* 799–805; 10.1039/P29930000799 (1993).

75. Tomasi, J., Mennucci, B. & Cammi, R. Quantum mechanical continuum solvation models. *Chem. Rev.* **105,** 2999–3093; 10.1021/cr9904009 (2005).

76. Helgaker, T., Klopper, W., Koch, H. & Noga, J. Basis-set convergence of correlated calculations on water. *J. Chem. Phys.* **106,** 9639–9646; 10.1063/1.473863 (1997).

77. Bär, R., Heinis, T., Nager, C. & Junger, M. Photoionization of ferrocene. *Chemical Physics Letters* **91,** 440–442; 10.1016/0009-2614(82)83086-8 (1982).

78. Bastide, J., Hall, D., Heilbronner, E., Maier, J. P. & Plevey, R. G. He (Iα) photoelectron spectra of some higher aromatic perfluoro compounds. *Journal of Electron Spectroscopy and Related Phenomena* **16,** 205–208; 10.1016/0368-2048(79)85020-3 (1979).

79. Kremer, F. & Schönhals, A. (eds.). *Broadband Dielectric Spectroscopy* (Springer Berlin Heidelberg, Berlin, Heidelberg, 2003).

80. Buchner, R. What can be learnt from dielectric relaxation spectroscopy about ion solvation and association? *Pure Appl. Chem.* **80,** 1239–1252; 10.1351/pac200880061239 (2008).

81. Hunger, J., Stoppa, A., Thoman, A., Walther, M. & Buchner, R. Broadband dielectric response of dichloromethane. *Chem. Phys. Lett.* **471,** 85–91; 10.1016/j.cplett.2009.02.024 (2009).

82. Samojłowicz, C. *et al.* The doping effect of fluorinated aromatic solvents on the rate of ruthenium-catalysed olefin metathesis. *Chem. Eur. J.* **17,** 12981–12993; 10.1002/chem.201100160 (2011).

83. Shiflett, M. B. & Yokozeki, A. Liquid−Liquid Equilibria in Binary Mixtures Containing Fluorinated Benzenes and Ionic Liquid 1-Ethyl-3-methylimidazolium Bis(trifluoromethylsulfonyl)imide. *J. Chem. Eng. Data* **53,** 2683–2691; 10.1021/je8006474 (2008).

84. Zhao, Y. & Truhlar, D. G. The M06 suite of density functionals for main group thermochemistry, thermochemical kinetics, noncovalent interactions, excited states, and transition elements: two new functionals and systematic testing of four M06-class functionals and 12 other functionals. *Theor. Chem. Acc.* **120,** 215–241; 10.1007/s00214-007-0310-x (2008).

85. Hunger, J., Tielrooij, K.-J., Buchner, R., Bonn, M. & Bakker, H. J. Complex formation in aqueous trimethylamine-N-oxide (TMAO) solutions. *J. Phys. Chem. B* **116,** 4783–4795; 10.1021/jp212542q (2012).

86. Darges, G. & Müller-Warmuth, W. NMR Relaxation in Solutions of Six Fluorinated Benzenes Containing a Free Radical. *Ber. Bunsen Phys. Chem.* **85,** 1018–1021; 10.1002/bbpc.19810851116 (1981).

87. Madden, P. & Kivelson, D. A Consistent Molecular Treatment of Dielectric Phenomena. In *Advances in Chemical Physics,* edited by I. Prigogine & S. A. Rice (John Wiley & Sons, Inc, Hoboken, NJ, USA, 1984),

pp. 467–566.

88. Pummer, W. J., Wall, L. A. & Florin, R. E. *Aromatic fluorocarbons* (1961).

89. Dias, A. M. A. *et al.* Densities and Vapor Pressures of Highly Fluorinated Compounds. *J. Chem. Eng. Data* **50,** 1328–1333; 10.1021/je050056e (2005).
